# Supplementary material for: Effect of pragmatic versus explanatory interventions on medication adherence in people with cardiometabolic conditions: a systematic review and meta-analysis
Source: BMJ Open. 2020 Jul 23;10(7):e036575. doi: 10.1136/bmjopen-2019-036575 (PMC7380877; doi:10.1136/bmjopen-2019-036575)
Supplement: Supplementary data [file bmjopen-2019-036575supp001.pdf]

Online only supplement

Supplementary eTable 1 – Medline Search Strategy

Supplementary eTable 2 – Definitions of Intervention components

Supplementary eTable 3 – Characteristics of included studies

Supplementary eFigure 1– PRECIS-2 graphs of trials identified as pragmatic

Supplementary eFigure 2 – PRECIS-2 graphs of trials identified as explanatory

Supplementary eFigure 3. Average PRECIS-2 wheel domain scores for studies identified as pragmatic or explanatory

Supplementary eFigure 4 - Forest plot of pooled standardised mean differences for medication adherence, stratified by PRECIS score

Supplementary eTable 4 - Meta-regression results showing the effects of study level covariates on medication adherence

Supplementary eTable 5 - Results of sub-group analyses

Supplementary eFigure 5 - Funnel plot for the meta-analysis of standardised mean differences

Supplementary eFigure 6. Funnel plot for the meta-analyses of odds ratios

Supplementary eTable 6 - Number and proportion of texts which report insufficient data for PRECIS-2 domain scoring

Supplementary eTable 1 – Medline Search Strategy

1	exp patient compliance/ or patient* adher*.mp.
2	exp medication compliance/ or medication* adher*.mp.
3	((patient* or medication* or medicine* or drug* or therap* or treatment*) adj2 (adher* or concordance or non adher* or nonadher* or compliance or non compliance or noncompliance or refusal)).mp. [mp=title, abstract, original title, name of substance word, subject heading word, floating sub-heading word, keyword heading word, protocol supplementary concept word, rare disease supplementary concept word, unique identifier, synonyms]
4	treatment refusal/ or treatment* refusal.mp.
5	exp *non insulin dependent diabetes mellitus/ or Type 2 diabet*.mp.
6	T2D*.mp.
7	((non insulin or noninsulin) adj dependent diabet*).mp. [mp=title, abstract, original title, name of substance word, subject heading word, floating sub-heading word, keyword heading word, protocol supplementary concept word, rare disease supplementary concept word, unique identifier, synonyms]
8	NIDD*.mp.
9	Diabetes Mellitus/ or diabet*.mp.
10	*cardiovascular disease/ or cardiovascular diseas*.mp. or CVD.mp. [mp=title, abstract, original title, name of substance word, subject heading word, floating sub-heading word, keyword heading word, protocol supplementary concept word, rare disease supplementary concept word, unique identifier, synonyms]
11	((heart or cardiac or cardiovascular or coronary) adj2 (disease* or disorder* or failure*)).mp. [mp=title, abstract, original title, name of substance word, subject heading word, floating sub-heading word, keyword heading word, protocol supplementary concept word, rare disease supplementary concept word, unique identifier, synonyms]
12	*heart arrhythmia/ or arrhythmia*.mp.
13	*atrial fibrillation/ or atrial fibrillat*.mp.
14	*heart infarction/ or myocardial infarct*.mp.
15	heart failure.mp. or *heart failure/
16	*ischemic heart disease/ or *Myocardial Ischemia/ or myocard* isch*.mp.
17	angina.mp. or *angina pectoris/

18	*cardiomyopathy/ or cardiomyopath*.mp.
19	*coronary artery disease/ or coronary artery diseas*.mp.
20	peripheral artery diseas*.mp. or *peripheral occlusive artery disease/ or *peripheral vascular disease/
21	stroke.mp. or *cerebrovascular accident/
22	*transient ischemic attack/ or transient isch* attack.mp.
23	TIA.mp.
24	controlled clinical trial.mp. or controlled clinical trial/
25	"randomized controlled trial (topic)"/ or RCT.mp.
26	clinical trial/
27	(randomi?ed controlled adj2 trial).mp. [mp=title, abstract, original title, name of substance word, subject heading word, floating sub-heading word, keyword heading word, protocol supplementary concept word, rare disease supplementary concept word, unique identifier, synonyms]
28	1 or 2 or 3 or 4
29	5 or 6 or 7 or 8 or 9 or 10 or 11 or 12 or 13 or 14 or 15 or 16 or 17 or 18 or 19 or 20 or 21 or 22 or 23
30	24 or 25 or 26 or 27
31	28 and 29 and 30
32	limit 31 to (english language and humans and yr="2013 - 2018")

**Supplementary eTable 2 – Definitions of Intervention components**	
Behavioural/educational	Educational interventions comprising of motivational interviewing, counselling, group or individual education sessions on disease, medication and general self-management and exercise classes. In general, these studies comprised of some form of structured education and or a motivational interviewing component.
Telemonitoring	Out-patient appointments were changed for telemonitoring visits, participants were contacted at regular intervals using web-based feedback, or interactive voice recognition when patients were due or overdue their medications
Collaborative care	Enhanced communication between hospital and homecare teams
Simplification of drug regimen	Simplification of drug regimen by using a polypill
Intensified patient care	Patients were contacted more regularly than usual or drug availability and compliance was more closely monitored
Personalised drug dispensing	Weekly contact via telephone or home visits with the participant to check drug availability and adherence
Multi-faceted	Varying combinations of the aforementioned interventions with electronic pill caps and pill boxes as additional interventions
Classification of intervention types were determined based on guidance obtain from previous adherence research[1, 2]	

Supplementary eTable 3 – **Characteristics of included studies**

First Author, year	Country	Sample size (analysed)<br>Duration<br>Data collection points	Condition	Key intervention detail	Medication adherence measure (adherence definition or method of reporting)
Al Haj Mohd, 2016[(25)]	United Arab Emirates	446 (446)<br>6 months<br>0 & 6 month	T2DM	Multifaceted intervention strategy<br>• Education - 30 minute session<br>• Intensified patient care - weekly phone calls for 3 months)	Self-report - MMAS-8<br>Higher scores = better adherence
Barker Collo 2015[(26)]	Australia	386 (326)<br>12 months<br>0, 3, 6, 9 & 12 months	Stroke/TIA	Behavioural/Educational<br>• 4 x motivational interviewing sessions which occurred either face to face or via telephone, initial interview 60-90mins, remainder 30 minutes	Self-report – asked whether in the last 7 days they had taken all their medication as prescribed, indicate number of doses/pills missed, reason for missed doses and any side effects if noticed.<br>Cross checked self-report with pharmacy data for validity.
Boyne, 2014[(27)]	Netherlands	382 (382)<br>12 months<br>0, 3, 6 12 months	Heart failure	Tele-monitoring/telemedicine<br>• Replace 2 of the 4 yearly outpatient appointments with tele-monitoring	Self-report – European heart failure self-care behavior scale
Buhse, 2018[(28)]	Germany	279 (279)<br>6 months<br>0 & 6 months	T2DM	Behavioural/Educational<br>• Decision aid about primary prevention of MI<br>• 90 minute group education session about complication prevention<br>• Patient document with defined treatment goals	Pharmacy data<br>Self-report via telephone interview
Caetano, 2018[(29)]	Portugal	709 (702)<br>6 months<br>0 & 6 months	T2DM	Behavioural/Educational<br>• Patient given one of 3 educational leaflets on diabetes, diabetes treatment or physical activity	MAT scale<br>Higher scores=better adherence
Cao, 2017[(47)]	China	236 (236)<br>90 days	Coronary heart disease	Collaborative care<br>• Enhanced communication between hospital and home care team	Self-report - MMAS-8<br>Higher scores = better adherence

		0, 30 & 90 days			
Carrasquillo, 2017[30]	United States	300 (215)<br><br>12 months<br><br>0 & 12 months	T2DM	Behavioural/Educational<br>• Education - (mentoring by a community health worker approx. 4 home visits and 12 calls)<br>• Education – monthly group education sessions<br>• Behavioral – Bimonthly group exercise classes	Self-report - MMAS-8<br>Higher scores = better adherence
Castellano, 2014[24]	Argentina, Italy, Spain & Paraguay	695 (594)<br><br>9 months<br><br>0, 1, 4, 9 months	Myocardial infarction	Simplification of drug regimen<br>• Fixed dose combination polypill containing Aspirin, Simvastatin, Ramipril	Pill count<br>Self-report – Morisky-Green-Levine Medication Adherence Scale
Chung, 2014[48]	Malaysia	241 (241)<br><br>12 months<br><br>0, 4, 8 12 months	T2DM	Multifaceted intervention strategy<br>• Education – on diabetes, hypertension, hyperlipidemia and medications<br>• Medication aids – pill box and blood glucose monitoring equipment<br>• Intensified patient care – medication reviews and monthly follow up calls	Self-report - MMAS-8 (Revised Malaysian version)<br>Higher scores = better adherence
Crowley, 2013[31]	United States	359 (329)<br><br>12 months<br><br>0, 3, 6, 9 & 12 months	T2DM	Multifaceted intervention strategy<br>• Education – monthly self-management education telephone calls<br>• Personalised medication management – nurse and primary care provider contact at 3 monthly intervals to discuss medication adherence and possible medication changes	Self-report Morisky-Green-Levine Medication Adherence Scale
Du, 2016[32]	China	979 (964)<br><br>36 months<br><br>0 & 36 months	Percutaneous coronary intervention	Multifaceted intervention strategy<br>• Intensified patient care/education/personalized medication management - regular telephone follow ups at 1, 2, 3, 6, 12 & 36 months to discuss medication usage, provide health education and disease prevention strategies and schedule lab tests and treatment adjustments where necessary	Self-report Morisky-Green-Levine Medication Adherence Scale<br>Lower score = better adherence<br>(Score 0-2 = adherent)
El Touky, 2017[33]	Saudi Arabia	321 (276)		Behavioural/Educational intervention	Pill count >80% classed as adherent

		12 months<br><br>0, 1, 3, 6, 9, & 12 months	Acute coronary syndrome	• Educated patients on several aspects of coronary artery disease		
Graumlich, 2016[34]	United States	674 (674)<br><br>12 months<br><br>0, immediately following intervention, 3 & 6 months	T2DM	Medication monitoring table<br>• Multi-media tool embedded within the electronic medical record that aims to:<br>1. Promote patient knowledge<br>2. Support collaborative medication planning		Self-report -PMAQ -(patient medication adherence questionnaire)<br>Four questions were asked regarding whether the patient had missed taking a dose yesterday, the day before yesterday, 3 days ago, and over the past weekend. Patients were rated as having proper adherence if they self-reported no missed doses in this time period
Hedegaard, 2014[35]	Denmark	211 (203)<br><br>12 months<br><br>0, 3, 6, 9 & 12 months	Stroke/ TIA	Multifaceted intervention strategy<br>• Personalized medication management-medication review by pharmacist and advice on relevant drug related problems<br>• Behavioral – 3 x 30 minute patient motivational interview to support adherence and lifestyle change		Pharmacy data - MPR >0.8
Ho, 2014[36]	United States	253 (241)<br><br>12 months<br><br>0 & 12 months	Acute coronary syndrome	Multifaceted intervention strategy<br>• Personalized medication management - Medication reconciliation and tailoring by pharmacist and provision of pill box<br>• Education - Medication education<br>• Collaborative care – Pharmacist contacted patients<br>• Telemedicine – Medication reminder and refill calls		Pharmacy refill data >0.8
Jeong, 2018[37]	South Korea	338 (338)<br><br>24 weeks	T2DM	Tele-monitoring/telemedicine (Home telemonitoring composed of web based	Tele-monitoring/telemedicine Reminders (Home telemonitoring	Does not report how medication adherence was assessed.

		0 & 24 weeks		feedback and education with conventional management)	with remote management)	
Jia, 2017[49]	China	669 (669)<br><br>36 months<br><br>0, 1, 3, 6, 12 & 36 months	Percutaneous coronary intervention	Intensified patient care<br>• Regular phone calls with face to face appointments scheduled as necessary		Self-report - Morisky-Green-Levine Medication Adherence Scale<br>Lower score = better adherence<br>(Score 0-2 = adherent)
Kronish, 2014[38]	United States	600 (600)<br><br>6 months<br><br>0 & 6 months	Stroke/ TIA	Behavioural/Educational intervention<br>• 6 x 90 minute weekly peer led workshops about self-management skills and medication adherence		Self-report -MMAS-8<br>Higher scores = better adherence
Lin, 2017[50]	Iran	288 (288)<br><br>18 months<br><br>0, 6, 12 & 18 months	Coronary artery bypass grafting	Multifaceted intervention strategy<br>• Educational - 3 x 60 minute weekly education sessions<br>• Behavioral – 5 x 50 minute weekly motivational interviewing<br>• Telemedicine – 4 SMS reminders sent each month, content varied on a monthly basis		Self-report – MARS (5 item)<br>Score range 5-25- Higher scores = better adherence<br>Pharmacy data
Marin, 2015[56]	Argentina	467 (459)<br><br>12 months<br><br>0 & 12 months	T2DM	Personalised medication management<br>• Weekly contact with patient to check drug availability and compliance either by phone or home visit		Self-report - Morisky-Green-Levine Medication Adherence Scale<br>Higher scores = better adherence
Marquez, 2018[39]	Spain	726 (625)<br><br>18 months<br><br>0, 6 & 12 months	Atrial Fibrillation	Multifaceted intervention strategy<br>• Education – healthcare and patient discussion of an educational booklet about medication and adherence<br>• Medication aid – medication reminder calendar		MEMs
Meng, 2014[23]	Germany	471 (425)<br><br>12 months	Coronary heart disease	Behavioural/Educational intervention<br>• 5 x 45minute patient centered self-management group education program		Self-report – MARS-D<br>Higher scores = better adherence

		Admission, discharge, 6 & 12 months			
Meng, 2016[(40)]	Germany	513 (449)<br><br>12 months<br><br>Admission, discharge, 6 & 12 months	Heart failure	Behavioural/Educational intervention<br>• 5 x 60minute patient centered self-management group education program	Self-report – MARS-D<br>Higher scores = better adherence
Peng, 2014[(51)]	China	3821 (3330)<br><br>12 months<br><br>Discharge, 6, 9 & 12 months	Stroke/ TIA	Behavioural/Educational intervention<br>• Lifestyle modification - diet, exercise, smoking and risk factor control	Does not report
Pladevall *2015*[(41)]	United states	1692 (1512)<br><br>18 months<br><br>0, 6, 12 & 18 months	T2DM	Behavioural/Educational intervention<br>• Adherence information provided to physicians to relay to patients<br>• Above plus motivational interviewing	Pharmacy data -PDC
Rinfret, 2013[(42)]	Canada	300 (300)<br><br>12 months<br><br>0 & 12 months	Percutaneous coronary intervention	Intensified patient care<br>• Telephone follow-up within 7 days of DES implantation and at 1, 6, and 9 months	Pharmacy refill data
Samtia, 2013[(54)]	Pakistan	348 (348)<br><br>5 months<br><br>0 & 5 months	T2DM	Behavioural/Educational<br>• Continual education by pharmacists about effects of complications, medication adherence, diet, exercise, foot care, smoking cessation and HbA1c	Self-report – adherent yes/no
Schou, 2014[(43)]	Denmark	921 (920)<br><br>13-72 months	Heart failure	Multi-faceted intervention strategy<br>• Intensified patient care – regular drug monitoring and access to regular telephone consultations	Pharmacy data - PDC >0.8

		Every 1-3 months		• Education – occurred if adherence decreased during drug monitoring	
Schwalm, 2015[44]	Canada	852 (852)<br><br>12 months<br><br>0, 3 & 12 months	Myocardial infarction	Behavioural/Educational<br>• Personalised letters at 1, 5, 8 and 11 months after angiogram which stated the importance and role of each medication.<br>• Prompt to talk to physician and pharmacist about meds	• Self-report – yes/no<br>• Self-report Morisky-Green-Levine Medication Adherence Scale perfect score %
Su, 2016[52]	China	1275 (1187)<br><br>12 months<br><br>0 & 12 months	Stroke/ TIA	Multi-faceted intervention strategy<br>• Education 3 x 30 minute sessions and easy to understand manual<br>• Intensified patient care – physician contact at 1, 3 and 6 months to monitor progress	Does not report
Vollmer, 2014[45]	United States	21752 (21752)<br><br>12 months<br><br>0 & 12 months	T2DM and/or CVD	Telemonitoring/telemedicine<br>• Interactive voice recognition (IVR) calls when pts due or overdue medications<br>• Enhanced IVR as above plus letter, EMR feedback, mailed materials	Pharmacy data - PDC >0.8
Volpp, 2017[46]	United States	1509 (1503)<br><br>12 months<br><br>0 & 12 months	Myocardial infarction	Multifaceted intervention strategy<br>• Medication aids - Electronic pill bottles used in place of regular pill bottles (Glow caps)<br>• Financial incentives – Patients included in a daily lottery with the potential to win $5<br>• Medication support partner – information on patients adherence shared with a support partner whose role it was to offer support and encouragement<br>• Intensified patient care – engagement advisor and social workers to provide support on adherence issues	Pharmacy data - PDC
Xavier, 2016[55]	India	805 (750)<br><br>12 months	Acute coronary syndrome	Multifaceted intervention strategy<br>• Behavioral/educational - Mentoring by a community health worker<br>• Medication aids - medication reminder calendar and	Pharmacy data -Composite medical adherence score >80%

		0 & 12 months		patient dairy which contained details on drugs, how to take them and benefits.	
Xin, 2015[53]	China	240 (227)<br>12 months<br>0 & 12 months	T2DM	Behavioural/Educational<br>• Individualised education<br>• Group education/activities<br>• Telephone counselling	• Prescription refill claims – 80-115<br>• Morisky-Green – perfect score %

Abbreviations- T2DM: type 2 diabetes; TIA: Transient Ischemic Attack; MMAS-8; Morisky Medication Adherence Scale (8-item); PDC: proportion of days covered; MARS-D: Medication Adherence Report Scale (German version); MPR: Medication Possession Ratio; MEMs: medication event monitoring system; 0 months =baseline

Supplementary eFigure 1– PRECIS-2 graphs of trials identified as pragmatic

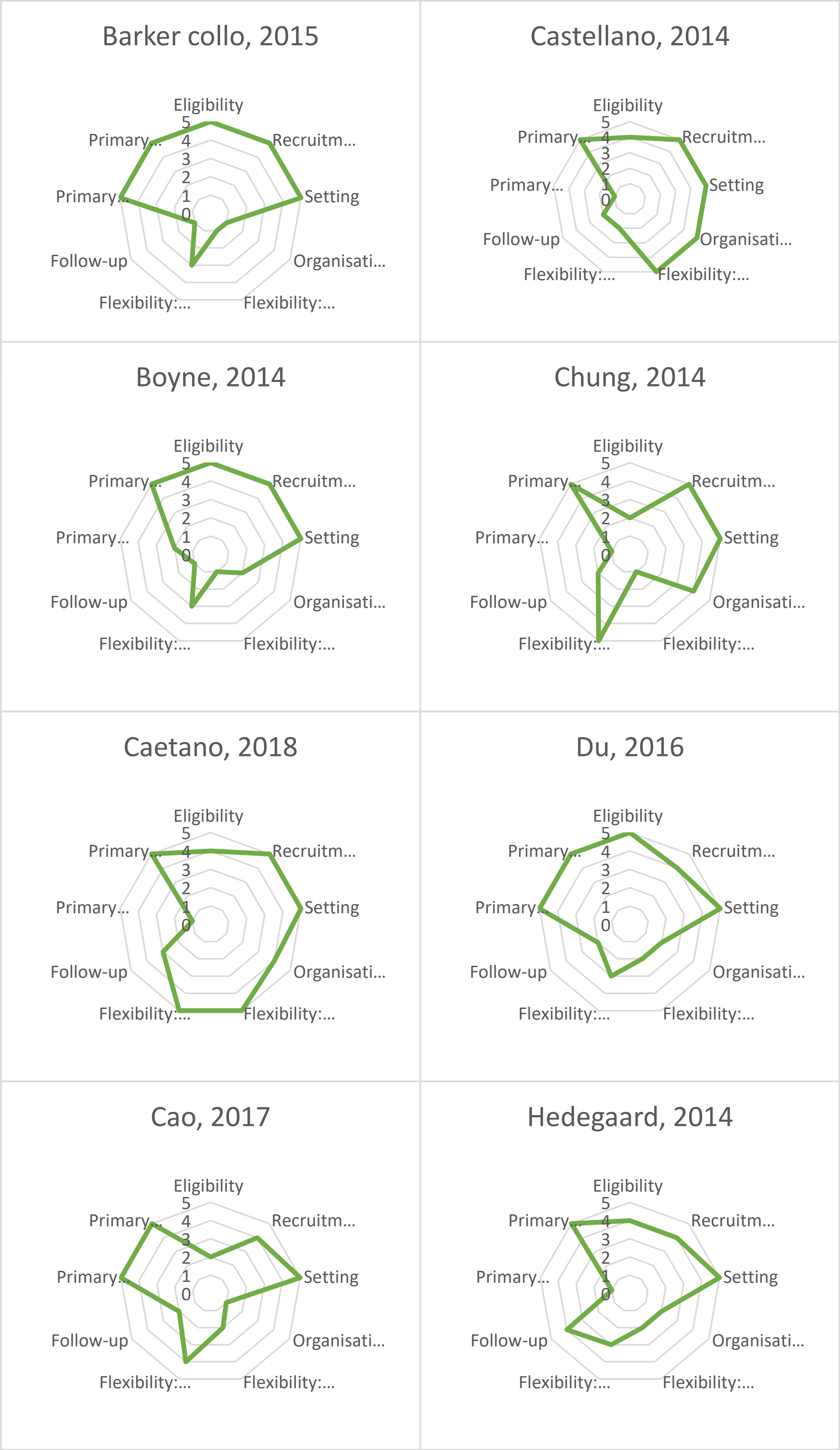

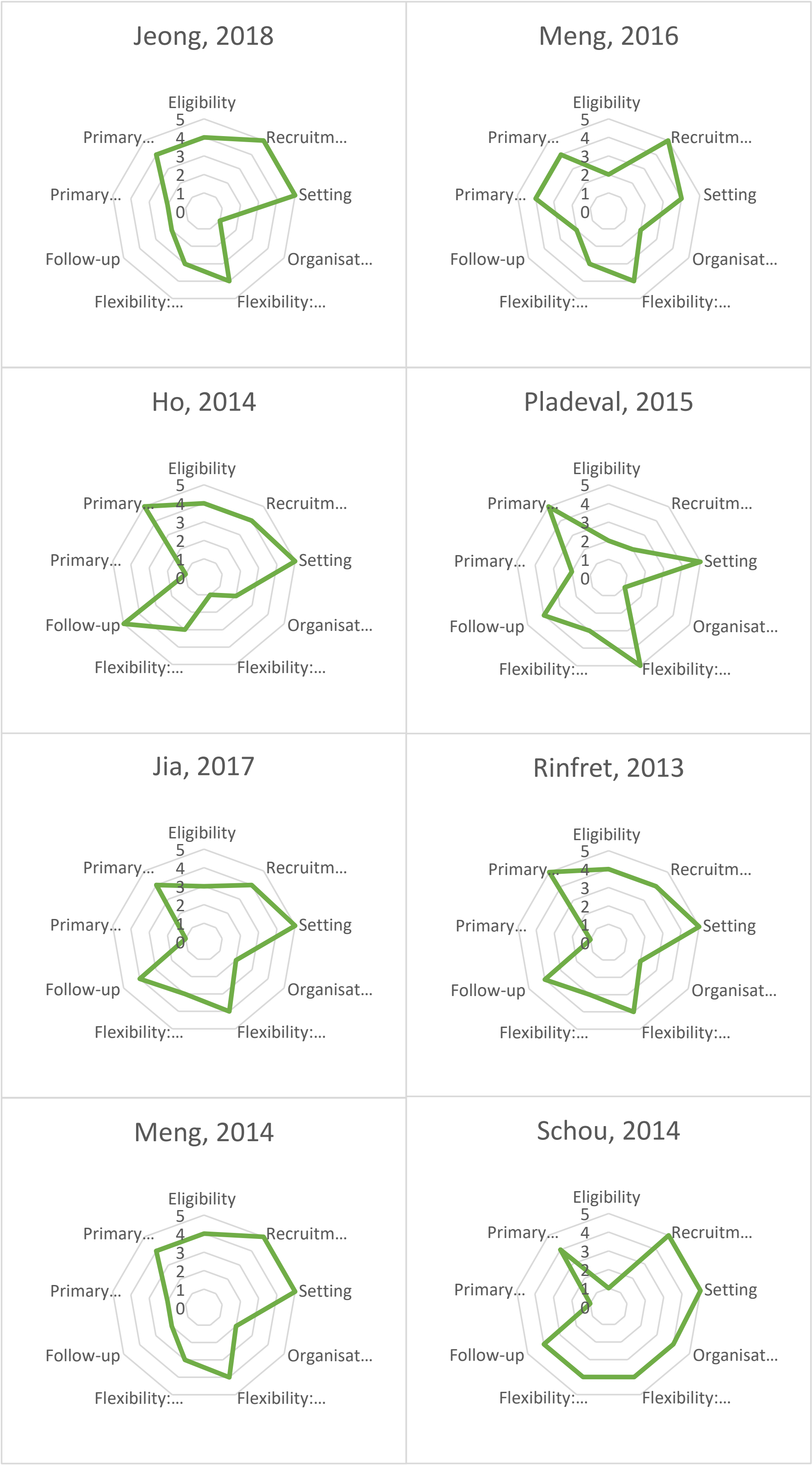
Jeong, 2018
Meng, 2016
Ho, 2014
Pladeval, 2015
Jia, 2017
Rinfret, 2013
Meng, 2014
Schou, 2014
Eligibility
Recruitm...
Setting
Organisat...
Flexibility:...
Flexibility:...
Follow-up
Primary...
Primary...
0
1
2
3
4
5

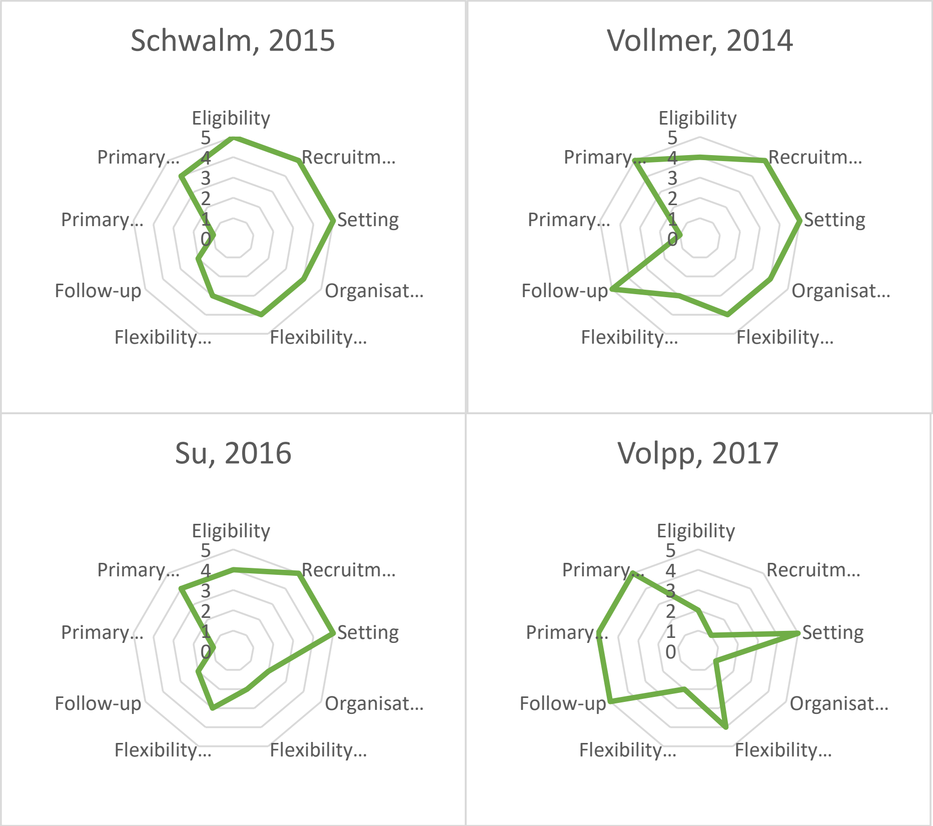
Schwalm, 2015
Vollmer, 2014
Su, 2016
Volpp, 2017
Eligibility
Recruitm...
Setting
Organisat...
Flexibility...
Flexibility...
Follow-up
Primary...
Primary...
5
4
3
2
1
0

Supplementary eFigure 2 – PRECIS-2 graphs of trials identified as explanatory

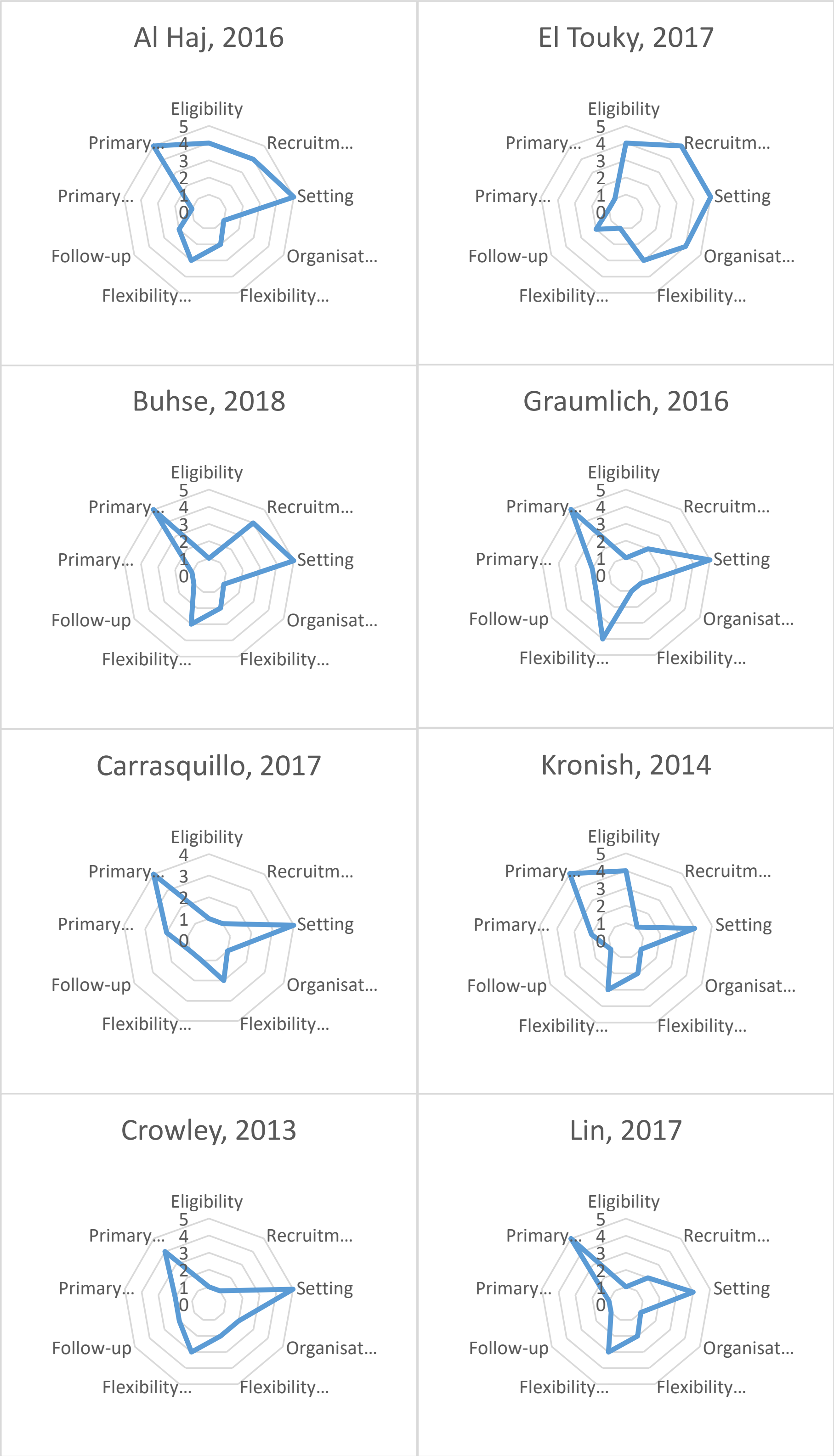

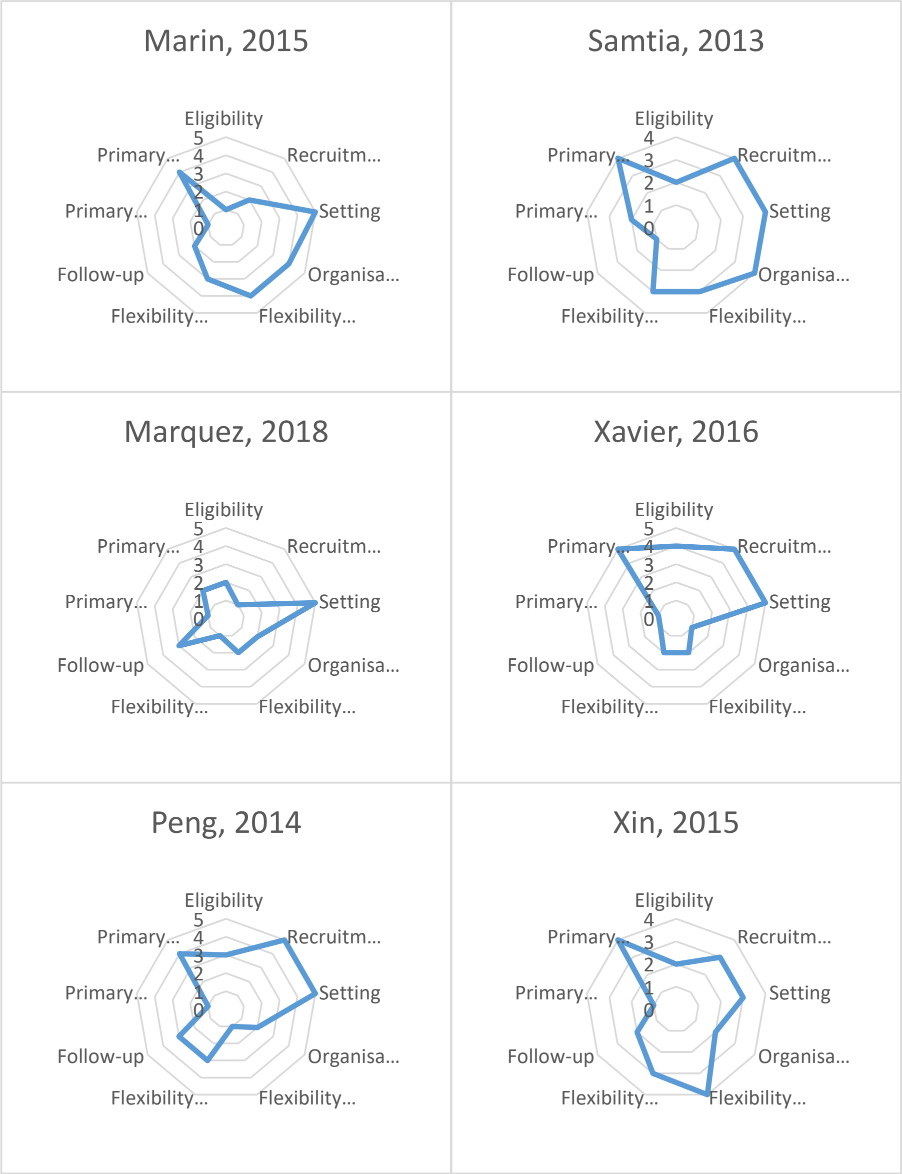
Marin, 2015
Eligibility
Recruitm…
Setting
Organisa…
Flexibility…
Flexibility…
Follow-up
Primary…
Primary…
0
1
2
3
4
5
Samtia, 2013
Eligibility
Recruitm…
Setting
Organisa…
Flexibility…
Flexibility…
Follow-up
Primary…
Primary…
0
1
2
3
4
Marquez, 2018
Eligibility
Recruitm…
Setting
Organisa…
Flexibility…
Flexibility…
Follow-up
Primary…
Primary…
0
1
2
3
4
5
Xavier, 2016
Eligibility
Recruitm…
Setting
Organisa…
Flexibility…
Flexibility…
Follow-up
Primary…
Primary…
0
1
2
3
4
5
Peng, 2014
Eligibility
Recruitm…
Setting
Organisa…
Flexibility…
Flexibility…
Follow-up
Primary…
Primary…
0
1
2
3
4
5
Xin, 2015
Eligibility
Recruitm…
Setting
Organisa…
Flexibility…
Flexibility…
Follow-up
Primary…
Primary…
0
1
2
3
4

Supplementary eFigure 3. Average PRECIS-2 wheel domain scores for studies identified as pragmatic or explanatory

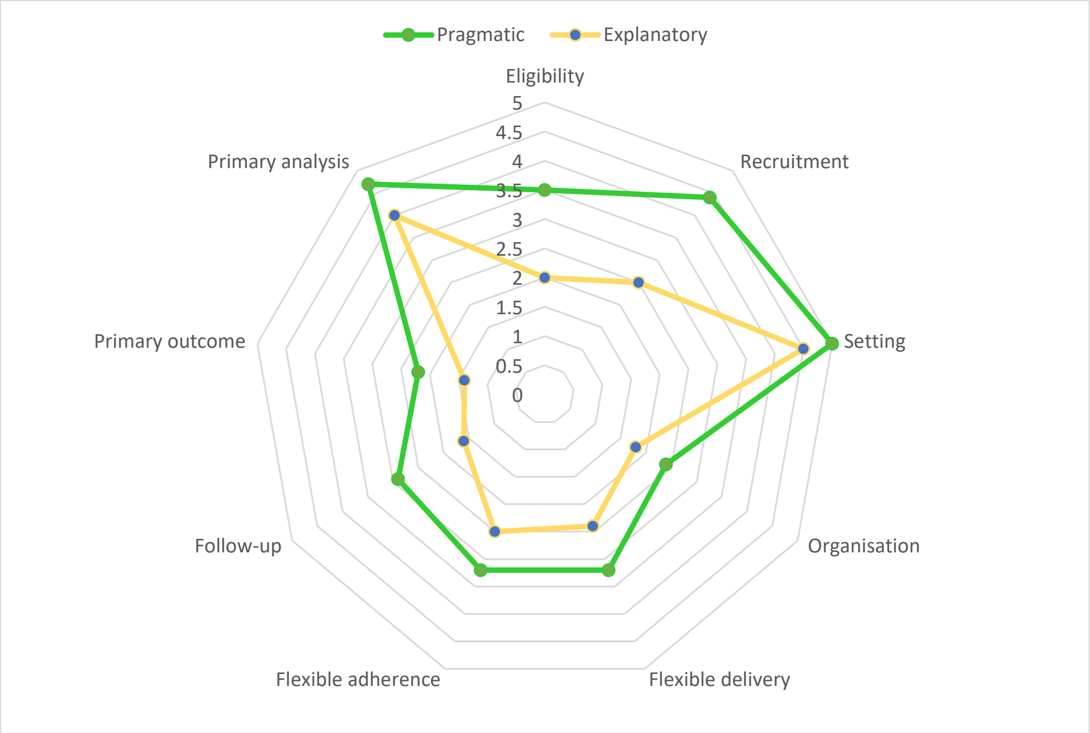

Supplementary eFigure 4 - Forest plot of pooled standardised mean differences for medication adherence, stratified by PRECIS score

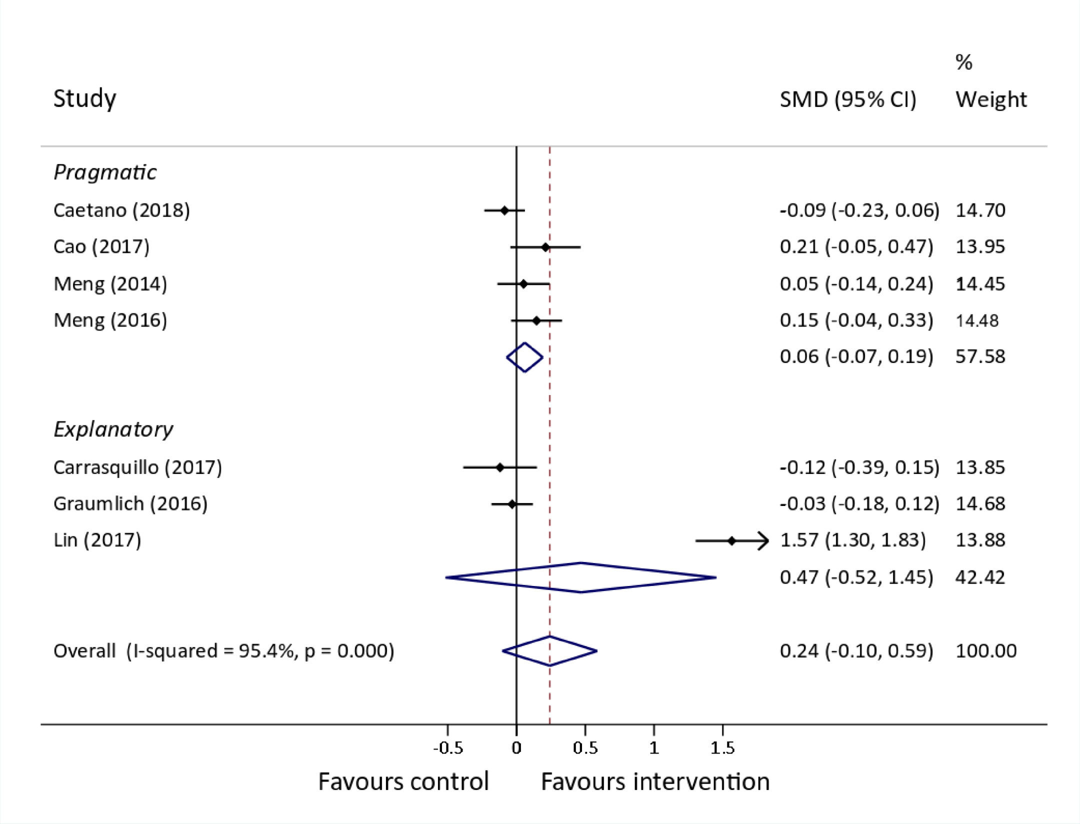

BMJ Publishing Group Limited (BMJ) disclaims all liability and responsibility arising from any reliance placed on this supplemental material which has been supplied by the author(s)

Supplementary eTable 4 - Meta-regression results showing the effects of study level covariates on medication adherence

	Coefficient (95% CI)	p-value
Odds Ratios		
Study length	0.01 (-0.01, 0.03)	0.408
Outcome (1^0 or 2^0)	0.01 (-0.39, 0.41)	0.964
Mean age	-0.02 (-0.06, 0.02)	0.317
Percent male	0.01 (-0.01, 0.02)	0.469
Disease	-0.09 (-0.42, 0.24)	0.581
Precis score	-0.02 (-0.07, 0.03)	0.367
Standardised mean differences		
Study length	0.07 (-0.04, 0.18)	0.162
Outcome (1^0 or 2^0)	-0.67 (-1.82, 0.48)	0.195
Mean age	0.06 (-0.01, 0.12)	0.071
Percent male	0.004 (-0.03, 0.04)	0.760
Disease	0.57 (-0.52, 1.65)	0.237
Precis score	-0.03 (-0.12, 0.06)	0.429

Supplementary eTable 5 - Results of sub-group analyses

	N studies	Effect size (95% CI)	p-value*
Odds ratios of adherence			
**Explanatory**	9	1.69 (1.24, 2.31)	0.598
**Pragmatic**	13	1.53 (1.23, 1.89)	
**Self-reported adherence measure**	15	1.63 (1.33, 1.98)	0.568
**Objective adherence measure**	7	1.47 (1.03, 2.08)	
**Multi-faceted intervention**	8	2.05 (1.62, 2.60)	0.010
**Single-faceted intervention**	14	1.28 (1.12, 1.46)	
SMD of adherence			
**Explanatory**	3	0.47 (-0.52, 1.45)	0.441
**Pragmatic**	4	0.06 (-0.07, 0.19)	
**Self-reported adherence measure**	7	*Not calculated*†	-
**Objective adherence measure**	0	*Not calculated*†	
**Multi-faceted intervention**	1	*Not calculated*†	-
**Single-faceted intervention**	6	*Not calculated*†	

*Comparing the effect size between the two sub-groups. †Pooled estimates by sub-group were not calculated where there was less than 2 studies to combine for one of the groupings

Supplementary eFigure 5 - Funnel plot for the meta-analysis of standardised mean differences

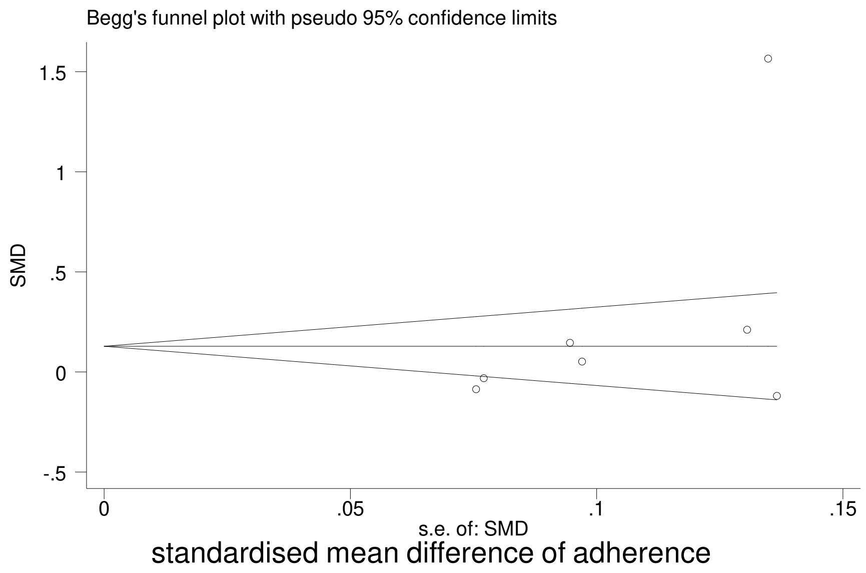

Supplementary eFigure 6 - Funnel plot for the meta-analyses of odds ratios

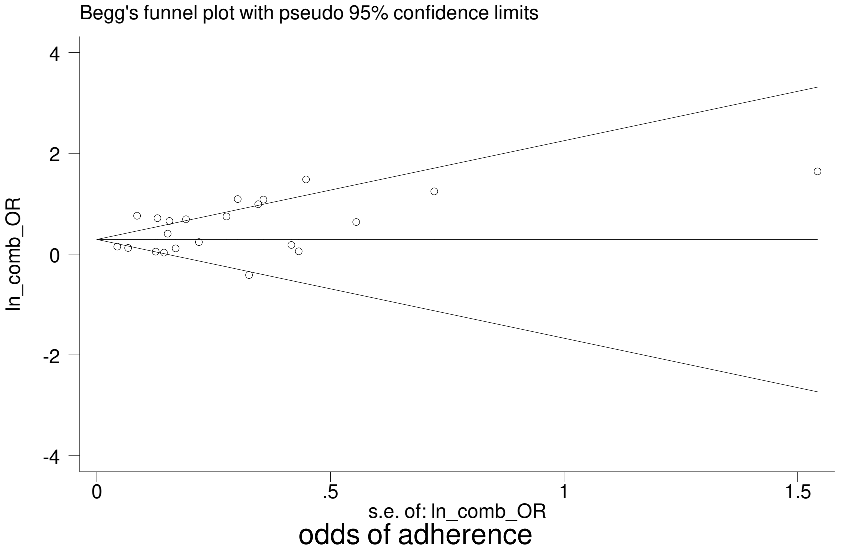

Supplementary eTable 6 - Number and proportion of texts which report insufficient data for PRECIS-2 domain scoring

Domains (n=9)	n	%
Eligibility	0	0
Recruitment	1	2.9
Setting	1	2.9
Organisation	0	0
Flexible delivery	2	5.9
Flexible Adherence	23	67.6
Follow-up	2	5.9
Primary outcome	0	0
Primary analysis	0	0
