# Supplementary material for: Effect of pragmatic versus explanatory interventions on medication adherence in people with cardiometabolic conditions: a systematic review and meta-analysis
Source: BMJ Open. 2020 Jul 23;10(7):e036575. doi: 10.1136/bmjopen-2019-036575 (PMC7380877; doi:10.1136/bmjopen-2019-036575)
Supplement: Author's manuscript [file bmjopen-2019-036575.draft_revisions.pdf]

BMJ Open is committed to open peer review. As part of this commitment we make the peer review history of every article we publish publicly available.

When an article is published we post the peer reviewers' comments and the authors' responses online. We also post the versions of the paper that were used during peer review. These are the versions that the peer review comments apply to.

The versions of the paper that follow are the versions that were submitted during the peer review process. They are not the versions of record or the final published versions. They should not be cited or distributed as the published version of this manuscript.

BMJ Open is an open access journal and the full, final, typeset and author-corrected version of record of the manuscript is available on our site with no access controls, subscription charges or pay-per-view fees (http://bmjopen.bmj.com).

If you have any questions on BMJ Open's open peer review process please email info.bmjopen@bmj.com

BMJ Open

Effect of pragmatic versus explanatory interventions on medication adherence in people with cardiometabolic conditions: a systematic review and meta-analysis

Journal:	*BMJ Open*
Manuscript ID	bmjopen-2019-036575
Article Type:	Original research
Date Submitted by the Author:	23-Dec-2019
Complete List of Authors:	Fitzpatrick, Claire; University of Leicester Diabetes Research Centre; University Hospitals of Leicester NHS Trust, Leicester Diabetes Centre<br>Gillies, Clare; University of Leicester Diabetes Research Centre; University Hospitals of Leicester NHS Trust, Leicester Diabetes Centre<br>Seidu, Samuel; University of Leicester Diabetes Research Centre; University Hospitals of Leicester NHS Trust, Leicester Diabetes Centre<br>Kar, Debasish; University of Sheffield Department of Oncology and Metabolism,<br>Ioannidou, Ekaterini; University of Leicester Diabetes Research Centre; University Hospitals of Leicester NHS Trust, Leicester Diabetes Centre<br>Davies, Melanie; University of Leicester Diabetes Research Centre, Diabetes Research Centre; University Hospitals of Leicester NHS Trust, Leicester Diabetes Centre<br>Patel, Prashanth; University of Leicester, Department of Cardiovascular Sciences; University Hospitals of Leicester NHS Trust, Department of Chemical Pathology and Metabolic Diseases<br>Gupta, Pankaj; University of Leicester, Department of Cardiovascular Sciences; University Hospitals of Leicester NHS Trust, Department of Chemical Pathology and Metabloic Diseases<br>Khunti, Kamlesh; University of Leicester Diabetes Research Centre; University Hospitals of Leicester NHS Trust, Leicester Diabetes Centre
Keywords:	DIABETES & ENDOCRINOLOGY, CARDIOLOGY, Diabetes & endocrinology < INTERNAL MEDICINE

SCHOLARONE™
Manuscripts

I, the Submitting Author has the right to grant and does grant on behalf of all authors of the Work (as defined in the below author licence), an exclusive licence and/or a non-exclusive licence for contributions from authors who are: i) UK Crown employees; ii) where BMJ has agreed a CC-BY licence shall apply, and/or iii) in accordance with the terms applicable for US Federal Government officers or employees acting as part of their official duties; on a worldwide, perpetual, irrevocable, royalty-free basis to BMJ Publishing Group Ltd ("BMJ") its licensees and where the relevant Journal is co-owned by BMJ to the co-owners of the Journal, to publish the Work in this journal and any other BMJ products and to exploit all rights, as set out in our licence.

The Submitting Author accepts and understands that any supply made under these terms is made by BMJ to the Submitting Author unless you are acting as an employee on behalf of your employer or a postgraduate student of an affiliated institution which is paying any applicable article publishing charge ("APC") for Open Access articles. Where the Submitting Author wishes to make the Work available on an Open Access basis (and intends to pay the relevant APC), the terms of reuse of such Open Access shall be governed by a Creative Commons licence – details of these licences and which Creative Commons licence will apply to this Work are set out in our licence referred to above.

Other than as permitted in any relevant BMJ Author's Self Archiving Policies, I confirm this Work has not been accepted for publication elsewhere, is not being considered for publication elsewhere and does not duplicate material already published. I confirm all authors consent to publication of this Work and authorise the granting of this licence.

For peer review only

ORIGINAL RESEARCH

Effect of pragmatic versus explanatory interventions on medication adherence in people with cardiometabolic conditions: a systematic review and meta-analysis

Author Names:
Claire Fitzpatrick MSc[1,2], Clare Gillies PhD[1,2], Samuel Seidu MD[1,2], Debasish Kar[3] MSc, Ekaterini Ioannidou MSc[1,2], Melanie Davies MD[1,2], Prashanth Patel FRCPath[4,5], Pankaj Gupta FRCPath[4,5], Kamlesh Khunti PhD[1,2]*

Claire Fitzpatrick & Clare Gillies contributed equally

[1] Diabetes Research Centre, University of Leicester, Leicester, UK,

[2] Leicester Diabetes Centre, University Hospitals of Leicester NHS Trust, Leicester General Hospital, Leicester, UK

[3] University of Sheffield, Sheffield, UK

[4] Department of Cardiovascular Sciences, University of Leicester, Leicester, UK

[5] Department of Chemical Pathology and Metabolic Diseases, University Hospitals of Leicester NHS Trust, Leicester, UK

Claire Fitzpatrick, (0000-0003-2150-8092), Researcher, Diabetes Research Centre, University of Leicester, Leicester General Hospital, Gwendolen Road, Leicester, LE5 4PW

Claire Gillies, (0000-0002-8417-9700), Lecturer in Medical Statistics, Diabetes Research Centre, University of Leicester, Leicester General Hospital, Gwendolen Road, Leicester, LE5 4PW

Samuel Seidu, (0000-0002-8335-7018) Primary Care Research Fellow, Diabetes Research Centre, University of Leicester, Leicester General Hospital, Gwendolen Road, Leicester, LE5 4PW

Debasish Kar, (0000-0002-1524-1312), Senior Clinical Lecturer (Honorary), University of Sheffield, Western bank, Sheffield, S10 2TN

Ekaterini Ioannidou, Research Assistant, Diabetes Research Centre, University of Leicester, Leicester General Hospital, Gwendolen Road, Leicester, LE5 4PW

Melanie Davies, (0000-0002-9987-9371), Professor of Diabetes Medicine, Diabetes Research Centre, University of Leicester, Leicester General Hospital, Gwendolen Road, Leicester, LE5 4PW

Prashanth Patel, (0000-0002-8981-2691), Consultant Metabolic Physician and Honorary Senior Lecturer and Co- Director National Centre for Adherence Testing, Department of Chemical Pathology and Metabolic Diseases, University of Leicester, Leicester Royal Infirmary, Infirmary Way, Leicester, LE1 5WW

Pankaj Gupta, (0000-0001-9481-6067), Consultant, Co- Director National Centre for Adherence Testing, Department of Chemical Pathology and Metabolic Diseases, University of Leicester, Leicester Royal Infirmary, Infirmary Way, Leicester, LE1 5WW

Corresponding author* - Kamlesh Khunti, (0000-0003-2343-7099) Professor of Primary Care, Diabetes & Vascular Medicine, Diabetes Research Centre, University of Leicester, Leicester General Hospital, Gwendolen Road, Leicester, LE5 4PW, kk22@le.ac.uk

Manuscript word count: 3499

For peer review only

For peer review only - http://bmjopen.bmj.com/site/about/guidelines.xhtml

ABSTRACT

Objective

To synthesise findings from randomised controlled trials (RCTs) of interventions aimed at increasing medication adherence in individuals with type 2 diabetes (T2DM) and/or cardiovascular disease (CVD). And to compare the effect of explanatory versus pragmatic trial designs to explore whether outcomes are relatable to real world clinical practice.

Design

Systematic review and meta-analysis

Data sources

Ovid Medline, Ovid Embase, Web of Science and CINAHL from January 1, 2013 to December 31, 2018

Eligibility criteria for selecting studies

RCTs lasting ≥3 months (90 days), involving ≥200 patients in the analysis, with either established CVD and/or T2DM and which measured medication adherence. From 4403 citations, 103 proceeded to full text review. Studies published in any language other than English and conference abstracts were excluded.

Main outcome measure

Change in medication adherence

Results

Of 4403 records identified, 34 studies were considered eligible, of which 28, including 30,861 participants, contained comparable outcome data for inclusion in the meta-analysis. Overall interventions were associated with an increase in medication adherence (odds ratio 1.57 (95% CI: 1.33, 1.84), p<0.001, standardised mean difference 0.24 (95%CI: -0.10, 0.59) p=0.101). The effectiveness of interventions did not differ significant between studies considered pragmatic versus explanatory (p=0.598), but did differ by intervention type, with studies that included a multi-faceted rather than a single-faceted intervention having a more significant effect (p=0.010). The analysis used random effect models and used The Cochrane Risk of Bias Tool to assess study quality.

Conclusions

In this meta-analysis interventions were associated with a significant increase in medication adherence. Overall multi-faceted interventions which included an element of education alongside regular patient contact or follow up showed the most promise. Effectiveness of interventions between pragmatic and explanatory trials was comparable, suggesting that findings can be transferred from idealised to real word conditions.

Study Registration

PROSPERO (ID: CRD42017059460)

For peer review only - http://bmjopen.bmj.com/site/about/guidelines.xhtml

For peer review only

Strengths and limitations

- In a novel approach, this systematic review categorised relevant studies as being more pragmatic or more explanatory using the Pragmatic-Explanatory Continuum Indicator Summary-2 (PRECIS-2) tool and, in a meta-analysis, explored whether this affected the intervention effect.
- Heterogeneity was explored using subgroup analyses.
- A few studies had to be excluded from this systematic review and meta-analysis as they did not categorise diabetes type.

For peer review only

For peer review only - http://bmjopen.bmj.com/site/about/guidelines.xhtml

Introduction

Prevalence of type 2 diabetes (T2DM) and cardiovascular diseases (CVDs) are increasing rapidly.[1] They have been identified as two of the most common cardiometabolic morbidities associated with multi-morbidity,[1] and are two of the leading causes of death worldwide.[2] Most people diagnosed with these conditions will likely have multi-morbidity[1] (coexistence of two or more chronic conditions), particularly those aged 65-84 years where prevalence is estimated at 65%.[3]

Management of multi-morbidity is complex, typically relying on the co-prescription of multiple drugs, which is strongly associated with medication non-adherence.[4] The World Health Organisation estimates that 50% of individuals receiving chronic treatment are non-adherent (taking less than 80%) to prescribed medications.[5] Medication non-adherence is considered the biggest cause of suboptimal clinical outcomes, accounting for approximately 57% of avoidable costs in relation to medication use.[5]

Despite the rise in cardiometabolic multi-morbidity,[6-8] interventions to increase medication adherence within this population are sparse. As such we decided to focus this review on T2DM and CVD as the most prevalent cluster cardiometabolic morbidities. Treatment non-adherence within these populations is well recognised, and has become the focus of considerable research. As randomised controlled trials (RCTs) are generally expensive to conduct, it is important that their findings show real-world intervention effectiveness that is relevant to routine clinical practice. This study therefore aimed to not only identify interventions to increase medication adherence, but also compare the effectiveness of interventions categorised as explanatory (undertaken in idealised settings) or pragmatic (undertaken in real world settings). Whilst a handful of reviews in different research areas have now been published which retrospectively applied PRECIS-2 (The Pragmatic-Explanatory Continuum Indicator Summary-2) to see if comparing pragmatic with explanatory trials altered review findings,[9, 10] to the authors knowledge, this is the first review to incorporate PRECIS-2 scoring into the initial review process. Using PRECIS-2 this review scored interventions from very explanatory to very pragmatic to explore whether the differences in characteristics between the study designs of these trials influenced intervention effectiveness estimates in a meta-analysis.

Methods

This systematic review has been registered on PROSPERO (ID: CRD42017059460) and was conducted in accordance with PRISMA guidelines.[11]

Data sources and searches

An extensive scoping search identified the great breath of research published within this topic area. It also identified a 2014 Cochrane Review on medication adherence which included studies published up to January 11, 2013. Due to factors relating to the focus of the review, changes in prescribed medication and the increasing use of mobile technology interventions in recent adherence research, we decided that this review would provide a contemporary update and therefore we refined our literature search to include studies published between January 1, 2013 to December 31, 2018. We searched Ovid Medline 1946 (Epub ahead of print, in process and other non-indexed citations), Ovid Embase 1974, Web of Science 1970 and Cumulative Index of Nursing and Allied Health Literature (EBSCO CINAHL-plus with full text). We searched “type 2 diabetes”, “cardiovascular disease”, “medication adherence” and “randomised control trial” using a combination of medical subject headings, keywords and synonyms with both English and American spellings. An example search strategy can be found in (Supplementary eTable 1).

For peer review only - http://bmjopen.bmj.com/site/about/guidelines.xhtml

Screening

Two investigators (CF and EI) independently screened all titles, abstracts and full text articles. Discrepancies were resolved through discussion with a third researcher (CG). Studies were assessed against five eligibility criteria to determine firstly, whether the study was an RCT; second, that patients were identified as having established CVD and/or T2DM; third, the trial measured medication adherence; fourth, study duration was ≥3 months (90 days) and fifth; the study included >200 people in the analysis. Studies published in any language other than English and conference abstracts were excluded. Additional reasons for exclusion were where articles failed to specify diabetes type or where results were combined for individuals at risk of and with established CVD.

Data extraction

Data extraction was performed by CF and checked by CG. Where available the following data were recorded: study characteristics (authors, year, country, duration, sample size); participant characteristics (age, gender, disease); methods of assessment (self-report, pharmacy records, pill count); intervention type (Supplementary eTable 2); and outcome measures of medication adherence. For trials reporting multiple follow-up, the final follow-up corresponding to study duration was used.

Risk of Bias

Risk of bias assessment was undertaken using The Cochrane Risk of Bias Tool for Randomized Controlled Trials.[12]

PRECIS-2

Using Loudon *et al.* (2015) for guidance,[13] PRECIS-2 scoring to explore how pragmatic/explanatory different components of the included study designs was undertaken independently by CF and CG. Each of the nine domains was given a score from one (very explanatory "ideal study conditions") to five (very pragmatic "usual care conditions"). Results were compared and a consensus score was reached.

Some studies did not clearly explain all components making scoring difficult. In order to determine whether the treatment of unclear data affected the overall score we conducted two classification processes. For the primary method we inputted a score of 3 and then calculated an average score by adding up the scores and dividing by 9. In the second a score was not given for the missing domains and then an average was calculated based on available domain scores.

Data synthesis

Study data were reported as means and medians for continuous data and as proportions for categorical data. Twenty two studies defined a cut point to determine adherence. Twenty used a binary outcome defining individuals as adherent or non-adherent, however two categorised individuals based on a pre-specified level of low medium and high. Of those reporting levels of low, medium or high adherence, as has been done in previous studies,[14, 15] we combined medium and high levels and separated low level to form a binary outcome of adherence or non-adherence respectively, therefore enabling odds ratio of adherence to be calculated.

For peer review only - http://bmjopen.bmj.com/site/about/guidelines.xhtml

A further six studies, plus one which was included in the previous analysis, reported adherence using a continuous scale which provided an overall group indication of adherence. For these studies, change in adherence for each study arm was calculated. As scores differed, we calculated standardised mean differences (SMD) to combine study estimates in a meta-analysis. For both meta-analysis models, funnel plots and Egger's test were carried out to assess for publication bias and random effects meta-analyses were fitted to account for heterogeneity in study design. We assessed heterogeneity between studies by calculating the Higgins I^2 statistic, with an I^2 statistic >75% considered high heterogeneity.[16] In one study (Boyne, 2014), all participants in the intervention arm were adherent at follow-up, so a continuity correction of 0.5 was added to allow an odds ratio to be calculated.

We fitted meta-regression models assessing; study length, whether adherence was a primary or secondary outcome, mean age, percent male, disease (T2DM or CVD), and PRECIS-2 score, to explore the impact of study heterogeneity on the intervention effect. Three different sub-group analyses were also undertaken. The first compared intervention effects of studies that included a self-reported measure of adherence, to those with an objective adherence measure. The second compared pragmatic with explanatory studies (pragmatic studies were those with an average PRECIS-2 score >3 and explanatory a score of 3 or less) and the third compared outcomes of interventions which were identified as multi-faceted against those with a singular intervention component.

Where studies had obtained both pharmacy and self-report data, pharmacy data was used in preference in the meta-analysis. In the case of a few studies which did not report overall level of adherence and instead listed individual drug adherence, we made the pragmatic decision to monitor the adherence effect of the first drug listed by the author.

All analyses were performed using Stata 15 with the METAN, METAREG and METABIAS commands. Results are reported with 95% confidence intervals (CI) with a p value <0.05 considered statistically significant. For the few studies not included within the meta-analysis, descriptive synthesis of intervention effects is discussed.

Results

Study selection

Following deduplication we screened 4403 titles and abstracts which yielded 103 potentially relevant studies. Thirty three met the inclusion criteria with an additional study[17] identified from reference list and forward citation screening (Figure 1).[17] Of the 34 studies, 28 including 30,861 participants contained comparable outcome data for inclusion in the meta-analysis.

Characteristics of included studies

The key characteristics of included studies are shown in Supplementary eTable 3. Studies were conducted in 17 countries, with one study[18] conducted across four countries. Twenty three trials were conducted in high income countries,[17, 19-40] seven in upper middle,[41-47] two in lower middle,[48, 49] one in a low income country, and one across both upper middle and high income economies[50]. The proportion of males ranged from 27.9%[25] to 94.0%[17]. Four trials did not report age[18, 20, 46, 50] and/or gender.[18, 20, 50]

For peer review only - http://bmjopen.bmj.com/site/about/guidelines.xhtml

For peer review only

Table 1: Shaded graph to show PRECIS-2 scoring of included studies														
First Author	Year	Eligibility	Recruitment	Setting	Organisation	Flexibility: delivery	Flexibility: Adherence	Follow-up	Primary outcome	Primary analysis	Total score	Average score	Amended Total score	Amended Average score
Al Haj	2016	4	4	5	1	2	3	2	1	5	27	3.00	24	3.00
Barker collo	2015	5	5	5	1	1	3	1	5	5	31	3.44	28	3.50
Boyne	2014	5	5	5	2	1	3	1	2	5	29	3.22	26	3.25
Buhse	2018	1	4	5	1	2	3	1	1	5	23	2.56	20	2.50

Medical conditions varied across studies; 12 studies were in participants with T2DM,[19, 22-25, 28, 31, 35, 42, 47, 48, 50] 21 in participants with CVD[17, 18, 20, 21, 26, 27, 29, 30, 32-34, 36-38, 40, 41, 43-46, 49] and one in people with both.[39] Follow up ranged from 3-36 months.

Medication adherence was assessed as a primary outcome in 19 studies.[18, 19, 21-23, 27, 29, 30, 33, 36, 38, 39, 42, 44-47, 49, 50] Methods of measuring medication adherence varied between studies. Adherence was primarily assessed through self-report (n=23),[17-26, 28, 32, 34, 36, 38, 41-44, 47-50] followed by pharmacy data (n=10),[22, 29, 30, 35-37, 39, 40, 44, 47] pill count (n=2),[18, 27] and electronic pill bottle opening (n=1).[33] Three studies did not report the method of medication adherence assessment used.[31, 45, 46] Of those measuring adherence through self-report, the Morisky-Green questionnaire[51] and the 8-item Morisky Medication Adherence scale (MMAS-8)[52] (or translated versions) were the most frequently used. Four studies measured adherence using two different methods, self-report and either pharmacy or pill count data.[18, 22, 44, 47] Two of the studies combined the data to provide an overall level or adherence,[18, 22] whereas the other two reported adherence outcomes separately.[44, 47] Of the two studies reporting separate outcomes, both reported consistent findings and statistically significant improvements in adherence whether self-report or pharmacy data were used.

Interventions varied greatly between studies. We categorised and defined interventions (Supplementary eTable 2) based on their most prominent feature in to one of the following seven groups; (1) behavioural/educational [17, 20, 22-24, 27, 28, 32, 34, 35, 38, 45, 47, 48], (2) intensified patient care[36, 43] (3) collaborative care[41] (4) simplification of drug regimen[18] (5) personalised drug dispensing[50] (6) telemonitoring/telemedicine[21, 31, 39] (7) multi-faceted intervention strategy.[19, 25, 26, 29, 30, 33, 37, 40, 42, 44, 46, 49]

PRECIS-2 Scoring

Details of PRECIS-2 scoring for each of the included studies can be found in Table 1. For the purpose of this study it was considered that each of the nine domains on the PRECIS-2 wheel had equal weighting. For visual clarity, PRECIS-2 results are presented as a shaded graph (Table 1) where darker shades represent more pragmatic components. We also inputted scores on the PRECIS-2 wheel (Supplementary eFigure 1, 2 & 3).

For peer review only - http://bmjopen.bmj.com/site/about/guidelines.xhtml

Caetano	2018	4	5	5	4	5	5	3	1	5	37	4.11	34	4.25
Cao	2017	2	4	5	1	2	4	2	5	5	30	3.33	30	3.33
Carrasquillo	2017	1	1	4	1	2	1	1	2	4	17	1.89	17	1.89
Castellano	2014	4	5	5	5	5	2	2	1	5	34	3.78	34	3.78
Chung	2014	2	5	5	4	1	5	2	1	5	30	3.33	30	3.33
Crowley	2013	1	1	5	2	2	3	2	2	4	22	2.44	19	2.38
Du	2016	5	4	5	2	2	3	2	5	5	33	3.67	30	3.75
El Touky	2017	4	5	5	4	3	1	2	1	1	26	2.89	23	2.88
Graumlich	2016	1	2	5	1	1	4	2	2	5	23	2.56	23	2.56
Hedegaard	2014	4	4	5	2	2	3	4	1	5	30	3.33	27	3.38
Ho	2014	4	4	5	2	1	3	5	1	5	30	3.33	27	3.38
Jeong	2018	4	5	5	1	4	3	2	2	4	30	3.33	27	3.38
Jia	2017	3	4	5	2	4	3	4	1	4	30	3.33	27	3.38
Kronish	2014	4	1	4	1	2	3	1	2	5	23	2.56	20	2.50
Lin	2017	1	2	4	1	2	3	1	1	5	20	2.22	17	2.13
Marin	2015	1	2	5	4	4	3	2	1	4	26	2.89	23	2.88
Marquez	2018	2	1	5	2	2	1	3	1	2	19	2.11	19	2.11
Meng	2014	4	5	5	2	4	3	2	2	4	31	3.44	28	3.50
Meng	2016	2	5	4	2	4	3	2	4	4	30	3.33	27	3.38
Peng	2014	3	5	5	2	1	3	3	1	4	27	3.00	21	3.00
Pladeval	2015	2	2	5	1	5	3	4	2	5	29	3.22	26	3.25
Rinfret	2013	4	4	5	2	4	3	4	1	5	32	3.56	29	3.63
Samtia	2013	2	4	4	4	3	3	1	2	4	27	3.00	21	3.00
Schou	2014	1	5	5	4	4	4	4	1	4	32	3.56	32	3.56
Schwalm	2015	5	5	5	4	4	3	2	1	4	33	3.67	30	3.75
Su	2016	4	5	5	2	2	3	2	1	4	28	3.11	25	3.13
Vollmer	2014	4	5	5	4	4	3	5	1	5	36	4.00	33	4.13
Volpp	2017	2	1	5	1	4	2	5	5	5	30	3.33	30	3.33
Xavier	2016	4	5	5	1	2	2	1	1	5	26	2.89	26	2.89
Xin	2015	2	3	3	2	4	3	2	1	4	24	2.67	15	2.50

For peer review only - http://bmjopen.bmj.com/site/about/guidelines.xhtml

Of the 34 studies, 20 studies were identified as being more pragmatic, with an average score >3 (range 3.11-4.11)[17, 18, 20, 21, 23, 26, 29-31, 34-43, 46]. Of these, most (n=18) received an average score between 3-4, demonstrating a slightly more pragmatic intention on the PRECIS-2 continuum. Two of the studies scored ≥4,[23, 39] demonstrating a greater degree of pragmatism. The majority of these studies scored poorly (score of 1) in relation to pragmatism of the primary outcome as, based on the guidance by Loudon *et al.* (2015),[13] medication adherence was not considered of obvious importance from the patients perspective and was typically assessed by methods not used routinely in primary care. Eleven studies were identified as being more explanatory receiving an average score of <3 (range 1.89-2.89).[22, 24, 25, 27, 28, 32, 33, 44, 47, 49, 50] Of these, most (n=10) received an average score between 2-3 demonstrating that they favour a more explanatory intention on the continuum. Finally, three studies were given a score of 3[19, 45, 48] suggesting that these were equally pragmatic and explanatory.

The domains within which trials scored a more pragmatic rating were recruitment, setting of the intervention and the primary analysis which received average scores of 3.7, 4.8 and 4.4 respectively. All other domains appeared more explanatory with average scores of 2.2, 2.8, 2.9, 2.4 and 1.8 for the domains of organisation, flexible delivery, flexible adherence, follow-up and primary outcome respectively. The eligibility domain was the only one to receive a score of 3.

When results were compared based on the way missing data were scored, no changes to any studies overall categorisation was observed.

Meta-analysis

Effect of interventions

Data from 22 studies were pooled in a meta-analysis (Figure 2). Irrespective of intervention type or duration, interventions significantly improved medication adherence when compared to control (OR 1.57 (95% CI: 1.33, 1.84), p<0.001). For the seven studies where adherence was reported as a continuous variable, the pooled SMD was estimated as 0.24 (95% CI: -0.10, 0.59), p=0.101) (Supplementary eFigure 4), in favour of the intervention group, but was not statistically significant.

Both meta-analyses showed statistically significant heterogeneity between studies (p<0.001) with high I^2 values of 80.5% and 95.4% for the meta-analyses of the odds ratios and SMDs respectively, where the I^2 value represents the percentage of variability due to heterogeneity rather than sampling error.

We investigated the heterogeneity by conducting meta-regression analyses (Supplementary eTable 4). No statistically significant association was found between the intervention effects and study length, mean study age, percent male, disease (CVD or T2DM), whether it was a primary or secondary outcome or PRECIS-2 score. Sub-group analyses identified no differences in the intervention effect between self-reported and objective measures of adherence, and between PRECIS-2 score (Supplementary eTable 5). Sub-group analyses comparing multi versus singular-faceted interventions showed multi-faceted interventions led to a statistically significant improvement in odds of adherence (p=0.010) (Figure 3, Supplementary eTable 5). The funnel plot and Egger's test (p=0.184) showed that no indication of publication bias was present for the meta-analysis of SMDs (Supplementary eFigure 5). For the meta-analysis of odds ratios, Egger's test for publication bias was statistically significant (p=0.041), with the funnel plot asymmetry indicating a

For peer review only - http://bmjopen.bmj.com/site/about/guidelines.xhtml

slightly uneven presence of small studies showing a favourable intervention effect (Supplementary eFigure 6).

Descriptive synthesis

Six studies did not contain comparable data for inclusion in the meta-analysis.[31, 33, 36, 37, 40, 50] Of those, four studies showed a positive intervention effect on rates of medication adherence,[31, 33, 36, 50] whilst two showed no statistically significant intervention effects.[37, 40]

Assessment of risk of bias

Table 2 displays trial specific risk of bias assessment. Few studies were deemed to be of fair quality with low risk of bias and none were considered to be free of bias. Twenty seven trials provided information about adequate sequence generation and 17 regarding allocation concealment. Incomplete outcome data was a concern in four trials and selective reporting was primarily deemed unclear (n=19) due to the number of trials without a published protocol. Blinding of participants and personnel was the main domain scoring poorly for risk of bias however the nature of these types of trials means it is often impractical to do so.

Table 2: Cochrane risk of bias of included studies

	Adequate random sequence generation	Allocation concealment	Blinding of participants and personnel	Blinding of outcome assessors	Incomplete outcome data	Selective reporting	Other
Al Haj 2016	+	+	-	?	+	?	?
Barker Collo 2015	+	+	-	+	-	+	+
Boyne 2014	+	?	?	?	+	?	?
Buhse 2018	+	+	-	+	+	-	+
Caetano 2018	+	?	-	-	+	?	?
Cao 2017	+	+	-	+	+	?	?
Carrasquillo 2017	+	+	-	+	+	+	?
Castellano 2014	+	+	-	-	+	+	+
Chung 2014	?	?	-	-	+	?	?
Crowley 2013	+	+	-	-	+	+	?
Du 2016	+	+	-	?	+	?	?
El Touky 2017	?	?	?	?	-	?	?
Graumlich 2016	+	+	-	-	+	+	+
Hedegaard 2014	+	+	-	+	+	?	?
Ho 2014	+	+	?	+	+	+	+
Jeong 2018	?	?	-	-	+	-	?
Jia 2017	?	+	?	?	-	?	?
Kronish 2014	+	+	-	+	+	?	?

For peer review only - http://bmjopen.bmj.com/site/about/guidelines.xhtml

Lin 2017	+	?	?	+	+	?	?
Marin 2015	+	?	?	?	+	-	-
Marquez 2018	+	+	?	?	-	?	?
Meng 2014	-	-	-	-	+	?	?
Meng 2016	+	+	+	?	+	+	?
Peng 2014	+	?	?	?	?	+	?
Pladeval 2015	+	?	-	+	+	?	-
Rinfret 213	+	+	-	+	+	?	?
Samtia 2013	?	?	?	?	+	?	-
Schou 2014	+	+	-	-	+	+	-
Schwalm 2015	+	-	?	+	+	+	-
Su 2016	+	?	?	?	-	?	?
Vollmer 2014	+	?	-	-	+	?	?
Volpp 2017	+	?	-	+	+	-	?
Xavier 2016	+	?	-	-	+	+	?
Xin 2015	?	?	-	+	+	?	?
**Key: +** low risk of bias, **-** high risk of bias, **?** unclear risk of bias							

Discussion

Medication adherence is a key component of chronic disease care yet many patients fail to follow prescribing guidelines.[(5)] This review identified 34 trials that evaluated the impact of interventions aimed at improving medication adherence in individuals with either T2DM and/or established CVD. Overall interventions significantly increased medication adherence.

To our knowledge this is the first review to systematically compare and synthesise medication adherence outcomes based on the PRECIS-2 classification of trials. Our review showed that interventions improve medication adherence and the PRECIS- 2 classification did not appear to affect the outcome when compared in subgroup and meta-regression analyses. This finding differs from previous research where explanatory trials have reported significantly larger effect sizes than pragmatic trials.[(9, 10)] This suggests that findings from these interventions are representation of real world clinical practice.

Using the PRECIS-2 tool retrospectively posed a number of challenges. Missing data in published manuscripts is a known problem when conducting systematic reviews, and posed additional problems for this review as manuscripts often did not include the level of detail required to accurately score certain domains (Supplementary eTable 6). As has been identified in previous research,[(53)] the most challenging domains to score were those relating to the flexibility of delivery and flexibility of participant adherence. Most studies focussed on detail the content of the intervention not its delivery and adherence. In order to define a study as having a more pragmatic or explanatory tendencies, we averaged domain scores to >3 or ≤3 respectively. Whilst considered the best method, we were aware of problems which could arise as a result of combining data in this way. Combining scores could result in two or more studies receiving the same or similar scores even though individual domain scores were very different. It also meant that each domain was given equal weighting in the overall score of pragmatism. Whilst a known limitation to the authors, no published guidance on how best to report the data could be found.

We restricted our review to articles published since 2013 to provide a contemporary update following a large Cochrane review published 2014 by Nieuwlaat et al.[(54)] Similarly to the 2014 review,

For peer review only - http://bmjopen.bmj.com/site/about/guidelines.xhtml

our findings suggest that interventions were diverse in nature and the majority were complex, involving multiple different components. Of the number of "successful" interventions, multi-faceted interventions which included an element of education alongside regular patient contact showed the most promise, suggesting that frequent engagement with the healthcare team may trigger behaviour change, or act as a reminder to undertake the behaviour. Whilst promising, considerations need to be made as to the ability of such interventions to be up-scaled and implemented. Multi-faceted interventions can be expensive and therefore their cost utility needs to be explored prior to such interventions becoming embedded in clinical care pathways.

As the number of people with access to mobile technologies has increased in the past decade, particularly in the over 60 age group, interventions which rely on frequent patient contact are becoming increasingly plausible and contact via either calls or SMS provide both a pragmatic and cheap alternative to face to face health care professional contact.[55] A systematic review by Changizi *et al.* (2017) looking into the effectiveness of mobile health in the elderly showed that it can be used effectively, and is widely accepted as a source of health literacy, particularly SMS messages due to their low requirement for technological competency.[56]

Our review highlighted a number of limitations to medication adherence research conducted to date. Firstly, the lack of gold standard method of measuring adherence makes it difficult to pool and compare outcomes. The most frequently used method within this review was self-report, particularly the tools developed by Morisky and colleagues.[51, 52] These tools are quick, easy and cost effective to administer making them ideal for use within a clinical care setting. The tools are validated and have shown moderate comparability with other indirect methods of medication adherence however their use has been associated with over estimation of true treatment adherence as they carry the potential for recall bias.[57]

Even amongst the studies utilising the Morisky scales methods of scoring and presenting data varied. For example, in the case of the Morisky-Green scale, some used a Likert scoring system,[25] some classed answers of no to all questions as indicative of good adherence,[38] whereas others classed good adherence as an answer of yes to all.[47] For the MMAS-8, studies such as Cao *et al.* (2017)[41] reported mean patient scores, with higher scores representing greater adherence. In contrast, Al Haj *et al.* (2016)[19] report the proportion of people categorised as low medium or highly adherent based on a score of <6, 6-7.9 or >8 respectively. Discrepancies in reporting make it difficult to compare outcomes and therefore there is a need for standardisation. The best method to measure medication adherence across disease populations, and the best approach to reporting said results, therefore continues to be an area requiring further exploration.

We also acknowledge that our findings may be limited by our strict inclusion criteria. All papers reviewed were written in English and published since 2013 and therefore results may not represent non-English and older research. Finally the lack of quality of included studies, particularly in relation to participant blinding and reporting bias could compromise the integrity of review findings.

Conclusion

This systematic review showed that interventions worked effectively to improve medication adherence in populations with T2DM and/or CVD. Multi-faceted interventions had the most significant effect. There is however a need to compare more standardised interventions and assess these using more uniform methods of measuring medication adherence to enable studies to be more realistically compared.

For peer review only - http://bmjopen.bmj.com/site/about/guidelines.xhtml

Recently there has been a focus on designing trials that are pragmatic and therefore more representative of ‘real life’ and the findings from this review suggest that these interventions are equally effective. There is however a need for further guidance to be developed to assist researchers in characterising and scoring studies.

WHAT IS ALREADY KNOWN ABOUT THIS TOPIC

Prevalence of multi-morbidity is increasing rapidly and typically relies on the co-prescription of multiple therapies. Adherence to prescribed therapies is poor with the World Health Organisation estimating that only 50% of individuals take their medication as prescribed. Despite the importance of identifying interventions to increase medication adherence being widely acknowledged, however the most effective strategies, and their transferability to routine clinical practice, have yet to be identified.

WHAT THIS STUDY ADDS

This review showed that interventions, particularly those which contain multiple intervention components, increase medication adherence in individuals with type 2 diabetes and/or CVD. This review compared effectiveness estimates between studies identified as being primarily pragmatic versus those characterised as being primarily explanatory and showed that intervention effects are not limited to studies which are undertaken in idealised study settings are therefore are representative of real world clinical practice.

For peer review only

For peer review only - http://bmjopen.bmj.com/site/about/guidelines.xhtml

Acknowledgements

Funding: The authors acknowledge support from the National Institute for Health Research Collaboration for Leadership in Applied Health Research and Care – East Midlands (NIHR CLAHRC – EM) and the Leicester Biomedical Research Centre. The views expressed are those of the authors and not necessarily those of the NHS, the NIHR or the Department of Health.

Author contributions: CF developed the study protocol, carried out the scientific literature search, screened and extracted data, undertook PRECIS-2 scoring, quality assessed studies, interpreted the results, and developed the first draft of the manuscript. CG quality assessed studies, interpreted the results, undertook data checking, PRECIS-2 scoring, performed all statistical analysis and contributed to the development of the manuscript. SS assisted with the design of the review and provided intellectual content to the drafted manuscript. DK assisted with the design of the review, applied for funding and assisted with protocol development. EK screened articles for inclusion in the review. MJD provided intellectual content to the drafted manuscript. PP and PG assisted with the design of the review. KK had the concept of the study, applied for funding, assisted with protocol development and provided intellectual oversight on the development of the manuscript. KK accepts full responsibility for the conduct of the study, has access to the data, and controlled the decision to publish. KK as the corresponding author attests that all listed authors meet authorship criteria and that no others meeting the criteria have been omitted.

The Corresponding Author has the right to grant on behalf of all authors and does grant on behalf of all authors, a worldwide licence to the Publishers and its licensees in perpetuity, in all forms, formats and media (whether known now or created in the future), to i) publish, reproduce, distribute, display and store the Contribution, ii) translate the Contribution into other languages, create adaptations, reprints, include within collections and create summaries, extracts and/or, abstracts of the Contribution, iii) create any other derivative work(s) based on the Contribution, iv) to exploit all subsidiary rights in the Contribution, v) the inclusion of electronic links from the Contribution to third party material where-ever it may be located; and, vi) licence any third party to do any or all of the above.

Competing interests: All authors have completed the Unified Competing Interest form (available on request from the corresponding author). CF, CG, SS, DK, EI, PP, PG declare: no support from any organisation for the submitted work; no financial relationships with any organisations that might have an interest in the submitted work in the previous three years; no other relationships or activities that could appear to have influenced the submitted work. MJD reports grants from Novo Nordisk, grants from Sanofi-Aventis, grants from Lilly, grants from Boehringer Ingelheim , grants from Janssen, personal fees from Novo Nordisk, personal fees from Sanofi-Aventis, personal fees from Lilly, personal fees from Merck Sharp & Dohme, personal fees from Boehringer Ingelheim, personal fees from AstraZeneca, personal fees from Janssen, personal fees from Servier, personal fees from Mitsubishi Tanabe Pharma Corporation , personal fees from Takeda Pharmaceuticals International Inc., outside the submitted work. KK reports personal fees from Amgen , personal fees from Astrazeneca, personal fees from Bayer, personal fees from NAPP, personal fees from Lilly , personal fees from Merck Sharp & Dohme , personal fees from Novartis, personal fees from Novo Nordisk , personal fees from Roche, personal fees from Berlin-Chemie AG / Menarini Group, personal fees from Sanofi-Aventis , personal fees from Servier, personal fees from Boehringer Ingelheim, grants from Pfizer , grants from Boehringer Ingelheim, grants from AstraZeneca, grants from Novartis, grants from Novo Nordisk, grants from Sanofi-Aventis, grants from Lilly, grants from Merck Sharp & Dohme, grants from Servier, outside the submitted work.

For peer review only - http://bmjopen.bmj.com/site/about/guidelines.xhtml

Ethical approval: Ethical approval for this meta-analysis is not required.

Data sharing: The datasets used or analysed during the current study are available from the corresponding author on reasonable request.

Transparency: The lead authors affirm that the manuscript is an honest, accurate, and transparent account of the study being reported; that no important aspects of the study have been omitted. The manuscript follows the PRISMA guidelines for reporting systematic reviews and meta-analyses.

Patient and public involvement: Patient and public involvement was undertaken prior to the development of the review to obtain an understanding of patient reasons for non-adherence. A number of themes were identified and included side effects of drugs and poor explanations from health care professionals about prescribed drugs. Following the completion of the review, alongside the views and opinions expressed during the PPI, the study team aim to develop a toolbox and education programme to support medication adherence. A second focus group is planned comprising of health professionals, with the objective of gathering their perspectives on how to promote the “toolbox” for general utilisation in clinical practice and how best to disseminate and mainstream the “toolbox” of interventions.

For peer review only

For peer review only - http://bmjopen.bmj.com/site/about/guidelines.xhtml

Reference List

1. Guthrie B, Payne K, Alderson P, McMurdo ME, Mercer SW. Adapting clinical guidelines to take account of multimorbidity. BMJ. 2012;345(oct04):e6341-e.doi:10.1136/bmj.e6341
2. World Health Organisation. Global Health Estimates 2016: Deaths by Cause, Age, Sex, by Country, and by Region, 2000-2016. Geneva: 2018.
3. Barnett K, Mercer SW, Norbury M, et al. Epidemiology of multimorbidity and implications for health care, research, and medical education: a cross-sectional study. Lancet. 2012;380(9836):37-43.doi:10.1016/S0140-6736(12)60240-2
4. Marengoni A, Onder G. Guidelines, polypharmacy, and drug-drug interactions in patients with multimorbidity. BMJ (Online). 2015;350.doi:10.1136/bmj.h1059
5. Sabaté E. Adherence to long-term therapies: evidence for action: World Health Organization; 2003.
6. Glynn LG. Multimorbidity: another key issue for cardiovascular medicine. Lancet. 2009;374(9699):1421-2.doi:10.1016/S0140-6736(09)61863-8
7. Tinetti ME, Fried TR, Boyd CM. Designing health care for the most common chronic condition—multimorbidity. JAMA. 2012;307(23):2493-4.doi:10.1001/jama.2012.5265
8. Weiss CO, Boyd CM, Yu Q, Wolff JL, Leff B. Patterns of prevalent major chronic disease among older adults in the United States. JAMA. 2007;298(10):1158-62.doi:10.1001/jama.298.10.1160-b
9. Sajobi TT, Li G, Awosoga O, et al. A comparison of meta-analytic methods for synthesizing evidence from explanatory and pragmatic trials. Systematic reviews. 2018;7(1):19.doi:10.1186/s13643-017-0668-3
10. Yoong SL, Wolfenden L, Clinton-McHarg T, et al. Exploring the pragmatic and explanatory study design on outcomes of systematic reviews of public health interventions: a case study on obesity prevention trials. Journal of Public Health. 2014;36(1):170-6.doi:10.1093/pubmed/fdu006
11. Moher D, Liberati A, Tetzlaff J, Altman DG. Preferred reporting items for systematic reviews and meta-analyses: the PRISMA statement. Ann Intern Med. 2009;151(4):264-9.doi:10.7326/0003-4819-151-4-200908180-00135
12. Higgins JP, Altman DG, Gøtzsche PC, et al. The Cochrane Collaboration's tool for assessing risk of bias in randomised trials. BMJ. 2011;343:d5928.doi:10.1136/bmj.d5928
13. Loudon K, Treweek S, Sullivan F, et al. The PRECIS-2 tool: designing trials that are fit for purpose. BMJ. 2015;350:h2147.doi:10.1136/bmj.h2147
14. Khayyat SM, Khayyat SMS, Alhazmi RSH, Mohamed MM, Hadi MA. Predictors of medication adherence and blood pressure control among Saudi hypertensive patients attending primary care clinics: a cross-sectional study. PloS one. 2017;12(1):e0171255.doi:10.1371/journal.pone.0171255
15. Krousel-Wood M, Islam T, Muntner P, et al. Association of depression with antihypertensive medication adherence in older adults: cross-sectional and longitudinal findings from CoSMO. Ann Behav Med. 2010;40(3):248-57.doi:10.1007/s12160-010-9217-1
16. Higgins JP, Thompson SG, Deeks JJ, Altman DG. Measuring inconsistency in meta-analyses. BMJ. 2003;327(7414):557-60.doi:10.1136/bmj.327.7414.557
17. Meng K, Seekatz B, Haug G, et al. Evaluation of a standardized patient education program for inpatient cardiac rehabilitation: impact on illness knowledge and self-management behaviors up to 1 year. Health Educ Res. 2014;29(2):235-46.doi:10.1093/her/cyt107
18. Castellano JM, Sanz G, Penalvo JL, et al. A polypill strategy to improve adherence: Results from the FOCUS Project. J Am Coll Cardiol. 2014;64(20):2071-82.doi:10.1016/j.jacc.2014.08.021
19. Al-Haj Mohd MM, Phung H, Sun J, Morisky DE. Improving adherence to medication in adults with diabetes in the United Arab Emirates. BMC Public Health. 2016;16(1):857.doi:10.1186/s12889-016-3492-0

For peer review only - http://bmjopen.bmj.com/site/about/guidelines.xhtml

20. Barker-Collo S, Krishnamurthi R, Witt E, et al. Improving Adherence to Secondary Stroke Prevention Strategies Through Motivational Interviewing Randomized Controlled Trial. Stroke. 2015;46(12):3451-8.doi:10.1161/strokeaha.115.011003

21. Boyne JJ, Vrijhoef HJ, Spreeuwenberg M, et al. Effects of tailored telemonitoring on heart failure patients' knowledge, self-care, self-efficacy and adherence: a randomized controlled trial. Eur J Cardiovasc Nurs. 2014;13(3):243-52.doi:10.1177/1474515113487464

22. Buhse S, Kuniss N, Liethmann K, et al. An informed shared decision making programme for patients with type 2 diabetes in primary care: Cluster randomised controlled trial. Diabetologia. 2017;60 (1 Supplement 1):S347.doi:10.1136/bmjopen-2018-024004

23. Caetano I, Santiago LM, Marques M. Impact of written information on control and adherence in type 2 diabetes. Rev Assoc Med Bras 1992. 2018;64(2):140-7.doi:10.1590/1806-9282.64.02.140

24. Carrasquillo O, Lebron C, Alonzo Y, et al. Effect of a Community Health Worker Intervention Among Latinos With Poorly Controlled Type 2 Diabetes The Miami Healthy Heart Initiative Randomized Clinical Trial. JAMA Intern Med. 2017;177(7):948-54.doi:10.1001/jamainternmed.2017.0926

25. Crowley MJ, Powers BJ, Olsen MK, et al. The Cholesterol, Hypertension, And Glucose Education (CHANGE) study: results from a randomized controlled trial in African Americans with diabetes. Am Heart J. 2013;166(1):179-86.doi:10.1016/j.ahj.2013.04.004

26. Du L, Dong P, Jia J, et al. Impacts of intensive follow-up on the long-term prognosis of percutaneous coronary intervention in acute coronary syndrome patients - A single center prospective randomized controlled study in a Chinese population. Eur J Prev Cardiol. 2016;23(10):1077-85.doi:10.1177/2047487315607041

27. El-Toukhy H, Omar A, Abou Samra M. Effect of acute coronary syndrome patients' education on adherence to dual antiplatelet therapy Adherence to dual antiplatelet therapy. J Saudi Heart Assoc. 2017;29(4):252-8.doi:10.1016/j.jsha.2017.02.003

28. Graumlich JF, Wang H, Madison A, et al. Effects of a Patient-Provider, Collaborative, Medication-Planning Tool: A Randomized, Controlled Trial. J Diabetes Res. 2016;2016:2129838.doi:10.1155/2016/2129838

29. Hedegaard U, Kjeldsen LJ, Pottegard A, Hallas J. Multifaceted intervention including motivational interviewing to support medication adherence after stroke/transient ischemic attack: a randomized trial. Cerebrovas Dis Extra. 2014;4(3):221-34.doi:10.1159/000369380

30. Ho PM, Lambert-Kerzner A, Carey EP, et al. Multifaceted intervention to improve medication adherence and secondary prevention measures after acute coronary syndrome hospital discharge : A randomized clinical trial. JAMA Intern Med. 2014;174(2):186-93.doi:10.1001/jamainternmed.2013.12944

31. Jeong JY, Jeon JH, Bae KH, et al. Smart Care Based on Telemonitoring and Telemedicine for Type 2 Diabetes Care: Multi-center Randomized Controlled Trial. Telemed J E Health. 2018;24(8):604-13.doi:10.1089/tmj.2017.0203

32. Kronish IM, Goldfinger JZ, Negron R, et al. Effect of peer education on stroke prevention: The prevent recurrence of all inner-city strokes through education randomized controlled trial. Stroke. 2014;45(11):3330-6.doi:10.1161/STROKEAHA.114.006623

33. Marquez-Contreras E, Martell-Claros N, Marquez-Rivero S, et al. Strategies for improving dabigatran adherence for stroke prevention in patients with non-valvular atrial fibrillation: education and drug intake reminders (FACILITA study). Curr Med Res Opinion. 2018;34(7):1301-8.doi:10.1080/03007995.2018

34. Meng K, Musekamp G, Schuler M, et al. The impact of a self-management patient education program for patients with chronic heart failure undergoing inpatient cardiac rehabilitation. Patient Educ Couns. 2016;99(7):1190-7.doi:10.1016/j.pec.2016.02.010.

For peer review only - http://bmjopen.bmj.com/site/about/guidelines.xhtml

35. Pladevall M, Divine G, Wells KE, Resnicow K, Williams LK. A Randomized Controlled Trial to Provide Adherence Information and Motivational Interviewing to Improve Diabetes and Lipid Control. Diabetes Educ. 2015;41(1):136-46.doi:10.1177/0145721714561031
36. Rinfret S, Rodes-Cabau J, Bagur R, et al. Telephone contact to improve adherence to dual antiplatelet therapy after drug-eluting stent implantation. Heart. 2013;99(8):562-9.doi:10.1136/heartjnl-2012-303004
37. Schou M, Gislason G, Videbaek L, et al. Effect of extended follow-up in a specialized heart failure clinic on adherence to guideline recommended therapy: NorthStar Adherence Study. Eur J Heart Fail. 2014;16(11):1249-55.doi:10.1002/ejhf.176
38. Schwalm JD, Ivers NM, Natarajan MK, et al. Cluster randomized controlled trial of Delayed Educational Reminders for Long-term Medication Adherence in ST-Elevation Myocardial Infarction (DERLA-STEMI). Am Heart J. 2015;170(5):903-13.doi:10.1016/j.ahj.2015.08.014
39. Vollmer WM, Owen-Smith AA, Tom JO, et al. Improving Adherence to Cardiovascular Disease Medications With Information Technology. Am J Manag Care. 2014;20:sp502-10
40. Volpp KG, Troxel AB, Mehta SJ, et al. Effect of electronic reminders, financial incentives, and social support on outcomes after myocardial infarction the heartstrong randomized clinical trial. JAMA Intern Med. 2017;177(8):1093-101.doi:10.1001/jamainternmed.2017.2449
41. Cao XY, Tian L, Chen L, Jiang XL. Effects of a hospital-community partnership transitional program in patients with coronary heart disease in Chengdu, China: A randomized controlled trial. Jpn J Nursing Sci. 2017;14(4):320-31.doi:10.1111/jjns.12160
42. Chung WW, Chua SS, Lai PSM, Chan SP. Effects of a pharmaceutical care model on medication adherence and glycemic control of people with type 2 diabetes. Patient Prefer Adherence. 2014;8:1185-94.doi:10.2147/ppa.s66619
43. Jia JJ, Dong PS, Du LJ, et al. Impact of Physician-Coordinated Intensive Follow-Up on Long-Term Medical Costs in Patients with Unstable Angina Undergoing Percutaneous Coronary Intervention. Acta Cardiol Sin. 2017;33(2):173-81.doi:10.6515/acs20160521a
44. Lin CY, Yaseri M, Pakpour AH, et al. Can a Multifaceted Intervention Including Motivational Interviewing Improve Medication Adherence, Quality of Life, and Mortality Rates in Older Patients Undergoing Coronary Artery Bypass Surgery? A Multicenter, Randomized Controlled Trial with 18-Month Follow-Up. Drugs & Aging. 2017;34(2):143-56.doi:10.1007/s40266-016-0429-3
45. Peng B, Ni J, Anderson CS, et al. Implementation of a structured guideline-based program for the secondary prevention of ischemic stroke in China. Stroke. 2014;45(2):515-9.doi:10.1161/STROKEAHA.113.001424
46. Su Q, Li C, Long F, et al. Effects of a health promotion program on medication adherence to antiplatelet therapy among ischemic stroke patients in Hainan Province, China. Vascular. 2017;25(3):242-8.doi:10.1177/1708538116666159
47. Xin CW, Xia ZN, Jiang C, Lin MM, Li GH. Effect of pharmaceutical care on medication adherence of patients newly prescribed insulin therapy: a randomized controlled study. Patient Prefer Adherence. 2015;9:797-802.doi:10.2147/ppa.s84411
48. Samtia AM, Rasool MF, Ranjha NM, Usman F, Javed I. A multifactorial intervention to enhance adherence to medications and disease-related knowledge in type 2 diabetic patients in Southern Punjab, Pakistan. Trop J Pharm Res. 2013;12(5):851-6.doi:10.4314/tjpr.v12i5.28
49. Xavier D, Gupta R, Kamath D, et al. Community health worker-based intervention for adherence to drugs and lifestyle change after acute coronary syndrome: a multicentre, open, randomised controlled trial. Lancet Diabetes Endocrinol. 2016;4(3):244-53.doi:10.1016/S2213-8587(15)00480-5
50. Marin GH, Risso P, Sbatella D, Haag G. Treatment adherence by personalizing the drug dispensing for diabetic patients in social vulnerable situation. Quality in Primary Care. 2015;23(2):93-6
51. Morisky DE, Green LW, Levine DM. Concurrent and predictive validity of a self-reported measure of medication adherence. Med Care. 1986:67-74.doi:10.1097/00005650-198601000-00007

For peer review only - http://bmjopen.bmj.com/site/about/guidelines.xhtml

52. Morisky DE, Ang A, Krousel-Wood M, Ward HJ. Predictive validity of a medication adherence measure in an outpatient setting. J Clin Hypertens. 2008;10(5):348-54.doi:10.1111/j.1751-7176.2008.07572
53. Johnson KE, Neta G, Dember LM, et al. Use of PRECIS ratings in the National Institutes of Health (NIH) health care systems research Collaboratory. Trials. 2016;17(1):32.doi:10.1186/s13063-016-1158-y
54. Nieuwlaat R, Wilczynski N, Navarro T, et al. Interventions for enhancing medication adherence. Cochrane Database of Syst Rev. 2014(11).doi:10.1002/14651858.CD000011.pub4
55. Zhou J, Rau P-LP, Salvendy G. Age-related difference in the use of mobile phones. Universal Access in the Information Society. 2014;13(4):401-13.doi:10.1007/s10209-013-0324-1
56. Changizi M, Kaveh MH. Effectiveness of the mHealth technology in improvement of healthy behaviors in an elderly population—a systematic review. Mhealth. 2017;3.doi:10.21037/mhealth.2017.08.06
57. Stirratt MJ, Dunbar-Jacob J, Crane HM, et al. Self-report measures of medication adherence behavior: recommendations on optimal use. Transl Behav Med. 2015;5(4):470-82.doi:10.1007/s13142-015-0315-2

For peer review only

For peer review only - http://bmjopen.bmj.com/site/about/guidelines.xhtml

Figure Legends

Figure 1 – PRISMA flow chart of included studies

Figure 2 – Forest plot of pooled odds ratios for medication adherence, stratified by PRECIS score

Figure 3 – Forest plot of odds ratios for adherence, stratified by complexity of the intervention

Supplementary eFigure 1– PRECIS-2 graphs of trials identified as pragmatic

Supplementary eFigure 2 – PRECIS-2 graphs of trials identified as explanatory

Supplementary eFigure 3. Average PRECIS-2 wheel domain scores for studies identified as pragmatic or explanatory

Supplementary eFigure 4 - Forest plot of pooled standardised mean differences for medication adherence, stratified by PRECIS score

Supplementary eFigure 5 - Funnel plot for the meta-analysis of standardised mean differences

Supplementary eFigure 6. Funnel plot for the meta-analyses of odds ratios

For peer review only

For peer review only - http://bmjopen.bmj.com/site/about/guidelines.xhtml

Figure 1- PRISMA flowchart of included studies

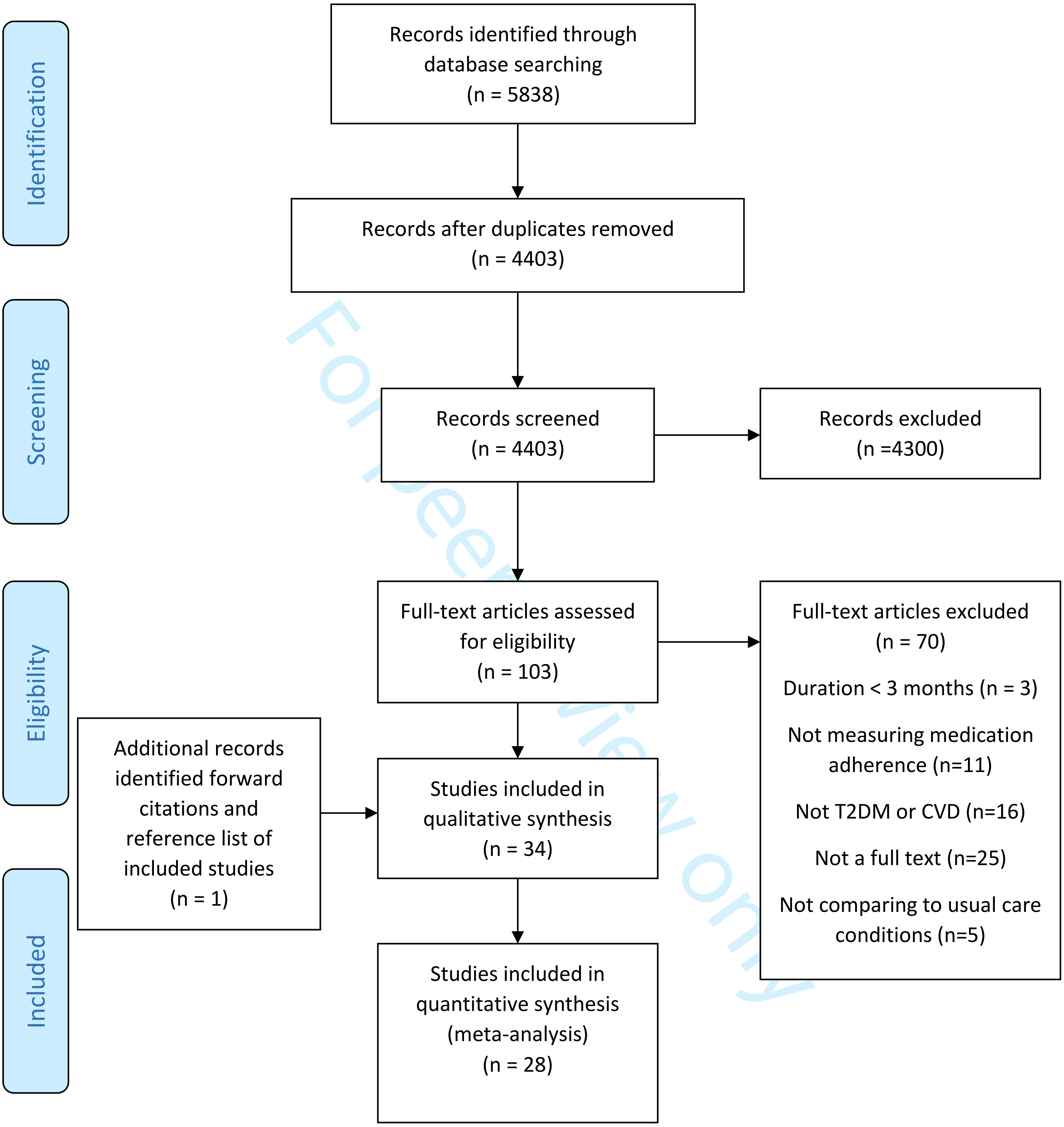

For peer review only

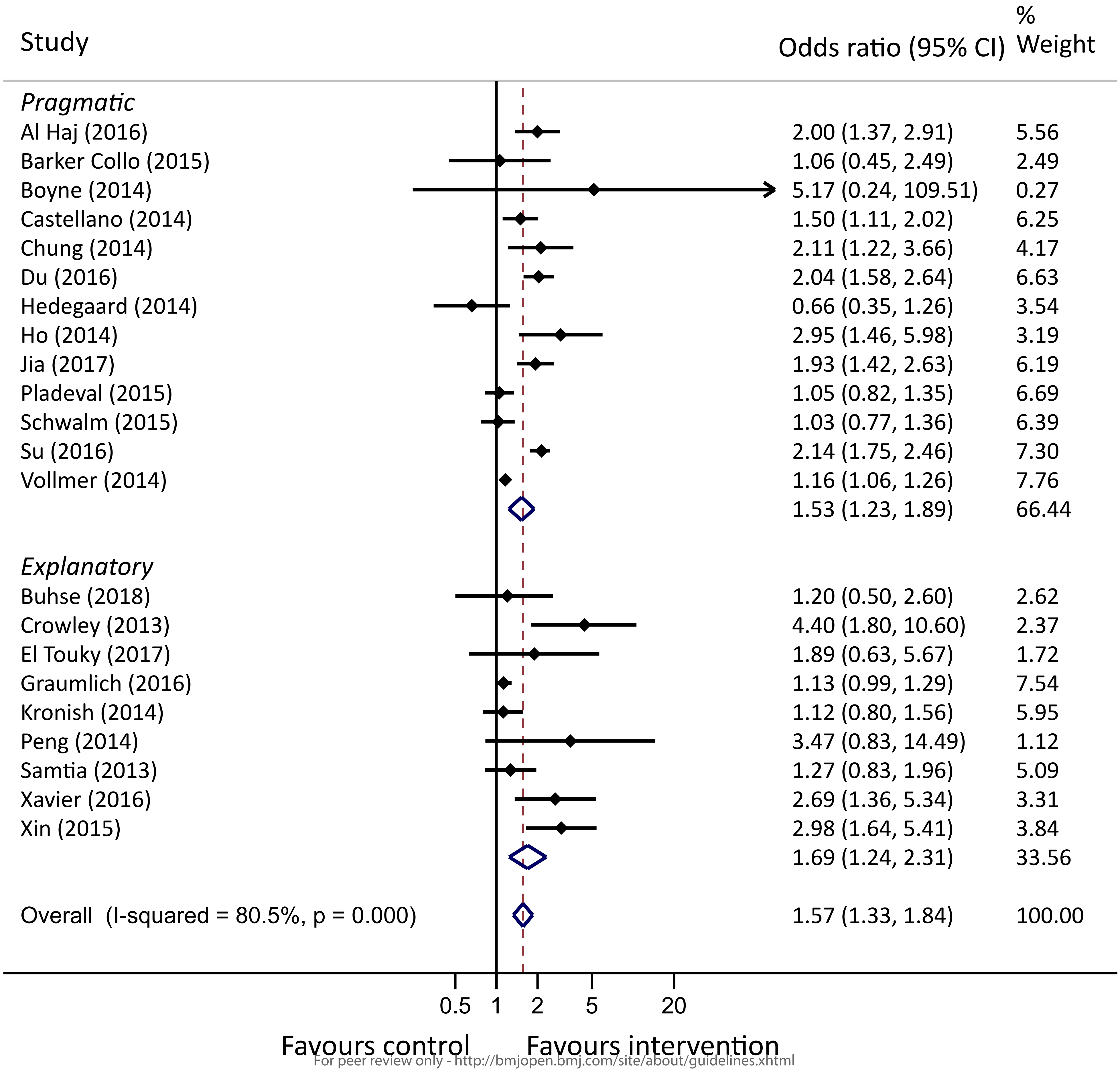

For peer review only - http://bmjopen.bmj.com/site/about/guidelines.xhtml

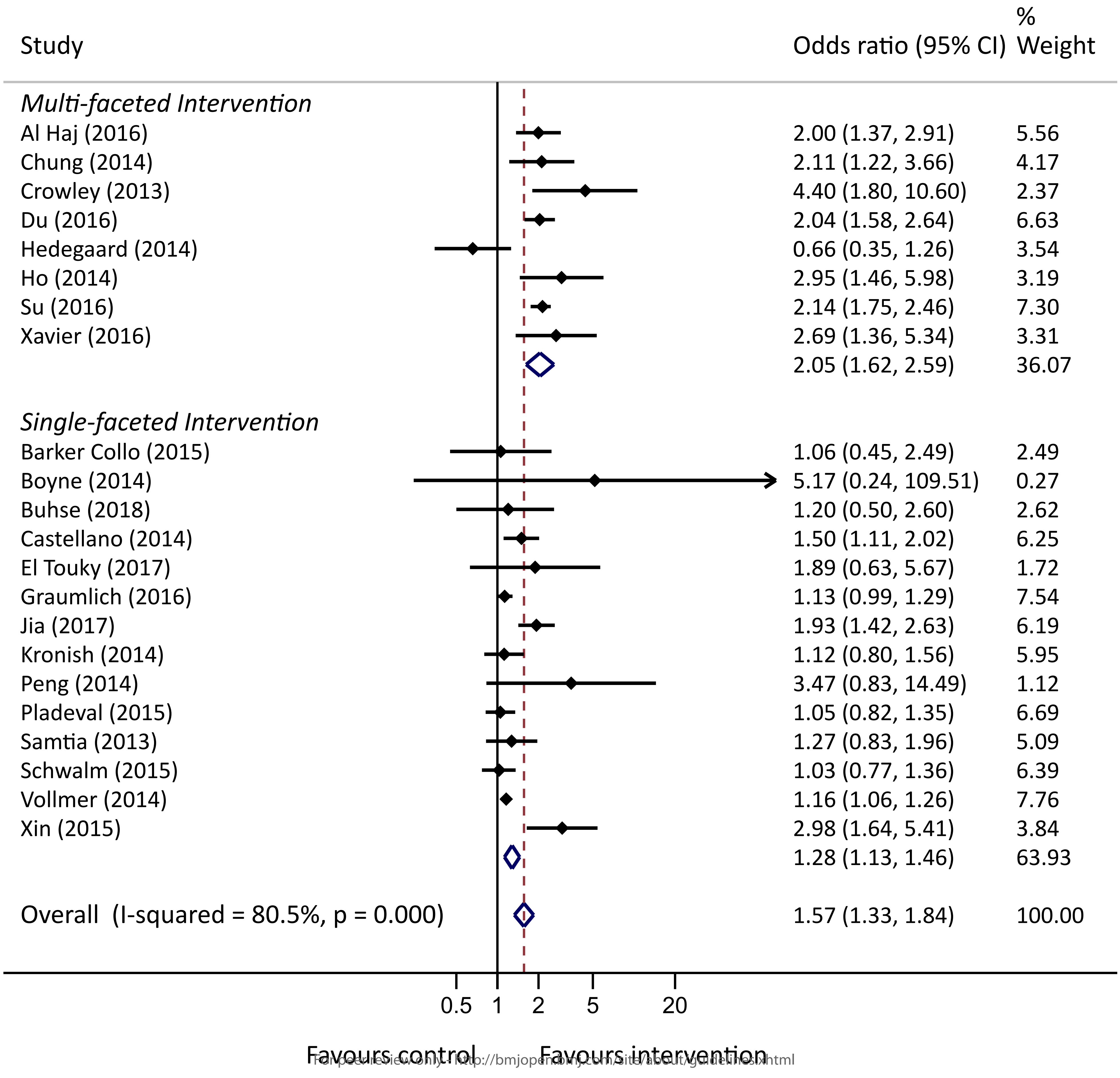

For peer review only - http://bmjopen.bmj.com/site/about/guidelines.xhtml

Supplementary eTable 1 – Medline Search Strategy

1	exp patient compliance/ or patient* adher*.mp.
2	exp medication compliance/ or medication* adher*.mp.
3	((patient* or medication* or medicine* or drug* or therap* or treatment*) adj2 (adher* or concordance or non adher* or nonadher* or compliance or non compliance or noncompliance or refusal)).mp. [mp=title, abstract, original title, name of substance word, subject heading word, floating sub-heading word, keyword heading word, protocol supplementary concept word, rare disease supplementary concept word, unique identifier, synonyms]
4	treatment refusal/ or treatment* refusal.mp.
5	exp *non insulin dependent diabetes mellitus/ or Type 2 diabet*.mp.
6	T2D*.mp.
7	((non insulin or noninsulin) adj dependent diabet*).mp. [mp=title, abstract, original title, name of substance word, subject heading word, floating sub-heading word, keyword heading word, protocol supplementary concept word, rare disease supplementary concept word, unique identifier, synonyms]
8	NIDD*.mp.
9	Diabetes Mellitus/ or diabet*.mp.
10	*cardiovascular disease/ or cardiovascular diseas*.mp. or CVD.mp. [mp=title, abstract, original title, name of substance word, subject heading word, floating sub-heading word, keyword heading word, protocol supplementary concept word, rare disease supplementary concept word, unique identifier, synonyms]
11	((heart or cardiac or cardiovascular or coronary) adj2 (disease* or disorder* or failure*)).mp. [mp=title, abstract, original title, name of substance word, subject heading word, floating sub-heading word, keyword heading word, protocol supplementary concept word, rare disease supplementary concept word, unique identifier, synonyms]
12	*heart arrhythmia/ or arrhythmia*.mp.
13	*atrial fibrillation/ or atrial fibrillat*.mp.
14	*heart infarction/ or myocardial infarct*.mp.
15	heart failure.mp. or *heart failure/
16	*ischemic heart disease/ or *Myocardial Ischemia/ or myocard* isch*.mp.
17	angina.mp. or *angina pectoris/

18	*cardiomyopathy/ or cardiomyopath*.mp.
19	*coronary artery disease/ or coronary artery diseas*.mp.
20	peripheral artery diseas*.mp. or *peripheral occlusive artery disease/ or *peripheral vascular disease/
21	stroke.mp. or *cerebrovascular accident/
22	*transient ischemic attack/ or transient isch* attack.mp.
23	TIA.mp.
24	controlled clinical trial.mp. or controlled clinical trial/
25	"randomized controlled trial (topic)"/ or RCT.mp.
26	clinical trial/
27	(randomi?ed controlled adj2 trial).mp. [mp=title, abstract, original title, name of substance word, subject heading word, floating sub-heading word, keyword heading word, protocol supplementary concept word, rare disease supplementary concept word, unique identifier, synonyms]
28	1 or 2 or 3 or 4
29	5 or 6 or 7 or 8 or 9 or 10 or 11 or 12 or 13 or 14 or 15 or 16 or 17 or 18 or 19 or 20 or 21 or 22 or 23
30	24 or 25 or 26 or 27
31	28 and 29 and 30
32	limit 31 to (english language and humans and yr="2013 - 2018")

only

Supplementary eTable 2 – Definitions of Intervention components	
Behavioural/educational	Educational interventions comprising of motivational interviewing, counselling, group or individual education sessions on disease, medication and general self-management and exercise classes. In general, these studies comprised of some form of structured education and or a motivational interviewing component.
Telemonitoring	Out-patient appointments were changed for telemonitoring visits, participants were contacted at regular intervals using web-based feedback, or interactive voice recognition when patients were due or overdue their medications
Collaborative care	Enhanced communication between hospital and homecare teams
Simplification of drug regimen	Simplification of drug regimen by using a polypill
Intensified patient care	Patients were contacted more regularly than usual or drug availability and compliance was more closely monitored
Personalised drug dispensing	Weekly contact via telephone or home visits with the participant to check drug availability and adherence
Multi-faceted	Varying combinations of the aforementioned interventions with electronic pill caps and pill boxes as additional interventions

For peer review only

Supplementary eTable 3 – **Characteristics of included studies**

First Author, year	Country	Sample size (analysed)<br>Duration<br>Data collection points	Condition	Key intervention detail	Medication adherence measure (adherence definition or method of reporting)
Al Haj Mohd, 2016[(19)]	United Arab Emirates	446 (446)<br>6 months<br>0 & 6 month	T2DM	Multifaceted intervention strategy<br>• Education - 30 minute session<br>• Intensified patient care - weekly phone calls for 3 months)	Self-report - MMAS-8<br>Higher scores = better adherence
Barker Collo 2015[(20)]	Australia	386 (326)<br>12 months<br>0, 3, 6, 9 & 12 months	Stroke/TIA	Behavioural/Educational<br>• 4 x motivational interviewing sessions which occurred either face to face or via telephone, initial interview 60-90mins, remainder 30 minutes	Self-report – asked whether in the last 7 days they had taken all their medication as prescribed, indicate number of doses/pills missed, reason for missed doses and any side effects if noticed.<br>Cross checked self-report with pharmacy data for validity.
Boyne, 2014[(21)]	Netherlands	382 (382)<br>12 months<br>0, 3, 6 12 months	Heart failure	Tele-monitoring/telemedicine<br>• Replace 2 of the 4 yearly outpatient appointments with tele-monitoring	Self-report – European heart failure self-care behavior scale
Buhse, 2018[(22)]	Germany	279 (279)<br>6 months<br>0, 3, 6, 9 & 12 months	T2DM	Behavioural/Educational<br>• Decision aid about primary prevention of MI<br>• 90 minute group education session about complication prevention<br>• Patient document with defined treatment goals	Pharmacy data<br>Self-report via telephone interview
Caetano, 2018[(23)]	Portugal	709 (702)<br>6 months<br>0 & 6 months	T2DM	Behavioural/Educational<br>• Patient given one of 3 educational leaflets on diabetes, diabetes treatment or physical activity	MAT scale<br>Higher scores=better adherence
Cao, 2017[(41)]	China	236 (236)<br>90 days	Coronary heart disease	Collaborative care<br>• Enhanced communication between hospital and home care team	Self-report - MMAS-8<br>Higher scores = better adherence

For peer review only

		0, 30 & 90 days			
Carrasquillo, 2017[24]	United States	300 (215)<br><br>12 months<br><br>0 & 12 months	T2DM	Behavioural/Educational<br>• Education - (mentoring by a community health worker approx. 4 home visits and 12 calls)<br>• Education – monthly group education sessions<br>• Behavioral – Bimonthly group exercise classes	Self-report - MMAS-8<br>Higher scores = better adherence
Castellano, 2014[18]	Argentina, Italy, Spain & Paraguay	695 (594)<br><br>9 months<br><br>0, 1, 4, 9 months	Myocardial infarction	Simplification of drug regimen<br>• Fixed dose combination polypill containing Aspirin, Simvastatin, Ramipril	Pill count<br>Self-report – Morisky-Green-Levine Medication Adherence Scale
Chung, 2014[42]	Malaysia	241 (241)<br><br>12 months<br><br>0, 4, 8 12 months	T2DM	Multifaceted intervention strategy<br>• Education – on diabetes, hypertension, hyperlipidemia and medications<br>• Medication aids – pill box and blood glucose monitoring equipment<br>• Intensified patient care – medication reviews and monthly follow up calls	Self-report - MMAS-8 (Revised Malaysian version)<br>Higher scores = better adherence
Crowley, 2013[25]	United States	359 (329)<br><br>12 months<br><br>0, 3, 6, 9 & 12 months	T2DM	Multifaceted intervention strategy<br>• Education – monthly self-management education telephone calls<br>• Personalised medication management – nurse and primary care provider contact at 3 monthly intervals to discuss medication adherence and possible medication changes	Self-report Morisky-Green-Levine Medication Adherence Scale
Du, 2016[26]	China	979 (964)<br><br>36 months<br><br>0 & 36 months	Percutaneous coronary intervention	Multifaceted intervention strategy<br>• Intensified patient care/education/personalized medication management - regular telephone follow ups at 1, 2, 3, 6, 12 & 36 months to discuss medication usage, provide health education and disease prevention strategies and schedule lab tests and treatment adjustments where necessary	Self-report Morisky-Green-Levine Medication Adherence Scale<br>Lower score = better adherence<br>(Score 0-2 = adherent)
El Touky, 2017[27]	Saudi Arabia	321 (276)		Behavioural/Educational intervention	Pill count >80% classed as adherent

For peer review only

		12 months<br><br>0, 1, 3, 6, 9, & 12 months	Acute coronary syndrome	• Educated patients on several aspects of coronary artery disease		
Graumlich, 2016[28]	United States	674 (674)<br><br>12 months<br><br>0, immediately following intervention, 3 & 6 months	T2DM	Medication monitoring table<br>• Multi-media tool embedded within the electronic medical record that aims to:<br>1. Promote patient knowledge<br>2. Support collaborative medication planning		Self-report -PMAQ -(patient medication adherence questionnaire)<br>Four questions were asked regarding whether the patient had missed taking a dose yesterday, the day before yesterday, 3 days ago, and over the past weekend. Patients were rated as having proper adherence if they self-reported no missed doses in this time period
Hedegaard, 2014[29]	Denmark	211 (203)<br><br>12 months<br><br>0, 3, 6, 9 & 12 months	Stroke/ TIA	Multifaceted intervention strategy<br>• Personalized medication management-medication review by pharmacist and advice on relevant drug related problems<br>• Behavioral – 3 x 30 minute patient motivational interview to support adherence and lifestyle change		Pharmacy data - MPR >0.8
Ho, 2013[30]	United States	253 (241)<br><br>12 months<br><br>0 & 12 months	Acute coronary syndrome	Multifaceted intervention strategy<br>• Personalized medication management - Medication reconciliation and tailoring by pharmacist and provision of pill box<br>• Education - Medication education<br>• Collaborative care – Pharmacist contacted patients<br>• Telemedicine – Medication reminder and refill calls		Pharmacy refill data >0.8
Jeong, 2018[31]	South Korea	338 (338)<br><br>24 weeks	T2DM	Tele-monitoring/telemedicine (Home telemonitoring composed of web based	Tele-monitoring/telemedicine Reminders (Home telemonitoring	Does not report how medication adherence was assessed.

For peer review only

		0 & 24 weeks		feedback and education with conventional management)	with remote management)	
Jia, 2017[43]	China	669 (669)<br><br>36 months<br><br>0, 1, 3, 6, 12 & 36 months	Percutaneous coronary intervention	Intensified patient care<br>• Regular phone calls with face to face appointments scheduled as necessary		Self-report Morisky-Green-Levine Medication Adherence Scale<br>Lower score = better adherence (Score 0-2 = adherent)
Kronish, 2014[32]	United States	600 (600)<br><br>6 months<br><br>0 & 6 months	Stroke/ TIA	Behavioural/Educational intervention<br>• 6 x 90 minute weekly peer led workshops about self-management skills and medication adherence		Self-report -MMAS-8<br>Higher scores = better adherence
Lin, 2017[44]	Iran	288 (288)<br><br>18 months<br><br>0, 6, 12 & 18 months	Coronary artery bypass grafting	Multifaceted intervention strategy<br>• Educational - 3 x 60 minute weekly education sessions<br>• Behavioral – 5 x 50 minute weekly motivational interviewing<br>• Telemedicine – 4 SMS reminders sent each month, content varied on a monthly basis		Self-report – MARS (5 item)<br>Score range 5-25- Higher scores = better adherence<br>Pharmacy data
Marin, 2015[50]	Argentina	467 (459)<br><br>12 months<br><br>0 & 12 months	T2DM	Personalised medication management<br>• Weekly contact with patient to check drug availability and compliance either by phone or home visit		Self-report Morisky-Green-Levine Medication Adherence Scale<br>Higher scores = better adherence
Marquez, 2018[33]	Spain	726 (625)<br><br>18 months<br><br>0, 6 & 12 months	Atrial Fibrillation	Multifaceted intervention strategy<br>• Education – healthcare and patient discussion of an educational booklet about medication and adherence<br>• Medication aid – medication reminder calendar		MEMs
Meng, 2014[17]	Germany	471 (425)<br><br>12 months	Coronary heart disease	Behavioural/Educational intervention<br>• 5 x 45minute patient centered self-management group education program		Self-report - MARS (German version)

For peer review only

		Admission, discharge, 6 & 12 months			Higher scores = better adherence
Meng, 2016[34]	Germany	513 (449)<br><br>12 months<br><br>Admission, discharge, 6 & 12 months	Heart failure	Behavioural/Educational intervention<br>• 5 x 60minute patient centered self-management group education program	Self-report MARS (German version)<br>Higher scores = better adherence
Peng, 2014[45]	China	3821 (3330)<br><br>12 months<br><br>Discharge, 6, 9 & 12 months	Stroke/ TIA	Behavioural/Educational intervention<br>• Lifestyle modification - diet, exercise, smoking and risk factor control	Does not report
Pladevall *2015*[35]	United states	1692 (1512)<br><br>18 months<br><br>0, 6, 12 & 18 months	T2DM	Behavioural/Educational intervention<br>• Adherence information provided to physicians to relay to patients<br>• Above plus motivational interviewing	Pharmacy data -PDC
Rinfret, 2013[36]	Canada	300 (300)<br><br>12 months<br><br>0 & 12 months	Percutaneous coronary intervention	Intensified patient care<br>• Telephone follow-up within 7 days of DES implantation and at 1, 6, and 9 months	Pharmacy refill data
Samtia, 2013[48]	Pakistan	348 (348)<br><br>5 months<br><br>0 & 5 months	T2DM	Behavioural/Educational<br>• Continual education by pharmacists about effects of complications, medication adherence, diet, exercise, foot care, smoking cessation and HbA1c	Self-report – adherent yes/no
Schou, 2014[37]	Denmark	921 (920)<br><br>13-72 months	Heart failure	Multi-faceted intervention strategy<br>• Intensified patient care – regular drug monitoring and access to regular telephone consultations	Pharmacy data - PDC >0.8

For peer review only

		Every 1-3 months		• Education – occurred if adherence decreased during drug monitoring	
Schwalm, 2015[38]	Canada	852 (852)<br><br>12 months<br><br>0, 3 & 12 months	Myocardial infarction	Behavioural/Educational<br>• Personalised letters at 1, 5, 8 and 11 months after angiogram which stated the importance and role of each medication.<br>• Prompt to talk to physician and pharmacist about meds	• Self-report – yes/no<br>• Self-report Morisky-Green-Levine Medication Adherence Scale perfect score %
Su, 2016[46]	China	1275 (1187)<br><br>12 months<br><br>0 & 12 months	Stroke/ TIA	Multi-faceted intervention strategy<br>• Education 3 x 30 minute sessions and easy to understand manual<br>• Intensified patient care – physician contact at 1, 3 and 6 months to monitor progress	Does not report
Vollmer, 2014[39]	United States	21752 (21752)<br><br>12 months<br><br>0 & 12 months	T2DM and/or CVD	Telemonitoring/telemedicine<br>• Interactive voice recognition (IVR) calls when pts due or overdue medications<br>• Enhanced IVR as above plus letter, EMR feedback, mailed materials	Pharmacy data - PDC >0.8
Volpp, 2017[40]	United States	1509 (1503)<br><br>12 months<br><br>0 & 12 months	Myocardial infarction	Multifaceted intervention strategy<br>• Medication aids - Electronic pill bottles used in place of regular pill bottles (Glow caps)<br>• Financial incentives – Patients included in a daily lottery with the potential to win $5<br>• Medication support partner – information on patients adherence shared with a support partner whose role it was to offer support and encouragement<br>• Intensified patient care – engagement advisor and social workers to provide support on adherence issues	Pharmacy data - PDC
Xavier, 2016[49]	India	805 (750)<br><br>12 months	Acute coronary syndrome	Multifaceted intervention strategy<br>• Behavioral/educational - Mentoring by a community health worker<br>• Medication aids - medication reminder calendar and	Pharmacy data -Composite medical adherence score >80%

For peer review only

		0 & 12 months		patient dairy which contained details on drugs, how to take them and benefits.	
Xin, 2015[(47)]	China	240 (227)<br>12 months<br>0 & 12 months	T2DM	Behavioural/Educational<br>• Individualised education<br>• Group education/activities<br>• Telephone counselling	• Prescription refill claims – 80-115<br>• Morisky-Green – perfect score %
Abbreviations- T2DM: type 2 diabetes; TIA: Transient Ischemic Attack; MMAS-8; Morisky Medication Adherence Scale (8-item); PDC: proportion of days covered; MARS-D: Medication Adherence Report Scale (German version); MPR: Medication Possession Ratio; MEMs: medication event monitoring system; 0 months =baseline					

For peer review only

Supplementary eFigure 1– PRECIS-2 graphs of trials identified as pragmatic

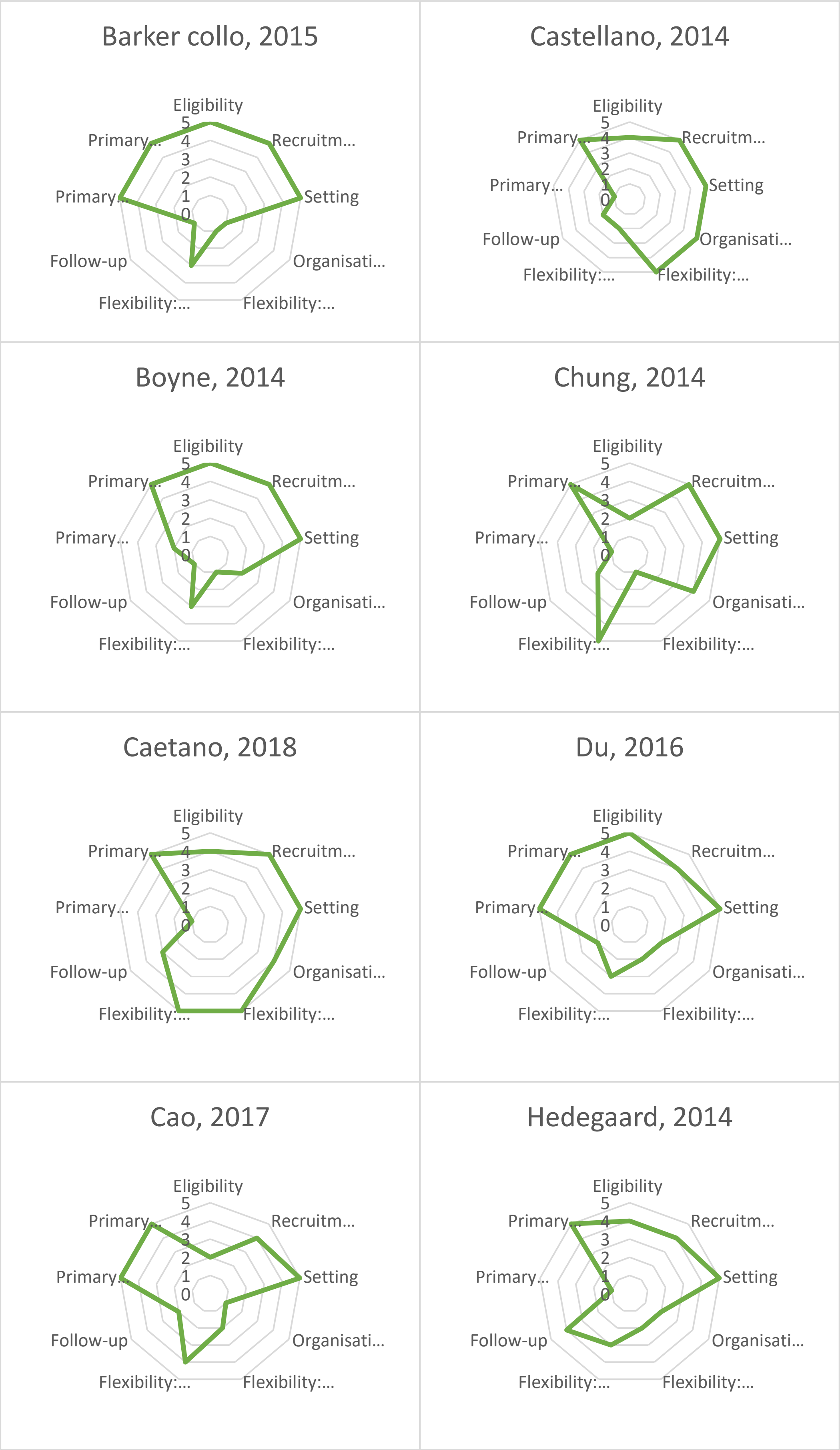

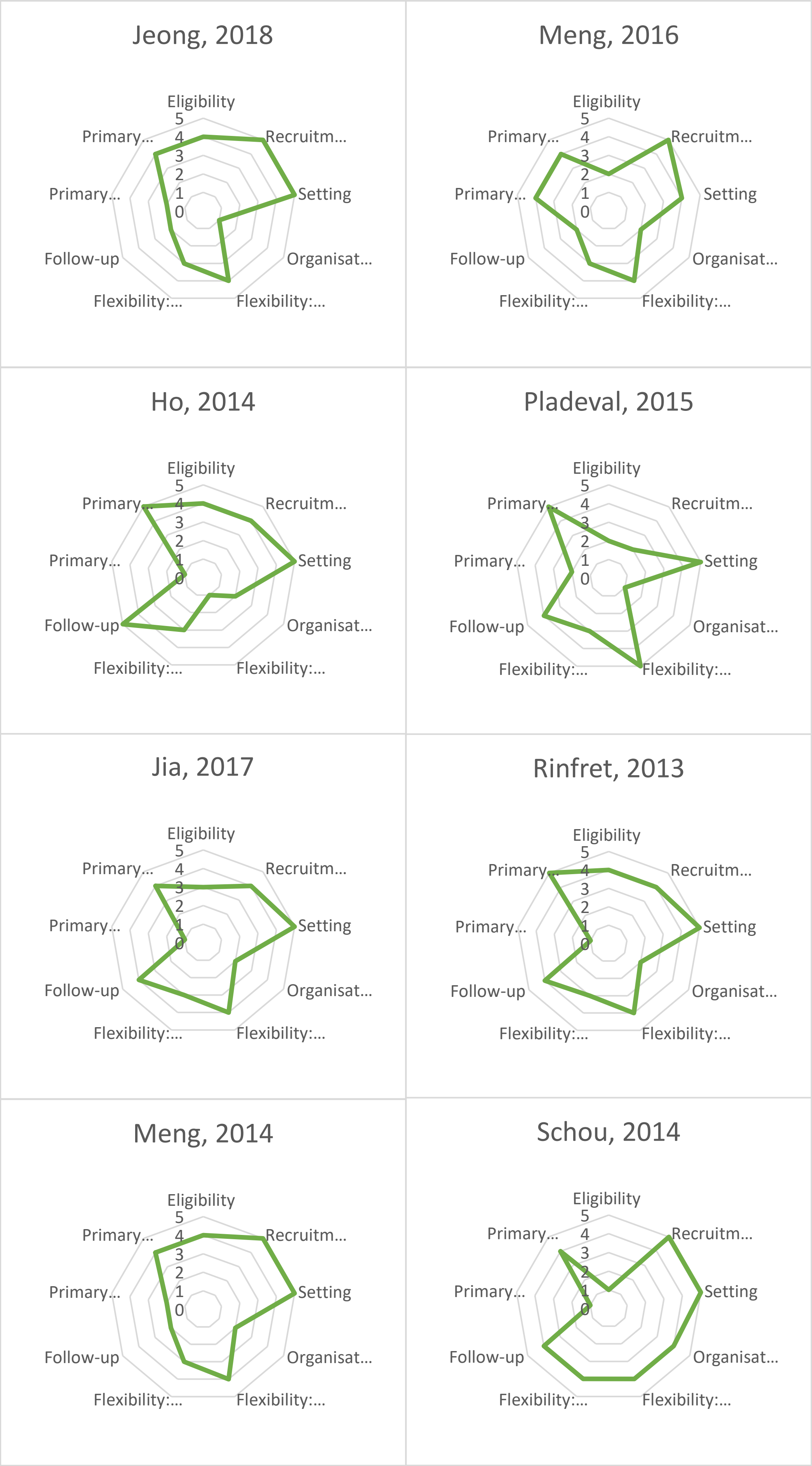
Jeong, 2018
Eligibility
Recruitm...
Setting
Organisat...
Flexibility:...
Flexibility:...
Follow-up
Primary...
Primary...
0
1
2
3
4
5
Meng, 2016
Eligibility
Recruitm...
Setting
Organisat...
Flexibility:...
Flexibility:...
Follow-up
Primary...
Primary...
0
1
2
3
4
5
Ho, 2014
Eligibility
Recruitm...
Setting
Organisat...
Flexibility:...
Flexibility:...
Follow-up
Primary...
Primary...
0
1
2
3
4
5
Pladeval, 2015
Eligibility
Recruitm...
Setting
Organisat...
Flexibility:...
Flexibility:...
Follow-up
Primary...
Primary...
0
1
2
3
4
5
Jia, 2017
Eligibility
Recruitm...
Setting
Organisat...
Flexibility:...
Flexibility:...
Follow-up
Primary...
Primary...
0
1
2
3
4
5
Rinfret, 2013
Eligibility
Recruitm...
Setting
Organisat...
Flexibility:...
Flexibility:...
Follow-up
Primary...
Primary...
0
1
2
3
4
5
Meng, 2014
Eligibility
Recruitm...
Setting
Organisat...
Flexibility:...
Flexibility:...
Follow-up
Primary...
Primary...
0
1
2
3
4
5
Schou, 2014
Eligibility
Recruitm...
Setting
Organisat...
Flexibility:...
Flexibility:...
Follow-up
Primary...
Primary...
0
1
2
3
4
5

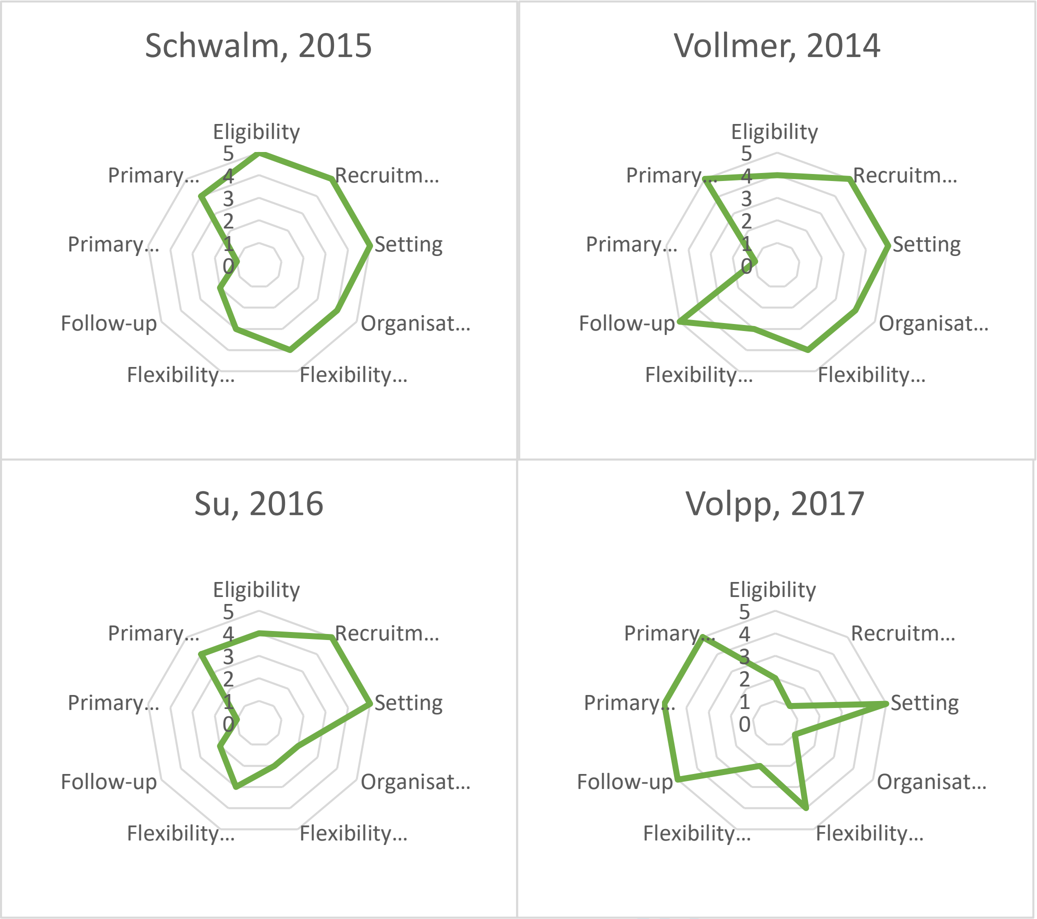
Schwalm, 2015
Vollmer, 2014
Su, 2016
Volpp, 2017
Eligibility
Recruitm...
Setting
Organisat...
Flexibility...
Flexibility...
Follow-up
Primary...
Primary...
0
1
2
3
4
5

eview only

Supplementary eFigure 2 – PRECIS-2 graphs of trials identified as explanatory

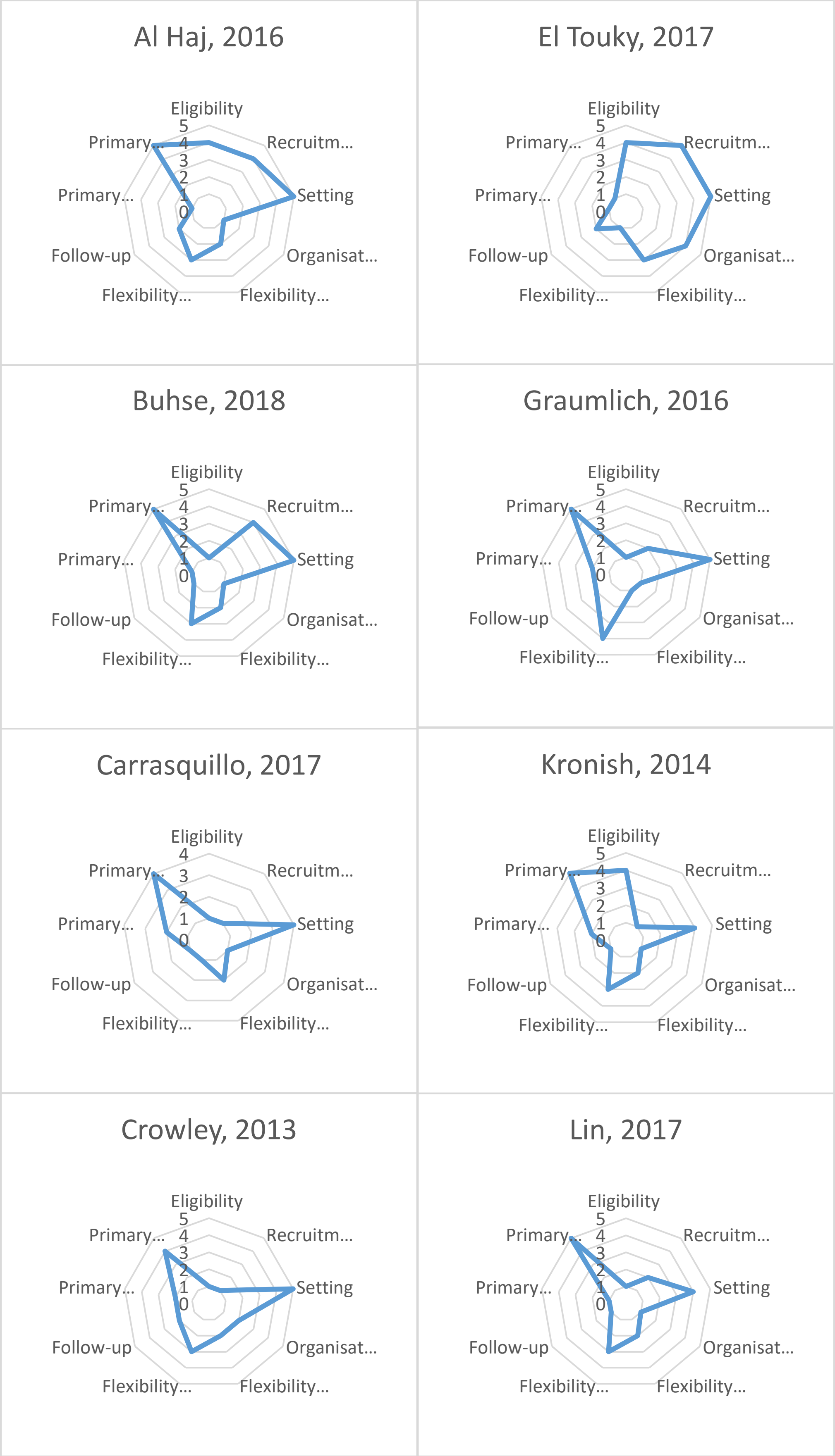

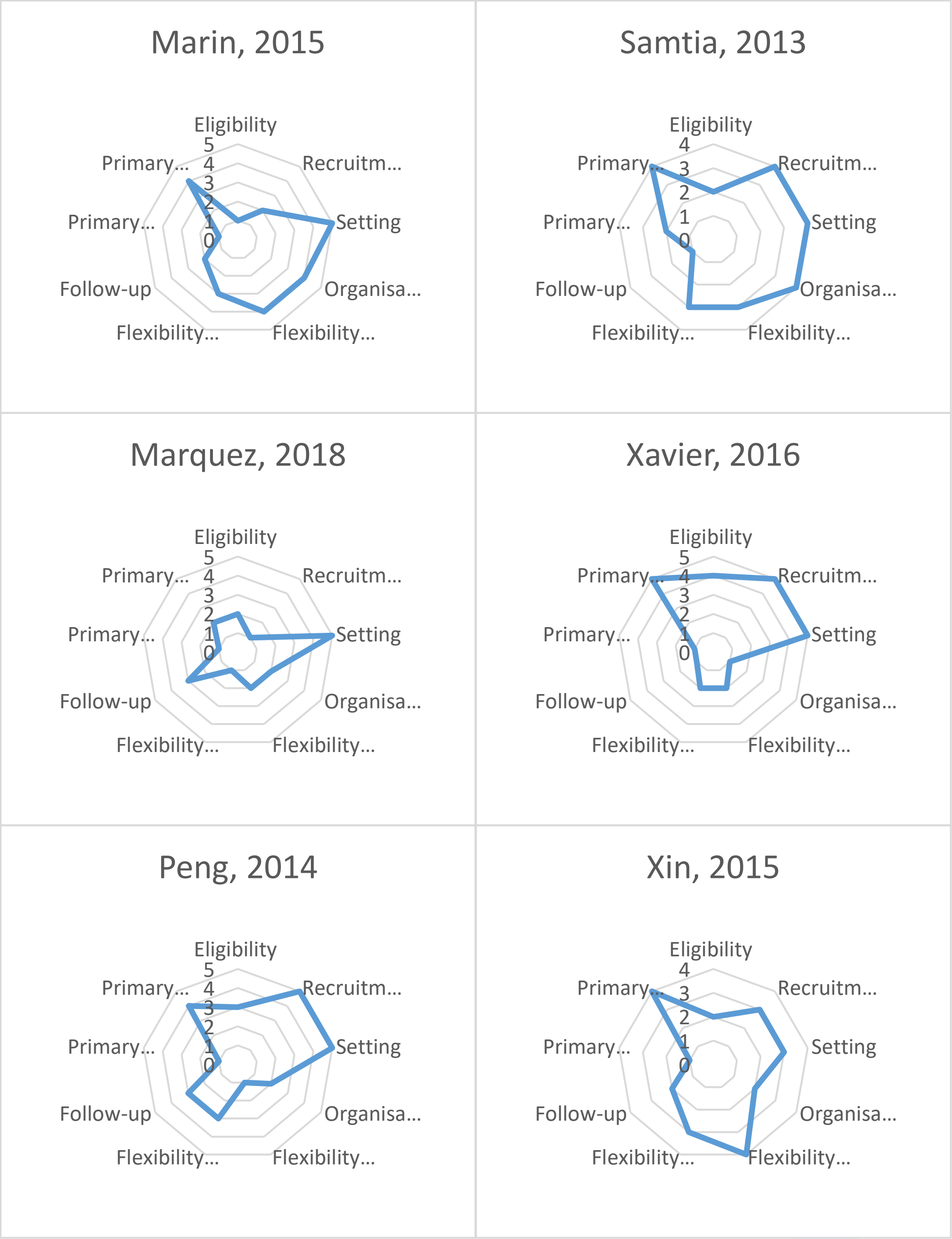
Marin, 2015
Eligibility
Recruitm...
Setting
Organisa...
Flexibility...
Flexibility...
Follow-up
Primary...
Primary...
0
1
2
3
4
5
Samtia, 2013
Eligibility
Recruitm...
Setting
Organisa...
Flexibility...
Flexibility...
Follow-up
Primary...
Primary...
0
1
2
3
4
Marquez, 2018
Eligibility
Recruitm...
Setting
Organisa...
Flexibility...
Flexibility...
Follow-up
Primary...
Primary...
0
1
2
3
4
5
Xavier, 2016
Eligibility
Recruitm...
Setting
Organisa...
Flexibility...
Flexibility...
Follow-up
Primary...
Primary...
0
1
2
3
4
5
Peng, 2014
Eligibility
Recruitm...
Setting
Organisa...
Flexibility...
Flexibility...
Follow-up
Primary...
Primary...
0
1
2
3
4
5
Xin, 2015
Eligibility
Recruitm...
Setting
Organisa...
Flexibility...
Flexibility...
Follow-up
Primary...
Primary...
0
1
2
3
4

Supplementary eFigure 3. Average PRECIS-2 wheel domain scores for studies identified as pragmatic or explanatory

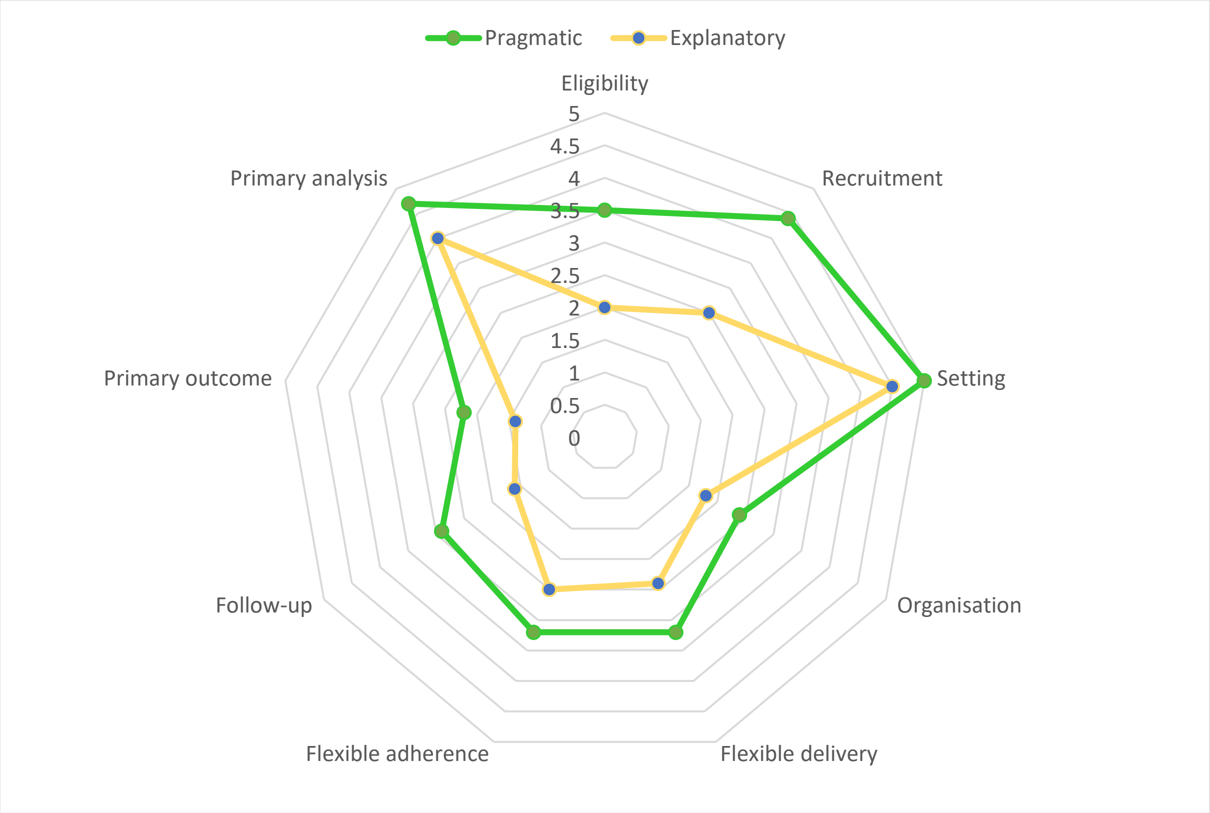

For peer review only

Supplementary eFigure 4 - Forest plot of pooled standardised mean differences for medication adherence, stratified by PRECIS score

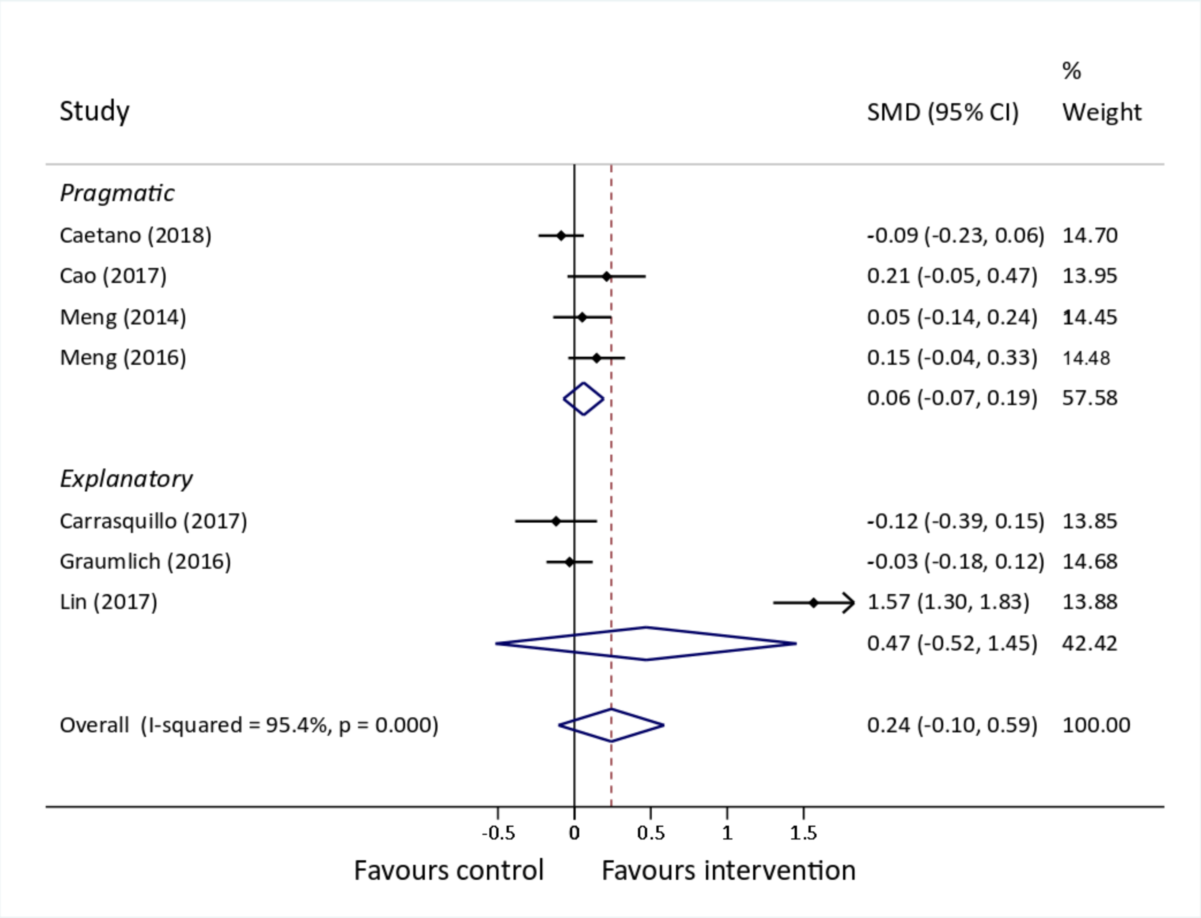

Supplementary eTable 4 - Meta-regression results showing the effects of study level covariates on medication adherence

	Coefficient (95% CI)	p-value
Odds Ratios		
Study length	0.01 (-0.01, 0.03)	0.408
Outcome (1^0 or 2^0)	0.01 (-0.39, 0.41)	0.964
Mean age	-0.02 (-0.06, 0.02)	0.317
Percent male	0.01 (-0.01, 0.02)	0.469
Disease	-0.09 (-0.42, 0.24)	0.581
Precis score	-0.02 (-0.07, 0.03)	0.367
Standardised mean differences		
Study length	0.07 (-0.04, 0.18)	0.162
Outcome (1^0 or 2^0)	-0.67 (-1.82, 0.48)	0.195
Mean age	0.06 (-0.01, 0.12)	0.071
Percent male	0.004 (-0.03, 0.04)	0.760
Disease	0.57 (-0.52, 1.65)	0.237
Precis score	-0.03 (-0.12, 0.06)	0.429

For peer review only

Supplementary eTable 5 - Results of sub-group analyses

	N studies	Effect size (95% CI)	p-value*
Odds ratios of adherence			
**Explanatory**	9	1.69 (1.24, 2.31)	0.598
**Pragmatic**	13	1.53 (1.23, 1.89)	
**Self-reported adherence measure**	15	1.63 (1.33, 1.98)	0.568
**Objective adherence measure**	7	1.47 (1.03, 2.08)	
**Multi-faceted intervention**	8	2.05 (1.62, 2.60)	0.010
**Single-faceted intervention**	14	1.28 (1.12, 1.46)	
SMD of adherence			
**Explanatory**	3	0.47 (-0.52, 1.45)	0.441
**Pragmatic**	4	0.06 (-0.07, 0.19)	
**Self-reported adherence measure**	7	*Not calculated*†	-
**Objective adherence measure**	0	*Not calculated*†	
**Multi-faceted intervention**	1	*Not calculated*†	-
**Single-faceted intervention**	6	*Not calculated*†	

*Comparing the effect size between the two sub-groups. †Pooled estimates by sub-group were not calculated where there was less than 2 studies to combine for one of the groupings

For peer review only

Supplementary eFigure 5 - Funnel plot for the meta-analysis of standardised mean differences

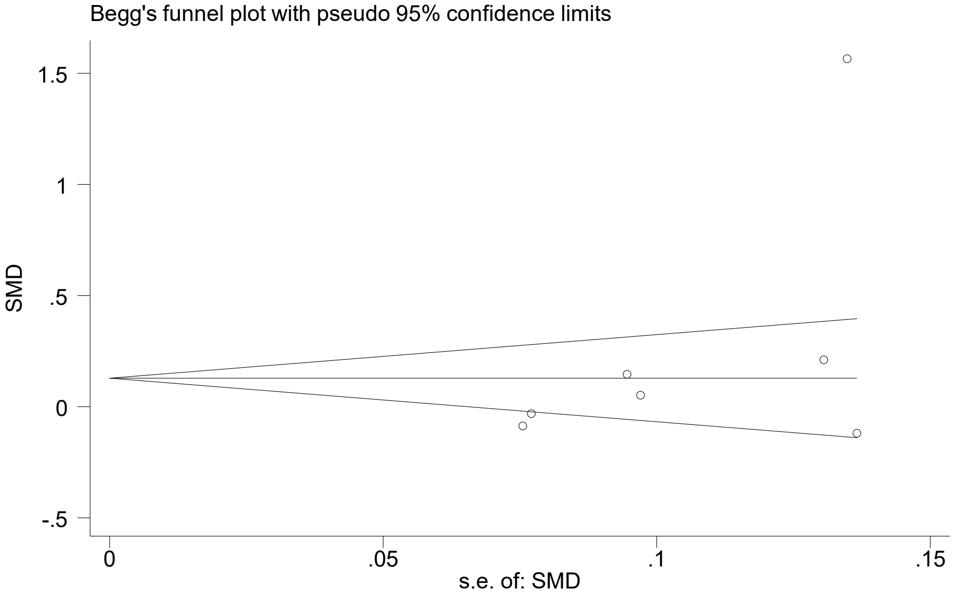

standardised mean difference of adherence

Supplementary eFigure 6 - Funnel plot for the meta-analyses of odds ratios

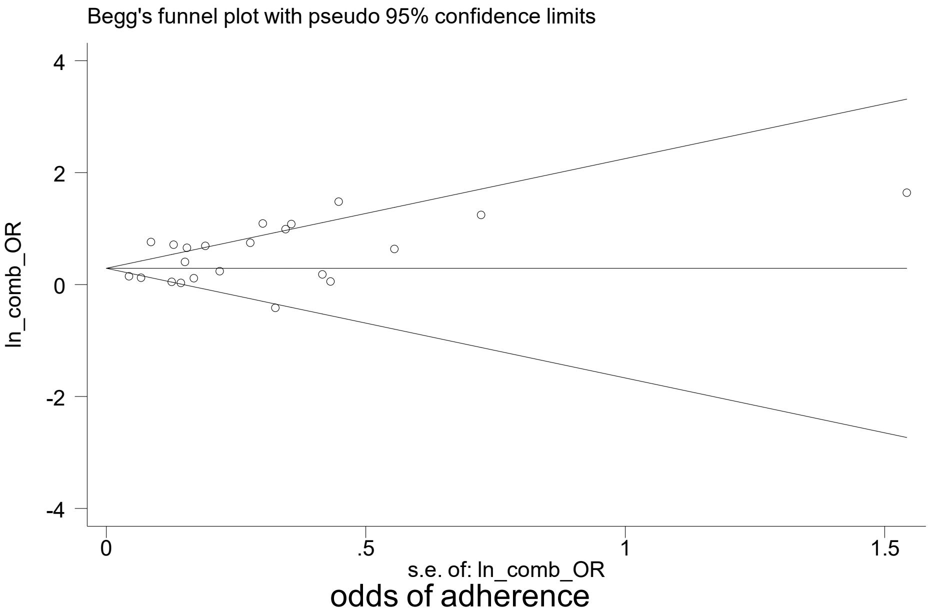

For peer review only

Supplementary eTable 6 - Number and proportion of texts which report insufficient data for PRECIS-2 domain scoring

Domains (n=9)	n	%
Eligibility	0	0
Recruitment	1	2.9
Setting	1	2.9
Organisation	0	0
Flexible delivery	2	5.9
Flexible Adherence	23	67.6
Follow-up	2	5.9
Primary outcome	0	0
Primary analysis	0	0

For peer review only

PRISMA 2009 Checklist based on draft 9

Section/topic	#	Checklist item	Reported on page #
**TITLE**			
Title	1	Identify the report as a systematic review, meta-analysis, or both.	1
**ABSTRACT**			
Structured summary	2	Provide a structured summary including, as applicable: background; objectives; data sources; study eligibility criteria, participants, and interventions; study appraisal and synthesis methods; results; limitations; conclusions and implications of key findings; systematic review registration number.	3+4
**INTRODUCTION**			
Rationale	3	Describe the rationale for the review in the context of what is already known.	5
Objectives	4	Provide an explicit statement of questions being addressed with reference to participants, interventions, comparisons, outcomes, and study design (PICOS).	5
**METHODS**			
Protocol and registration	5	Indicate if a review protocol exists, if and where it can be accessed (e.g., Web address), and, if available, provide registration information including registration number.	6
Eligibility criteria	6	Specify study characteristics (e.g., PICOS, length of follow-up) and report characteristics (e.g., years considered, language, publication status) used as criteria for eligibility, giving rationale.	6+7
Information sources	7	Describe all information sources (e.g., databases with dates of coverage, contact with study authors to identify additional studies) in the search and date last searched.	6
Search	8	Present full electronic search strategy for at least one database, including any limits used, such that it could be repeated.	26+27
Study selection	9	State the process for selecting studies (i.e., screening, eligibility, included in systematic review, and, if applicable, included in the meta-analysis).	6+7
Data collection process	10	Describe method of data extraction from reports (e.g., piloted forms, independently, in duplicate) and any processes for obtaining and confirming data from investigators.	7
Data items	11	List and define all variables for which data were sought (e.g., PICOS, funding sources) and any assumptions and simplifications made.	7
Risk of bias in individual studies	12	Describe methods used for assessing risk of bias of individual studies (including specification of whether this was done at the study or outcome level), and how this information is to be used in any data synthesis.	7

For peer review only

For peer review only - http://bmjopen.bmj.com/site/about/guidelines.xhtml

PRISMA 2009 Checklist based on draft 9

Summary measures	13	State the principal summary measures (e.g., risk ratio, difference in means).	8
Synthesis of results	14	Describe the methods of handling data and combining results of studies, if done, including measures of consistency (e.g., I^2) for each meta-analysis.	8+9

Section/topic	#	Checklist item	Reported on page #
Risk of bias across studies	15	Specify any assessment of risk of bias that may affect the cumulative evidence (e.g., publication bias, selective reporting within studies).	8+9
Additional analyses	16	Describe methods of additional analyses (e.g., sensitivity or subgroup analyses, meta-regression), if done, indicating which were pre-specified.	8+9
**RESULTS**			
Study selection	17	Give numbers of studies screened, assessed for eligibility, and included in the review, with reasons for exclusions at each stage, ideally with a flow diagram.	21
Study characteristics	18	For each study, present characteristics for which data were extracted (e.g., study size, PICOS, follow-up period) and provide the citations.	29-35
Risk of bias within studies	19	Present data on risk of bias of each study and, if available, any outcome level assessment (see item 12).	25
Results of individual studies	20	For all outcomes considered (benefits or harms), present, for each study: (a) simple summary data for each intervention group (b) effect estimates and confidence intervals, ideally with a forest plot.	10-13, 22, 23 + 42
Synthesis of results	21	Present results of each meta-analysis done, including confidence intervals and measures of consistency.	12, 22, 23 + 42
Risk of bias across studies	22	Present results of any assessment of risk of bias across studies (see Item 15).	13
Additional analysis	23	Give results of additional analyses, if done (e.g., sensitivity or subgroup analyses, meta-regression [see Item 16]).	12, 43 + 44
**DISCUSSION**			
Summary of evidence	24	Summarize the main findings including the strength of evidence for each main outcome; consider their relevance to key groups (e.g., healthcare providers,	13-15

For peer review only

PRISMA 2009 Checklist based on draft 9

		users, and policy makers).	
Limitations	25	Discuss limitations at study and outcome level (e.g., risk of bias), and at review-level (e.g., incomplete retrieval of identified research, reporting bias).	15
Conclusions	26	Provide a general interpretation of the results in the context of other evidence, and implications for future research.	16
**FUNDING**			
Funding	27	Describe sources of funding for the systematic review and other support (e.g., supply of data); role of funders for the systematic review.	16

From: Moher D, Liberati A, Tetzlaff J, Altman DG, The PRISMA Group (2009). Preferred Reporting Items for Systematic Reviews and Meta-Analyses: The PRISMA Statement. PLoS Med 6(7): e1000097. doi:10.1371/journal.pmed1000097

For more information, visit: **www.prisma-statement.org**.

For peer review only

For peer review only - http://bmjopen.bmj.com/site/about/guidelines.xhtml

Effect of pragmatic versus explanatory interventions on medication adherence in people with cardiometabolic conditions: a systematic review and meta-analysis

Journal:	*BMJ Open*
Manuscript ID	bmjopen-2019-036575.R1
Article Type:	Original research
Date Submitted by the Author:	11-Mar-2020
Complete List of Authors:	Fitzpatrick, Claire; University of Leicester Diabetes Research Centre; University Hospitals of Leicester NHS Trust, Leicester Diabetes Centre<br>Gillies, Clare; University of Leicester Diabetes Research Centre; University Hospitals of Leicester NHS Trust, Leicester Diabetes Centre<br>Seidu, Samuel; University of Leicester Diabetes Research Centre; University Hospitals of Leicester NHS Trust, Leicester Diabetes Centre<br>Kar, Debasish; University of Sheffield Department of Oncology and Metabolism,<br>Ioannidou, Ekaterini; University of Leicester Diabetes Research Centre; University Hospitals of Leicester NHS Trust, Leicester Diabetes Centre<br>Davies, Melanie; University of Leicester Diabetes Research Centre, Diabetes Research Centre; University Hospitals of Leicester NHS Trust, Leicester Diabetes Centre<br>Patel, Prashanth; University of Leicester, Department of Cardiovascular Sciences; University Hospitals of Leicester NHS Trust, Department of Chemical Pathology and Metabolic Diseases<br>Gupta, Pankaj; University of Leicester, Department of Cardiovascular Sciences; University Hospitals of Leicester NHS Trust, Department of Chemical Pathology and Metabloic Diseases<br>Khunti, Kamlesh; University of Leicester Diabetes Research Centre; University Hospitals of Leicester NHS Trust, Leicester Diabetes Centre
**Primary Subject Heading**:	Diabetes and endocrinology
Secondary Subject Heading:	Medical education and training, Research methods
Keywords:	DIABETES & ENDOCRINOLOGY, CARDIOLOGY, Diabetes & endocrinology < INTERNAL MEDICINE

SCHOLARONE™
Manuscripts

I, the Submitting Author has the right to grant and does grant on behalf of all authors of the Work (as defined in the below author licence), an exclusive licence and/or a non-exclusive licence for contributions from authors who are: i) UK Crown employees; ii) where BMJ has agreed a CC-BY licence shall apply, and/or iii) in accordance with the terms applicable for US Federal Government officers or employees acting as part of their official duties; on a worldwide, perpetual, irrevocable, royalty-free basis to BMJ Publishing Group Ltd ("BMJ") its licensees and where the relevant Journal is co-owned by BMJ to the co-owners of the Journal, to publish the Work in this journal and any other BMJ products and to exploit all rights, as set out in our licence.

The Submitting Author accepts and understands that any supply made under these terms is made by BMJ to the Submitting Author unless you are acting as an employee on behalf of your employer or a postgraduate student of an affiliated institution which is paying any applicable article publishing charge ("APC") for Open Access articles. Where the Submitting Author wishes to make the Work available on an Open Access basis (and intends to pay the relevant APC), the terms of reuse of such Open Access shall be governed by a Creative Commons licence – details of these licences and which Creative Commons licence will apply to this Work are set out in our licence referred to above.

Other than as permitted in any relevant BMJ Author's Self Archiving Policies, I confirm this Work has not been accepted for publication elsewhere, is not being considered for publication elsewhere and does not duplicate material already published. I confirm all authors consent to publication of this Work and authorise the granting of this licence.

For peer review only

ORIGINAL RESEARCH

Effect of pragmatic versus explanatory interventions on medication adherence in people with cardiometabolic conditions: a systematic review and meta-analysis

Author Names:
Claire Fitzpatrick MSc[1,2], Clare Gillies PhD[1,2], Samuel Seidu MD[1,2], Debasish Kar[3] MSc, Ekaterini Ioannidou MSc[1,2], Melanie Davies MD[1,2], Prashanth Patel FRCPath[4,5], Pankaj Gupta FRCPath[4,5], Kamlesh Khunti PhD[1,2]*

Claire Fitzpatrick & Clare Gillies contributed equally

[1] Diabetes Research Centre, University of Leicester, Leicester, UK,

[2] Leicester Diabetes Centre, University Hospitals of Leicester NHS Trust, Leicester General Hospital, Leicester, UK

[3] University of Sheffield, Sheffield, UK

[4] Department of Cardiovascular Sciences, University of Leicester, Leicester, UK

[5] Department of Chemical Pathology and Metabolic Diseases, University Hospitals of Leicester NHS Trust, Leicester, UK

Claire Fitzpatrick, (0000-0003-2150-8092), Researcher, Diabetes Research Centre, University of Leicester, Leicester General Hospital, Gwendolen Road, Leicester, LE5 4PW

Claire Gillies, (0000-0002-8417-9700), Lecturer in Medical Statistics, Diabetes Research Centre, University of Leicester, Leicester General Hospital, Gwendolen Road, Leicester, LE5 4PW

Samuel Seidu, (0000-0002-8335-7018) Primary Care Research Fellow, Diabetes Research Centre, University of Leicester, Leicester General Hospital, Gwendolen Road, Leicester, LE5 4PW

Debasish Kar, (0000-0002-1524-1312), Senior Clinical Lecturer (Honorary), University of Sheffield, Western bank, Sheffield, S10 2TN

Ekaterini Ioannidou, Research Assistant, Diabetes Research Centre, University of Leicester, Leicester General Hospital, Gwendolen Road, Leicester, LE5 4PW

Melanie Davies, (0000-0002-9987-9371), Professor of Diabetes Medicine, Diabetes Research Centre, University of Leicester, Leicester General Hospital, Gwendolen Road, Leicester, LE5 4PW

Prashanth Patel, (0000-0002-8981-2691), Consultant Metabolic Physician and Honorary Senior Lecturer and Co- Director National Centre for Adherence Testing, Department of Chemical Pathology and Metabolic Diseases, University of Leicester, Leicester Royal Infirmary, Infirmary Way, Leicester, LE1 5WW

Pankaj Gupta, (0000-0001-9481-6067), Consultant, Co- Director National Centre for Adherence Testing, Department of Chemical Pathology and Metabolic Diseases, University of Leicester, Leicester Royal Infirmary, Infirmary Way, Leicester, LE1 5WW

Corresponding author* - Kamlesh Khunti, (0000-0003-2343-7099) Professor of Primary Care, Diabetes & Vascular Medicine, Diabetes Research Centre, University of Leicester, Leicester General Hospital, Gwendolen Road, Leicester, LE5 4PW, kk22@le.ac.uk

Manuscript word count: 3499

For peer review only

For peer review only - http://bmjopen.bmj.com/site/about/guidelines.xhtml

ABSTRACT

Objective

To synthesise findings from randomised controlled trials (RCTs) of interventions aimed at increasing medication adherence in individuals with type 2 diabetes (T2DM) and/or cardiovascular disease (CVD). And, in a novel approach, to compare the intervention effect of studies which were categorised as being more pragmatic or more explanatory using the Pragmatic-Explanatory Continuum Indicator Summary-2 (PRECIS-2) tool, to identify whether study design affects outcomes. As explanatory trials are typically held under controlled conditions, findings from such trials may not be relatable to real world clinical practice. In comparison, pragmatic trials are designed to replicate real world conditions and therefore findings are more likely to represent those found if the intervention were to be implemented in routine care.

Design

Systematic review and meta-analysis

Data sources

Ovid Medline, Ovid Embase, Web of Science and CINAHL from January 1, 2013 to December 31, 2018

Eligibility criteria for selecting studies

RCTs lasting ≥3 months (90 days), involving ≥200 patients in the analysis, with either established CVD and/or T2DM and which measured medication adherence. From 4403 citations, 103 proceeded to full text review. Studies published in any language other than English and conference abstracts were excluded.

Main outcome measure

Change in medication adherence

Results

Of 4403 records identified, 34 studies were considered eligible, of which 28, including 30,861 participants, contained comparable outcome data for inclusion in the meta-analysis. Overall interventions were associated with an increase in medication adherence (odds ratio 1.57 (95% CI: 1.33, 1.84), p<0.001, standardised mean difference 0.24 (95%CI: -0.10, 0.59) p=0.101). The effectiveness of interventions did not differ significant between studies considered pragmatic versus explanatory (p=0.598), but did differ by intervention type, with studies that included a multi-faceted rather than a single-faceted intervention having a more significant effect (p=0.010). The analysis used random effect models and used the revised Cochrane Risk of Bias Tool to assess study quality.

Conclusions

In this meta-analysis interventions were associated with a significant increase in medication adherence. Overall multi-faceted interventions which included an element of education alongside regular patient contact or follow up showed the most promise. Effectiveness of interventions between pragmatic and explanatory trials was comparable, suggesting that findings can be transferred from idealised to real word conditions.

For peer review only - http://bmjopen.bmj.com/site/about/guidelines.xhtml

Study Registration

PROSPERO (ID: CRD42017059460)

For peer review only - http://bmjopen.bmj.com/site/about/guidelines.xhtml

For peer review only

Strengths and limitations

- In a novel approach, this systematic review categorised relevant studies as being more pragmatic or more explanatory using the Pragmatic-Explanatory Continuum Indicator Summary-2 (PRECIS-2) tool and, in a meta-analysis, explored whether this affected the intervention effect.
- Heterogeneity was explored using subgroup analyses.
- A few studies had to be excluded from this systematic review and meta-analysis as they did not categorise diabetes type.

For peer review only

For peer review only - http://bmjopen.bmj.com/site/about/guidelines.xhtml

Introduction

Prevalence of type 2 diabetes (T2DM) and cardiovascular diseases (CVDs) are increasing rapidly.(1) They have been identified as two of the most common cardiometabolic morbidities associated with multi-morbidity,(1) and are two of the leading causes of death worldwide.(2) Most people diagnosed with these conditions will likely have multi-morbidity(1) (coexistence of two or more chronic conditions), particularly those aged 65-84 years where prevalence is estimated at 65%.(3)

Management of multi-morbidity is complex, typically relying on the co-prescription of multiple drugs, which is strongly associated with medication non-adherence.(4) The World Health Organisation estimates that 50% of individuals receiving chronic treatment are non-adherent (taking less than 80%) to prescribed medications.(5) Medication non-adherence is considered the biggest cause of suboptimal clinical outcomes, accounting for approximately 57% of avoidable costs in relation to medication use.(5)

Despite the rise in cardiometabolic multi-morbidity,(6-8) interventions to increase medication adherence within this population are sparse. As such we decided to focus this review on T2DM and CVD as the most prevalent cluster cardiometabolic morbidities. Treatment non-adherence within these populations is well recognised, and has become the focus of considerable research. Whilst randomised controlled trials (RCTs) are widely accepted as a rigorous way of exploring the impact of interventions on specific health behaviour change outcomes, it has been identified that numerous components within a trials design can lead to biased interpretations of intervention effects.(9) One such bias may be the controlled nature of these trials and the impact they impose upon the cooperation of their participants, and may prelude an action which does not necessarily represent what may occur in routine clinical practice.(10) In pragmatic trials, the intervention is less strict and mimics usual practice as much as possible, thus lessening the unexpected reactions from the patients which lead to the biases.(11) As randomised controlled trials (RCTs) are generally expensive to conduct, it is important that their findings show real-world intervention effectiveness that is relevant to routine clinical practice. This study therefore aimed to not only identify interventions to increase medication adherence, but also compare the effectiveness of interventions categorised as explanatory (undertaken in idealised settings) or pragmatic (undertaken in real world settings). Described by Schwartz & Lellouch (1967),(12) explanatory trial are those which confirm a physiological or clinical hypothesis, in contrast, pragmatic trails are those which inform clinical or policy decisions by evidencing the effect that adoption would have on routine care. As treatment effects of explanatory trials may be larger than those observed in pragmatic trials, traditional meta-analytic approaches may not account for this heterogeneity resulting in biased estimated treatment effects. Whilst a handful of reviews in different research areas have now been published which retrospectively applied PRECIS-2 (The Pragmatic-Explanatory Continuum Indicator Summary-2) to see if comparing pragmatic with explanatory trials altered review findings,(13, 14) to the authors knowledge, this is the first review to incorporate PRECIS-2 scoring into the initial review process. Whilst retrospective application provides an interesting comparison, whereby the overall intervention effects can be compared to explanatory and/or pragmatic intervention effects, pre or post use of PRECIS-2, it could lead to an initial misinterpretation of findings. For example, a review containing a high number of explanatory trials may be much less applicable to routine clinical practice than one containing a greater number of pragmatic trials. Identification and comparison during the trial design stage would allow researchers to more easily identify how applicable findings would be to real world clinical practice, rather than making an assumption based on a generalised outcome. In addition, this approach removes any risk of bias as the analysts are unaware of the results of the study results. Using PRECIS-2 this review scored interventions from very explanatory to

For peer review only - http://bmjopen.bmj.com/site/about/guidelines.xhtml

very pragmatic to explore whether the differences in characteristics between the study designs of these trials influenced intervention effectiveness estimates in a meta-analysis.

Methods

This systematic review has been registered on PROSPERO (ID: CRD42017059460) and was conducted in accordance with PRISMA guidelines.[15]

Data sources and searches

An extensive scoping search identified the great breath of research published within this topic area. It also identified a 2014 Cochrane Review on medication adherence which included studies published up to January 11, 2013. Due to factors relating to the focus of the review, changes in prescribed medication and the increasing use of mobile technology interventions in recent adherence research, we decided that this review would provide a contemporary update and therefore we refined our literature search to include studies published between January 1, 2013 to December 31, 2018. We searched Ovid Medline 1946 (Epub ahead of print, in process and other non-indexed citations), Ovid Embase 1974, Web of Science 1970 and Cumulative Index of Nursing and Allied Health Literature (EBSCO CINAHL-plus with full text). We searched "type 2 diabetes", "cardiovascular disease", "medication adherence" and "randomised control trial" using a combination of medical subject headings, keywords and synonyms with both English and American spellings. An example search strategy can be found in (Supplementary eTable 1).

Screening

Two investigators (CF and EI) independently screened all titles, abstracts and full text articles. Discrepancies were resolved through discussion with a third researcher (CG). Studies were assessed against five eligibility criteria to determine firstly, whether the study was an RCT; second, that patients were identified as having established CVD and/or T2DM; third, the trial measured medication adherence; fourth, study duration was ≥3 months (90 days) and fifth; the study included >200 people in the analysis. Studies published in any language other than English and conference abstracts were excluded. Additional reasons for exclusion were where articles failed to specify diabetes type or where results were combined for individuals at risk of and with established CVD.

Data extraction

Data extraction was performed by CF and checked by CG. Where available the following data were recorded: study characteristics (authors, year, country, duration, sample size); participant characteristics (age, gender, disease); methods of assessment (self-report, pharmacy records, pill count); intervention type (Supplementary eTable 2); and outcome measures of medication adherence. For trials reporting multiple follow-up, the final follow-up corresponding to study duration was used.

Risk of Bias

Risk of bias assessment was undertaken using The revised Cochrane Risk of Bias Tool for Randomized Controlled Trials.[16, 17]

PRECIS-2

Using Loudon *et al.* (2015) for guidance,[18] PRECIS-2 scoring to explore how pragmatic/explanatory different components of the included study designs was undertaken independently by CF and CG. Each of the nine domains was given a score from one (very explanatory "ideal study conditions") to

For peer review only - http://bmjopen.bmj.com/site/about/guidelines.xhtml

five (very pragmatic "usual care conditions"). Results were compared and a consensus score was reached.

Some studies did not clearly explain all components making scoring difficult. In order to determine whether the treatment of unclear data affected the overall score we conducted two classification processes. For the primary method we inputted a score of 3 and then calculated an average score by adding up the scores and dividing by 9. In the second a score was not given for the missing domains and then an average was calculated based on available domain scores.

Data synthesis

Study data were reported as means and medians for continuous data and as proportions for categorical data. Twenty two studies defined a cut point to determine adherence. Twenty used a binary outcome defining individuals as adherent or non-adherent, however two categorised individuals based on a pre-specified level of low, medium, and high. Of those reporting levels of low, medium or high adherence, as has been done in previous studies,[19, 20] we combined medium and high levels and separated low level to form a binary outcome of adherence or non-adherence respectively, therefore enabling odds ratio of adherence to be calculated. In one study (Boyne, 2014), all participants in the intervention arm were adherent at follow-up, so a continuity correction of 0.5 was added to allow an odds ratio to be calculated. Where an odds ratio for medication adherence was reported in study results, this was used for the meta-analyses, rather than calculating an estimated odds ratio from the raw numbers. Where adjusted odds ratios were reported, the odds ratio adjusted for the most covariates was used in the meta-analyses.

A further six studies, plus one which was included in the previous analysis, reported adherence using a continuous scale which provided an overall group indication of adherence. For these studies, change in adherence for each study arm was calculated. As scores differed, we calculated standardised mean differences (SMD) to combine study estimates in a meta-analysis. The standardised mean difference was calculated as the mean change in medication adherence in the intervention group minus mean change in the control group, divided by the pooled standard deviation, using Cohen's method.[21]

For both meta-analysis models, funnel plots and Egger's test were carried out to assess for publication bias and random effects meta-analyses were fitted to account for heterogeneity in study design. We assessed heterogeneity between studies by calculating the Higgins I^2 statistic, with an I^2 statistic >75% considered high heterogeneity.[22] In one study (Boyne, 2014), all participants in the intervention arm were adherent at follow-up, so a continuity correction of 0.5 was added to allow an odds ratio to be calculated.

We fitted meta-regression models assessing; study length, whether adherence was a primary or secondary outcome, mean age, percent male, disease (T2DM or CVD), and PRECIS-2 score, to explore the impact of study heterogeneity on the intervention effect. Three sub-group analyses were also carried out, whereby as well as fitting a meta-regression model to compare the statistical difference between groups, separate meta-analyses were also run for each subgroup to enable the pooled estimate for each sub-group to be calculated, and hence a more explicit comparison to be made. The first compared intervention effects of studies that included a self-reported measure of adherence, to those with an objective adherence measure. The second compared pragmatic with explanatory studies (pragmatic studies were those with an average PRECIS-2 score >3 and explanatory a score of 3 or less) and the third compared outcomes of interventions which were identified as multi-faceted against those with a singular intervention component.

For peer review only - http://bmjopen.bmj.com/site/about/guidelines.xhtml

Where studies had obtained both pharmacy and self-report data, pharmacy data was used in preference in the meta-analysis. In the case of a few studies which did not report overall level of adherence and instead listed individual drug adherence, we made the pragmatic decision to monitor the adherence effect of the first drug listed by the author.

All analyses were performed using Stata 15 with the METAN, METAREG and METABIAS commands. Results are reported with 95% confidence intervals (CI) with a p value <0.05 considered statistically significant. For the few studies not included within the meta-analysis, descriptive synthesis of intervention effects is discussed.

Results

Study selection

Following deduplication we screened 4403 titles and abstracts which yielded 103 potentially relevant studies. Thirty three met the inclusion criteria with an additional study[(23)] identified from reference list and forward citation screening (Figure 1).[(23)] Of the 34 studies, 28 including 30,861 participants contained comparable outcome data for inclusion in the meta-analysis.

Characteristics of included studies

The key characteristics of included studies are shown in Supplementary eTable 3. Studies were conducted in 17 countries, with one study[(24)] conducted across four countries. Twenty three trials were conducted in high income countries,[(23, 25-46)] seven in upper middle,[(47-53)] two in lower middle,[(54, 55)] one in a low income country, and one across both upper middle and high income economies[(56)]. The proportion of males ranged from 27.9%[(31)] to 94.0%[(23)]. Four trials did not report age[(24, 26, 52, 56)] and/or gender.[(24, 26, 56)]

Medical conditions varied across studies; 12 studies were in participants with T2DM,[(25, 28-31, 34, 37, 41, 48, 53, 54, 56)] 21 in participants with CVD[(23, 24, 26, 27, 32, 33, 35, 36, 38-40, 42-44, 46, 47, 49-52, 55)] and one in people with both.[(45)] Follow up ranged from 3-36 months.

Medication adherence was assessed as a primary outcome in 19 studies.[(24, 25, 27-29, 33, 35, 36, 39, 42, 44, 45, 48, 50-53, 55, 56)] Methods of measuring medication adherence varied between studies. Adherence was primarily assessed through self-report (n=23),[(23-32, 34, 38, 40, 42, 44, 47-50, 53-56)] followed by pharmacy data (n=10),[(28, 35, 36, 41-43, 45, 46, 50, 53)] pill count (n=2),[(24, 33)] and electronic pill bottle opening (n=1).[(39)] Three studies did not report the method of medication adherence assessment used.[(37, 51, 52)] Of those measuring adherence through self-report, the Morisky-Green questionnaire[(57)] and the 8-item Morisky Medication Adherence scale (MMAS-8)[(58)] (or translated versions) were the most frequently used. Four studies measured adherence using two different methods, self-report and either pharmacy or pill count data.[(24, 28, 50, 53)] Two of the studies combined the data to provide an overall level or adherence,[(24, 28)] whereas the other two reported adherence outcomes separately.[(50, 53)] Of the two studies reporting separate outcomes, both reported consistent findings and statistically significant improvements in adherence whether self-report or pharmacy data were used.

Interventions varied greatly between studies. We categorised and defined interventions (Supplementary eTable 2) based on their most prominent feature in to one of the following seven groups; (1) behavioural/educational [(23, 26, 28-30, 33, 34, 38, 40, 41, 44, 51, 53, 54)], (2) intensified patient care[(42, 49)] (3) collaborative care[(47)] (4) simplification of drug regimen[(24)] (5) personalised drug dispensing[(56)] (6) telemonitoring/telemedicine[(27, 37, 45)] (7) multi-faceted intervention strategy.[(25, 31, 32, 35, 36, 39, 43, 46, 48, 50, 52, 55)]

For peer review only - http://bmjopen.bmj.com/site/about/guidelines.xhtml

Table 1: Shaded graph to show PRECIS-2 scoring of included studies

First Author	Year	Eligibility	Recruitment	Setting	Organisation	Flexibility: delivery	Flexibility: Adherence	Follow-up	Primary outcome	Primary analysis	Total score	Average score	Amended Total score	Amended Average score
Al Haj	2016	4	4	5	1	2	3	2	1	5	27	3.00	24	3.00
Barker collo	2015	5	5	5	1	1	3	1	5	5	31	3.44	28	3.50
Boyne	2014	5	5	5	2	1	3	1	2	5	29	3.22	26	3.25
Buhse	2018	1	4	5	1	2	3	1	1	5	23	2.56	20	2.50
Caetano	2018	4	5	5	4	5	5	3	1	5	37	4.11	34	4.25
Cao	2017	2	4	5	1	2	4	2	5	5	30	3.33	30	3.33
Carrasquillo	2017	1	1	4	1	2	1	1	2	4	17	1.89	17	1.89
Castellano	2014	4	5	5	5	5	2	2	1	5	34	3.78	34	3.78
Chung	2014	2	5	5	4	1	5	2	1	5	30	3.33	30	3.33
Crowley	2013	1	1	5	2	2	3	2	2	4	22	2.44	19	2.38
Du	2016	5	4	5	2	2	3	2	5	5	33	3.67	30	3.75
El Touky	2017	4	5	5	4	3	1	2	1	1	26	2.89	23	2.88
Graumlich	2016	1	2	5	1	1	4	2	2	5	23	2.56	23	2.56
Hedegaard	2014	4	4	5	2	2	3	4	1	5	30	3.33	27	3.38
Ho	2014	4	4	5	2	1	3	5	1	5	30	3.33	27	3.38
Jeong	2018	4	5	5	1	4	3	2	2	4	30	3.33	27	3.38
Jia	2017	3	4	5	2	4	3	4	1	4	30	3.33	27	3.38
Kronish	2014	4	1	4	1	2	3	1	2	5	23	2.56	20	2.50
Lin	2017	1	2	4	1	2	3	1	1	5	20	2.22	17	2.13
Marin	2015	1	2	5	4	4	3	2	1	4	26	2.89	23	2.88
Marquez	2018	2	1	5	2	2	1	3	1	2	19	2.11	19	2.11
Meng	2014	4	5	5	2	4	3	2	2	4	31	3.44	28	3.50
Meng	2016	2	5	4	2	4	3	2	4	4	30	3.33	27	3.38
Peng	2014	3	5	5	2	1	3	3	1	4	27	3.00	21	3.00
Pladeval	2015	2	2	5	1	5	3	4	2	5	29	3.22	26	3.25
Rinfret	2013	4	4	5	2	4	3	4	1	5	32	3.56	29	3.63
Samtia	2013	2	4	4	4	3	3	1	2	4	27	3.00	21	3.00
Schou	2014	1	5	5	4	4	4	4	1	4	32	3.56	32	3.56
Schwalm	2015	5	5	5	4	4	3	2	1	4	33	3.67	30	3.75
Su	2016	4	5	5	2	2	3	2	1	4	28	3.11	25	3.13
Vollmer	2014	4	5	5	4	4	3	5	1	5	36	4.00	33	4.13

PRECIS-2 Scoring

Details of PRECIS-2 scoring for each of the included studies can be found in Table 1. For the purpose of this study it was considered that each of the nine domains on the PRECIS-2 wheel had equal weighting. For visual clarity, PRECIS-2 results are presented as a shaded graph (Table 1) where darker shades represent more pragmatic components. We also inputted scores on the PRECIS-2 wheel (Supplementary eFigure 1, 2 & 3).

For peer review only - http://bmjopen.bmj.com/site/about/guidelines.xhtml

Volpp	2017	2	1	5	1	4	2	5	5	5	30	3.33	30	3.33
Xavier	2016	4	5	5	1	2	2	1	1	5	26	2.89	26	2.89
Xin	2015	2	3	3	2	4	3	2	1	4	24	2.67	15	2.50

Of the 34 studies, 20 studies were identified as being more pragmatic, with an average score >3 (range 3.11-4.11)[23, 24, 26, 27, 29, 32, 35-37, 40-49, 52]. Of these, most (n=18) received an average score between 3-4, demonstrating a slightly more pragmatic intention on the PRECIS-2 continuum. Two of the studies scored ≥4,[29, 45] demonstrating a greater degree of pragmatism. The majority of these studies scored poorly (score of 1) in relation to pragmatism of the primary outcome as, based on the guidance by Loudon *et al.* (2015),[18] medication adherence was not considered of obvious importance from the patients perspective and was typically assessed by methods not used routinely in primary care. Eleven studies were identified as being more explanatory receiving an average score of <3 (range 1.89-2.89).[28, 30, 31, 33, 34, 38, 39, 50, 53, 55, 56] Of these, most (n=10) received an average score between 2-3 demonstrating that they favour a more explanatory intention on the continuum. Finally, three studies were given a score of 3[25, 51, 54] suggesting that these were equally pragmatic and explanatory.

The domains within which trials scored a more pragmatic rating were recruitment, setting of the intervention and the primary analysis which received average scores of 3.7, 4.8 and 4.4 respectively. All other domains appeared more explanatory with average scores of 2.2, 2.8, 2.9, 2.4 and 1.8 for the domains of organisation, flexible delivery, flexible adherence, follow-up and primary outcome respectively. The eligibility domain was the only one to receive a score of 3.

When results were compared based on the way missing data were scored, no changes to any studies overall categorisation was observed.

Meta-analysis

Effect of interventions

Data from 22 studies were pooled in a meta-analysis (Figure 2). Irrespective of intervention type or duration, interventions significantly improved medication adherence when compared to control (OR 1.57 (95% CI: 1.33, 1.84), p<0.001). For the seven studies where adherence was reported as a continuous variable, the pooled SMD was estimated as 0.24 (95% CI: -0.10, 0.59), p=0.101) (Supplementary eFigure 4), in favour of the intervention group, but was not statistically significant.

Both meta-analyses showed statistically significant heterogeneity between studies (p<0.001) with high I^2 values of 80.5% and 95.4% for the meta-analyses of the odds ratios and SMDs respectively, where the I^2 value represents the percentage of variability due to heterogeneity rather than sampling error.

We investigated the heterogeneity by conducting meta-regression analyses (Supplementary eTable 4). No statistically significant association was found between the intervention effects and study length, mean study age, percent male, disease (CVD or T2DM), whether it was a primary or secondary outcome or PRECIS-2 score. Sub-group analyses identified no differences in the intervention effect between self-reported and objective measures of adherence, and between PRECIS-2 score (Supplementary eTable 5). Sub-group analyses comparing multi versus singular-faceted interventions showed multi-faceted interventions led to a statistically significant

For peer review only - http://bmjopen.bmj.com/site/about/guidelines.xhtml

improvement in odds of adherence (p=0.010) (Figure 3, Supplementary eTable 5). The funnel plot and Egger's test (p=0.184) showed that no indication of publication bias was present for the meta-analysis of SMDs (Supplementary eFigure 5). For the meta-analysis of odds ratios, Egger's test for publication bias was statistically significant (p=0.041), with the funnel plot asymmetry indicating a slightly uneven presence of small studies showing a favourable intervention effect (Supplementary eFigure 6).

Descriptive synthesis

Six studies did not contain comparable data for inclusion in the meta-analysis.[37, 39, 42, 43, 46, 56] Of those, four studies showed a positive intervention effect on rates of medication adherence,[37, 39, 42, 56] whilst two showed no statistically significant intervention effects.[43, 46]

Assessment of risk of bias

Table 2 displays trial specific risk of bias assessment. Few studies were deemed to be of fair quality with low risk of bias and none were considered to be free of bias. Twenty seven trials provided information about adequate sequence generation and 17 regarding allocation concealment. Incomplete outcome data was a concern in four trials and selective reporting was primarily deemed unclear (n=19) due to the number of trials without a published protocol. Blinding of participants and personnel was the main domain scoring poorly for risk of bias however the nature of these types of trials means it is often impractical to do so.

Table 2: revised Cochrane risk of bias of included studies

	Adequate random sequence generation	Allocation concealment	Blinding of participants	Blinding of personnel	Blinding of outcome assessors	Incomplete outcome data	Selective reporting	Other
Al Haj 2016	+	+	-	-	?	+	?	?
Barker Collo 2015	+	+	-	-	+	-	+	+
Boyne 2014	+	?	?	?	?	+	?	?
Buhse 2018	+	+	-	-	+	+	-	+
Caetano 2018	+	?	-	-	-	+	?	?
Cao 2017	+	+	-	-	+	+	?	?
Carrasquillo 2017	+	+	-	-	+	+	+	?
Castellano 2014	+	+	-	-	-	+	+	+
Chung 2014	?	?	-	-	-	+	?	?
Crowley 2013	+	+	-	-	-	+	+	?
Du 2016	+	+	-	-	?	+	?	?

For peer review only - http://bmjopen.bmj.com/site/about/guidelines.xhtml

El Touky 2017	?	?	?	?	?	-	?	?
Graumlich 2016	+	+	-	-	-	+	+	+
Hedegaard 2014	+	+	-	-	+	+	?	?
Ho 2014	+	+	?	?	+	+	+	+
Jeong 2018	?	?	-	-	-	+	-	?
Jia 2017	?	+	?	?	?	-	?	?
Kronish 2014	+	+	-	-	+	+	?	?
Lin 2017	+	?	?	?	+	+	?	?
Marin 2015	+	?	?	?	?	+	-	-
Marquez 2018	+	+	?	?	?	-	?	?
Meng 2014	-	-	-	-	-	+	?	?
Meng 2016	+	+	+	+	?	+	+	?
Peng 2014	+	?	?	?	?	?	+	?
Pladeval 2015	+	?	-	-	+	+	?	-
Rinfret 213	+	+	-	-	+	+	?	?
Samtia 2013	?	?	?	?	?	+	?	-
Schou 2014	+	+	-	-	-	+	+	-
Schwalm 2015	+	-	?	?	+	+	+	-
Su 2016	+	?	?	?	?	-	?	?
Vollmer 2014	+	?	-	-	-	+	?	?
Volpp 2017	+	?	-	-	+	+	-	?
Xavier 2016	+	?	-	-	-	+	+	?
Xin 2015	?	?	-	-	+	+	?	?
	**Key: +** low risk of bias, **-** high risk of bias, **?** unclear risk of bias							

For peer review only

Discussion

Medication adherence is a key component of chronic disease care yet many patients fail to follow prescribing guidelines.(5) This review identified 34 trials that evaluated the impact of interventions aimed at improving medication adherence in individuals with either T2DM and/or established CVD. Overall interventions significantly increased medication adherence.

To our knowledge this is the first review to systematically compare and synthesise medication adherence outcomes based on the PRECIS-2 classification of trials. Our review showed that interventions improve medication adherence and the PRECIS- 2 classification did not appear to affect the outcome when compared in subgroup and meta-regression analyses. This finding differs from previous research where explanatory trials have reported significantly larger effect sizes than pragmatic trials.(13, 14) This suggests that findings from these interventions are representation of real world clinical practice.

Using the PRECIS-2 tool retrospectively posed a number of challenges. Missing data in published manuscripts is a known problem when conducting systematic reviews, and posed additional problems for this review as manuscripts often did not include the level of detail required to accurately score certain domains (Supplementary eTable 6). As has been identified in previous research,(59) the most challenging domains to score were those relating to the flexibility of delivery and flexibility of participant adherence. Most studies focussed on detail the content of the intervention not its delivery and adherence. In order to define a study as having a more pragmatic

For peer review only - http://bmjopen.bmj.com/site/about/guidelines.xhtml

or explanatory tendencies, we averaged domain scores to >3 or ≤3 respectively. Whilst considered the best method, we were aware of problems which could arise as a result of combining data in this way. Combining scores could result in two or more studies receiving the same or similar scores even though individual domain scores were very different. It also meant that each domain was given equal weighting in the overall score of pragmatism. Whilst a known limitation to the authors, no published guidance on how best to report the data could be found.

We restricted our review to articles published since 2013 to provide a contemporary update following a large Cochrane review published 2014 by Nieuwlaat et al.[(60)] Similarly to the 2014 review, our findings suggest that interventions were diverse in nature and the majority were complex, involving multiple different components. Of the number of "successful" interventions, multi-faceted interventions which included an element of education alongside regular patient contact showed the most promise, suggesting that frequent engagement with the healthcare team may trigger behaviour change, or act as a reminder to undertake the behaviour. Whilst promising, considerations need to be made as to the ability of such interventions to be up-scaled and implemented. Multi-faceted interventions can be expensive and therefore their cost utility needs to be explored prior to such interventions becoming embedded in clinical care pathways.

As the number of people with access to mobile technologies has increased in the past decade, particularly in the over 60 age group, interventions which rely on frequent patient contact are becoming increasingly plausible and contact via either calls or SMS provide both a pragmatic and cheap alternative to face to face health care professional contact.[(61)] A systematic review by Changizi *et al.* (2017) looking into the effectiveness of mobile health in the elderly showed that it can be used effectively, and is widely accepted as a source of health literacy, particularly SMS messages due to their low requirement for technological competency.[(62)]

Our review highlighted a number of limitations to medication adherence research conducted to date. Firstly, the lack of gold standard method of measuring adherence makes it difficult to pool and compare outcomes. The most frequently used method within this review was self-report, particularly the tools developed by Morisky and colleagues.[(57, 58)] These tools are quick, easy and cost effective to administer making them ideal for use within a clinical care setting. The tools are validated and have shown moderate comparability with other indirect methods of medication adherence however their use has been associated with over estimation of true treatment adherence as they carry the potential for recall bias.[(63)]

Even amongst the studies utilising the Morisky scales methods of scoring and presenting data varied. For example, in the case of the Morisky-Green scale, some used a Likert scoring system,[(31)] some classed answers of no to all questions as indicative of good adherence,[(44)] whereas others classed good adherence as an answer of yes to all.[(53)] For the MMAS-8, studies such as Cao *et al.* (2017)[(47)] reported mean patient scores, with higher scores representing greater adherence. In contrast, Al Haj *et al.* (2016)[(25)] report the proportion of people categorised as low medium or highly adherent based on a score of <6, 6-7.9 or >8 respectively. Discrepancies in reporting make it difficult to compare outcomes and therefore there is a need for standardisation. The best method to measure medication adherence across disease populations, and the best approach to reporting said results, therefore continues to be an area requiring further exploration.

We also acknowledge that our findings may be limited by our strict inclusion criteria. All papers reviewed were written in English and published since 2013 and therefore results may not represent non-English and older research. Finally the lack of quality of included studies, particularly in relation to participant blinding and reporting bias could compromise the integrity of review findings. In

For peer review only - http://bmjopen.bmj.com/site/about/guidelines.xhtml

addition, there was some indication of publication bias for the meta-analysis of odds ratios (p=0.041). This suggest that some smaller studies showing a negative result might not have been published, therefore the results of this meta-analysis need to be interpreted with some caution.

Conclusion

This systematic review showed that interventions worked effectively to improve medication adherence in populations with T2DM and/or CVD. Multi-faceted interventions had the most significant effect. There is however a need to compare more standardised interventions and assess these using more uniform methods of measuring medication adherence to enable studies to be more realistically compared.

Recently there has been a focus on designing trials that are pragmatic and therefore more representative of 'real life' and the findings from this review suggest that these interventions are equally effective. There is however a need for further guidance to be developed to assist researchers in characterising and scoring studies.

For peer review only

For peer review only - http://bmjopen.bmj.com/site/about/guidelines.xhtml

Acknowledgements

Funding: The authors acknowledge support from the National Institute for Health Research Collaboration for Leadership in Applied Health Research and Care – East Midlands (NIHR CLAHRC – EM) and the Leicester Biomedical Research Centre. The views expressed are those of the authors and not necessarily those of the NHS, the NIHR or the Department of Health.

Author contributions: CF developed the study protocol, carried out the scientific literature search, screened and extracted data, undertook PRECIS-2 scoring, quality assessed studies, interpreted the results, and developed the first draft of the manuscript. CG quality assessed studies, interpreted the results, undertook data checking, PRECIS-2 scoring, performed all statistical analysis and contributed to the development of the manuscript. SS assisted with the design of the review and provided intellectual content to the drafted manuscript. DK assisted with the design of the review, applied for funding and assisted with protocol development. EI screened articles for inclusion in the review. MJD provided intellectual content to the drafted manuscript. PP and PG assisted with the design of the review. KK had the concept of the study, applied for funding, assisted with protocol development and provided intellectual oversight on the development of the manuscript. KK accepts full responsibility for the conduct of the study, has access to the data, and controlled the decision to publish. KK as the corresponding author attests that all listed authors meet authorship criteria and that no others meeting the criteria have been omitted.

The Corresponding Author has the right to grant on behalf of all authors and does grant on behalf of all authors, a worldwide licence to the Publishers and its licensees in perpetuity, in all forms, formats and media (whether known now or created in the future), to i) publish, reproduce, distribute, display and store the Contribution, ii) translate the Contribution into other languages, create adaptations, reprints, include within collections and create summaries, extracts and/or, abstracts of the Contribution, iii) create any other derivative work(s) based on the Contribution, iv) to exploit all subsidiary rights in the Contribution, v) the inclusion of electronic links from the Contribution to third party material where-ever it may be located; and, vi) licence any third party to do any or all of the above.

Competing interests: All authors have completed the Unified Competing Interest form (available on request from the corresponding author). CF, CG, SS, DK, EI, PP, PG declare: no support from any organisation for the submitted work; no financial relationships with any organisations that might have an interest in the submitted work in the previous three years; no other relationships or activities that could appear to have influenced the submitted work. MJD reports grants from Novo Nordisk, grants from Sanofi-Aventis, grants from Lilly, grants from Boehringer Ingelheim , grants from Janssen, personal fees from Novo Nordisk, personal fees from Sanofi-Aventis, personal fees from Lilly, personal fees from Merck Sharp & Dohme, personal fees from Boehringer Ingelheim, personal fees from AstraZeneca, personal fees from Janssen, personal fees from Servier, personal fees from Mitsubishi Tanabe Pharma Corporation , personal fees from Takeda Pharmaceuticals International Inc., outside the submitted work. KK reports personal fees from Amgen , personal fees from Astrazeneca, personal fees from Bayer, personal fees from NAPP, personal fees from Lilly ,

For peer review only - http://bmjopen.bmj.com/site/about/guidelines.xhtml

personal fees from Merck Sharp & Dohme , personal fees from Novartis, personal fees from Novo Nordisk , personal fees from Roche, personal fees from Berlin-Chemie AG / Menarini Group, personal fees from Sanofi-Aventis , personal fees from Servier, personal fees from Boehringer Ingelheim, grants from Pfizer , grants from Boehringer Ingelheim, grants from AstraZeneca, grants from Novartis, grants from Novo Nordisk, grants from Sanofi-Aventis, grants from Lilly, grants from Merck Sharp & Dohme, grants from Servier, outside the submitted work.

Ethical approval: Ethical approval for this meta-analysis is not required.

Data sharing: The datasets used or analysed during the current study are available from the corresponding author on reasonable request.

Transparency: The lead authors affirm that the manuscript is an honest, accurate, and transparent account of the study being reported; that no important aspects of the study have been omitted. The manuscript follows the PRISMA guidelines for reporting systematic reviews and meta-analyses.

Patient and public involvement: Patient and public involvement was undertaken prior to the development of the review to obtain an understanding of patient reasons for non-adherence. A number of themes were identified and included side effects of drugs and poor explanations from health care professionals about prescribed drugs. Following the completion of the review, alongside the views and opinions expressed during the PPI, the study team aim to develop a toolbox and education programme to support medication adherence. A second focus group is planned comprising of health professionals, with the objective of gathering their perspectives on how to promote the “toolbox” for general utilisation in clinical practice and how best to disseminate and mainstream the “toolbox” of interventions.

For peer review only

For peer review only - http://bmjopen.bmj.com/site/about/guidelines.xhtml

Reference List

1. Guthrie B, Payne K, Alderson P, McMurdo ME, Mercer SW. Adapting clinical guidelines to take account of multimorbidity. BMJ. 2012;345(oct04):e6341-e.
2. World Health Organisation. Global Health Estimates 2016: Deaths by Cause, Age, Sex, by Country, and by Region, 2000-2016. Geneva; 2018.
3. Barnett K, Mercer SW, Norbury M, Watt G, Wyke S, Guthrie B. Epidemiology of multimorbidity and implications for health care, research, and medical education: a cross-sectional study. Lancet. 2012;380(9836):37-43.
4. Marengoni A, Onder G. Guidelines, polypharmacy, and drug-drug interactions in patients with multimorbidity. BMJ (Online). 2015;350.
5. Sabaté E. Adherence to long-term therapies: evidence for action: World Health Organization; 2003.
6. Glynn LG. Multimorbidity: another key issue for cardiovascular medicine. Lancet. 2009;374(9699):1421-2.
7. Tinetti ME, Fried TR, Boyd CM. Designing health care for the most common chronic condition—multimorbidity. JAMA. 2012;307(23):2493-4.
8. Weiss CO, Boyd CM, Yu Q, Wolff JL, Leff B. Patterns of prevalent major chronic disease among older adults in the United States. JAMA. 2007;298(10):1158-62.
9. McCambridge J, Kypri K, Elbourne D. In randomization we trust? There are overlooked problems in experimenting with people in behavioral intervention trials. Journal of clinical epidemiology. 2014;67(3):247-53.
10. Godin G, Sheeran P, Conner M, Germain M. Asking questions changes behavior: mere measurement effects on frequency of blood donation. Health Psychology. 2008;27(2):179.
11. McCambridge J, Kypri K, Elbourne D. Research participation effects: a skeleton in the methodological cupboard. Journal of clinical epidemiology. 2014;67(8):845-9.
12. Schwartz D, Lellouch J. Explanatory and pragmatic attitudes in therapeutical trials. Journal of chronic diseases. 1967;20(8):637-48.
13. Sajobi TT, Li G, Awosoga O, Wang M, Menon BK, Hill MD, et al. A comparison of meta-analytic methods for synthesizing evidence from explanatory and pragmatic trials. Systematic reviews. 2018;7(1):19.
14. Yoong SL, Wolfenden L, Clinton-McHarg T, Waters E, Pettman TL, Steele E, et al. Exploring the pragmatic and explanatory study design on outcomes of systematic reviews of public health interventions: a case study on obesity prevention trials. Journal of Public Health. 2014;36(1):170-6.
15. Moher D, Liberati A, Tetzlaff J, Altman DG. Preferred reporting items for systematic reviews and meta-analyses: the PRISMA statement. Ann Intern Med. 2009;151(4):264-9.
16. Sterne JA, Savović J, Page MJ, Elbers RG, Blencowe NS, Boutron I, et al. RoB 2: a revised tool for assessing risk of bias in randomised trials. bmj. 2019;366.
17. Higgins JP, Altman DG, Gøtzsche PC, Jüni P, Moher D, Oxman AD, et al. The Cochrane Collaboration's tool for assessing risk of bias in randomised trials. BMJ. 2011;343:d5928.
18. Loudon K, Treweek S, Sullivan F, Donnan P, Thorpe KE, Zwarenstein M. The PRECIS-2 tool: designing trials that are fit for purpose. BMJ. 2015;350:h2147.
19. Khayyat SM, Khayyat SMS, Alhazmi RSH, Mohamed MM, Hadi MA. Predictors of medication adherence and blood pressure control among Saudi hypertensive patients attending primary care clinics: a cross-sectional study. PloS one. 2017;12(1):e0171255.
20. Krousel-Wood M, Islam T, Muntner P, Holt E, Joyce C, Morisky DE, et al. Association of depression with antihypertensive medication adherence in older adults: cross-sectional and longitudinal findings from CoSMO. Annals of Behavioral Medicine. 2010;40(3):248-57.
21. Cohen J. Statistical power analysis for the behavioral sciences: Routledge; 2013.
22. Higgins JP, Thompson SG, Deeks JJ, Altman DG. Measuring inconsistency in meta-analyses. BMJ. 2003;327(7414):557-60.

For peer review only - http://bmjopen.bmj.com/site/about/guidelines.xhtml

23. Meng K, Seekatz B, Haug G, Mosler G, Schwaab B, Worringen U, et al. Evaluation of a standardized patient education program for inpatient cardiac rehabilitation: impact on illness knowledge and self-management behaviors up to 1 year. Health education research. 2014;29(2):235-46.
24. Castellano JM, Sanz G, Penalvo JL, Bansilal S, Fernandez-Ortiz A, Alvarez L, et al. A polypill strategy to improve adherence: Results from the FOCUS Project. Journal of the American College of Cardiology. 2014;64(20):2071-82.
25. Al-Haj Mohd MM, Phung H, Sun J, Morisky DE. Improving adherence to medication in adults with diabetes in the United Arab Emirates. BMC Public Health. 2016;16(1):857.
26. Barker-Collo S, Krishnamurthi R, Witt E, Feigin V, Jones A, McPherson K, et al. Improving Adherence to Secondary Stroke Prevention Strategies Through Motivational Interviewing Randomized Controlled Trial. Stroke. 2015;46(12):3451-8.
27. Boyne JJ, Vrijhoef HJ, Spreeuwenberg M, De Weerd G, Kragten J, Gorgels AP, et al. Effects of tailored telemonitoring on heart failure patients' knowledge, self-care, self-efficacy and adherence: a randomized controlled trial. European Journal of Cardiovascular Nursing. 2014;13(3):243-52.
28. Buhse S, Kuniss N, Liethmann K, Muller UA, Lehmann T, Muhlhauser I. An informed shared decision making programme for patients with type 2 diabetes in primary care: Cluster randomised controlled trial. Diabetologia. 2017;60 (1 Supplement 1):S347.
29. Caetano I, Santiago LM, Marques M. Impact of written information on control and adherence in type 2 diabetes. Revista Da Associacao Medica Brasileira. 2018;64(2):140-7.
30. Carrasquillo O, Lebron C, Alonzo Y, Li H, Chang A, Kenya S. Effect of a Community Health Worker Intervention Among Latinos With Poorly Controlled Type 2 Diabetes The Miami Healthy Heart Initiative Randomized Clinical Trial. Jama Internal Medicine. 2017;177(7):948-54.
31. Crowley MJ, Powers BJ, Olsen MK, Grubber JM, Koropchak C, Rose CM, et al. The Cholesterol, Hypertension, And Glucose Education (CHANGE) study: results from a randomized controlled trial in African Americans with diabetes. American Heart Journal. 2013;166(1):179-86.
32. Du L, Dong P, Jia J, Lai L, Wang S, Yang X, et al. Impacts of intensive follow-up on the long-term prognosis of percutaneous coronary intervention in acute coronary syndrome patients - A single center prospective randomized controlled study in a Chinese population. European Journal of Preventive Cardiology. 2016;23(10):1077-85.
33. El-Toukhy H, Omar A, Abou Samra M. Effect of acute coronary syndrome patients' education on adherence to dual antiplatelet therapy Adherence to dual antiplatelet therapy. Journal of the Saudi Heart Association. 2017;29(4):252-8.
34. Graumlich JF, Wang H, Madison A, Wolf MS, Kaiser D, Dahal K, et al. Effects of a Patient-Provider, Collaborative, Medication-Planning Tool: A Randomized, Controlled Trial. Journal of Diabetes Research. 2016;2016:2129838.
35. Hedegaard U, Kjeldsen LJ, Pottegard A, Hallas J. A multifaceted pharmacist intervention to support medication adherence after stroke and transient ischemic attack. International Journal of Clinical Pharmacy. 2015;37 (1):187.
36. Ho PM, Lambert-Kerzner A, Carey EP, Fahdi IE, Bryson CL, Dee Melnyk S, et al. Multifaceted intervention to improve medication adherence and secondary prevention measures after acute coronary syndrome hospital discharge : A randomized clinical trial. JAMA Internal Medicine. 2014;174(2):186-93.
37. Jeong JY, Jeon JH, Bae KH, Choi YK, Park KG, Kim JG, et al. Smart Care Based on Telemonitoring and Telemedicine for Type 2 Diabetes Care: Multi-center Randomized Controlled Trial. Telemedicine and E-Health. 2018;24(8):604-13.
38. Kronish IM, Goldfinger JZ, Negron R, Fei K, Tuhrim S, Arniella G, et al. Effect of peer education on stroke prevention: The prevent recurrence of all inner-city strokes through education randomized controlled trial. Stroke. 2014;45(11):3330-6.
39. Marquez-Contreras E, Martell-Claros N, Marquez-Rivero S, Hermida-Campa E, Gracia-Diez C, Sanchez-Lopez E, et al. Strategies for improving dabigatran adherence for stroke prevention in

For peer review only - http://bmjopen.bmj.com/site/about/guidelines.xhtml

patients with non-valvular atrial fibrillation: education and drug intake reminders (FACILITA study). Current Medical Research and Opinion. 2018;34(7):1301-8.
40. Meng K, Musekamp G, Schuler M, Seekatz B, Glatz J, Karger G, et al. The impact of a self-management patient education program for patients with chronic heart failure undergoing inpatient cardiac rehabilitation. Patient Education & Counseling. 2016;99(7):1190-7.
41. Pladevall M, Divine G, Wells KE, Resnicow K, Williams LK. A Randomized Controlled Trial to Provide Adherence Information and Motivational Interviewing to Improve Diabetes and Lipid Control. Diabetes Educator. 2015;41(1):136-46.
42. Rinfret S, Rodes-Cabau J, Bagur R, Dery JP, Dorais M, Larose E, et al. Telephone contact to improve adherence to dual antiplatelet therapy after drug-eluting stent implantation. Heart. 2013;99(8):562-9.
43. Schou M, Gislason G, Videbaek L, Kober L, Tuxen C, Torp-Pedersen C, et al. Effect of extended follow-up in a specialized heart failure clinic on adherence to guideline recommended therapy: NorthStar Adherence Study. European Journal of Heart Failure. 2014;16(11):1249-55.
44. Schwalm JD, Ivers NM, Natarajan MK, Taljaard M, Rao-Melacini P, Witteman HO, et al. Cluster randomized controlled trial of Delayed Educational Reminders for Long-term Medication Adherence in ST-Elevation Myocardial Infarction (DERLA-STEMI). American Heart Journal. 2015;170(5):903-13.
45. Vollmer WM, Owen-Smith AA, Tom JO, Laws R, Ditmer DG, Smith DH, et al. Improving Adherence to Cardiovascular Disease Medications With Information Technology. American Journal of Managed Care. 2014;20:sp502-10.
46. Volpp KG, Troxel AB, Mehta SJ, Norton L, Zhu J, Lim R, et al. Effect of electronic reminders, financial incentives, and social support on outcomes after myocardial infarction the heartstrong randomized clinical trial. JAMA Internal Medicine. 2017;177(8):1093-101.
47. Cao XY, Tian L, Chen L, Jiang XL. Effects of a hospital-community partnership transitional program in patients with coronary heart disease in Chengdu, China: A randomized controlled trial. Japan Journal of Nursing Science. 2017;14(4):320-31.
48. Chung WW, Chua SS, Lai PSM, Chan SP. Effects of a pharmaceutical care model on medication adherence and glycemic control of people with type 2 diabetes. Patient Preference and Adherence. 2014;8:1185-94.
49. Jia JJ, Dong PS, Du LJ, Li ZG, Lai LH, Yang XM, et al. Impact of Physician-Coordinated Intensive Follow-Up on Long-Term Medical Costs in Patients with Unstable Angina Undergoing Percutaneous Coronary Intervention. Acta Cardiologica Sinica. 2017;33(2):173-81.
50. Lin CY, Yaseri M, Pakpour AH, Malm D, Brostrom A, Fridlund B, et al. Can a Multifaceted Intervention Including Motivational Interviewing Improve Medication Adherence, Quality of Life, and Mortality Rates in Older Patients Undergoing Coronary Artery Bypass Surgery? A Multicenter, Randomized Controlled Trial with 18-Month Follow-Up. Drugs & Aging. 2017;34(2):143-56.
51. Peng B, Ni J, Anderson CS, Zhu Y, Wang Y, Pu C, et al. Implementation of a structured guideline-based program for the secondary prevention of ischemic stroke in China. Stroke. 2014;45(2):515-9.
52. Su Q, Li C, Long F, Chen B, Wan Z, Wu Y, et al. Effects of a health promotion program on medication adherence to antiplatelet therapy among ischemic stroke patients in Hainan Province, China. Vascular. 2017;25(3):242-8.
53. Xin CW, Xia ZN, Jiang C, Lin MM, Li GH. Effect of pharmaceutical care on medication adherence of patients newly prescribed insulin therapy: a randomized controlled study. Patient Preference and Adherence. 2015;9:797-802.
54. Samtia AM, Rasool MF, Ranjha NM, Usman F, Javed I. A multifactorial intervention to enhance adherence to medications and disease-related knowledge in type 2 diabetic patients in Southern Punjab, Pakistan. Tropical Journal of Pharmaceutical Research. 2013;12(5):851-6.
55. Xavier D, Gupta R, Kamath D, Sigamani A, Devereaux PJ, George N, et al. Community health worker-based intervention for adherence to drugs and lifestyle change after acute coronary

For peer review only - http://bmjopen.bmj.com/site/about/guidelines.xhtml

syndrome: a multicentre, open, randomised controlled trial. The Lancet Diabetes & Endocrinology. 2016;4(3):244-53.
56. Marin GH, Risso P, Sbatella D, Haag G. Treatment adherence by personalizing the drug dispensing for diabetic patients in social vulnerable situation. Quality in Primary Care. 2015;23(2):93-6.
57. Morisky DE, Green LW, Levine DM. Concurrent and predictive validity of a self-reported measure of medication adherence. Med Care. 1986:67-74.
58. Morisky DE, Ang A, Krousel-Wood M, Ward HJ. Predictive validity of a medication adherence measure in an outpatient setting. J Clin Hypertens. 2008;10(5):348-54.
59. Johnson KE, Neta G, Dember LM, Coronado GD, Suls J, Chambers DA, et al. Use of PRECIS ratings in the National Institutes of Health (NIH) health care systems research Collaboratory. Trials. 2016;17(1):32.
60. Nieuwlaat R, Wilczynski N, Navarro T, Hobson N, Jeffery R, Keepanasseril A, et al. Interventions for enhancing medication adherence. Cochrane Database of Syst Rev. 2014(11).
61. Zhou J, Rau P-LP, Salvendy G. Age-related difference in the use of mobile phones. Universal Access in the Information Society. 2014;13(4):401-13.
62. Changizi M, Kaveh MH. Effectiveness of the mHealth technology in improvement of healthy behaviors in an elderly population—a systematic review. Mhealth. 2017;3.
63. Stirratt MJ, Dunbar-Jacob J, Crane HM, Simoni JM, Czajkowski S, Hilliard ME, et al. Self-report measures of medication adherence behavior: recommendations on optimal use. Transl Behav Med. 2015;5(4):470-82.

For peer review only

For peer review only - http://bmjopen.bmj.com/site/about/guidelines.xhtml

Figure Legends

Figure 1 – PRISMA flow chart of included studies

Figure 2 – Forest plot of pooled odds ratios for medication adherence, stratified by PRECIS score

Figure 3 – Forest plot of odds ratios for adherence, stratified by complexity of the intervention

Supplementary eFigure 1– PRECIS-2 graphs of trials identified as pragmatic

Supplementary eFigure 2 – PRECIS-2 graphs of trials identified as explanatory

Supplementary eFigure 3. Average PRECIS-2 wheel domain scores for studies identified as pragmatic or explanatory

Supplementary eFigure 4 - Forest plot of pooled standardised mean differences for medication adherence, stratified by PRECIS score

Supplementary eFigure 5 - Funnel plot for the meta-analysis of standardised mean differences

Supplementary eFigure 6. Funnel plot for the meta-analyses of odds ratios

For peer review only

For peer review only - http://bmjopen.bmj.com/site/about/guidelines.xhtml

Figure 1- PRISMA flowchart of included studies

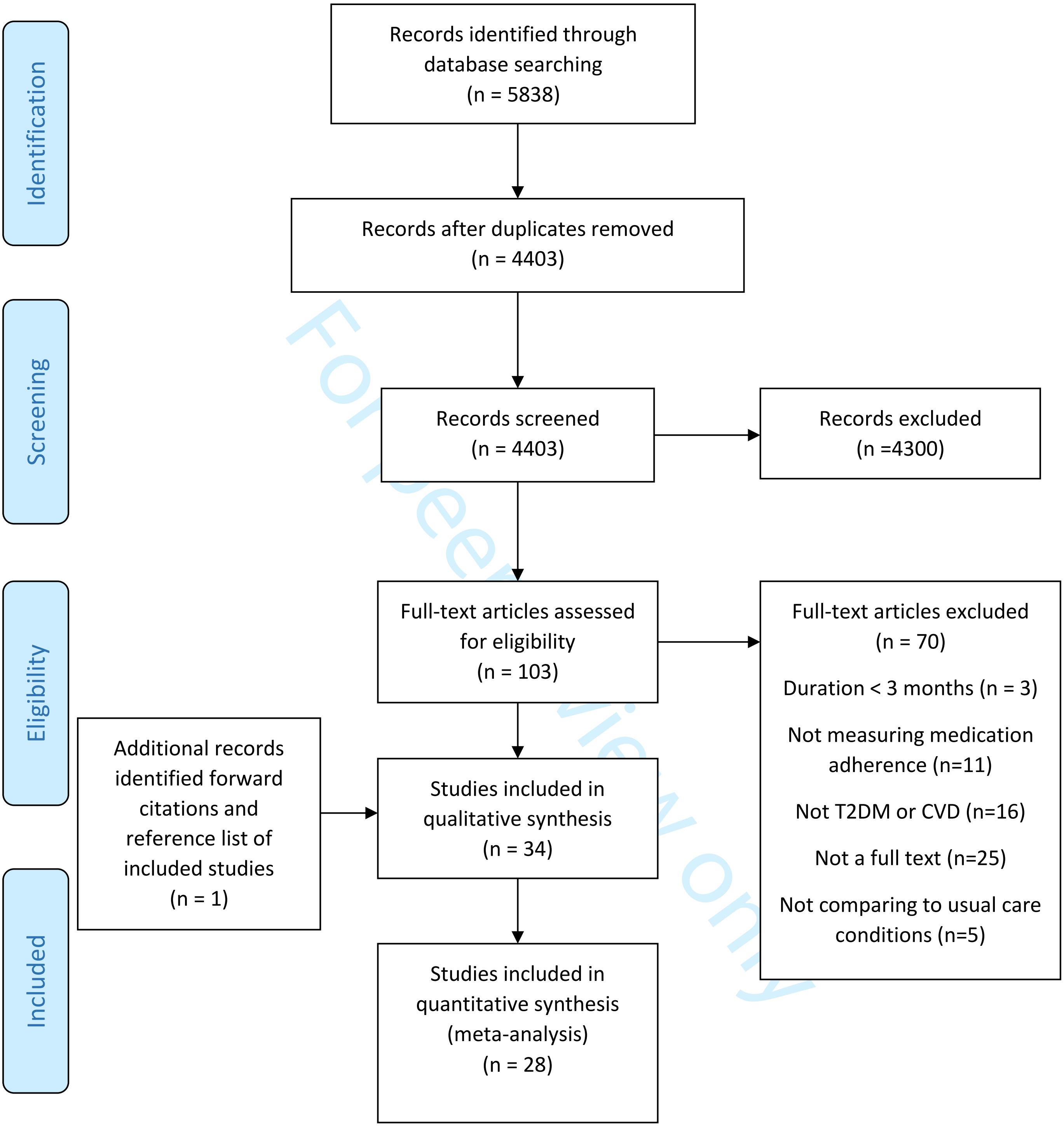

For peer review only

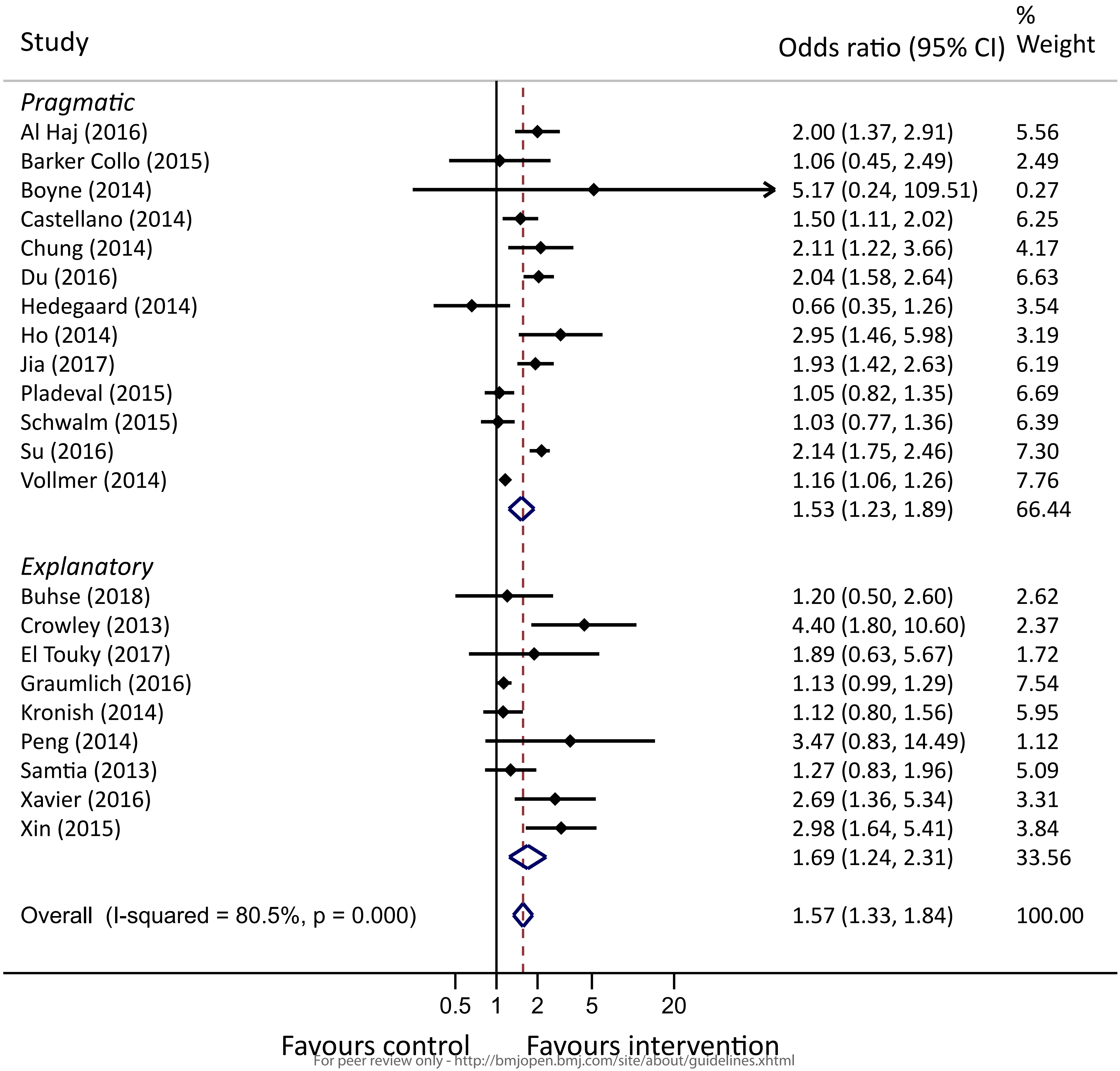
Study
Odds ratio (95% CI)
% Weight
Pragmatic
Al Haj (2016) 2.00 (1.37, 2.91) 5.56
Barker Collo (2015) 1.06 (0.45, 2.49) 2.49
Boyne (2014) 5.17 (0.24, 109.51) 0.27
Castellano (2014) 1.50 (1.11, 2.02) 6.25
Chung (2014) 2.11 (1.22, 3.66) 4.17
Du (2016) 2.04 (1.58, 2.64) 6.63
Hedegaard (2014) 0.66 (0.35, 1.26) 3.54
Ho (2014) 2.95 (1.46, 5.98) 3.19
Jia (2017) 1.93 (1.42, 2.63) 6.19
Pladeval (2015) 1.05 (0.82, 1.35) 6.69
Schwalm (2015) 1.03 (0.77, 1.36) 6.39
Su (2016) 2.14 (1.75, 2.46) 7.30
Vollmer (2014) 1.16 (1.06, 1.26) 7.76
1.53 (1.23, 1.89) 66.44
Explanatory
Buhse (2018) 1.20 (0.50, 2.60) 2.62
Crowley (2013) 4.40 (1.80, 10.60) 2.37
El Touky (2017) 1.89 (0.63, 5.67) 1.72
Graumlich (2016) 1.13 (0.99, 1.29) 7.54
Kronish (2014) 1.12 (0.80, 1.56) 5.95
Peng (2014) 3.47 (0.83, 14.49) 1.12
Samtia (2013) 1.27 (0.83, 1.96) 5.09
Xavier (2016) 2.69 (1.36, 5.34) 3.31
Xin (2015) 2.98 (1.64, 5.41) 3.84
1.69 (1.24, 2.31) 33.56
Overall (I-squared = 80.5%, p = 0.000) 1.57 (1.33, 1.84) 100.00
0.5
1
2
5
20
Favours control
Favours intervention

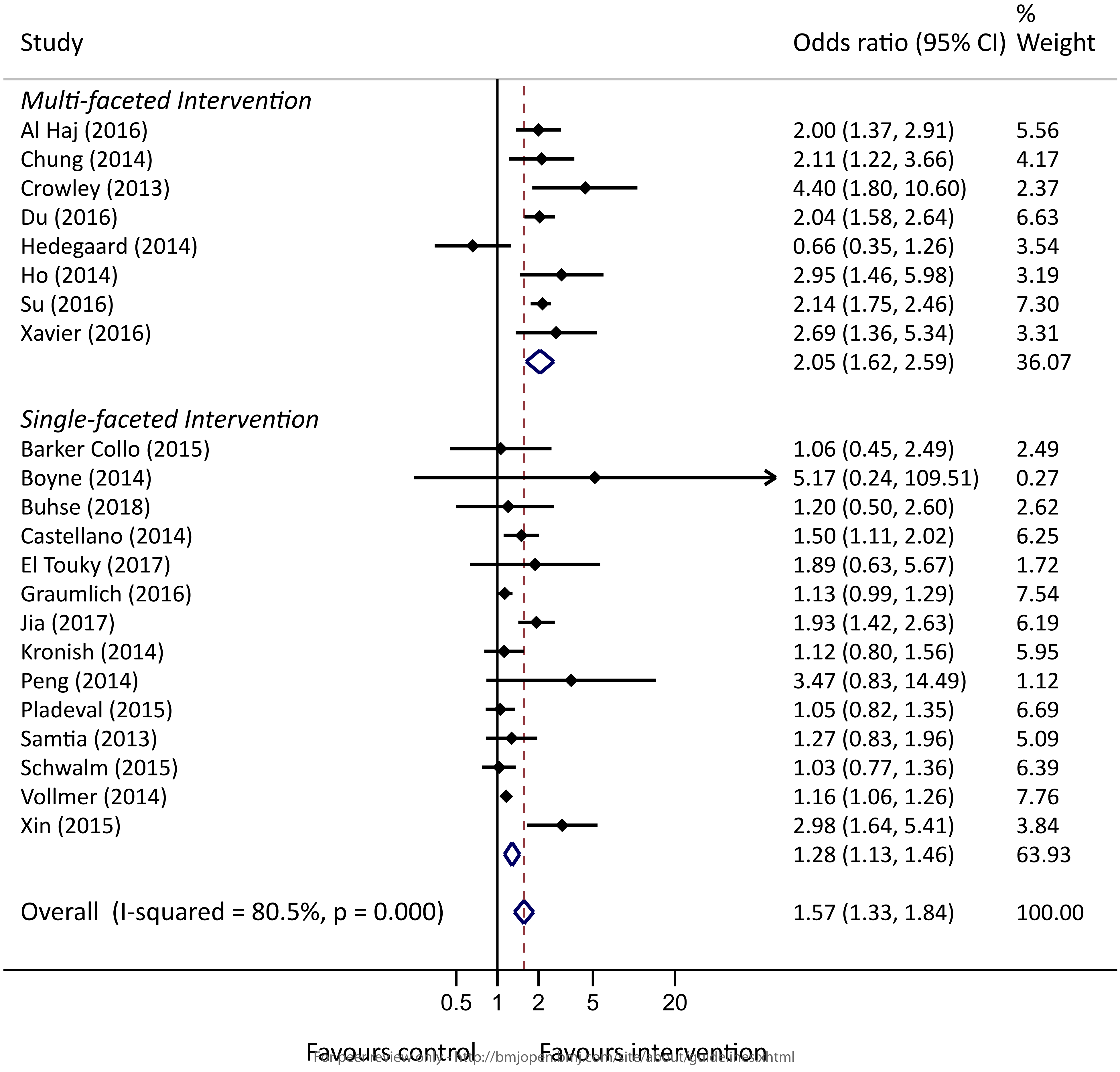

Study
Odds ratio (95% CI)
% Weight
Multi-faceted Intervention
Al Haj (2016) 2.00 (1.37, 2.91) 5.56
Chung (2014) 2.11 (1.22, 3.66) 4.17
Crowley (2013) 4.40 (1.80, 10.60) 2.37
Du (2016) 2.04 (1.58, 2.64) 6.63
Hedegaard (2014) 0.66 (0.35, 1.26) 3.54
Ho (2014) 2.95 (1.46, 5.98) 3.19
Su (2016) 2.14 (1.75, 2.46) 7.30
Xavier (2016) 2.69 (1.36, 5.34) 3.31
2.05 (1.62, 2.59) 36.07
Single-faceted Intervention
Barker Collo (2015) 1.06 (0.45, 2.49) 2.49
Boyne (2014) 5.17 (0.24, 109.51) 0.27
Buhse (2018) 1.20 (0.50, 2.60) 2.62
Castellano (2014) 1.50 (1.11, 2.02) 6.25
El Touky (2017) 1.89 (0.63, 5.67) 1.72
Graumlich (2016) 1.13 (0.99, 1.29) 7.54
Jia (2017) 1.93 (1.42, 2.63) 6.19
Kronish (2014) 1.12 (0.80, 1.56) 5.95
Peng (2014) 3.47 (0.83, 14.49) 1.12
Pladeval (2015) 1.05 (0.82, 1.35) 6.69
Samtia (2013) 1.27 (0.83, 1.96) 5.09
Schwalm (2015) 1.03 (0.77, 1.36) 6.39
Vollmer (2014) 1.16 (1.06, 1.26) 7.76
Xin (2015) 2.98 (1.64, 5.41) 3.84
1.28 (1.13, 1.46) 63.93
Overall (I-squared = 80.5%, p = 0.000) 1.57 (1.33, 1.84) 100.00
0.5 1 2 5 20
Favours control
Favours intervention

Online only supplement

Supplementary eTable 1 – Medline Search Strategy

Supplementary eTable 2 – Definitions of Intervention components

Supplementary eTable 3 – Characteristics of included studies

Supplementary eFigure 1– PRECIS-2 graphs of trials identified as pragmatic

Supplementary eFigure 2 – PRECIS-2 graphs of trials identified as explanatory

Supplementary eFigure 3. Average PRECIS-2 wheel domain scores for studies identified as pragmatic or explanatory

Supplementary eFigure 4 - Forest plot of pooled standardised mean differences for medication adherence, stratified by PRECIS score

Supplementary eTable 4 - Meta-regression results showing the effects of study level covariates on medication adherence

Supplementary eTable 5 - Results of sub-group analyses

Supplementary eFigure 5 - Funnel plot for the meta-analysis of standardised mean differences

Supplementary eFigure 6. Funnel plot for the meta-analyses of odds ratios

Supplementary eTable 6 - Number and proportion of texts which report insufficient data for PRECIS-2 domain scoring

For peer review only

Supplementary eTable 1 – Medline Search Strategy

1	exp patient compliance/ or patient* adher*.mp.
2	exp medication compliance/ or medication* adher*.mp.
3	((patient* or medication* or medicine* or drug* or therap* or treatment*) adj2 (adher* or concordance or non adher* or nonadher* or compliance or non compliance or noncompliance or refusal)).mp. [mp=title, abstract, original title, name of substance word, subject heading word, floating sub-heading word, keyword heading word, protocol supplementary concept word, rare disease supplementary concept word, unique identifier, synonyms]
4	treatment refusal/ or treatment* refusal.mp.
5	exp *non insulin dependent diabetes mellitus/ or Type 2 diabet*.mp.
6	T2D*.mp.
7	((non insulin or noninsulin) adj dependent diabet*).mp. [mp=title, abstract, original title, name of substance word, subject heading word, floating sub-heading word, keyword heading word, protocol supplementary concept word, rare disease supplementary concept word, unique identifier, synonyms]
8	NIDD*.mp.
9	Diabetes Mellitus/ or diabet*.mp.
10	*cardiovascular disease/ or cardiovascular diseas*.mp. or CVD.mp. [mp=title, abstract, original title, name of substance word, subject heading word, floating sub-heading word, keyword heading word, protocol supplementary concept word, rare disease supplementary concept word, unique identifier, synonyms]
11	((heart or cardiac or cardiovascular or coronary) adj2 (disease* or disorder* or failure*)).mp. [mp=title, abstract, original title, name of substance word, subject heading word, floating sub-heading word, keyword heading word, protocol supplementary concept word, rare disease supplementary concept word, unique identifier, synonyms]
12	*heart arrhythmia/ or arrhythmia*.mp.
13	*atrial fibrillation/ or atrial fibrillat*.mp.
14	*heart infarction/ or myocardial infarct*.mp.
15	heart failure.mp. or *heart failure/
16	*ischemic heart disease/ or *Myocardial Ischemia/ or myocard* isch*.mp.
17	angina.mp. or *angina pectoris/

18	*cardiomyopathy/ or cardiomyopath*.mp.
19	*coronary artery disease/ or coronary artery diseas*.mp.
20	peripheral artery diseas*.mp. or *peripheral occlusive artery disease/ or *peripheral vascular disease/
21	stroke.mp. or *cerebrovascular accident/
22	*transient ischemic attack/ or transient isch* attack.mp.
23	TIA.mp.
24	controlled clinical trial.mp. or controlled clinical trial/
25	"randomized controlled trial (topic)"/ or RCT.mp.
26	clinical trial/
27	(randomi?ed controlled adj2 trial).mp. [mp=title, abstract, original title, name of substance word, subject heading word, floating sub-heading word, keyword heading word, protocol supplementary concept word, rare disease supplementary concept word, unique identifier, synonyms]
28	1 or 2 or 3 or 4
29	5 or 6 or 7 or 8 or 9 or 10 or 11 or 12 or 13 or 14 or 15 or 16 or 17 or 18 or 19 or 20 or 21 or 22 or 23
30	24 or 25 or 26 or 27
31	28 and 29 and 30
32	limit 31 to (english language and humans and yr="2013 - 2018")

only

Supplementary eTable 2 – Definitions of Intervention components	
Behavioural/educational	Educational interventions comprising of motivational interviewing, counselling, group or individual education sessions on disease, medication and general self-management and exercise classes. In general, these studies comprised of some form of structured education and or a motivational interviewing component.
Telemonitoring	Out-patient appointments were changed for telemonitoring visits, participants were contacted at regular intervals using web-based feedback, or interactive voice recognition when patients were due or overdue their medications
Collaborative care	Enhanced communication between hospital and homecare teams
Simplification of drug regimen	Simplification of drug regimen by using a polypill
Intensified patient care	Patients were contacted more regularly than usual or drug availability and compliance was more closely monitored
Personalised drug dispensing	Weekly contact via telephone or home visits with the participant to check drug availability and adherence
Multi-faceted	Varying combinations of the aforementioned interventions with electronic pill caps and pill boxes as additional interventions

Classification of intervention types were determined based on guidance obtain from previous adherence research[1, 2]

For peer review only

Supplementary eTable 3 – **Characteristics of included studies**

First Author, year	Country	Sample size (analysed)<br>Duration<br>Data collection points	Condition	Key intervention detail	Medication adherence measure (adherence definition or method of reporting)
Al Haj Mohd, 2016[(3)]	United Arab Emirates	446 (446)<br>6 months<br>0 & 6 month	T2DM	Multifaceted intervention strategy<br>• Education - 30 minute session<br>• Intensified patient care - weekly phone calls for 3 months)	Self-report - MMAS-8<br>Higher scores = better adherence
Barker Collo 2015[(4)]	Australia	386 (326)<br>12 months<br>0, 3, 6, 9 & 12 months	Stroke/TIA	Behavioural/Educational<br>• 4 x motivational interviewing sessions which occurred either face to face or via telephone, initial interview 60-90mins, remainder 30 minutes	Self-report – asked whether in the last 7 days they had taken all their medication as prescribed, indicate number of doses/pills missed, reason for missed doses and any side effects if noticed.<br>Cross checked self-report with pharmacy data for validity.
Boyne, 2014[(5)]	Netherlands	382 (382)<br>12 months<br>0, 3, 6 12 months	Heart failure	Tele-monitoring/telemedicine<br>• Replace 2 of the 4 yearly outpatient appointments with tele-monitoring	Self-report – European heart failure self-care behavior scale
Buhse, 2018[(6)]	Germany	279 (279)<br>6 months<br>0, 3, 6, 9 & 12 months	T2DM	Behavioural/Educational<br>• Decision aid about primary prevention of MI<br>• 90 minute group education session about complication prevention<br>• Patient document with defined treatment goals	Pharmacy data<br>Self-report via telephone interview
Caetano, 2018[(7)]	Portugal	709 (702)<br>6 months<br>0 & 6 months	T2DM	Behavioural/Educational<br>• Patient given one of 3 educational leaflets on diabetes, diabetes treatment or physical activity	MAT scale<br>Higher scores=better adherence
Cao, 2017[(8)]	China	236 (236)<br>90 days	Coronary heart disease	Collaborative care<br>• Enhanced communication between hospital and home care team	Self-report - MMAS-8<br>Higher scores = better adherence

For peer review only

		0, 30 & 90 days			
Carrasquillo, 2017[(9)]	United States	300 (215)<br><br>12 months<br><br>0 & 12 months	T2DM	Behavioural/Educational<br>• Education - (mentoring by a community health worker approx. 4 home visits and 12 calls)<br>• Education – monthly group education sessions<br>• Behavioral – Bimonthly group exercise classes	Self-report - MMAS-8<br>Higher scores = better adherence
Castellano, 2014[(10)]	Argentina, Italy, Spain & Paraguay	695 (594)<br><br>9 months<br><br>0, 1, 4, 9 months	Myocardial infarction	Simplification of drug regimen<br>• Fixed dose combination polypill containing Aspirin, Simvastatin, Ramipril	Pill count<br>Self-report – Morisky-Green-Levine Medication Adherence Scale
Chung, 2014[(11)]	Malaysia	241 (241)<br><br>12 months<br><br>0, 4, 8 12 months	T2DM	Multifaceted intervention strategy<br>• Education – on diabetes, hypertension, hyperlipidemia and medications<br>• Medication aids – pill box and blood glucose monitoring equipment<br>• Intensified patient care – medication reviews and monthly follow up calls	Self-report - MMAS-8 (Revised Malaysian version)<br>Higher scores = better adherence
Crowley, 2013[(12)]	United States	359 (329)<br><br>12 months<br><br>0, 3, 6, 9 & 12 months	T2DM	Multifaceted intervention strategy<br>• Education – monthly self-management education telephone calls<br>• Personalised medication management – nurse and primary care provider contact at 3 monthly intervals to discuss medication adherence and possible medication changes	Self-report Morisky-Green-Levine Medication Adherence Scale
Du, 2016[(13)]	China	979 (964)<br><br>36 months<br><br>0 & 36 months	Percutaneous coronary intervention	Multifaceted intervention strategy<br>• Intensified patient care/education/personalized medication management - regular telephone follow ups at 1, 2, 3, 6, 12 & 36 months to discuss medication usage, provide health education and disease prevention strategies and schedule lab tests and treatment adjustments where necessary	Self-report Morisky-Green-Levine Medication Adherence Scale<br>Lower score = better adherence<br>(Score 0-2 = adherent)
El Touky, 2017[(14)]	Saudi Arabia	321 (276)		Behavioural/Educational intervention	Pill count >80% classed as adherent

For peer review only

		12 months<br><br>0, 1, 3, 6, 9, & 12 months	Acute coronary syndrome	• Educated patients on several aspects of coronary artery disease		
Graumlich, 2016[15]	United States	674 (674)<br><br>12 months<br><br>0, immediately following intervention, 3 & 6 months	T2DM	Medication monitoring table<br>• Multi-media tool embedded within the electronic medical record that aims to:<br>1. Promote patient knowledge<br>2. Support collaborative medication planning		Self-report -PMAQ -(patient medication adherence questionnaire)<br>Four questions were asked regarding whether the patient had missed taking a dose yesterday, the day before yesterday, 3 days ago, and over the past weekend. Patients were rated as having proper adherence if they self-reported no missed doses in this time period
Hedegaard, 2014[16]	Denmark	211 (203)<br><br>12 months<br><br>0, 3, 6, 9 & 12 months	Stroke/ TIA	Multifaceted intervention strategy<br>• Personalized medication management-medication review by pharmacist and advice on relevant drug related problems<br>• Behavioral – 3 x 30 minute patient motivational interview to support adherence and lifestyle change		Pharmacy data - MPR >0.8
Ho, 2013[17]	United States	253 (241)<br><br>12 months<br><br>0 & 12 months	Acute coronary syndrome	Multifaceted intervention strategy<br>• Personalized medication management - Medication reconciliation and tailoring by pharmacist and provision of pill box<br>• Education - Medication education<br>• Collaborative care – Pharmacist contacted patients<br>• Telemedicine – Medication reminder and refill calls		Pharmacy refill data >0.8
Jeong, 2018[18]	South Korea	338 (338)<br><br>24 weeks	T2DM	Tele-monitoring/telemedicine (Home telemonitoring composed of web based	Tele-monitoring/telemedicine Reminders (Home telemonitoring	Does not report how medication adherence was assessed.

For peer review only

		0 & 24 weeks		feedback and education with conventional management)	with remote management)	
Jia, 2017[19]	China	669 (669)<br><br>36 months<br><br>0, 1, 3, 6, 12 & 36 months	Percutaneous coronary intervention	Intensified patient care<br>• Regular phone calls with face to face appointments scheduled as necessary		Self-report Morisky-Green-Levine Medication Adherence Scale<br>Lower score = better adherence (Score 0-2 = adherent)
Kronish, 2014[20]	United States	600 (600)<br><br>6 months<br><br>0 & 6 months	Stroke/ TIA	Behavioural/Educational intervention<br>• 6 x 90 minute weekly peer led workshops about self-management skills and medication adherence		Self-report -MMAS-8<br>Higher scores = better adherence
Lin, 2017[21]	Iran	288 (288)<br><br>18 months<br><br>0, 6, 12 & 18 months	Coronary artery bypass grafting	Multifaceted intervention strategy<br>• Educational - 3 x 60 minute weekly education sessions<br>• Behavioral – 5 x 50 minute weekly motivational interviewing<br>• Telemedicine – 4 SMS reminders sent each month, content varied on a monthly basis		Self-report – MARS (5 item)<br>Score range 5-25- Higher scores = better adherence<br>Pharmacy data
Marin, 2015[22]	Argentina	467 (459)<br><br>12 months<br><br>0 & 12 months	T2DM	Personalised medication management<br>• Weekly contact with patient to check drug availability and compliance either by phone or home visit		Self-report Morisky-Green-Levine Medication Adherence Scale<br>Higher scores = better adherence
Marquez, 2018[23]	Spain	726 (625)<br><br>18 months<br><br>0, 6 & 12 months	Atrial Fibrillation	Multifaceted intervention strategy<br>• Education – healthcare and patient discussion of an educational booklet about medication and adherence<br>• Medication aid – medication reminder calendar		MEMs
Meng, 2014[24]	Germany	471 (425)<br><br>12 months	Coronary heart disease	Behavioural/Educational intervention<br>• 5 x 45minute patient centered self-management group education program		Self-report - MARS (German version)

		Admission, discharge, 6 & 12 months			Higher scores = better adherence
Meng, 2016[25]	Germany	513 (449)<br><br>12 months<br><br>Admission, discharge, 6 & 12 months	Heart failure	Behavioural/Educational intervention<br>• 5 x 60minute patient centered self-management group education program	Self-report MARS (German version)<br>Higher scores = better adherence
Peng, 2014[26]	China	3821 (3330)<br><br>12 months<br><br>Discharge, 6, 9 & 12 months	Stroke/ TIA	Behavioural/Educational intervention<br>• Lifestyle modification - diet, exercise, smoking and risk factor control	Does not report
Pladevall 2015[27]	United states	1692 (1512)<br><br>18 months<br><br>0, 6, 12 & 18 months	T2DM	Behavioural/Educational intervention<br>• Adherence information provided to physicians to relay to patients<br>• Above plus motivational interviewing	Pharmacy data -PDC
Rinfret, 2013[28]	Canada	300 (300)<br><br>12 months<br><br>0 & 12 months	Percutaneous coronary intervention	Intensified patient care<br>• Telephone follow-up within 7 days of DES implantation and at 1, 6, and 9 months	Pharmacy refill data
Samtia, 2013[29]	Pakistan	348 (348)<br><br>5 months<br><br>0 & 5 months	T2DM	Behavioural/Educational<br>• Continual education by pharmacists about effects of complications, medication adherence, diet, exercise, foot care, smoking cessation and HbA1c	Self-report – adherent yes/no
Schou, 2014[30]	Denmark	921 (920)<br><br>13-72 months	Heart failure	Multi-faceted intervention strategy<br>• Intensified patient care – regular drug monitoring and access to regular telephone consultations	Pharmacy data - PDC >0.8

For peer review only

		Every 1-3 months		• Education – occurred if adherence decreased during drug monitoring	
Schwalm, 2015[31]	Canada	852 (852)<br><br>12 months<br><br>0, 3 & 12 months	Myocardial infarction	Behavioural/Educational<br>• Personalised letters at 1, 5, 8 and 11 months after angiogram which stated the importance and role of each medication.<br>• Prompt to talk to physician and pharmacist about meds	• Self-report – yes/no<br>• Self-report Morisky-Green-Levine Medication Adherence Scale perfect score %
Su, 2016[32]	China	1275 (1187)<br><br>12 months<br><br>0 & 12 months	Stroke/ TIA	Multi-faceted intervention strategy<br>• Education 3 x 30 minute sessions and easy to understand manual<br>• Intensified patient care – physician contact at 1, 3 and 6 months to monitor progress	Does not report
Vollmer, 2014[33]	United States	21752 (21752)<br><br>12 months<br><br>0 & 12 months	T2DM and/or CVD	Telemonitoring/telemedicine<br>• Interactive voice recognition (IVR) calls when pts due or overdue medications<br>• Enhanced IVR as above plus letter, EMR feedback, mailed materials	Pharmacy data - PDC >0.8
Volpp, 2017[34]	United States	1509 (1503)<br><br>12 months<br><br>0 & 12 months	Myocardial infarction	Multifaceted intervention strategy<br>• Medication aids - Electronic pill bottles used in place of regular pill bottles (Glow caps)<br>• Financial incentives – Patients included in a daily lottery with the potential to win $5<br>• Medication support partner – information on patients adherence shared with a support partner whose role it was to offer support and encouragement<br>• Intensified patient care – engagement advisor and social workers to provide support on adherence issues	Pharmacy data - PDC
Xavier, 2016[35]	India	805 (750)<br><br>12 months	Acute coronary syndrome	Multifaceted intervention strategy<br>• Behavioral/educational - Mentoring by a community health worker<br>• Medication aids - medication reminder calendar and	Pharmacy data -Composite medical adherence score >80%

For peer review only

		0 & 12 months		patient dairy which contained details on drugs, how to take them and benefits.	
Xin, 2015[(36)]	China	240 (227)<br>12 months<br>0 & 12 months	T2DM	Behavioural/Educational<br>• Individualised education<br>• Group education/activities<br>• Telephone counselling	• Prescription refill claims – 80-115<br>• Morisky-Green – perfect score %
Abbreviations- T2DM: type 2 diabetes; TIA: Transient Ischemic Attack; MMAS-8; Morisky Medication Adherence Scale (8-item); PDC: proportion of days covered; MARS-D: Medication Adherence Report Scale (German version); MPR: Medication Possession Ratio; MEMs: medication event monitoring system; 0 months =baseline					

For peer review only

Supplementary eFigure 1– PRECIS-2 graphs of trials identified as pragmatic

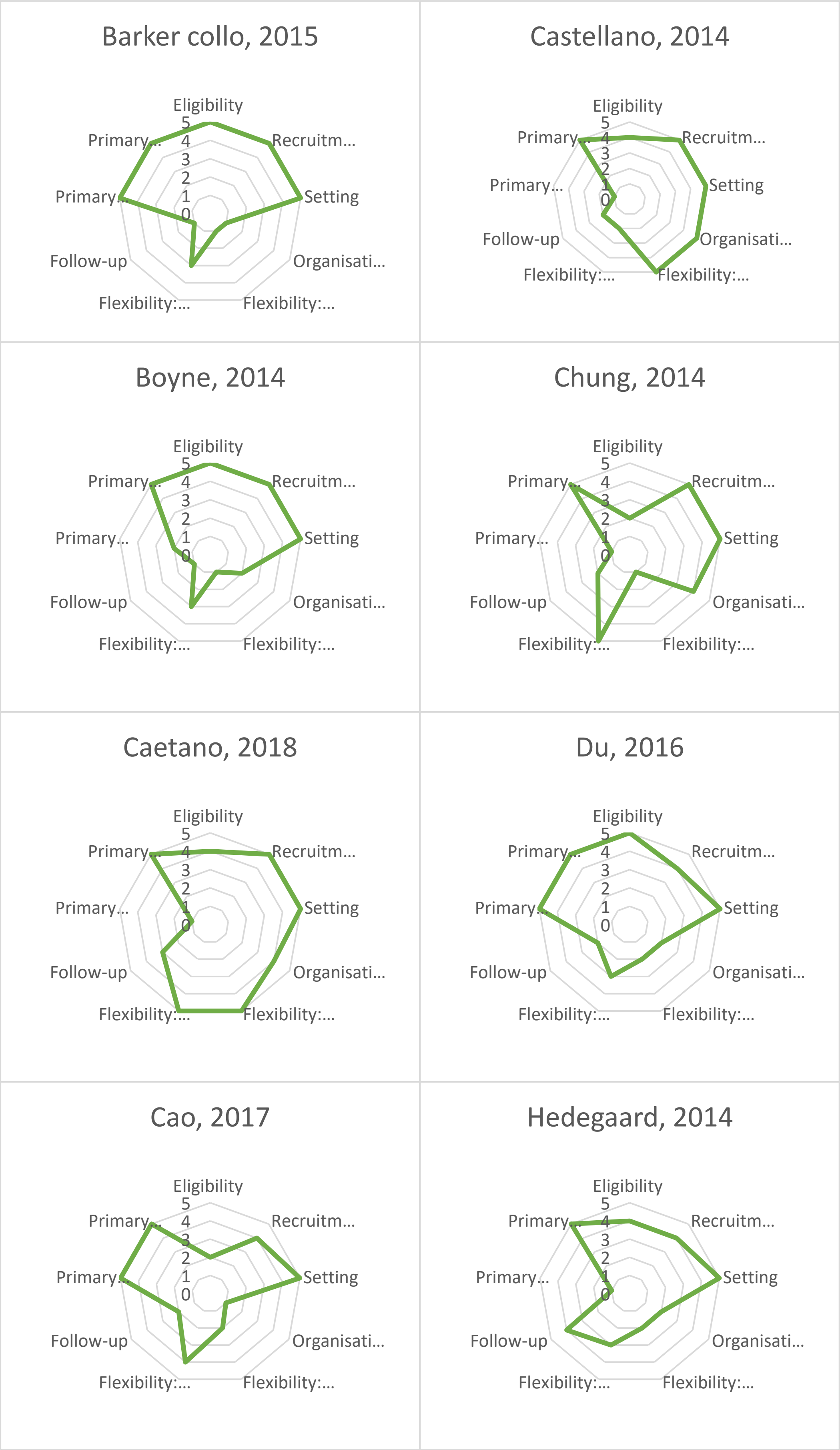

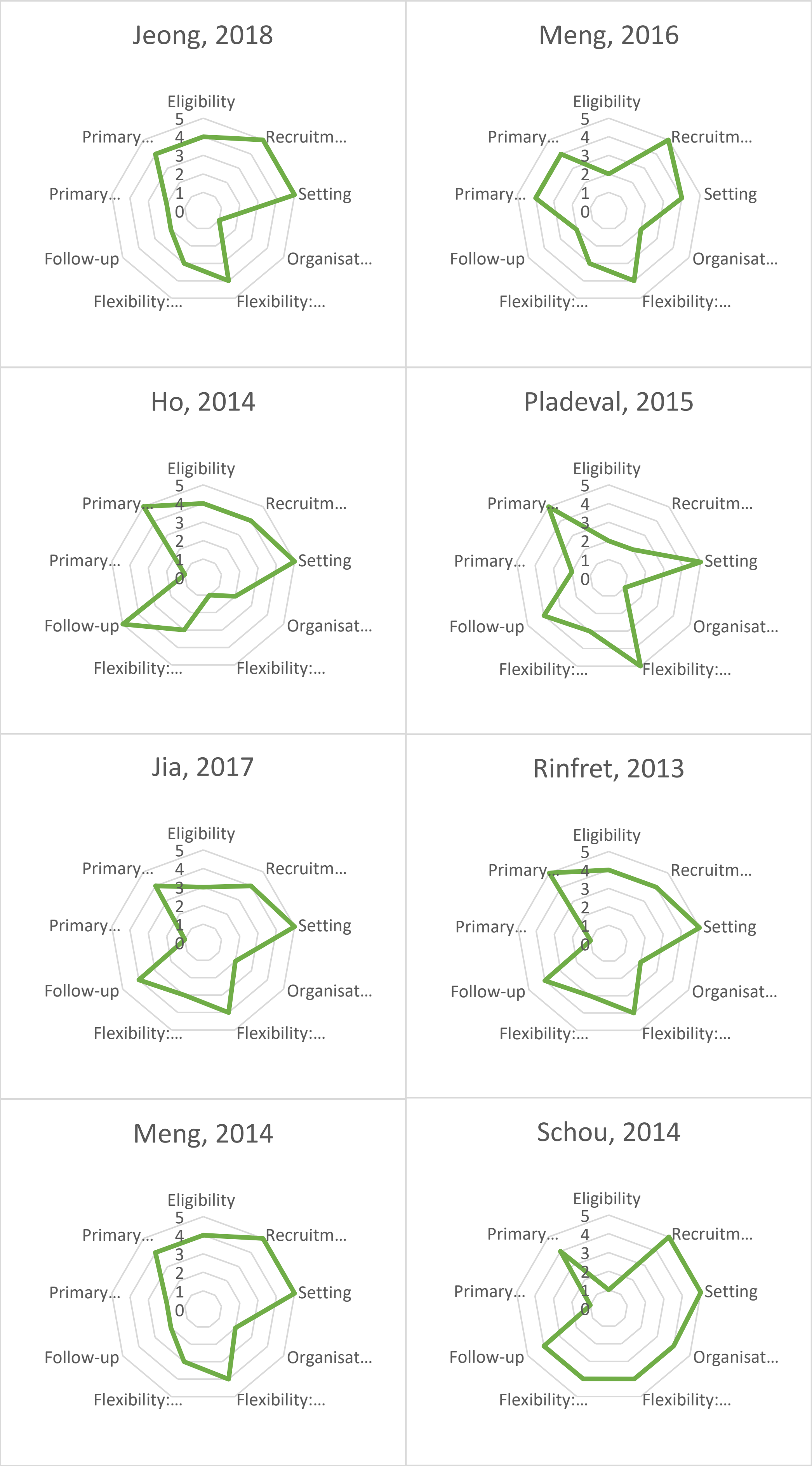
Jeong, 2018
Eligibility
Recruitm...
Setting
Organisat...
Flexibility:...
Flexibility:...
Follow-up
Primary...
Primary...
0
1
2
3
4
5
Meng, 2016
Eligibility
Recruitm...
Setting
Organisat...
Flexibility:...
Flexibility:...
Follow-up
Primary...
Primary...
0
1
2
3
4
5
Ho, 2014
Eligibility
Recruitm...
Setting
Organisat...
Flexibility:...
Flexibility:...
Follow-up
Primary...
Primary...
0
1
2
3
4
5
Pladeval, 2015
Eligibility
Recruitm...
Setting
Organisat...
Flexibility:...
Flexibility:...
Follow-up
Primary...
Primary...
0
1
2
3
4
5
Jia, 2017
Eligibility
Recruitm...
Setting
Organisat...
Flexibility:...
Flexibility:...
Follow-up
Primary...
Primary...
0
1
2
3
4
5
Rinfret, 2013
Eligibility
Recruitm...
Setting
Organisat...
Flexibility:...
Flexibility:...
Follow-up
Primary...
Primary...
0
1
2
3
4
5
Meng, 2014
Eligibility
Recruitm...
Setting
Organisat...
Flexibility:...
Flexibility:...
Follow-up
Primary...
Primary...
0
1
2
3
4
5
Schou, 2014
Eligibility
Recruitm...
Setting
Organisat...
Flexibility:...
Flexibility:...
Follow-up
Primary...
Primary...
0
1
2
3
4
5

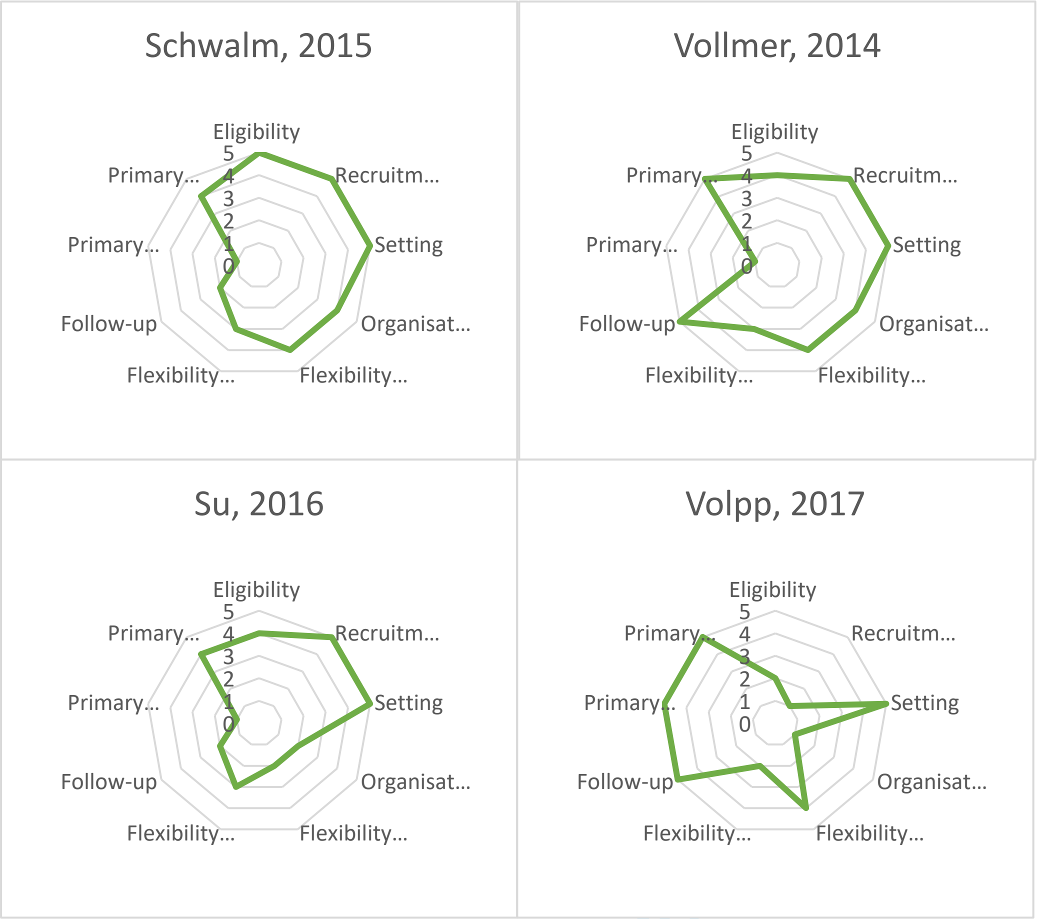

For peer review only

Supplementary eFigure 2 – PRECIS-2 graphs of trials identified as explanatory

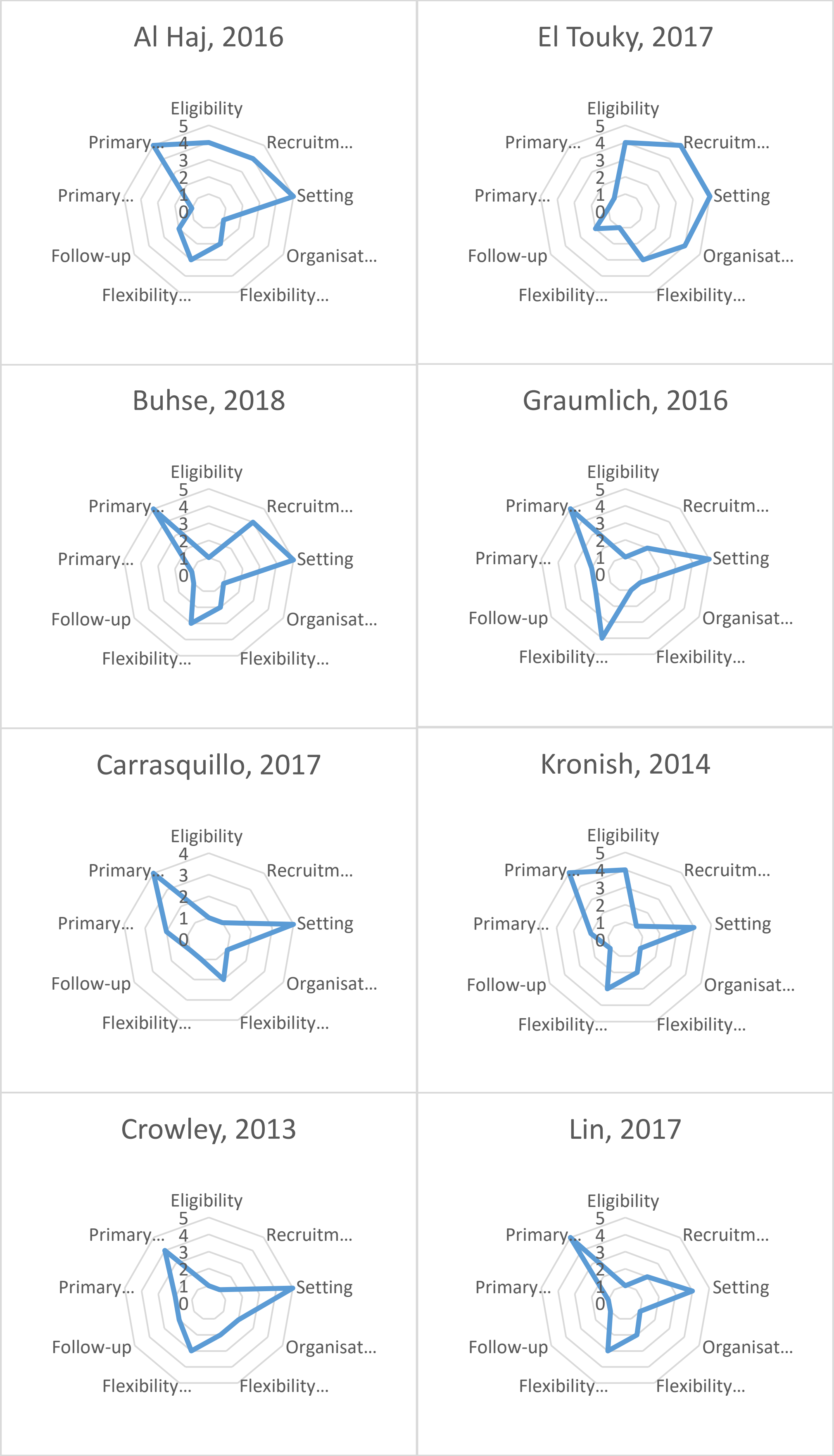

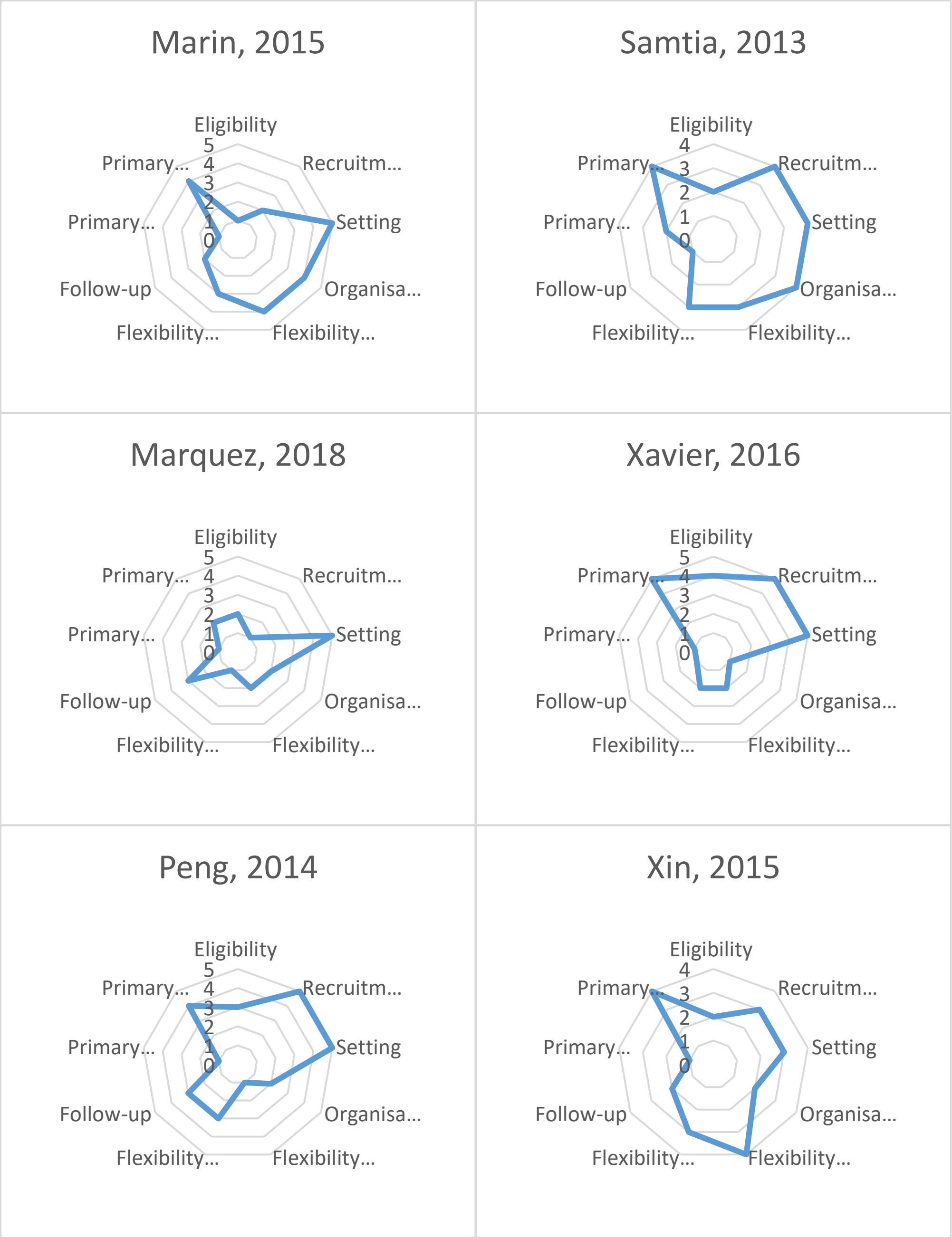
Marin, 2015
Eligibility
Recruitm...
Setting
Organisa...
Flexibility...
Flexibility...
Follow-up
Primary...
Primary...
0
1
2
3
4
5
Samtia, 2013
Eligibility
Recruitm...
Setting
Organisa...
Flexibility...
Flexibility...
Follow-up
Primary...
Primary...
0
1
2
3
4
Marquez, 2018
Eligibility
Recruitm...
Setting
Organisa...
Flexibility...
Flexibility...
Follow-up
Primary...
Primary...
0
1
2
3
4
5
Xavier, 2016
Eligibility
Recruitm...
Setting
Organisa...
Flexibility...
Flexibility...
Follow-up
Primary...
Primary...
0
1
2
3
4
5
Peng, 2014
Eligibility
Recruitm...
Setting
Organisa...
Flexibility...
Flexibility...
Follow-up
Primary...
Primary...
0
1
2
3
4
5
Xin, 2015
Eligibility
Recruitm...
Setting
Organisa...
Flexibility...
Flexibility...
Follow-up
Primary...
Primary...
0
1
2
3
4

Supplementary eFigure 3. Average PRECIS-2 wheel domain scores for studies identified as pragmatic or explanatory

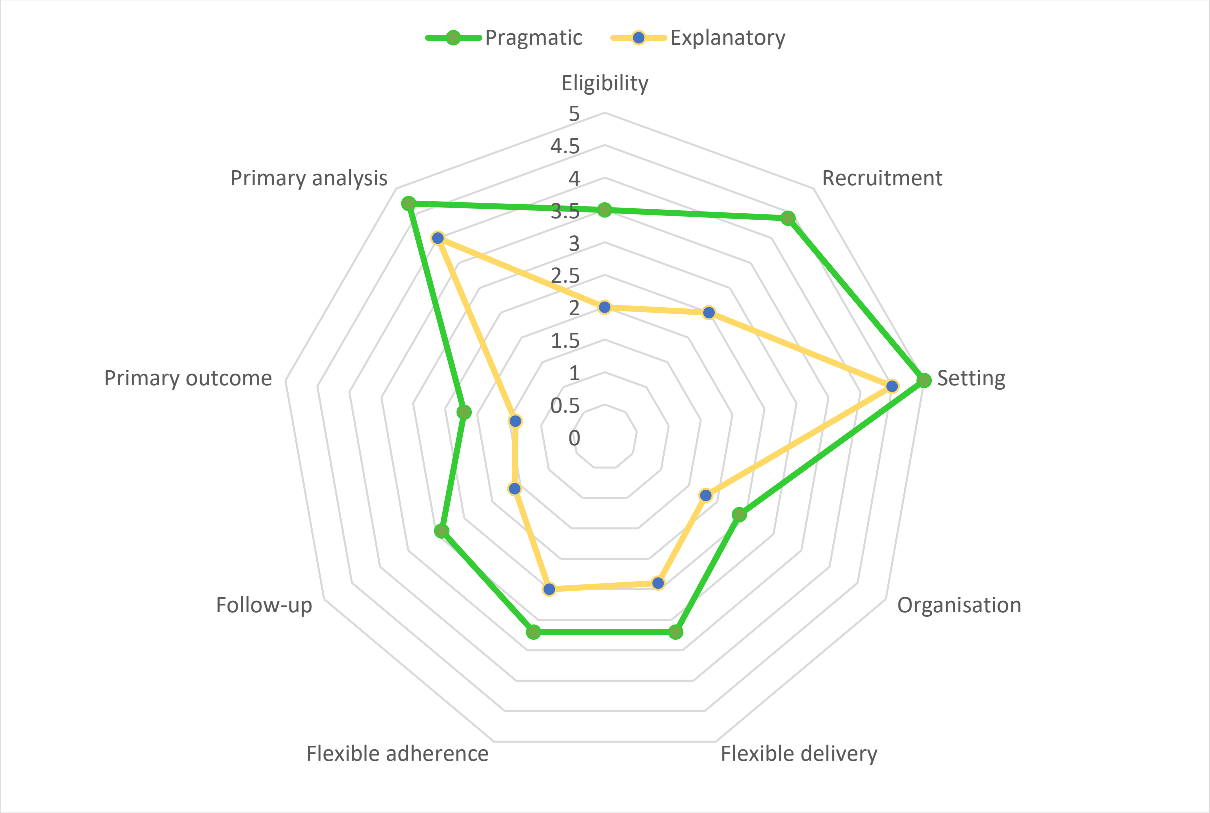

Supplementary eFigure 4 - Forest plot of pooled standardised mean differences for medication adherence, stratified by PRECIS score

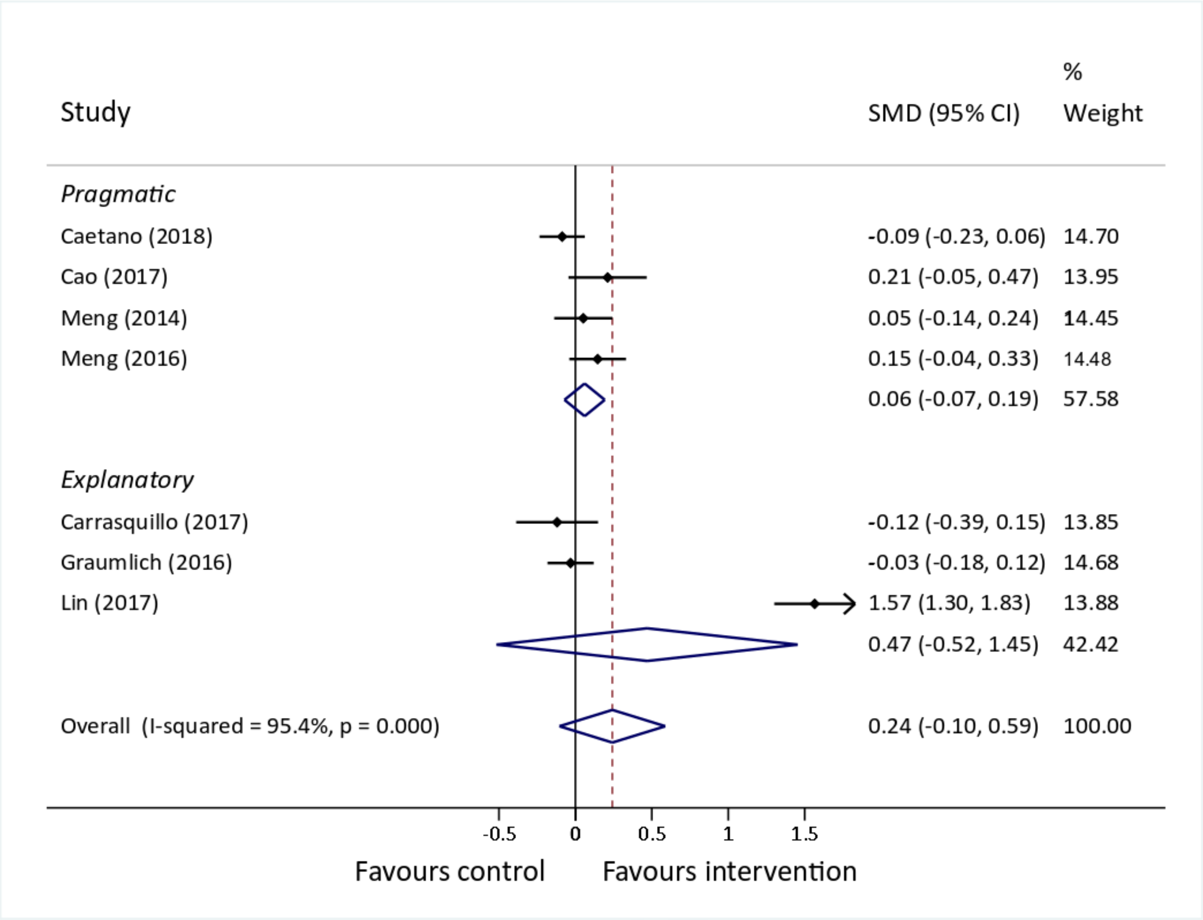

ew only

Supplementary eTable 4 - Meta-regression results showing the effects of study level covariates on medication adherence

	Coefficient (95% CI)	p-value
Odds Ratios		
Study length	0.01 (-0.01, 0.03)	0.408
Outcome (1^0 or 2^0)	0.01 (-0.39, 0.41)	0.964
Mean age	-0.02 (-0.06, 0.02)	0.317
Percent male	0.01 (-0.01, 0.02)	0.469
Disease	-0.09 (-0.42, 0.24)	0.581
Precis score	-0.02 (-0.07, 0.03)	0.367
Standardised mean differences		
Study length	0.07 (-0.04, 0.18)	0.162
Outcome (1^0 or 2^0)	-0.67 (-1.82, 0.48)	0.195
Mean age	0.06 (-0.01, 0.12)	0.071
Percent male	0.004 (-0.03, 0.04)	0.760
Disease	0.57 (-0.52, 1.65)	0.237
Precis score	-0.03 (-0.12, 0.06)	0.429

For peer review only

Supplementary eTable 5 - Results of sub-group analyses

	N studies	Effect size (95% CI)	p-value*
Odds ratios of adherence			
**Explanatory**	9	1.69 (1.24, 2.31)	0.598
**Pragmatic**	13	1.53 (1.23, 1.89)	
**Self-reported adherence measure**	15	1.63 (1.33, 1.98)	0.568
**Objective adherence measure**	7	1.47 (1.03, 2.08)	
**Multi-faceted intervention**	8	2.05 (1.62, 2.60)	0.010
**Single-faceted intervention**	14	1.28 (1.12, 1.46)	
SMD of adherence			
**Explanatory**	3	0.47 (-0.52, 1.45)	0.441
**Pragmatic**	4	0.06 (-0.07, 0.19)	
**Self-reported adherence measure**	7	*Not calculated*†	-
**Objective adherence measure**	0	*Not calculated*†	
**Multi-faceted intervention**	1	*Not calculated*†	-
**Single-faceted intervention**	6	*Not calculated*†	
*Comparing the effect size between the two sub-groups. †Pooled estimates by sub-group were not calculated where there was less than 2 studies to combine for one of the groupings			

For peer review only

Supplementary eFigure 5 - Funnel plot for the meta-analysis of standardised mean differences

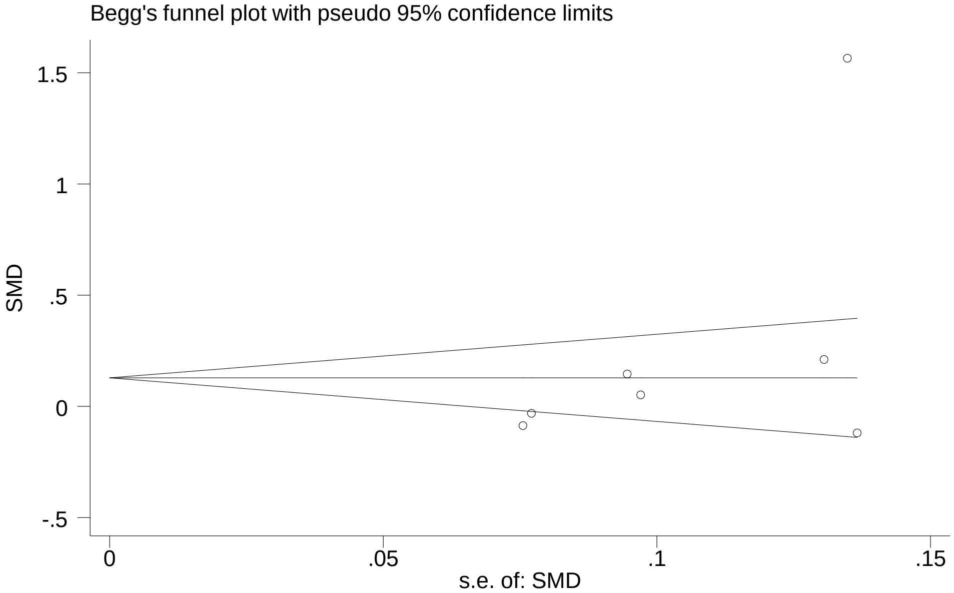

standardised mean difference of adherence

Supplementary eFigure 6 - Funnel plot for the meta-analyses of odds ratios

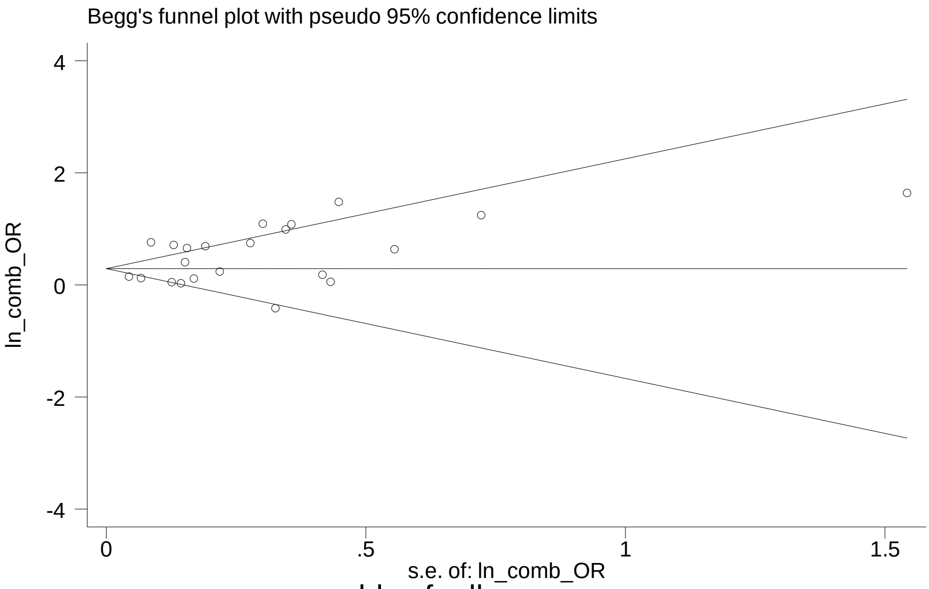

odds of adherence

For peer review only

Supplementary eTable 6 - Number and proportion of texts which report insufficient data for PRECIS-2 domain scoring

Domains (n=9)	n	%
Eligibility	0	0
Recruitment	1	2.9
Setting	1	2.9
Organisation	0	0
Flexible delivery	2	5.9
Flexible Adherence	23	67.6
Follow-up	2	5.9
Primary outcome	0	0
Primary analysis	0	0

References

1. Fuller RH, Perel P, Navarro-Ruan T, Nieuwlaat R, Haynes RB, Huffman MD. Improving medication adherence in patients with cardiovascular disease: a systematic review. Heart. 2018;104(15):1238-43.

2. Santo K, Kirkendall S, Laba T, Thakkar J, Webster R, Chalmers J, et al. Interventions to improve medication adherence in coronary disease patients: A systematic review of randomised controlled trials. Global Heart. 2016;1):e83-e4.

For peer review only

PRISMA 2009 Checklist based on draft 9

Section/topic	#	Checklist item	Reported on page #
**TITLE**			
Title	1	Identify the report as a systematic review, meta-analysis, or both.	1
**ABSTRACT**			
Structured summary	2	Provide a structured summary including, as applicable: background; objectives; data sources; study eligibility criteria, participants, and interventions; study appraisal and synthesis methods; results; limitations; conclusions and implications of key findings; systematic review registration number.	3+4
**INTRODUCTION**			
Rationale	3	Describe the rationale for the review in the context of what is already known.	5
Objectives	4	Provide an explicit statement of questions being addressed with reference to participants, interventions, comparisons, outcomes, and study design (PICOS).	5
**METHODS**			
Protocol and registration	5	Indicate if a review protocol exists, if and where it can be accessed (e.g., Web address), and, if available, provide registration information including registration number.	6
Eligibility criteria	6	Specify study characteristics (e.g., PICOS, length of follow-up) and report characteristics (e.g., years considered, language, publication status) used as criteria for eligibility, giving rationale.	6+7
Information sources	7	Describe all information sources (e.g., databases with dates of coverage, contact with study authors to identify additional studies) in the search and date last searched.	6
Search	8	Present full electronic search strategy for at least one database, including any limits used, such that it could be repeated.	26+27
Study selection	9	State the process for selecting studies (i.e., screening, eligibility, included in systematic review, and, if applicable, included in the meta-analysis).	6+7
Data collection process	10	Describe method of data extraction from reports (e.g., piloted forms, independently, in duplicate) and any processes for obtaining and confirming data from investigators.	7
Data items	11	List and define all variables for which data were sought (e.g., PICOS, funding sources) and any assumptions and simplifications made.	7
Risk of bias in individual studies	12	Describe methods used for assessing risk of bias of individual studies (including specification of whether this was done at the study or outcome level), and how this information is to be used in any data synthesis.	7

PRISMA 2009 Checklist based on draft 9

Summary measures	13	State the principal summary measures (e.g., risk ratio, difference in means).	8
Synthesis of results	14	Describe the methods of handling data and combining results of studies, if done, including measures of consistency (e.g., I^2) for each meta-analysis.	8+9

Section/topic	#	Checklist item	Reported on page #
Risk of bias across studies	15	Specify any assessment of risk of bias that may affect the cumulative evidence (e.g., publication bias, selective reporting within studies).	8+9
Additional analyses	16	Describe methods of additional analyses (e.g., sensitivity or subgroup analyses, meta-regression), if done, indicating which were pre-specified.	8+9
**RESULTS**			
Study selection	17	Give numbers of studies screened, assessed for eligibility, and included in the review, with reasons for exclusions at each stage, ideally with a flow diagram.	21
Study characteristics	18	For each study, present characteristics for which data were extracted (e.g., study size, PICOS, follow-up period) and provide the citations.	29-35
Risk of bias within studies	19	Present data on risk of bias of each study and, if available, any outcome level assessment (see item 12).	25
Results of individual studies	20	For all outcomes considered (benefits or harms), present, for each study: (a) simple summary data for each intervention group (b) effect estimates and confidence intervals, ideally with a forest plot.	10-13, 22, 23 + 42
Synthesis of results	21	Present results of each meta-analysis done, including confidence intervals and measures of consistency.	12, 22, 23 + 42
Risk of bias across studies	22	Present results of any assessment of risk of bias across studies (see Item 15).	13
Additional analysis	23	Give results of additional analyses, if done (e.g., sensitivity or subgroup analyses, meta-regression [see Item 16]).	12, 43 + 44
**DISCUSSION**			
Summary of evidence	24	Summarize the main findings including the strength of evidence for each main outcome; consider their relevance to key groups (e.g., healthcare providers,	13-15

For peer review only

PRISMA 2009 Checklist based on draft 9

		users, and policy makers).	
Limitations	25	Discuss limitations at study and outcome level (e.g., risk of bias), and at review-level (e.g., incomplete retrieval of identified research, reporting bias).	15
Conclusions	26	Provide a general interpretation of the results in the context of other evidence, and implications for future research.	16
**FUNDING**			
Funding	27	Describe sources of funding for the systematic review and other support (e.g., supply of data); role of funders for the systematic review.	16

From: Moher D, Liberati A, Tetzlaff J, Altman DG, The PRISMA Group (2009). Preferred Reporting Items for Systematic Reviews and Meta-Analyses: The PRISMA Statement. PLoS Med 6(7): e1000097. doi:10.1371/journal.pmed1000097

For more information, visit: **www.prisma-statement.org**.

For peer review only

BMJ Open

Effect of pragmatic versus explanatory interventions on medication adherence in people with cardiometabolic conditions: a systematic review and meta-analysis

Journal:	*BMJ Open*
Manuscript ID	bmjopen-2019-036575.R2
Article Type:	Original research
Date Submitted by the Author:	07-Jun-2020
Complete List of Authors:	Fitzpatrick, Claire; University of Leicester Diabetes Research Centre; University Hospitals of Leicester NHS Trust, Leicester Diabetes Centre<br>Gillies, Clare; University of Leicester Diabetes Research Centre; University Hospitals of Leicester NHS Trust, Leicester Diabetes Centre<br>Seidu, Samuel; University of Leicester Diabetes Research Centre; University Hospitals of Leicester NHS Trust, Leicester Diabetes Centre<br>Kar, Debasish; University of Sheffield Department of Oncology and Metabolism,<br>Ioannidou, Ekaterini; University of Leicester Diabetes Research Centre; University Hospitals of Leicester NHS Trust, Leicester Diabetes Centre<br>Davies, Melanie; University of Leicester Diabetes Research Centre, Diabetes Research Centre; University Hospitals of Leicester NHS Trust, Leicester Diabetes Centre<br>Patel, Prashanth; University of Leicester, Department of Cardiovascular Sciences; University Hospitals of Leicester NHS Trust, Department of Chemical Pathology and Metabolic Diseases<br>Gupta, Pankaj; University of Leicester, Department of Cardiovascular Sciences; University Hospitals of Leicester NHS Trust, Department of Chemical Pathology and Metabloic Diseases<br>Khunti, Kamlesh; University of Leicester Diabetes Research Centre; University Hospitals of Leicester NHS Trust, Leicester Diabetes Centre
**Primary Subject Heading**:	Diabetes and endocrinology
Secondary Subject Heading:	Medical education and training, Research methods
Keywords:	DIABETES & ENDOCRINOLOGY, CARDIOLOGY, Diabetes & endocrinology < INTERNAL MEDICINE

SCHOLARONE™
Manuscripts

I, the Submitting Author has the right to grant and does grant on behalf of all authors of the Work (as defined in the below author licence), an exclusive licence and/or a non-exclusive licence for contributions from authors who are: i) UK Crown employees; ii) where BMJ has agreed a CC-BY licence shall apply, and/or iii) in accordance with the terms applicable for US Federal Government officers or employees acting as part of their official duties; on a worldwide, perpetual, irrevocable, royalty-free basis to BMJ Publishing Group Ltd ("BMJ") its licensees and where the relevant Journal is co-owned by BMJ to the co-owners of the Journal, to publish the Work in this journal and any other BMJ products and to exploit all rights, as set out in our licence.

The Submitting Author accepts and understands that any supply made under these terms is made by BMJ to the Submitting Author unless you are acting as an employee on behalf of your employer or a postgraduate student of an affiliated institution which is paying any applicable article publishing charge ("APC") for Open Access articles. Where the Submitting Author wishes to make the Work available on an Open Access basis (and intends to pay the relevant APC), the terms of reuse of such Open Access shall be governed by a Creative Commons licence – details of these licences and which Creative Commons licence will apply to this Work are set out in our licence referred to above.

Other than as permitted in any relevant BMJ Author's Self Archiving Policies, I confirm this Work has not been accepted for publication elsewhere, is not being considered for publication elsewhere and does not duplicate material already published. I confirm all authors consent to publication of this Work and authorise the granting of this licence.

For peer review only

ORIGINAL RESEARCH

Effect of pragmatic versus explanatory interventions on medication adherence in people with cardiometabolic conditions: a systematic review and meta-analysis

Author Names:
Claire Fitzpatrick MSc[1,2], Clare Gillies PhD[1,2], Samuel Seidu MD[1,2], Debasish Kar[3] MSc, Ekaterini Ioannidou MSc[1,2], Melanie Davies MD[1,2], Prashanth Patel FRCPath[4,5], Pankaj Gupta FRCPath[4,5], Kamlesh Khunti PhD[1,2,6,7]*

Claire Fitzpatrick & Clare Gillies contributed equally

[1] Diabetes Research Centre, University of Leicester, Leicester, UK,

[2] Leicester Diabetes Centre, University Hospitals of Leicester NHS Trust, Leicester General Hospital, Leicester, UK

[3] University of Sheffield, Sheffield, UK

[4] Department of Cardiovascular Sciences, University of Leicester, Leicester, UK

[5] Department of Chemical Pathology and Metabolic Diseases, University Hospitals of Leicester NHS Trust, Leicester, UK

[6] NIHR CLAHRC East Midlands, Leicester, UK

[7] NIHR Applied Research Collaboration East Midlands, Leicester, UK

Claire Fitzpatrick, (0000-0003-2150-8092), Researcher, Diabetes Research Centre, University of Leicester, Leicester General Hospital, Gwendolen Road, Leicester, LE5 4PW

Claire Gillies, (0000-0002-8417-9700), Lecturer in Medical Statistics, Diabetes Research Centre, University of Leicester, Leicester General Hospital, Gwendolen Road, Leicester, LE5 4PW

Samuel Seidu, (0000-0002-8335-7018) Primary Care Research Fellow, Diabetes Research Centre, University of Leicester, Leicester General Hospital, Gwendolen Road, Leicester, LE5 4PW

Debasish Kar, (0000-0002-1524-1312), Senior Clinical Lecturer (Honorary), University of Sheffield, Western bank, Sheffield, S10 2TN

Ekaterini Ioannidou, Research Assistant, Diabetes Research Centre, University of Leicester, Leicester General Hospital, Gwendolen Road, Leicester, LE5 4PW

Melanie Davies, (0000-0002-9987-9371), Professor of Diabetes Medicine, Diabetes Research Centre, University of Leicester, Leicester General Hospital, Gwendolen Road, Leicester, LE5 4PW

Prashanth Patel, (0000-0002-8981-2691), Consultant Metabolic Physician and Honorary Senior Lecturer and Co- Director National Centre for Adherence Testing, Department of Chemical Pathology and Metabolic Diseases, University of Leicester, Leicester Royal Infirmary, Infirmary Way, Leicester, LE1 5WW

Pankaj Gupta, (0000-0001-9481-6067), Consultant, Co- Director National Centre for Adherence Testing, Department of Chemical Pathology and Metabolic Diseases, University of Leicester, Leicester Royal Infirmary, Infirmary Way, Leicester, LE1 5WW

For peer review only - http://bmjopen.bmj.com/site/about/guidelines.xhtml

Corresponding author* - Kamlesh Khunti, (0000-0003-2343-7099) Professor of Primary Care, Diabetes & Vascular Medicine, Diabetes Research Centre, University of Leicester, Leicester General Hospital, Gwendolen Road, Leicester, LE5 4PW, kk22@le.ac.uk

Manuscript word count: 4051

For peer review only

For peer review only - http://bmjopen.bmj.com/site/about/guidelines.xhtml

ABSTRACT

Objective

To synthesise findings from randomised controlled trials (RCTs) of interventions aimed at increasing medication adherence in individuals with type 2 diabetes (T2DM) and/or cardiovascular disease (CVD). And, in a novel approach, to compare the intervention effect of studies which were categorised as being more pragmatic or more explanatory using the Pragmatic-Explanatory Continuum Indicator Summary-2 (PRECIS-2) tool, to identify whether study design affects outcomes. As explanatory trials are typically held under controlled conditions, findings from such trials may not be relatable to real world clinical practice. In comparison, pragmatic trials are designed to replicate real world conditions and therefore findings are more likely to represent those found if the intervention were to be implemented in routine care.

Design

Systematic review and meta-analysis

Data sources

Ovid Medline, Ovid Embase, Web of Science and CINAHL from January 1, 2013 to December 31, 2018

Eligibility criteria for selecting studies

RCTs lasting ≥3 months (90 days), involving ≥200 patients in the analysis, with either established CVD and/or T2DM and which measured medication adherence. From 4403 citations, 103 proceeded to full text review. Studies published in any language other than English and conference abstracts were excluded.

Main outcome measure

Change in medication adherence

Results

Of 4403 records identified, 34 studies were considered eligible, of which 28, including 30,861 participants, contained comparable outcome data for inclusion in the meta-analysis. Overall interventions were associated with an increase in medication adherence (odds ratio 1.57 (95% CI: 1.33, 1.84), p<0.001, standardised mean difference 0.24 (95%CI: -0.10, 0.59) p=0.101). The effectiveness of interventions did not differ significant between studies considered pragmatic versus explanatory (p=0.598), but did differ by intervention type, with studies that included a multi-faceted rather than a single-faceted intervention having a more significant effect (p=0.010). The analysis used random effect models and used the revised Cochrane Risk of Bias Tool to assess study quality.

Conclusions

In this meta-analysis interventions were associated with a significant increase in medication adherence. Overall multi-faceted interventions which included an element of education alongside regular patient contact or follow up showed the most promise. Effectiveness of interventions between pragmatic and explanatory trials was comparable, suggesting that findings can be transferred from idealised to real word conditions.

For peer review only - http://bmjopen.bmj.com/site/about/guidelines.xhtml

For peer review only

Study Registration

PROSPERO (ID: CRD42017059460)

For peer review only

For peer review only - http://bmjopen.bmj.com/site/about/guidelines.xhtml

Strengths and limitations

- In a novel approach, this systematic review compared whether study design (pragmatic versus explanatory as defined by the Pragmatic-Explanatory Continuum Indicator Summary-2 (PRECIS-2) tool) had an impact on intervention effect.
- The study selection was undertaken independently by two researchers to ensure that all relevant studies were included as well as preventing the risk of individual biases on study selection.
- The impact of study heterogeneity was explored using subgroup analyses.
- This review provides a contemporary update to the 2014 Cochrane Review on medication adherence.
- A potential limitation of this systematic review and meta-analysis is that a small number of studies had to be excluded as they did not categorise diabetes type.

For peer review only

For peer review only - http://bmjopen.bmj.com/site/about/guidelines.xhtml

Introduction

Prevalence of type 2 diabetes (T2DM) and cardiovascular diseases (CVDs) are increasing rapidly.[1] They have been identified as two of the most common cardiometabolic morbidities associated with multi-morbidity,[1] and are two of the leading causes of death worldwide.[2] Most people diagnosed with these conditions will likely have multi-morbidity[1] (coexistence of two or more chronic conditions), particularly those aged 65-84 years where prevalence is estimated at 65%.[3]

Management of multi-morbidity is complex, typically relying on the co-prescription of multiple drugs, which is strongly associated with medication non-adherence.[4] The World Health Organisation estimates that 50% of individuals receiving chronic treatment are non-adherent (taking less than 80%) to prescribed medications.[5] Medication non-adherence is considered the biggest cause of suboptimal clinical outcomes, accounting for approximately 57% of avoidable costs in relation to medication use.[5]

Despite the rise in cardiometabolic multi-morbidity,[6-8] interventions to increase medication adherence within this population are sparse. As such we decided to focus this review on T2DM and CVD as the most prevalent cluster cardiometabolic morbidities. Treatment non-adherence within these populations is well recognised, and has become the focus of considerable research. Whilst randomised controlled trials (RCTs) are widely accepted as a rigorous way of exploring the impact of interventions on specific health behaviour change outcomes, it has been identified that numerous components within a trials design can lead to biased interpretations of intervention effects.[9] One such bias may be the controlled nature of these trials and the impact they impose upon the cooperation of their participants, and may prelude an action which does not necessarily represent what may occur in routine clinical practice.[10] In pragmatic trials, the intervention is less strict and mimics usual practice as much as possible, thus lessening the unexpected reactions from the patients which lead to the biases.[11] As randomised controlled trials (RCTs) are generally expensive to conduct, it is important that their findings show real-world intervention effectiveness that is relevant to routine clinical practice. This study therefore aimed to not only identify interventions to increase medication adherence, but also compare the effectiveness of interventions categorised as explanatory (undertaken in idealised settings) or pragmatic (undertaken in real world settings). Described by Schwartz & Lellouch (1967),[12] explanatory trial are those which confirm a physiological or clinical hypothesis, in contrast, pragmatic trails are those which inform clinical or policy decisions by evidencing the effect that adoption would have on routine care. As treatment effects of explanatory trials may be larger than those observed in pragmatic trials, traditional meta-analytic approaches may not account for this heterogeneity resulting in biased estimated treatment effects. Whilst a handful of reviews in different research areas have now been published which retrospectively applied PRECIS-2 (The Pragmatic-Explanatory Continuum Indicator Summary-2) to see if comparing pragmatic with explanatory trials altered review findings,[13, 14] to the authors knowledge, this is the first review to incorporate PRECIS-2 scoring into the initial review process. Whilst retrospective application provides an interesting comparison, whereby the overall intervention effects can be compared to explanatory and/or pragmatic intervention effects, pre or post use of PRECIS-2, it could lead to an initial misinterpretation of findings. For example, a review containing a high number of explanatory trials may be much less applicable to routine clinical practice than one containing a greater number of pragmatic trials. Identification and comparison during the trial design stage would allow researchers to more easily identify how applicable findings would be to real world clinical practice, rather than making an assumption based on a generalised outcome. In addition, this approach removes any risk of bias as the analysts are unaware of the results of the study results. Using PRECIS-2 this review scored interventions from very explanatory to

For peer review only - http://bmjopen.bmj.com/site/about/guidelines.xhtml

very pragmatic to explore whether the differences in characteristics between the study designs of these trials influenced intervention effectiveness estimates in a meta-analysis.

Methods

This systematic review has been registered on PROSPERO (ID: CRD42017059460) and was conducted in accordance with PRISMA guidelines.[15]

Data sources and searches

An extensive scoping search identified the great breath of research published within this topic area. It also identified a 2014 Cochrane Review on medication adherence which included studies published up to January 11, 2013. Due to factors relating to the focus of the review, changes in prescribed medication and the increasing use of mobile technology interventions in recent adherence research, we decided that this review would provide a contemporary update and therefore we refined our literature search to include studies published between January 1, 2013 to December 31, 2018. We searched Ovid Medline 1946 (Epub ahead of print, in process and other non-indexed citations), Ovid Embase 1974, Web of Science 1970 and Cumulative Index of Nursing and Allied Health Literature (EBSCO CINAHL-plus with full text). We searched “type 2 diabetes”, “cardiovascular disease”, “medication adherence” and “randomised control trial” using a combination of medical subject headings, keywords and synonyms with both English and American spellings. An example search strategy can be found in (Supplementary eTable 1).

Screening

Two investigators (CF and EI) independently screened all titles, abstracts and full text articles. Discrepancies were resolved through discussion with a third researcher (CG). Studies were assessed against five eligibility criteria to determine firstly, whether the study was an RCT; second, that patients were identified as having established CVD and/or T2DM; third, the trial measured medication adherence; fourth, study duration was ≥3 months (90 days) and fifth; the study included >200 people in the analysis. Studies published in any language other than English and conference abstracts were excluded. Additional reasons for exclusion were where articles failed to specify diabetes type or where results were combined for individuals at risk of and with established CVD.

Data extraction

Data extraction was performed by CF and checked by CG. Where available the following data were recorded: study characteristics (authors, year, country, duration, sample size); participant characteristics (age, gender, disease); methods of assessment (self-report, pharmacy records, pill count); intervention type (Supplementary eTable 2); and outcome measures of medication adherence. For trials reporting multiple follow-up, the final follow-up corresponding to study duration was used.

Risk of Bias

Risk of bias assessment was undertaken using The revised Cochrane Risk of Bias Tool for Randomized Controlled Trials.[16, 17]

PRECIS-2

Using Loudon *et al.* (2015) for guidance,[18] PRECIS-2 scoring to explore how pragmatic/explanatory different components of the included study designs was undertaken independently by CF and CG. Each of the nine domains was given a score from one (very explanatory “ideal study conditions”) to

For peer review only - http://bmjopen.bmj.com/site/about/guidelines.xhtml

five (very pragmatic "usual care conditions"). Results were compared and a consensus score was reached.

Some studies did not clearly explain all components making scoring difficult. In order to determine whether the treatment of unclear data affected the overall score we conducted two classification processes. For the primary method we inputted a score of 3 and then calculated an average score by adding up the scores and dividing by 9. In the second a score was not given for the missing domains and then an average was calculated based on available domain scores.

Data synthesis

Study data were reported as means and medians for continuous data and as proportions for categorical data. Twenty two studies defined a cut point to determine adherence. Twenty used a binary outcome defining individuals as adherent or non-adherent, however two categorised individuals based on a pre-specified level of low, medium, and high. Of those reporting levels of low, medium or high adherence, as has been done in previous studies,[19, 20] we combined medium and high levels and separated low level to form a binary outcome of adherence or non-adherence respectively, therefore enabling odds ratio of adherence to be calculated. In one study (Boyne, 2014), all participants in the intervention arm were adherent at follow-up, so a continuity correction of 0.5 was added to allow an odds ratio to be calculated. Where an odds ratio for medication adherence was reported in study results, this was used for the meta-analyses, rather than calculating an estimated odds ratio from the raw numbers. Where adjusted odds ratios were reported, the odds ratio adjusted for the most covariates was used in the meta-analyses.

A further six studies, plus one which was included in the previous analysis, reported adherence using a continuous scale which provided an overall group indication of adherence. For these studies, change in adherence for each study arm was calculated. As scores differed, we calculated standardised mean differences (SMD) to combine study estimates in a meta-analysis. The standardised mean difference was calculated as the mean change in medication adherence in the intervention group minus mean change in the control group, divided by the pooled standard deviation, using Cohen's method.[21]

For both meta-analysis models, funnel plots and Egger's test were carried out to assess for publication bias and random effects meta-analyses were fitted to account for heterogeneity in study design. We assessed heterogeneity between studies by calculating the Higgins I^2 statistic, with an I^2 statistic >75% considered high heterogeneity.[22] In one study (Boyne, 2014), all participants in the intervention arm were adherent at follow-up, so a continuity correction of 0.5 was added to allow an odds ratio to be calculated.

We fitted meta-regression models assessing; study length, whether adherence was a primary or secondary outcome, mean age, percent male, disease (T2DM or CVD), and PRECIS-2 score, to explore the impact of study heterogeneity on the intervention effect. Three sub-group analyses were also carried out, whereby as well as fitting a meta-regression model to compare the statistical difference between groups, separate meta-analyses were also run for each subgroup to enable the pooled estimate for each sub-group to be calculated, and hence a more explicit comparison to be made. The first compared intervention effects of studies that included a self-reported measure of adherence, to those with an objective adherence measure. The second compared pragmatic with explanatory studies (pragmatic studies were those with an average PRECIS-2 score >3 and explanatory a score of 3 or less) and the third compared outcomes of interventions which were identified as multi-faceted against those with a singular intervention component.

For peer review only - http://bmjopen.bmj.com/site/about/guidelines.xhtml

Where studies had obtained both pharmacy and self-report data, pharmacy data was used in preference in the meta-analysis. In the case of a few studies which did not report overall level of adherence and instead listed individual drug adherence, we made the pragmatic decision to monitor the adherence effect of the first drug listed by the author.

All analyses were performed using Stata 15 with the METAN, METAREG and METABIAS commands. Results are reported with 95% confidence intervals (CI) with a p value <0.05 considered statistically significant. For the few studies not included within the meta-analysis, descriptive synthesis of intervention effects is discussed.

Results

Study selection

Following deduplication we screened 4403 titles and abstracts which yielded 103 potentially relevant studies. Thirty three met the inclusion criteria with an additional study[23] identified from reference list and forward citation screening (Figure 1).[23] Of the 34 studies, 28 including 30,861 participants contained comparable outcome data for inclusion in the meta-analysis.

Characteristics of included studies

An overview of the characteristics of the included studies is available in Table 1. A more detailed key characteristics of included studies is available in Supplementary eTable 3. Studies were conducted in 17 countries, with one study[24] conducted across four countries. Twenty three trials were conducted in high income countries,[23, 25-46] seven in upper middle,[47-53] two in lower middle,[54, 55] one in a low income country, and one across both upper middle and high income economies[56]. The proportion of males ranged from 27.9%[31] to 94.0%[23]. Four trials did not report age[24, 26, 52, 56] and/or gender.[24, 26, 56]

Medical conditions varied across studies; 12 studies were in participants with T2DM,[25, 28-31, 34, 37, 41, 48, 53, 54, 56] 21 in participants with CVD[23, 24, 26, 27, 32, 33, 35, 36, 38-40, 42-44, 46, 47, 49-52, 55] and one in people with both.[45] Follow up ranged from 3-36 months.

Medication adherence was assessed as a primary outcome in 19 studies.[24, 25, 27-29, 33, 35, 36, 39, 42, 44, 45, 48, 50-53, 55, 56] Methods of measuring medication adherence varied between studies. Adherence was primarily assessed through self-report (n=23),[23-32, 34, 38, 40, 42, 44, 47-50, 53-56] followed by pharmacy data (n=10),[28, 35, 36, 41-43, 45, 46, 50, 53] pill count (n=2),[24, 33] and electronic pill bottle opening (n=1).[39] Three studies did not report the method of medication adherence assessment used.[37, 51, 52] Of those measuring adherence through self-report, the Morisky-Green questionnaire[57] and the 8-item Morisky Medication Adherence scale (MMAS-8)[58] (or translated versions) were the most frequently used. Four studies measured adherence using two different methods, self-report and either pharmacy or pill count data.[24, 28, 50, 53] Two of the studies combined the data to provide an overall level or adherence,[24, 28] whereas the other two reported adherence outcomes separately.[50, 53] Of the two studies reporting separate outcomes, both reported consistent findings and statistically significant improvements in adherence whether self-report or pharmacy data were used.

Interventions varied greatly between studies. We categorised and defined interventions (Supplementary eTable 2) based on their most prominent feature in to one of the following seven groups; (1) behavioural/educational [23, 26, 28-30, 33, 34, 38, 40, 41, 44, 51, 53, 54], (2) intensified patient care[42, 49] (3) collaborative care[47] (4) simplification of drug regimen[24] (5) personalised drug dispensing[56] (6)

For peer review only - http://bmjopen.bmj.com/site/about/guidelines.xhtml

telemonitoring/telemedicine[27, 37, 45] (7) multi-faceted intervention strategy.[25, 31, 32, 35, 36, 39, 43, 46, 48, 50, 52, 55]

PRECIS-2 Scoring

Details of PRECIS-2 scoring for each of the included studies can be found in Table 2. For the purpose of this study it was considered that each of the nine domains on the PRECIS-2 wheel had equal weighting. For visual clarity, PRECIS-2 results are presented as a shaded graph (Table 2) where darker shades represent more pragmatic components. We also inputted scores on the PRECIS-2 wheel (Supplementary eFigure 1, 2 & 3).

For peer review only

For peer review only - http://bmjopen.bmj.com/site/about/guidelines.xhtml

Table 1. Overview of characteristics of included studies				
First Author, year	Sample size (analysed)<br>Duration (Data collection points)	Condition	Key intervention detail	Medication adherence measure
Al Haj Mohd, 2016[25]	446 (446)<br>6 months (0 & 6 months)	T2DM	Multifaceted intervention strategy	Self-report (MMAS-8)
Barker Collo 2015[26]	386 (326)<br>12 months (0, 3, 6, 9 & 12 months)	Stroke/TIA	Behavioural/Educational	Self-report with cross check of pharmacy data
Boyne, 2014[27]	382 (382)<br>12 months (0, 3, 6, 12 months)	Heart failure	Tele-monitoring/telemedicine	Self-report (European heart failure self-care behavior scale)
Buhse, 2018[28]	279 (279)<br>6 months (0 & 6 months)	T2DM	Behavioural/Educational	Pharmacy data<br>Self-report (interview)
Caetano, 2018[29]	709 (702)<br>6 months (0 & 6 months)	T2DM	Behavioural/Educational	MAT scale
Cao, 2017[47]	236 (236)<br>90 days (0, 30 & 90 days)	Coronary heart disease	Collaborative care	Self-report (MMAS-8)
Carrasquillo, 2017[30]	300 (215)<br>12 months (0 & 12 months)	T2DM	Behavioural/Educational	Self-report (MMAS-8)
Castellano, 2014[24]	695 (594)<br>9 months (0, 1, 4, 9 months)	Myocardial infarction	Simplification of drug regimen	Pill count<br>Self-report (Morisky Green Levine Adherence Scale)
Chung, 2014[48]	241 (241)<br>12 months (0, 4, 8 12 months)	T2DM	Multifaceted intervention strategy	Self-report - MMAS-8 (Revised Malaysian version)
Crowley, 2013[31]	359 (329)<br>12 months (0, 3, 6, 9 & 12 months)	T2DM	Multifaceted intervention strategy	Self-report (Morisky Green Levine Adherence Scale)
Du, 2016[32]	979 (964)<br>36 months (0 & 36 months)	Percutaneous coronary intervention	Multifaceted intervention strategy	Self-report (Morisky Green Levine Adherence Scale)
El Touky, 2017[33]	321 (276)<br>12 months (0, 1, 3, 6, 9, & 12 months)	Acute coronary syndrome	Behavioural/Educational intervention	Pill count
Graumlich, 2016[34]	674 (674)<br>12 months (0, immediately post intervention, 3 & 6 months)	T2DM	Medication monitoring table	Self-report (PMAQ -patient medication adherence questionnaire)

For peer review only - http://bmjopen.bmj.com/site/about/guidelines.xhtml

Hedegaard, 2014[35]	211 (203)<br>12 months (0, 3, 6, 9 & 12 months)	Stroke/ TIA	Multifaceted intervention strategy	Pharmacy data - MPR
Ho, 2014[36]	253 (241)<br>12 months (0 & 12 months)	Acute coronary syndrome	Multifaceted intervention strategy	Pharmacy refill data
Jeong, 2018[37]	338 (338)<br>24 weeks (0 & 24 weeks)	T2DM	Tele-monitoring/telemedicine	Does not report
Jia, 2017[49]	669 (669)<br>36 months (0, 1, 3, 6, 12 & 36 months)	Percutaneous coronary intervention	Intensified patient care	Self-report (Morisky Green Levine Adherence Scale)
Kronish, 2014[38]	600 (600)<br>6 months (0 & 6 months)	Stroke/ TIA	Behavioural/Educational intervention	Self-report (MMAS-8)
Lin, 2017[50]	288 (288)<br>18 months (0, 6, 12 & 18 months)	Coronary artery bypass grafting	Multifaceted intervention strategy	Self-report – MARS (5 item)
Marin, 2015[56]	467 (459)<br>12 months (0 & 12 months)	T2DM	Personalised medication management	Self-report (Morisky Green Levine Adherence Scale)
Marquez, 2018[39]	726 (625)<br>18 months (0, 6 & 12 months)	Atrial Fibrillation	Multifaceted intervention strategy	MEMs
Meng, 2014[23]	471 (425)<br>12 months (Admission, discharge, 6 & 12 months)	Coronary heart disease	Behavioural/Educational intervention	Self-report – MARS-D
Meng, 2016[40]	513 (449)<br>12 months (Admission, discharge, 6 & 12 months)	Heart failure	Behavioural/Educational intervention	Self-report MARS-D
Peng, 2014[51]	3821 (3330)<br>12 months (Discharge, 6, 9 & 12 months)	Stroke/ TIA	Behavioural/Educational intervention	Does not report
Pladevall 2015[41]	1692 (1512)<br>18 months (0, 6, 12 & 18 months)	T2DM	Behavioural/Educational intervention	Pharmacy data -PDC
Rinfret, 2013[42]	300 (300)<br>12 months (0 & 12 months)	Percutaneous coronary intervention	Intensified patient care	Pharmacy refill data
Samtia,	348 (348)	T2DM	Behavioural/Educational	Self-report

For peer review only - http://bmjopen.bmj.com/site/about/guidelines.xhtml

2013[54]	5 months (0 & 5 months)			
Schou, 2014[43]	921 (920)<br>13-72 months (Every 1-3 months)	Heart failure	Multi-faceted intervention strategy	Pharmacy data - PDC
Schwalm, 2015[44]	852 (852)<br>12 months (0, 3 & 12 months)	Myocardial infarction	Behavioural/Educational	Self-report (Morisky Green Levine Adherence Scale)
Su, 2016[52]	1275 (1187)<br>12 months (0 & 12 months)	Stroke/ TIA	Multi-faceted intervention strategy	Does not report
Vollmer, 2014[45]	21752 (21752)<br>12 months (0 & 12 months)	T2DM and/or CVD	Telemonitoring/telemedicine	Pharmacy data - PDC
Volpp, 2017[46]	1509 (1503)<br>12 months (0 & 12 months)	Myocardial infarction	Multifaceted intervention strategy	Pharmacy data - PDC
Xavier, 2016[55]	805 (750)<br>12 months (0 & 12 months)	Acute coronary syndrome	Multifaceted intervention strategy	Pharmacy data -Composite medical adherence score >80%
Xin, 2015[53]	240 (227)<br>12 months (0 & 12 months)	T2DM	Behavioural/Educational	Prescription refill claims<br>Self-report (Morisky Green Levine Adherence Scale)
Abbreviations- T2DM: type 2 diabetes; TIA: Transient Ischemic Attack; MMAS-8; Morisky Medication Adherence Scale (8-item); PDC: proportion of days covered; MARS-D: Medication Adherence Report Scale (German version); MPR: Medication Possession Ratio; 0 months =baseline				

For peer review only - http://bmjopen.bmj.com/site/about/guidelines.xhtml

Table 2: Shaded graph to show PRECIS-2 scoring of included studies

First Author	Year	Eligibility	Recruitment	Setting	Organisation	Flexibility: delivery	Flexibility: Adherence	Follow-up	Primary outcome	Primary analysis	Total score	Average score	Amended Total score	Amended Average score
Al Haj	2016	4	4	5	1	2	3	2	1	5	27	3.00	24	3.00
Barker collo	2015	5	5	5	1	1	3	1	5	5	31	3.44	28	3.50
Boyne	2014	5	5	5	2	1	3	1	2	5	29	3.22	26	3.25
Buhse	2018	1	4	5	1	2	3	1	1	5	23	2.56	20	2.50
Caetano	2018	4	5	5	4	5	5	3	1	5	37	4.11	34	4.25
Cao	2017	2	4	5	1	2	4	2	5	5	30	3.33	30	3.33
Carrasquillo	2017	1	1	4	1	2	1	1	2	4	17	1.89	17	1.89
Castellano	2014	4	5	5	5	5	2	2	1	5	34	3.78	34	3.78
Chung	2014	2	5	5	4	1	5	2	1	5	30	3.33	30	3.33
Crowley	2013	1	1	5	2	2	3	2	2	4	22	2.44	19	2.38
Du	2016	5	4	5	2	2	3	2	5	5	33	3.67	30	3.75
El Touky	2017	4	5	5	4	3	1	2	1	1	26	2.89	23	2.88
Graumlich	2016	1	2	5	1	1	4	2	2	5	23	2.56	23	2.56
Hedegaard	2014	4	4	5	2	2	3	4	1	5	30	3.33	27	3.38
Ho	2014	4	4	5	2	1	3	5	1	5	30	3.33	27	3.38
Jeong	2018	4	5	5	1	4	3	2	2	4	30	3.33	27	3.38
Jia	2017	3	4	5	2	4	3	4	1	4	30	3.33	27	3.38
Kronish	2014	4	1	4	1	2	3	1	2	5	23	2.56	20	2.50
Lin	2017	1	2	4	1	2	3	1	1	5	20	2.22	17	2.13
Marin	2015	1	2	5	4	4	3	2	1	4	26	2.89	23	2.88
Marquez	2018	2	1	5	2	2	1	3	1	2	19	2.11	19	2.11
Meng	2014	4	5	5	2	4	3	2	2	4	31	3.44	28	3.50
Meng	2016	2	5	4	2	4	3	2	4	4	30	3.33	27	3.38
Peng	2014	3	5	5	2	1	3	3	1	4	27	3.00	21	3.00
Pladeval	2015	2	2	5	1	5	3	4	2	5	29	3.22	26	3.25
Rinfret	2013	4	4	5	2	4	3	4	1	5	32	3.56	29	3.63
Samtia	2013	2	4	4	4	3	3	1	2	4	27	3.00	21	3.00
Schou	2014	1	5	5	4	4	4	4	1	4	32	3.56	32	3.56
Schwalm	2015	5	5	5	4	4	3	2	1	4	33	3.67	30	3.75
Su	2016	4	5	5	2	2	3	2	1	4	28	3.11	25	3.13
Vollmer	2014	4	5	5	4	4	3	5	1	5	36	4.00	33	4.13
Volpp	2017	2	1	5	1	4	2	5	5	5	30	3.33	30	3.33
Xavier	2016	4	5	5	1	2	2	1	1	5	26	2.89	26	2.89
Xin	2015	2	3	3	2	4	3	2	1	4	24	2.67	15	2.50

Of the 34 studies, 20 studies were identified as being more pragmatic, with an average score >3 (range 3.11-4.11)[23, 24, 26, 27, 29, 32, 35-37, 40-49, 52]. Of these, most (n=18) received an average score between 3-4, demonstrating a slightly more pragmatic intention on the PRECIS-2 continuum. Two of the studies scored ≥4,[29, 45] demonstrating a greater degree of pragmatism. The majority of these studies scored poorly (score of 1) in relation to pragmatism of the primary outcome as, based on the

For peer review only - http://bmjopen.bmj.com/site/about/guidelines.xhtml

guidance by Loudon *et al.* (2015),[18] medication adherence was not considered of obvious importance from the patients perspective and was typically assessed by methods not used routinely in primary care. Eleven studies were identified as being more explanatory receiving an average score of <3 (range 1.89-2.89).[28, 30, 31, 33, 34, 38, 39, 50, 53, 55, 56] Of these, most (n=10) received an average score between 2-3 demonstrating that they favour a more explanatory intention on the continuum. Finally, three studies were given a score of 3[25, 51, 54] suggesting that these were equally pragmatic and explanatory.

The domains within which trials scored a more pragmatic rating were recruitment, setting of the intervention and the primary analysis which received average scores of 3.7, 4.8 and 4.4 respectively. All other domains appeared more explanatory with average scores of 2.2, 2.8, 2.9, 2.4 and 1.8 for the domains of organisation, flexible delivery, flexible adherence, follow-up and primary outcome respectively. The eligibility domain was the only one to receive a score of 3.

When results were compared based on the way missing data were scored, no changes to any studies overall categorisation was observed.

Meta-analysis

Effect of interventions

Data from 22 studies were pooled in a meta-analysis (Figure 2). Irrespective of intervention type or duration, interventions significantly improved medication adherence when compared to control (OR 1.57 (95% CI: 1.33, 1.84), p<0.001). For the seven studies where adherence was reported as a continuous variable, the pooled SMD was estimated as 0.24 (95% CI: -0.10, 0.59), p=0.101) (Supplementary eFigure 4), in favour of the intervention group, but was not statistically significant.

Both meta-analyses showed statistically significant heterogeneity between studies (p<0.001) with high I^2 values of 80.5% and 95.4% for the meta-analyses of the odds ratios and SMDs respectively, where the I^2 value represents the percentage of variability due to heterogeneity rather than sampling error.

We investigated the heterogeneity by conducting meta-regression analyses (Supplementary eTable 4). No statistically significant association was found between the intervention effects and study length, mean study age, percent male, disease (CVD or T2DM), whether it was a primary or secondary outcome or PRECIS-2 score. Sub-group analyses identified no differences in the intervention effect between self-reported and objective measures of adherence, and between PRECIS-2 score (Supplementary eTable 5). Sub-group analyses comparing multi versus singular-faceted interventions showed multi-faceted interventions led to a statistically significant improvement in odds of adherence (p=0.010) (Figure 3, Supplementary eTable 5). The funnel plot and Egger's test (p=0.184) showed that no indication of publication bias was present for the meta-analysis of SMDs (Supplementary eFigure 5). For the meta-analysis of odds ratios, Egger's test for publication bias was statistically significant (p=0.041), with the funnel plot asymmetry indicating a slightly uneven presence of small studies showing a favourable intervention effect (Supplementary eFigure 6).

Descriptive synthesis

Six studies did not contain comparable data for inclusion in the meta-analysis.[37, 39, 42, 43, 46, 56] Of those, four studies showed a positive intervention effect on rates of medication adherence,[37, 39, 42, 56] whilst two showed no statistically significant intervention effects.[43, 46]

For peer review only - http://bmjopen.bmj.com/site/about/guidelines.xhtml

Assessment of risk of bias

Table 3 displays trial specific risk of bias assessment. Few studies were deemed to be of fair quality with low risk of bias and none were considered to be free of bias. Twenty seven trials provided information about adequate sequence generation and 17 regarding allocation concealment. Incomplete outcome data was a concern in four trials and selective reporting was primarily deemed unclear (n=19) due to the number of trials without a published protocol. Blinding of participants and personnel was the main domain scoring poorly for risk of bias however the nature of these types of trials means it is often impractical to do so.

Table 3: revised Cochrane risk of bias of included studies

	Adequate random sequence generation	Allocation concealment	Blinding of participants	Blinding of personnel	Blinding of outcome assessors	Incomplete outcome data	Selective reporting	Other
Al Haj 2016	+	+	-	-	?	+	?	?
Barker Collo 2015	+	+	-	-	+	-	+	+
Boyne 2014	+	?	?	?	?	+	?	?
Buhse 2018	+	+	-	-	+	+	-	+
Caetano 2018	+	?	-	-	-	+	?	?
Cao 2017	+	+	-	-	+	+	?	?
Carrasquillo 2017	+	+	-	-	+	+	+	?
Castellano 2014	+	+	-	-	-	+	+	+
Chung 2014	?	?	-	-	-	+	?	?
Crowley 2013	+	+	-	-	-	+	+	?
Du 2016	+	+	-	-	?	+	?	?
El Touky 2017	?	?	?	?	?	-	?	?
Graumlich 2016	+	+	-	-	-	+	+	+
Hedegaard 2014	+	+	-	-	+	+	?	?
Ho 2014	+	+	?	?	+	+	+	+
Jeong 2018	?	?	-	-	-	+	-	?
Jia 2017	?	+	?	?	?	-	?	?
Kronish 2014	+	+	-	-	+	+	?	?
Lin 2017	+	?	?	?	+	+	?	?
Marin 2015	+	?	?	?	?	+	-	-
Marquez 2018	+	+	?	?	?	-	?	?
Meng 2014	-	-	-	-	-	+	?	?

For peer review only

Meng 2016	+	+	+	+	?	+	+	?
Peng 2014	+	?	?	?	?	?	+	?
Pladeval 2015	+	?	-	-	+	+	?	-
Rinfret 213	+	+	-	-	+	+	?	?
Samtia 2013	?	?	?	?	?	+	?	-
Schou 2014	+	+	-	-	-	+	+	-
Schwalm 2015	+	-	?	?	+	+	+	-
Su 2016	+	?	?	?	?	-	?	?
Vollmer 2014	+	?	-	-	-	+	?	?
Volpp 2017	+	?	-	-	+	+	-	?
Xavier 2016	+	?	-	-	-	+	+	?
Xin 2015	?	?	-	-	+	+	?	?
	**Key: +** low risk of bias, **-** high risk of bias, **?** unclear risk of bias							

Discussion

Medication adherence is a key component of chronic disease care yet many patients fail to follow prescribing guidelines.[5] This review identified 34 trials that evaluated the impact of interventions aimed at improving medication adherence in individuals with either T2DM and/or established CVD. Overall interventions significantly increased medication adherence.

To our knowledge this is the first review to systematically compare and synthesise medication adherence outcomes based on the PRECIS-2 classification of trials. Our review showed that interventions improve medication adherence and the PRECIS- 2 classification did not appear to affect the outcome when compared in subgroup and meta-regression analyses. This finding differs from previous research where explanatory trials have reported significantly larger effect sizes than pragmatic trials.[13, 14] This suggests that findings from these interventions are representation of real world clinical practice.

Using the PRECIS-2 tool retrospectively posed a number of challenges. Missing data in published manuscripts is a known problem when conducting systematic reviews, and posed additional problems for this review as manuscripts often did not include the level of detail required to accurately score certain domains (Supplementary eTable 6). As has been identified in previous research,[59] the most challenging domains to score were those relating to the flexibility of delivery and flexibility of participant adherence. Most studies focussed on detail the content of the intervention not its delivery and adherence. In order to define a study as having a more pragmatic or explanatory tendencies, we averaged domain scores to >3 or ≤3 respectively. Whilst considered the best method, we were aware of problems which could arise as a result of combining data in this way. Combining scores could result in two or more studies receiving the same or similar scores even though individual domain scores were very different. It also meant that each domain was given equal weighting in the overall score of pragmatism. Whilst a known limitation to the authors, no published guidance on how best to report the data could be found.

We restricted our review to articles published since 2013 to provide a contemporary update following a large Cochrane review published 2014 by Nieuwlaat et al.[60] Similarly to the 2014 review, our findings suggest that interventions were diverse in nature and the majority were complex, involving multiple different components. Of the number of "successful" interventions, multi-faceted interventions which included an element of education alongside regular patient contact showed the

For peer review only - http://bmjopen.bmj.com/site/about/guidelines.xhtml

most promise, suggesting that frequent engagement with the healthcare team may trigger behaviour change, or act as a reminder to undertake the behaviour. Whilst promising, considerations need to be made as to the ability of such interventions to be up-scaled and implemented. Multi-faceted interventions can be expensive and therefore their cost utility needs to be explored prior to such interventions becoming embedded in clinical care pathways.

As the number of people with access to mobile technologies has increased in the past decade, particularly in the over 60 age group, interventions which rely on frequent patient contact are becoming increasingly plausible and contact via either calls or SMS provide both a pragmatic and cheap alternative to face to face health care professional contact.[(61)] A systematic review by Changizi *et al.* (2017) looking into the effectiveness of mobile health in the elderly showed that it can be used effectively, and is widely accepted as a source of health literacy, particularly SMS messages due to their low requirement for technological competency.[(62)]

Our review highlighted a number of limitations to medication adherence research conducted to date. Firstly, the lack of gold standard method of measuring adherence makes it difficult to pool and compare outcomes. The most frequently used method within this review was self-report, particularly the tools developed by Morisky and colleagues.[(57, 58)] These tools are quick, easy and cost effective to administer making them ideal for use within a clinical care setting. The tools are validated and have shown moderate comparability with other indirect methods of medication adherence however their use has been associated with over estimation of true treatment adherence as they carry the potential for recall bias.[(63)]

Even amongst the studies utilising the Morisky scales methods of scoring and presenting data varied. For example, in the case of the Morisky-Green scale, some used a Likert scoring system,[(31)] some classed answers of no to all questions as indicative of good adherence,[(44)] whereas others classed good adherence as an answer of yes to all.[(53)] For the MMAS-8, studies such as Cao *et al.* (2017)[(47)] reported mean patient scores, with higher scores representing greater adherence. In contrast, Al Haj *et al.* (2016)[(25)] report the proportion of people categorised as low medium or highly adherent based on a score of <6, 6-7.9 or >8 respectively. Discrepancies in reporting make it difficult to compare outcomes and therefore there is a need for standardisation. The best method to measure medication adherence across disease populations, and the best approach to reporting said results, therefore continues to be an area requiring further exploration.

We also acknowledge that our findings may be limited by our strict inclusion criteria. All papers reviewed were written in English and published since 2013 and therefore results may not represent non-English and older research. Finally the lack of quality of included studies, particularly in relation to participant blinding and reporting bias could compromise the integrity of review findings. In addition, there was some indication of publication bias for the meta-analysis of odds ratios (p=0.041). This suggest that some smaller studies showing a negative result might not have been published, therefore the results of this meta-analysis need to be interpreted with some caution.

Conclusion

This systematic review showed that interventions had a significant effect on improving medication adherence in populations with T2DM and/or CVD. Multi-faceted interventions which included either regular patient contact or an element of education had the most significant effect. There is however a need to compare more standardised interventions and assess these using more uniform methods of measuring medication adherence to enable studies to be more realistically compared.

For peer review only - http://bmjopen.bmj.com/site/about/guidelines.xhtml

With regard to trial design, recently there has been a focus on designing trials that are pragmatic and therefore more representative of 'real life'. The findings from this review suggest that the effectiveness of interventions between pragmatic and explanatory trials was comparable, suggesting that findings can be transferred from idealised to real word conditions. There is however a need for further guidance to be developed to assist researchers in characterising and scoring studies.

For peer review only

For peer review only - http://bmjopen.bmj.com/site/about/guidelines.xhtml

Acknowledgements

Funding: The authors acknowledge support from the National Institute for Health Research Collaboration for Leadership in Applied Health Research and Care – East Midlands (NIHR CLAHRC – EM) and the Leicester Biomedical Research Centre. The views expressed are those of the authors and not necessarily those of the NHS, the NIHR or the Department of Health.

Author contributions: CF developed the study protocol, carried out the scientific literature search, screened and extracted data, undertook PRECIS-2 scoring, quality assessed studies, interpreted the results, and developed the first draft of the manuscript. CG quality assessed studies, interpreted the results, undertook data checking, PRECIS-2 scoring, performed all statistical analysis and contributed to the development of the manuscript. SS assisted with the design of the review and provided intellectual content to the drafted manuscript. DK assisted with the design of the review, applied for funding and assisted with protocol development. EI screened articles for inclusion in the review. MJD provided intellectual content to the drafted manuscript. PP and PG assisted with the design of the review. KK had the concept of the study, applied for funding, assisted with protocol development and provided intellectual oversight on the development of the manuscript. KK accepts full responsibility for the conduct of the study, has access to the data, and controlled the decision to publish. KK as the corresponding author attests that all listed authors meet authorship criteria and that no others meeting the criteria have been omitted.

The Corresponding Author has the right to grant on behalf of all authors and does grant on behalf of all authors, a worldwide licence to the Publishers and its licensees in perpetuity, in all forms, formats and media (whether known now or created in the future), to i) publish, reproduce, distribute, display and store the Contribution, ii) translate the Contribution into other languages, create adaptations, reprints, include within collections and create summaries, extracts and/or, abstracts of the Contribution, iii) create any other derivative work(s) based on the Contribution, iv) to exploit all subsidiary rights in the Contribution, v) the inclusion of electronic links from the Contribution to third party material where-ever it may be located; and, vi) licence any third party to do any or all of the above.

Competing interests: All authors have completed the Unified Competing Interest form (available on request from the corresponding author). CF, CG, SS, DK, EI, PP, PG declare: no support from any organisation for the submitted work; no financial relationships with any organisations that might have an interest in the submitted work in the previous three years; no other relationships or activities that could appear to have influenced the submitted work. MJD reports grants from Novo Nordisk, grants from Sanofi-Aventis, grants from Lilly, grants from Boehringer Ingelheim , grants from Janssen, personal fees from Novo Nordisk, personal fees from Sanofi-Aventis, personal fees from Lilly, personal fees from Merck Sharp & Dohme, personal fees from Boehringer Ingelheim, personal fees from AstraZeneca, personal fees from Janssen, personal fees from Servier, personal fees from Mitsubishi Tanabe Pharma Corporation , personal fees from Takeda Pharmaceuticals International Inc., outside the submitted work. KK reports personal fees from Amgen , personal fees from Astrazeneca, personal fees from Bayer, personal fees from NAPP, personal fees from Lilly , personal fees from Merck Sharp & Dohme , personal fees from Novartis, personal fees from Novo Nordisk , personal fees from Roche, personal fees from Berlin-Chemie AG / Menarini Group, personal fees from Sanofi-Aventis , personal fees from Servier, personal fees from Boehringer Ingelheim, grants from Pfizer , grants from Boehringer Ingelheim, grants from AstraZeneca, grants from Novartis, grants from Novo Nordisk, grants from Sanofi-Aventis, grants from Lilly, grants from Merck Sharp & Dohme, grants from Servier, outside the submitted work.

For peer review only - http://bmjopen.bmj.com/site/about/guidelines.xhtml

Ethical approval: Ethical approval for this meta-analysis is not required.

Data sharing: The datasets used or analysed during the current study are available from the corresponding author on reasonable request.

Transparency: The lead authors affirm that the manuscript is an honest, accurate, and transparent account of the study being reported; that no important aspects of the study have been omitted. The manuscript follows the PRISMA guidelines for reporting systematic reviews and meta-analyses.

Patient and public involvement: Patient and public involvement was undertaken prior to the development of the review to obtain an understanding of patient reasons for non-adherence. A number of themes were identified and included side effects of drugs and poor explanations from health care professionals about prescribed drugs. Following the completion of the review, alongside the views and opinions expressed during the PPI, the study team aim to develop a toolbox and education programme to support medication adherence. A second focus group is planned comprising of health professionals, with the objective of gathering their perspectives on how to promote the "toolbox" for general utilisation in clinical practice and how best to disseminate and mainstream the "toolbox" of interventions.

For peer review only

For peer review only - http://bmjopen.bmj.com/site/about/guidelines.xhtml

Reference List

1. Guthrie B, Payne K, Alderson P, McMurdo ME, Mercer SW. Adapting clinical guidelines to take account of multimorbidity. BMJ. 2012;345(oct04):e6341-e.
2. World Health Organisation. Global Health Estimates 2016: Deaths by Cause, Age, Sex, by Country, and by Region, 2000-2016. Geneva; 2018.
3. Barnett K, Mercer SW, Norbury M, Watt G, Wyke S, Guthrie B. Epidemiology of multimorbidity and implications for health care, research, and medical education: a cross-sectional study. Lancet. 2012;380(9836):37-43.
4. Marengoni A, Onder G. Guidelines, polypharmacy, and drug-drug interactions in patients with multimorbidity. BMJ (Online). 2015;350.
5. Sabaté E. Adherence to long-term therapies: evidence for action: World Health Organization; 2003.
6. Glynn LG. Multimorbidity: another key issue for cardiovascular medicine. Lancet. 2009;374(9699):1421-2.
7. Tinetti ME, Fried TR, Boyd CM. Designing health care for the most common chronic condition—multimorbidity. JAMA. 2012;307(23):2493-4.
8. Weiss CO, Boyd CM, Yu Q, Wolff JL, Leff B. Patterns of prevalent major chronic disease among older adults in the United States. JAMA. 2007;298(10):1158-62.
9. McCambridge J, Kypri K, Elbourne D. In randomization we trust? There are overlooked problems in experimenting with people in behavioral intervention trials. Journal of clinical epidemiology. 2014;67(3):247-53.
10. Godin G, Sheeran P, Conner M, Germain M. Asking questions changes behavior: mere measurement effects on frequency of blood donation. Health Psychology. 2008;27(2):179.
11. McCambridge J, Kypri K, Elbourne D. Research participation effects: a skeleton in the methodological cupboard. Journal of clinical epidemiology. 2014;67(8):845-9.
12. Schwartz D, Lellouch J. Explanatory and pragmatic attitudes in therapeutical trials. Journal of chronic diseases. 1967;20(8):637-48.
13. Sajobi TT, Li G, Awosoga O, Wang M, Menon BK, Hill MD, et al. A comparison of meta-analytic methods for synthesizing evidence from explanatory and pragmatic trials. Systematic reviews. 2018;7(1):19.
14. Yoong SL, Wolfenden L, Clinton-McHarg T, Waters E, Pettman TL, Steele E, et al. Exploring the pragmatic and explanatory study design on outcomes of systematic reviews of public health interventions: a case study on obesity prevention trials. Journal of Public Health. 2014;36(1):170-6.
15. Moher D, Liberati A, Tetzlaff J, Altman DG. Preferred reporting items for systematic reviews and meta-analyses: the PRISMA statement. Ann Intern Med. 2009;151(4):264-9.
16. Sterne JA, Savović J, Page MJ, Elbers RG, Blencowe NS, Boutron I, et al. RoB 2: a revised tool for assessing risk of bias in randomised trials. bmj. 2019;366.
17. Higgins JP, Altman DG, Gøtzsche PC, Jüni P, Moher D, Oxman AD, et al. The Cochrane Collaboration's tool for assessing risk of bias in randomised trials. BMJ. 2011;343:d5928.
18. Loudon K, Treweek S, Sullivan F, Donnan P, Thorpe KE, Zwarenstein M. The PRECIS-2 tool: designing trials that are fit for purpose. BMJ. 2015;350:h2147.
19. Khayyat SM, Khayyat SMS, Alhazmi RSH, Mohamed MM, Hadi MA. Predictors of medication adherence and blood pressure control among Saudi hypertensive patients attending primary care clinics: a cross-sectional study. PloS one. 2017;12(1):e0171255.
20. Krousel-Wood M, Islam T, Muntner P, Holt E, Joyce C, Morisky DE, et al. Association of depression with antihypertensive medication adherence in older adults: cross-sectional and longitudinal findings from CoSMO. Annals of Behavioral Medicine. 2010;40(3):248-57.
21. Cohen J. Statistical power analysis for the behavioral sciences: Routledge; 2013.
22. Higgins JP, Thompson SG, Deeks JJ, Altman DG. Measuring inconsistency in meta-analyses. BMJ. 2003;327(7414):557-60.

For peer review only - http://bmjopen.bmj.com/site/about/guidelines.xhtml

23. Meng K, Seekatz B, Haug G, Mosler G, Schwaab B, Worringen U, et al. Evaluation of a standardized patient education program for inpatient cardiac rehabilitation: impact on illness knowledge and self-management behaviors up to 1 year. Health education research. 2014;29(2):235-46.
24. Castellano JM, Sanz G, Penalvo JL, Bansilal S, Fernandez-Ortiz A, Alvarez L, et al. A polypill strategy to improve adherence: Results from the FOCUS Project. Journal of the American College of Cardiology. 2014;64(20):2071-82.
25. Al-Haj Mohd MM, Phung H, Sun J, Morisky DE. Improving adherence to medication in adults with diabetes in the United Arab Emirates. BMC Public Health. 2016;16(1):857.
26. Barker-Collo S, Krishnamurthi R, Witt E, Feigin V, Jones A, McPherson K, et al. Improving Adherence to Secondary Stroke Prevention Strategies Through Motivational Interviewing Randomized Controlled Trial. Stroke. 2015;46(12):3451-8.
27. Boyne JJ, Vrijhoef HJ, Spreeuwenberg M, De Weerd G, Kragten J, Gorgels AP, et al. Effects of tailored telemonitoring on heart failure patients' knowledge, self-care, self-efficacy and adherence: a randomized controlled trial. European Journal of Cardiovascular Nursing. 2014;13(3):243-52.
28. Buhse S, Kuniss N, Liethmann K, Muller UA, Lehmann T, Muhlhauser I. An informed shared decision making programme for patients with type 2 diabetes in primary care: Cluster randomised controlled trial. Diabetologia. 2017;60 (1 Supplement 1):S347.
29. Caetano I, Santiago LM, Marques M. Impact of written information on control and adherence in type 2 diabetes. Revista Da Associacao Medica Brasileira. 2018;64(2):140-7.
30. Carrasquillo O, Lebron C, Alonzo Y, Li H, Chang A, Kenya S. Effect of a Community Health Worker Intervention Among Latinos With Poorly Controlled Type 2 Diabetes The Miami Healthy Heart Initiative Randomized Clinical Trial. Jama Internal Medicine. 2017;177(7):948-54.
31. Crowley MJ, Powers BJ, Olsen MK, Grubber JM, Koropchak C, Rose CM, et al. The Cholesterol, Hypertension, And Glucose Education (CHANGE) study: results from a randomized controlled trial in African Americans with diabetes. American Heart Journal. 2013;166(1):179-86.
32. Du L, Dong P, Jia J, Lai L, Wang S, Yang X, et al. Impacts of intensive follow-up on the long-term prognosis of percutaneous coronary intervention in acute coronary syndrome patients - A single center prospective randomized controlled study in a Chinese population. European Journal of Preventive Cardiology. 2016;23(10):1077-85.
33. El-Toukhy H, Omar A, Abou Samra M. Effect of acute coronary syndrome patients' education on adherence to dual antiplatelet therapy Adherence to dual antiplatelet therapy. Journal of the Saudi Heart Association. 2017;29(4):252-8.
34. Graumlich JF, Wang H, Madison A, Wolf MS, Kaiser D, Dahal K, et al. Effects of a Patient-Provider, Collaborative, Medication-Planning Tool: A Randomized, Controlled Trial. Journal of Diabetes Research. 2016;2016:2129838.
35. Hedegaard U, Kjeldsen LJ, Pottegard A, Hallas J. A multifaceted pharmacist intervention to support medication adherence after stroke and transient ischemic attack. International Journal of Clinical Pharmacy. 2015;37 (1):187.
36. Ho PM, Lambert-Kerzner A, Carey EP, Fahdi IE, Bryson CL, Dee Melnyk S, et al. Multifaceted intervention to improve medication adherence and secondary prevention measures after acute coronary syndrome hospital discharge : A randomized clinical trial. JAMA Internal Medicine. 2014;174(2):186-93.
37. Jeong JY, Jeon JH, Bae KH, Choi YK, Park KG, Kim JG, et al. Smart Care Based on Telemonitoring and Telemedicine for Type 2 Diabetes Care: Multi-center Randomized Controlled Trial. Telemedicine and E-Health. 2018;24(8):604-13.
38. Kronish IM, Goldfinger JZ, Negron R, Fei K, Tuhrim S, Arniella G, et al. Effect of peer education on stroke prevention: The prevent recurrence of all inner-city strokes through education randomized controlled trial. Stroke. 2014;45(11):3330-6.
39. Marquez-Contreras E, Martell-Claros N, Marquez-Rivero S, Hermida-Campa E, Gracia-Diez C, Sanchez-Lopez E, et al. Strategies for improving dabigatran adherence for stroke prevention in

For peer review only - http://bmjopen.bmj.com/site/about/guidelines.xhtml

patients with non-valvular atrial fibrillation: education and drug intake reminders (FACILITA study). Current Medical Research and Opinion. 2018;34(7):1301-8.
40. Meng K, Musekamp G, Schuler M, Seekatz B, Glatz J, Karger G, et al. The impact of a self-management patient education program for patients with chronic heart failure undergoing inpatient cardiac rehabilitation. Patient Education & Counseling. 2016;99(7):1190-7.
41. Pladevall M, Divine G, Wells KE, Resnicow K, Williams LK. A Randomized Controlled Trial to Provide Adherence Information and Motivational Interviewing to Improve Diabetes and Lipid Control. Diabetes Educator. 2015;41(1):136-46.
42. Rinfret S, Rodes-Cabau J, Bagur R, Dery JP, Dorais M, Larose E, et al. Telephone contact to improve adherence to dual antiplatelet therapy after drug-eluting stent implantation. Heart. 2013;99(8):562-9.
43. Schou M, Gislason G, Videbaek L, Kober L, Tuxen C, Torp-Pedersen C, et al. Effect of extended follow-up in a specialized heart failure clinic on adherence to guideline recommended therapy: NorthStar Adherence Study. European Journal of Heart Failure. 2014;16(11):1249-55.
44. Schwalm JD, Ivers NM, Natarajan MK, Taljaard M, Rao-Melacini P, Witteman HO, et al. Cluster randomized controlled trial of Delayed Educational Reminders for Long-term Medication Adherence in ST-Elevation Myocardial Infarction (DERLA-STEMI). American Heart Journal. 2015;170(5):903-13.
45. Vollmer WM, Owen-Smith AA, Tom JO, Laws R, Ditmer DG, Smith DH, et al. Improving Adherence to Cardiovascular Disease Medications With Information Technology. American Journal of Managed Care. 2014;20:sp502-10.
46. Volpp KG, Troxel AB, Mehta SJ, Norton L, Zhu J, Lim R, et al. Effect of electronic reminders, financial incentives, and social support on outcomes after myocardial infarction the heartstrong randomized clinical trial. JAMA Internal Medicine. 2017;177(8):1093-101.
47. Cao XY, Tian L, Chen L, Jiang XL. Effects of a hospital-community partnership transitional program in patients with coronary heart disease in Chengdu, China: A randomized controlled trial. Japan Journal of Nursing Science. 2017;14(4):320-31.
48. Chung WW, Chua SS, Lai PSM, Chan SP. Effects of a pharmaceutical care model on medication adherence and glycemic control of people with type 2 diabetes. Patient Preference and Adherence. 2014;8:1185-94.
49. Jia JJ, Dong PS, Du LJ, Li ZG, Lai LH, Yang XM, et al. Impact of Physician-Coordinated Intensive Follow-Up on Long-Term Medical Costs in Patients with Unstable Angina Undergoing Percutaneous Coronary Intervention. Acta Cardiologica Sinica. 2017;33(2):173-81.
50. Lin CY, Yaseri M, Pakpour AH, Malm D, Brostrom A, Fridlund B, et al. Can a Multifaceted Intervention Including Motivational Interviewing Improve Medication Adherence, Quality of Life, and Mortality Rates in Older Patients Undergoing Coronary Artery Bypass Surgery? A Multicenter, Randomized Controlled Trial with 18-Month Follow-Up. Drugs & Aging. 2017;34(2):143-56.
51. Peng B, Ni J, Anderson CS, Zhu Y, Wang Y, Pu C, et al. Implementation of a structured guideline-based program for the secondary prevention of ischemic stroke in China. Stroke. 2014;45(2):515-9.
52. Su Q, Li C, Long F, Chen B, Wan Z, Wu Y, et al. Effects of a health promotion program on medication adherence to antiplatelet therapy among ischemic stroke patients in Hainan Province, China. Vascular. 2017;25(3):242-8.
53. Xin CW, Xia ZN, Jiang C, Lin MM, Li GH. Effect of pharmaceutical care on medication adherence of patients newly prescribed insulin therapy: a randomized controlled study. Patient Preference and Adherence. 2015;9:797-802.
54. Samtia AM, Rasool MF, Ranjha NM, Usman F, Javed I. A multifactorial intervention to enhance adherence to medications and disease-related knowledge in type 2 diabetic patients in Southern Punjab, Pakistan. Tropical Journal of Pharmaceutical Research. 2013;12(5):851-6.
55. Xavier D, Gupta R, Kamath D, Sigamani A, Devereaux PJ, George N, et al. Community health worker-based intervention for adherence to drugs and lifestyle change after acute coronary

For peer review only - http://bmjopen.bmj.com/site/about/guidelines.xhtml

syndrome: a multicentre, open, randomised controlled trial. The Lancet Diabetes & Endocrinology. 2016;4(3):244-53.
56. Marin GH, Risso P, Sbatella D, Haag G. Treatment adherence by personalizing the drug dispensing for diabetic patients in social vulnerable situation. Quality in Primary Care. 2015;23(2):93-6.
57. Morisky DE, Green LW, Levine DM. Concurrent and predictive validity of a self-reported measure of medication adherence. Med Care. 1986:67-74.
58. Morisky DE, Ang A, Krousel-Wood M, Ward HJ. Predictive validity of a medication adherence measure in an outpatient setting. J Clin Hypertens. 2008;10(5):348-54.
59. Johnson KE, Neta G, Dember LM, Coronado GD, Suls J, Chambers DA, et al. Use of PRECIS ratings in the National Institutes of Health (NIH) health care systems research Collaboratory. Trials. 2016;17(1):32.
60. Nieuwlaat R, Wilczynski N, Navarro T, Hobson N, Jeffery R, Keepanasseril A, et al. Interventions for enhancing medication adherence. Cochrane Database of Syst Rev. 2014(11).
61. Zhou J, Rau P-LP, Salvendy G. Age-related difference in the use of mobile phones. Universal Access in the Information Society. 2014;13(4):401-13.
62. Changizi M, Kaveh MH. Effectiveness of the mHealth technology in improvement of healthy behaviors in an elderly population—a systematic review. Mhealth. 2017;3.
63. Stirratt MJ, Dunbar-Jacob J, Crane HM, Simoni JM, Czajkowski S, Hilliard ME, et al. Self-report measures of medication adherence behavior: recommendations on optimal use. Transl Behav Med. 2015;5(4):470-82.

For peer review only

For peer review only - http://bmjopen.bmj.com/site/about/guidelines.xhtml

Figure Legends

Figure 1 – PRISMA flow chart of included studies

Figure 2 – Forest plot of pooled odds ratios for medication adherence, stratified by PRECIS score

Figure 3 – Forest plot of odds ratios for adherence, stratified by complexity of the intervention

Supplementary eFigure 1– PRECIS-2 graphs of trials identified as pragmatic

Supplementary eFigure 2 – PRECIS-2 graphs of trials identified as explanatory

Supplementary eFigure 3. Average PRECIS-2 wheel domain scores for studies identified as pragmatic or explanatory

Supplementary eFigure 4 - Forest plot of pooled standardised mean differences for medication adherence, stratified by PRECIS score

Supplementary eFigure 5 - Funnel plot for the meta-analysis of standardised mean differences

Supplementary eFigure 6. Funnel plot for the meta-analyses of odds ratios

For peer review only

For peer review only - http://bmjopen.bmj.com/site/about/guidelines.xhtml

Figure 1- PRISMA flowchart of included studies

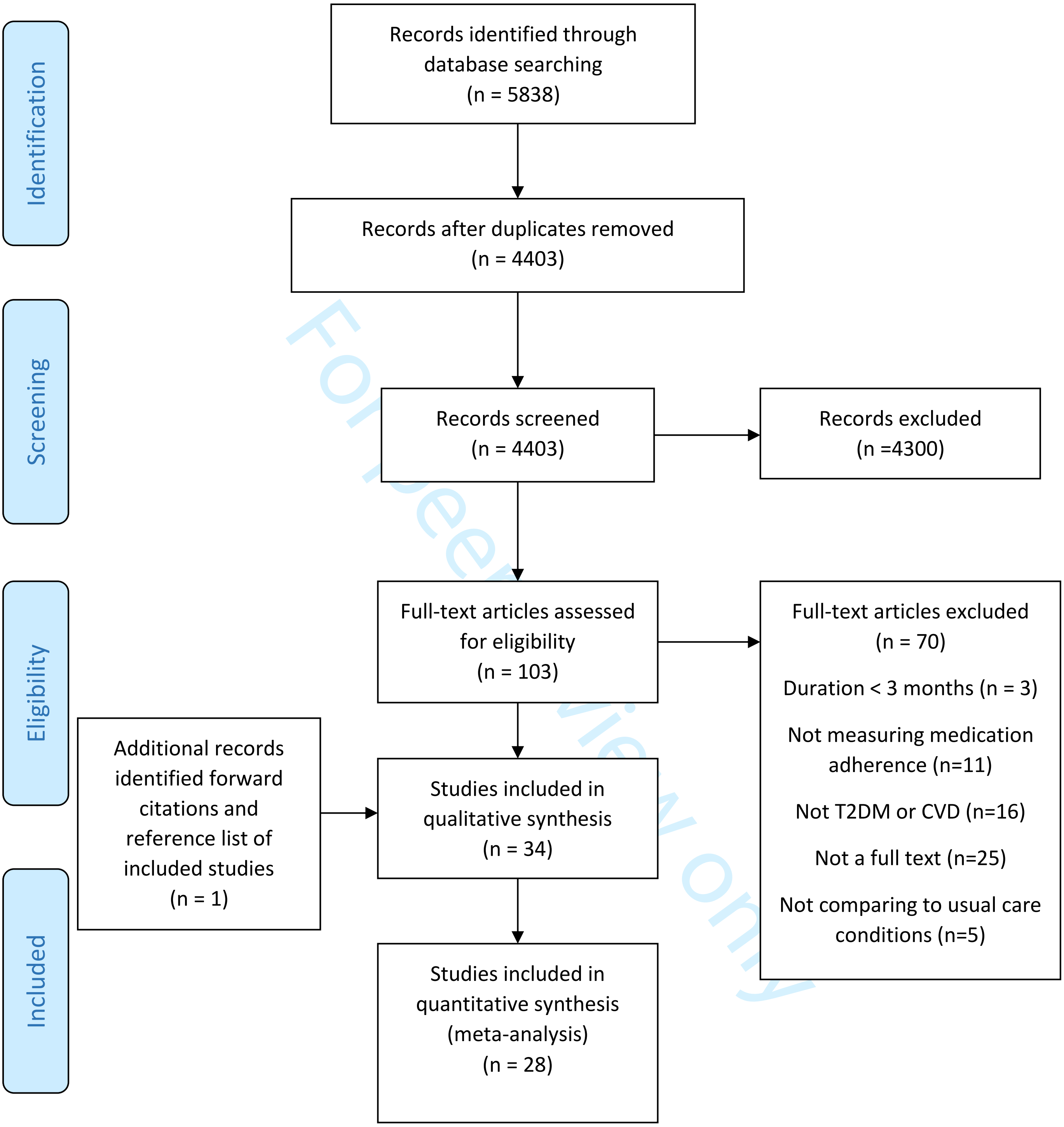

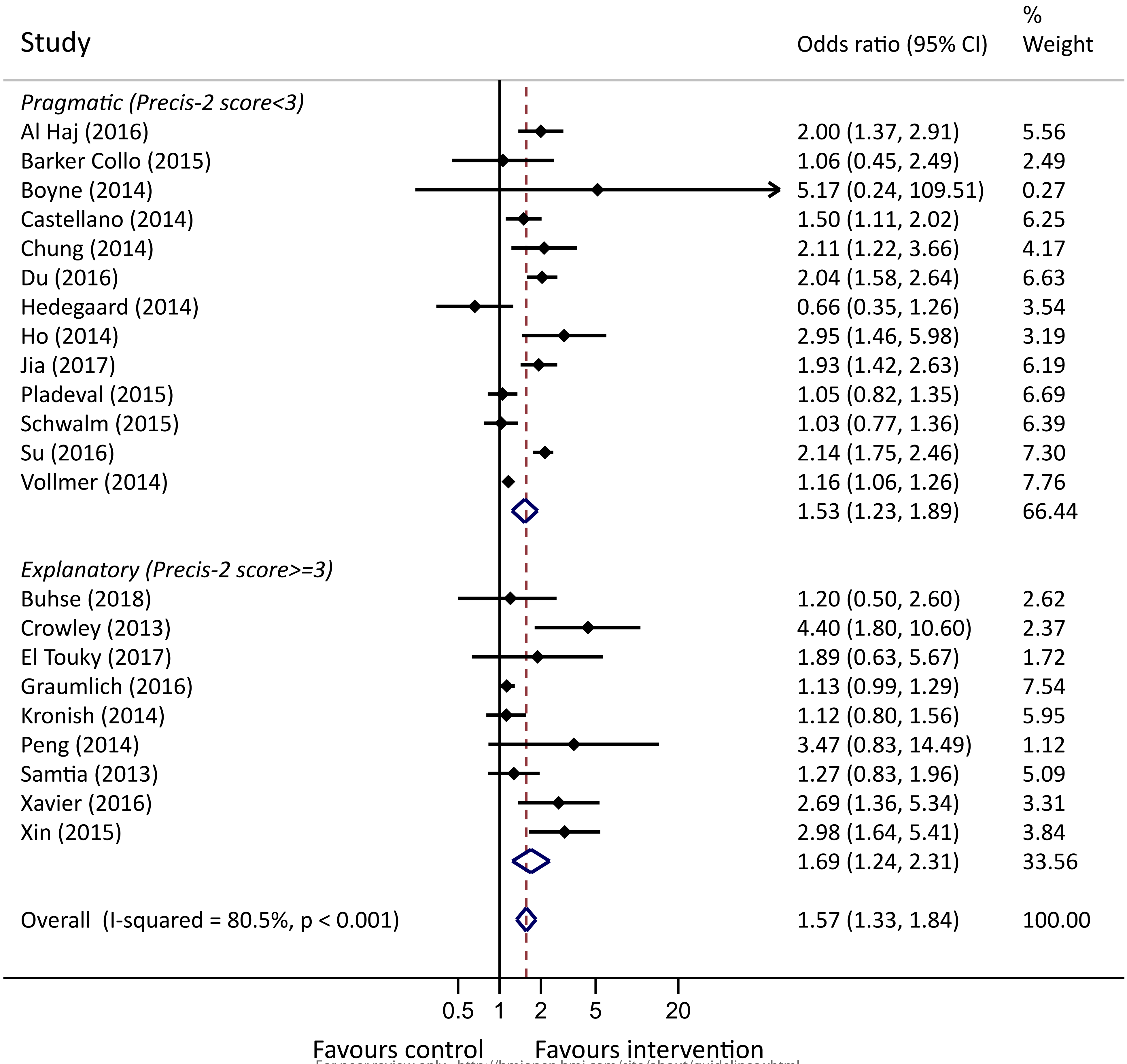
Study
Odds ratio (95% CI)
% Weight
Pragmatic (Precis-2 score<3)
Al Haj (2016) 2.00 (1.37, 2.91) 5.56
Barker Collo (2015) 1.06 (0.45, 2.49) 2.49
Boyne (2014) 5.17 (0.24, 109.51) 0.27
Castellano (2014) 1.50 (1.11, 2.02) 6.25
Chung (2014) 2.11 (1.22, 3.66) 4.17
Du (2016) 2.04 (1.58, 2.64) 6.63
Hedegaard (2014) 0.66 (0.35, 1.26) 3.54
Ho (2014) 2.95 (1.46, 5.98) 3.19
Jia (2017) 1.93 (1.42, 2.63) 6.19
Pladeval (2015) 1.05 (0.82, 1.35) 6.69
Schwalm (2015) 1.03 (0.77, 1.36) 6.39
Su (2016) 2.14 (1.75, 2.46) 7.30
Vollmer (2014) 1.16 (1.06, 1.26) 7.76
1.53 (1.23, 1.89) 66.44
Explanatory (Precis-2 score>=3)
Buhse (2018) 1.20 (0.50, 2.60) 2.62
Crowley (2013) 4.40 (1.80, 10.60) 2.37
El Touky (2017) 1.89 (0.63, 5.67) 1.72
Graumlich (2016) 1.13 (0.99, 1.29) 7.54
Kronish (2014) 1.12 (0.80, 1.56) 5.95
Peng (2014) 3.47 (0.83, 14.49) 1.12
Samtia (2013) 1.27 (0.83, 1.96) 5.09
Xavier (2016) 2.69 (1.36, 5.34) 3.31
Xin (2015) 2.98 (1.64, 5.41) 3.84
1.69 (1.24, 2.31) 33.56
Overall (I-squared = 80.5%, p < 0.001) 1.57 (1.33, 1.84) 100.00
0.5 1 2 5 20
Favours control
Favours intervention

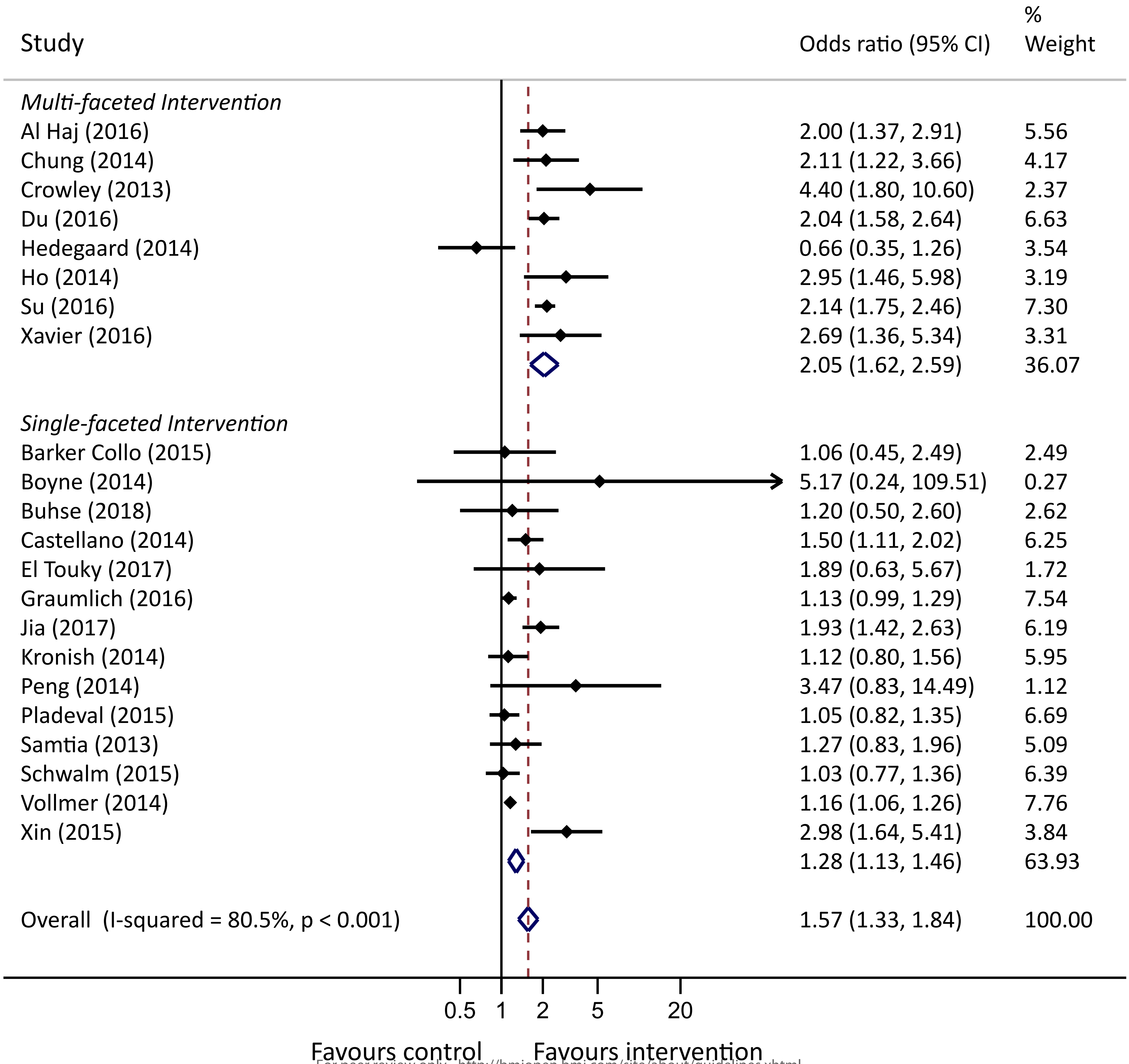

For peer review only - http://bmjopen.bmj.com/site/about/guidelines.xhtml

Online only supplement

Supplementary eTable 1 – Medline Search Strategy

Supplementary eTable 2 – Definitions of Intervention components

Supplementary eTable 3 – Characteristics of included studies

Supplementary eFigure 1– PRECIS-2 graphs of trials identified as pragmatic

Supplementary eFigure 2 – PRECIS-2 graphs of trials identified as explanatory

Supplementary eFigure 3. Average PRECIS-2 wheel domain scores for studies identified as pragmatic or explanatory

Supplementary eFigure 4 - Forest plot of pooled standardised mean differences for medication adherence, stratified by PRECIS score

Supplementary eTable 4 - Meta-regression results showing the effects of study level covariates on medication adherence

Supplementary eTable 5 - Results of sub-group analyses

Supplementary eFigure 5 - Funnel plot for the meta-analysis of standardised mean differences

Supplementary eFigure 6. Funnel plot for the meta-analyses of odds ratios

Supplementary eTable 6 - Number and proportion of texts which report insufficient data for PRECIS-2 domain scoring

For peer review only

Supplementary eTable 1 – Medline Search Strategy

1	exp patient compliance/ or patient* adher*.mp.
2	exp medication compliance/ or medication* adher*.mp.
3	((patient* or medication* or medicine* or drug* or therap* or treatment*) adj2 (adher* or concordance or non adher* or nonadher* or compliance or non compliance or noncompliance or refusal)).mp. [mp=title, abstract, original title, name of substance word, subject heading word, floating sub-heading word, keyword heading word, protocol supplementary concept word, rare disease supplementary concept word, unique identifier, synonyms]
4	treatment refusal/ or treatment* refusal.mp.
5	exp *non insulin dependent diabetes mellitus/ or Type 2 diabet*.mp.
6	T2D*.mp.
7	((non insulin or noninsulin) adj dependent diabet*).mp. [mp=title, abstract, original title, name of substance word, subject heading word, floating sub-heading word, keyword heading word, protocol supplementary concept word, rare disease supplementary concept word, unique identifier, synonyms]
8	NIDD*.mp.
9	Diabetes Mellitus/ or diabet*.mp.
10	*cardiovascular disease/ or cardiovascular diseas*.mp. or CVD.mp. [mp=title, abstract, original title, name of substance word, subject heading word, floating sub-heading word, keyword heading word, protocol supplementary concept word, rare disease supplementary concept word, unique identifier, synonyms]
11	((heart or cardiac or cardiovascular or coronary) adj2 (disease* or disorder* or failure*)).mp. [mp=title, abstract, original title, name of substance word, subject heading word, floating sub-heading word, keyword heading word, protocol supplementary concept word, rare disease supplementary concept word, unique identifier, synonyms]
12	*heart arrhythmia/ or arrhythmia*.mp.
13	*atrial fibrillation/ or atrial fibrillat*.mp.
14	*heart infarction/ or myocardial infarct*.mp.
15	heart failure.mp. or *heart failure/
16	*ischemic heart disease/ or *Myocardial Ischemia/ or myocard* isch*.mp.
17	angina.mp. or *angina pectoris/

18	*cardiomyopathy/ or cardiomyopath*.mp.
19	*coronary artery disease/ or coronary artery diseas*.mp.
20	peripheral artery diseas*.mp. or *peripheral occlusive artery disease/ or *peripheral vascular disease/
21	stroke.mp. or *cerebrovascular accident/
22	*transient ischemic attack/ or transient isch* attack.mp.
23	TIA.mp.
24	controlled clinical trial.mp. or controlled clinical trial/
25	"randomized controlled trial (topic)"/ or RCT.mp.
26	clinical trial/
27	(randomi?ed controlled adj2 trial).mp. [mp=title, abstract, original title, name of substance word, subject heading word, floating sub-heading word, keyword heading word, protocol supplementary concept word, rare disease supplementary concept word, unique identifier, synonyms]
28	1 or 2 or 3 or 4
29	5 or 6 or 7 or 8 or 9 or 10 or 11 or 12 or 13 or 14 or 15 or 16 or 17 or 18 or 19 or 20 or 21 or 22 or 23
30	24 or 25 or 26 or 27
31	28 and 29 and 30
32	limit 31 to (english language and humans and yr="2013 - 2018")

only

Supplementary eTable 2 – Definitions of Intervention components	
Behavioural/educational	Educational interventions comprising of motivational interviewing, counselling, group or individual education sessions on disease, medication and general self-management and exercise classes. In general, these studies comprised of some form of structured education and or a motivational interviewing component.
Telemonitoring	Out-patient appointments were changed for telemonitoring visits, participants were contacted at regular intervals using web-based feedback, or interactive voice recognition when patients were due or overdue their medications
Collaborative care	Enhanced communication between hospital and homecare teams
Simplification of drug regimen	Simplification of drug regimen by using a polypill
Intensified patient care	Patients were contacted more regularly than usual or drug availability and compliance was more closely monitored
Personalised drug dispensing	Weekly contact via telephone or home visits with the participant to check drug availability and adherence
Multi-faceted	Varying combinations of the aforementioned interventions with electronic pill caps and pill boxes as additional interventions
Classification of intervention types were determined based on guidance obtain from previous adherence research[1, 2]	

For peer review only

Supplementary eTable 3 **– Characteristics of included studies**

First Author, year	Country	Sample size (analysed)<br>Duration<br>Data collection points	Condition	Key intervention detail	Medication adherence measure (adherence definition or method of reporting)
Al Haj Mohd, 2016[25]	United Arab Emirates	446 (446)<br>6 months<br>0 & 6 month	T2DM	Multifaceted intervention strategy<br>• Education - 30 minute session<br>• Intensified patient care - weekly phone calls for 3 months)	Self-report - MMAS-8<br>Higher scores = better adherence
Barker Collo 2015[26]	Australia	386 (326)<br>12 months<br>0, 3, 6, 9 & 12 months	Stroke/TIA	Behavioural/Educational<br>• 4 x motivational interviewing sessions which occurred either face to face or via telephone, initial interview 60-90mins, remainder 30 minutes	Self-report – asked whether in the last 7 days they had taken all their medication as prescribed, indicate number of doses/pills missed, reason for missed doses and any side effects if noticed.<br>Cross checked self-report with pharmacy data for validity.
Boyne, 2014[27]	Netherlands	382 (382)<br>12 months<br>0, 3, 6 12 months	Heart failure	Tele-monitoring/telemedicine<br>• Replace 2 of the 4 yearly outpatient appointments with tele-monitoring	Self-report – European heart failure self-care behavior scale
Buhse, 2018[28]	Germany	279 (279)<br>6 months<br>0 & 6 months	T2DM	Behavioural/Educational<br>• Decision aid about primary prevention of MI<br>• 90 minute group education session about complication prevention<br>• Patient document with defined treatment goals	Pharmacy data<br>Self-report via telephone interview
Caetano, 2018[29]	Portugal	709 (702)<br>6 months<br>0 & 6 months	T2DM	Behavioural/Educational<br>• Patient given one of 3 educational leaflets on diabetes, diabetes treatment or physical activity	MAT scale<br>Higher scores=better adherence
Cao, 2017[47]	China	236 (236)<br>90 days	Coronary heart disease	Collaborative care<br>• Enhanced communication between hospital and home care team	Self-report - MMAS-8<br>Higher scores = better adherence

For peer review only

		0, 30 & 90 days			
Carrasquillo, 2017[30]	United States	300 (215)<br><br>12 months<br><br>0 & 12 months	T2DM	Behavioural/Educational<br>• Education - (mentoring by a community health worker approx. 4 home visits and 12 calls)<br>• Education – monthly group education sessions<br>• Behavioral – Bimonthly group exercise classes	Self-report - MMAS-8<br>Higher scores = better adherence
Castellano, 2014[24]	Argentina, Italy, Spain & Paraguay	695 (594)<br><br>9 months<br><br>0, 1, 4, 9 months	Myocardial infarction	Simplification of drug regimen<br>• Fixed dose combination polypill containing Aspirin, Simvastatin, Ramipril	Pill count<br>Self-report – Morisky-Green-Levine Medication Adherence Scale
Chung, 2014[48]	Malaysia	241 (241)<br><br>12 months<br><br>0, 4, 8 12 months	T2DM	Multifaceted intervention strategy<br>• Education – on diabetes, hypertension, hyperlipidemia and medications<br>• Medication aids – pill box and blood glucose monitoring equipment<br>• Intensified patient care – medication reviews and monthly follow up calls	Self-report - MMAS-8 (Revised Malaysian version)<br>Higher scores = better adherence
Crowley, 2013[31]	United States	359 (329)<br><br>12 months<br><br>0, 3, 6, 9 & 12 months	T2DM	Multifaceted intervention strategy<br>• Education – monthly self-management education telephone calls<br>• Personalised medication management – nurse and primary care provider contact at 3 monthly intervals to discuss medication adherence and possible medication changes	Self-report Morisky-Green-Levine Medication Adherence Scale
Du, 2016[32]	China	979 (964)<br><br>36 months<br><br>0 & 36 months	Percutaneous coronary intervention	Multifaceted intervention strategy<br>• Intensified patient care/education/personalized medication management - regular telephone follow ups at 1, 2, 3, 6, 12 & 36 months to discuss medication usage, provide health education and disease prevention strategies and schedule lab tests and treatment adjustments where necessary	Self-report Morisky-Green-Levine Medication Adherence Scale<br>Lower score = better adherence<br>(Score 0-2 = adherent)
El Touky, 2017[33]	Saudi Arabia	321 (276)		Behavioural/Educational intervention	Pill count >80% classed as adherent

		12 months<br><br>0, 1, 3, 6, 9, & 12 months	Acute coronary syndrome	• Educated patients on several aspects of coronary artery disease		
Graumlich, 2016[34]	United States	674 (674)<br><br>12 months<br><br>0, immediately following intervention, 3 & 6 months	T2DM	Medication monitoring table<br>• Multi-media tool embedded within the electronic medical record that aims to:<br>1. Promote patient knowledge<br>2. Support collaborative medication planning		Self-report -PMAQ -(patient medication adherence questionnaire)<br>Four questions were asked regarding whether the patient had missed taking a dose yesterday, the day before yesterday, 3 days ago, and over the past weekend. Patients were rated as having proper adherence if they self-reported no missed doses in this time period
Hedegaard, 2014[35]	Denmark	211 (203)<br><br>12 months<br><br>0, 3, 6, 9 & 12 months	Stroke/ TIA	Multifaceted intervention strategy<br>• Personalized medication management-medication review by pharmacist and advice on relevant drug related problems<br>• Behavioral – 3 x 30 minute patient motivational interview to support adherence and lifestyle change		Pharmacy data - MPR >0.8
Ho, 2014[36]	United States	253 (241)<br><br>12 months<br><br>0 & 12 months	Acute coronary syndrome	Multifaceted intervention strategy<br>• Personalized medication management - Medication reconciliation and tailoring by pharmacist and provision of pill box<br>• Education - Medication education<br>• Collaborative care – Pharmacist contacted patients<br>• Telemedicine – Medication reminder and refill calls		Pharmacy refill data >0.8
Jeong, 2018[37]	South Korea	338 (338)<br><br>24 weeks	T2DM	Tele-monitoring/telemedicine (Home telemonitoring composed of web based	Tele-monitoring/telemedicine Reminders (Home telemonitoring	Does not report how medication adherence was assessed.

For peer review only

		0 & 24 weeks		feedback and education with conventional management)	with remote management)	
Jia, 2017[49]	China	669 (669)<br><br>36 months<br><br>0, 1, 3, 6, 12 & 36 months	Percutaneous coronary intervention	Intensified patient care<br>• Regular phone calls with face to face appointments scheduled as necessary		Self-report - Morisky-Green-Levine Medication Adherence Scale<br>Lower score = better adherence (Score 0-2 = adherent)
Kronish, 2014[38]	United States	600 (600)<br><br>6 months<br><br>0 & 6 months	Stroke/ TIA	Behavioural/Educational intervention<br>• 6 x 90 minute weekly peer led workshops about self-management skills and medication adherence		Self-report -MMAS-8<br>Higher scores = better adherence
Lin, 2017[50]	Iran	288 (288)<br><br>18 months<br><br>0, 6, 12 & 18 months	Coronary artery bypass grafting	Multifaceted intervention strategy<br>• Educational - 3 x 60 minute weekly education sessions<br>• Behavioral – 5 x 50 minute weekly motivational interviewing<br>• Telemedicine – 4 SMS reminders sent each month, content varied on a monthly basis		Self-report – MARS (5 item)<br>Score range 5-25- Higher scores = better adherence<br>Pharmacy data
Marin, 2015[56]	Argentina	467 (459)<br><br>12 months<br><br>0 & 12 months	T2DM	Personalised medication management<br>• Weekly contact with patient to check drug availability and compliance either by phone or home visit		Self-report - Morisky-Green-Levine Medication Adherence Scale<br>Higher scores = better adherence
Marquez, 2018[39]	Spain	726 (625)<br><br>18 months<br><br>0, 6 & 12 months	Atrial Fibrillation	Multifaceted intervention strategy<br>• Education – healthcare and patient discussion of an educational booklet about medication and adherence<br>• Medication aid – medication reminder calendar		MEMs
Meng, 2014[23]	Germany	471 (425)<br><br>12 months	Coronary heart disease	Behavioural/Educational intervention<br>• 5 x 45minute patient centered self-management group education program		Self-report – MARS-D<br>Higher scores = better adherence

For peer review only

		Admission, discharge, 6 & 12 months			
Meng, 2016[40]	Germany	513 (449)<br><br>12 months<br><br>Admission, discharge, 6 & 12 months	Heart failure	Behavioural/Educational intervention<br>• 5 x 60minute patient centered self-management group education program	Self-report – MARS-D Higher scores = better adherence
Peng, 2014[51]	China	3821 (3330)<br><br>12 months<br><br>Discharge, 6, 9 & 12 months	Stroke/ TIA	Behavioural/Educational intervention<br>• Lifestyle modification - diet, exercise, smoking and risk factor control	Does not report
Pladevall *2015*[41]	United states	1692 (1512)<br><br>18 months<br><br>0, 6, 12 & 18 months	T2DM	Behavioural/Educational intervention<br>• Adherence information provided to physicians to relay to patients<br>• Above plus motivational interviewing	Pharmacy data -PDC
Rinfret, 2013[42]	Canada	300 (300)<br><br>12 months<br><br>0 & 12 months	Percutaneous coronary intervention	Intensified patient care<br>• Telephone follow-up within 7 days of DES implantation and at 1, 6, and 9 months	Pharmacy refill data
Samtia, 2013[54]	Pakistan	348 (348)<br><br>5 months<br><br>0 & 5 months	T2DM	Behavioural/Educational<br>• Continual education by pharmacists about effects of complications, medication adherence, diet, exercise, foot care, smoking cessation and HbA1c	Self-report – adherent yes/no
Schou, 2014[43]	Denmark	921 (920)<br><br>13-72 months	Heart failure	Multi-faceted intervention strategy<br>• Intensified patient care – regular drug monitoring and access to regular telephone consultations	Pharmacy data - PDC >0.8

For peer review only

		Every 1-3 months		• Education – occurred if adherence decreased during drug monitoring	
Schwalm, 2015[44]	Canada	852 (852)<br><br>12 months<br><br>0, 3 & 12 months	Myocardial infarction	Behavioural/Educational<br>• Personalised letters at 1, 5, 8 and 11 months after angiogram which stated the importance and role of each medication.<br>• Prompt to talk to physician and pharmacist about meds	• Self-report – yes/no<br>• Self-report Morisky-Green-Levine Medication Adherence Scale perfect score %
Su, 2016[52]	China	1275 (1187)<br><br>12 months<br><br>0 & 12 months	Stroke/ TIA	Multi-faceted intervention strategy<br>• Education 3 x 30 minute sessions and easy to understand manual<br>• Intensified patient care – physician contact at 1, 3 and 6 months to monitor progress	Does not report
Vollmer, 2014[45]	United States	21752 (21752)<br><br>12 months<br><br>0 & 12 months	T2DM and/or CVD	Telemonitoring/telemedicine<br>• Interactive voice recognition (IVR) calls when pts due or overdue medications<br>• Enhanced IVR as above plus letter, EMR feedback, mailed materials	Pharmacy data - PDC >0.8
Volpp, 2017[46]	United States	1509 (1503)<br><br>12 months<br><br>0 & 12 months	Myocardial infarction	Multifaceted intervention strategy<br>• Medication aids - Electronic pill bottles used in place of regular pill bottles (Glow caps)<br>• Financial incentives – Patients included in a daily lottery with the potential to win $5<br>• Medication support partner – information on patients adherence shared with a support partner whose role it was to offer support and encouragement<br>• Intensified patient care – engagement advisor and social workers to provide support on adherence issues	Pharmacy data - PDC
Xavier, 2016[55]	India	805 (750)<br><br>12 months	Acute coronary syndrome	Multifaceted intervention strategy<br>• Behavioral/educational - Mentoring by a community health worker<br>• Medication aids - medication reminder calendar and	Pharmacy data -Composite medical adherence score >80%

For peer review only

		0 & 12 months		patient dairy which contained details on drugs, how to take them and benefits.	
Xin, 2015[(53)]	China	240 (227)<br>12 months<br>0 & 12 months	T2DM	Behavioural/Educational<br>• Individualised education<br>• Group education/activities<br>• Telephone counselling	• Prescription refill claims – 80-115<br>• Morisky-Green – perfect score %

Abbreviations- T2DM: type 2 diabetes; TIA: Transient Ischemic Attack; MMAS-8; Morisky Medication Adherence Scale (8-item); PDC: proportion of days covered; MARS-D: Medication Adherence Report Scale (German version); MPR: Medication Possession Ratio; MEMs: medication event monitoring system; 0 months =baseline

For peer review only

Supplementary eFigure 1– PRECIS-2 graphs of trials identified as pragmatic

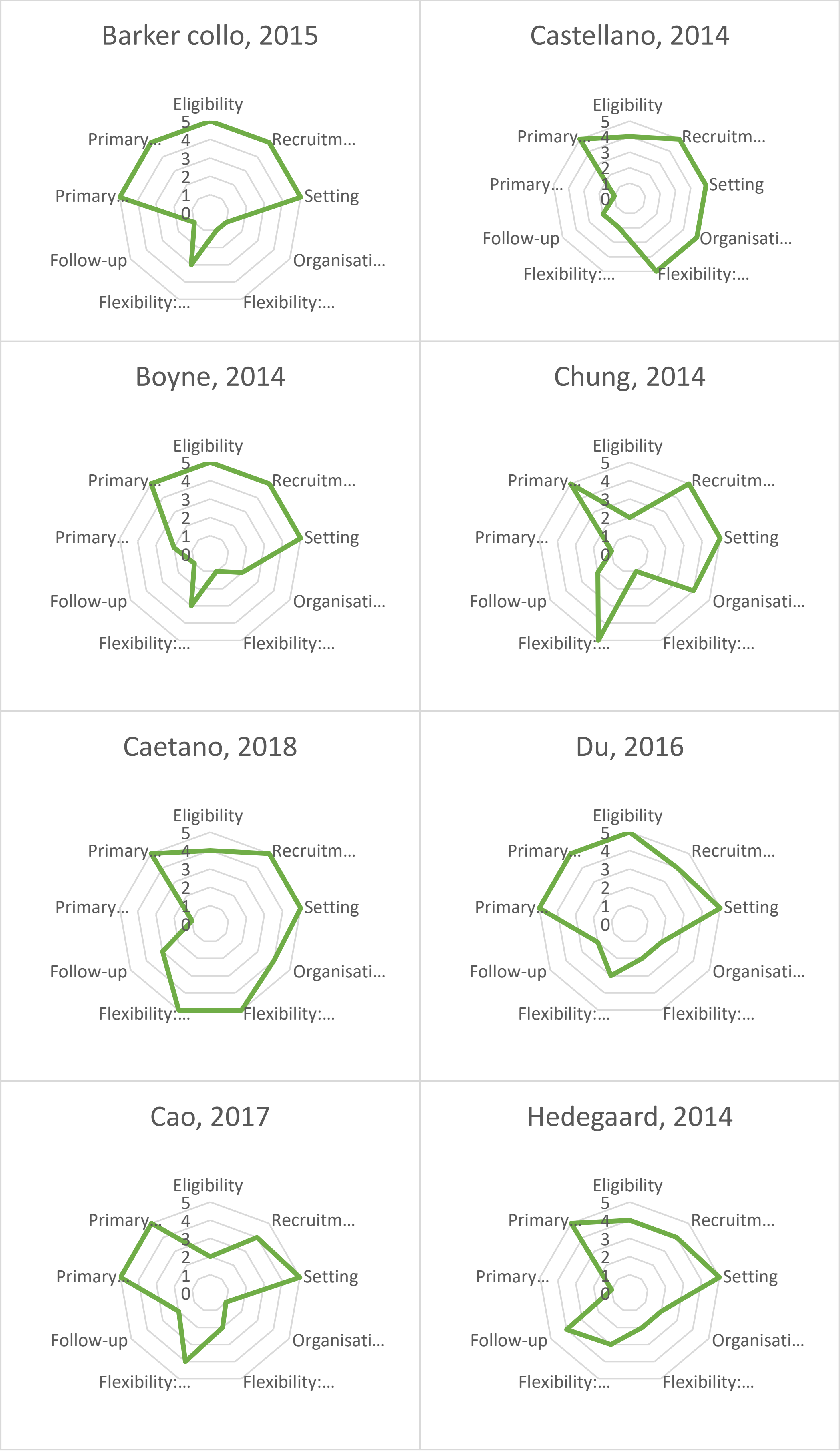

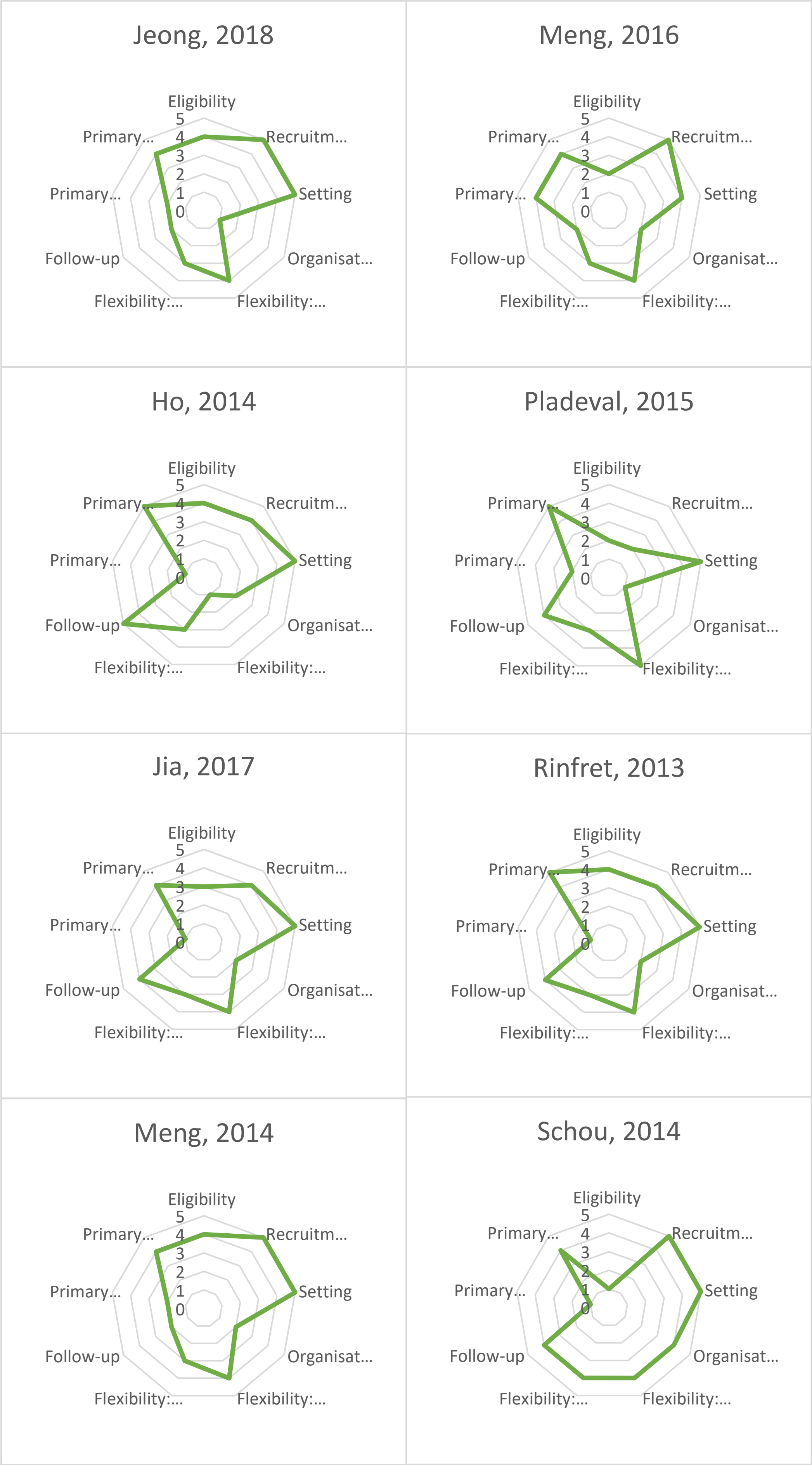
Jeong, 2018
Eligibility
Recruitm...
Setting
Organisat...
Flexibility:...
Flexibility:...
Follow-up
Primary...
Primary...
0
1
2
3
4
5
Meng, 2016
Eligibility
Recruitm...
Setting
Organisat...
Flexibility:...
Flexibility:...
Follow-up
Primary...
Primary...
0
1
2
3
4
5
Ho, 2014
Eligibility
Recruitm...
Setting
Organisat...
Flexibility:...
Flexibility:...
Follow-up
Primary...
Primary...
0
1
2
3
4
5
Pladeval, 2015
Eligibility
Recruitm...
Setting
Organisat...
Flexibility:...
Flexibility:...
Follow-up
Primary...
Primary...
0
1
2
3
4
5
Jia, 2017
Eligibility
Recruitm...
Setting
Organisat...
Flexibility:...
Flexibility:...
Follow-up
Primary...
Primary...
0
1
2
3
4
5
Rinfret, 2013
Eligibility
Recruitm...
Setting
Organisat...
Flexibility:...
Flexibility:...
Follow-up
Primary...
Primary...
0
1
2
3
4
5
Meng, 2014
Eligibility
Recruitm...
Setting
Organisat...
Flexibility:...
Flexibility:...
Follow-up
Primary...
Primary...
0
1
2
3
4
5
Schou, 2014
Eligibility
Recruitm...
Setting
Organisat...
Flexibility:...
Flexibility:...
Follow-up
Primary...
Primary...
0
1
2
3
4
5

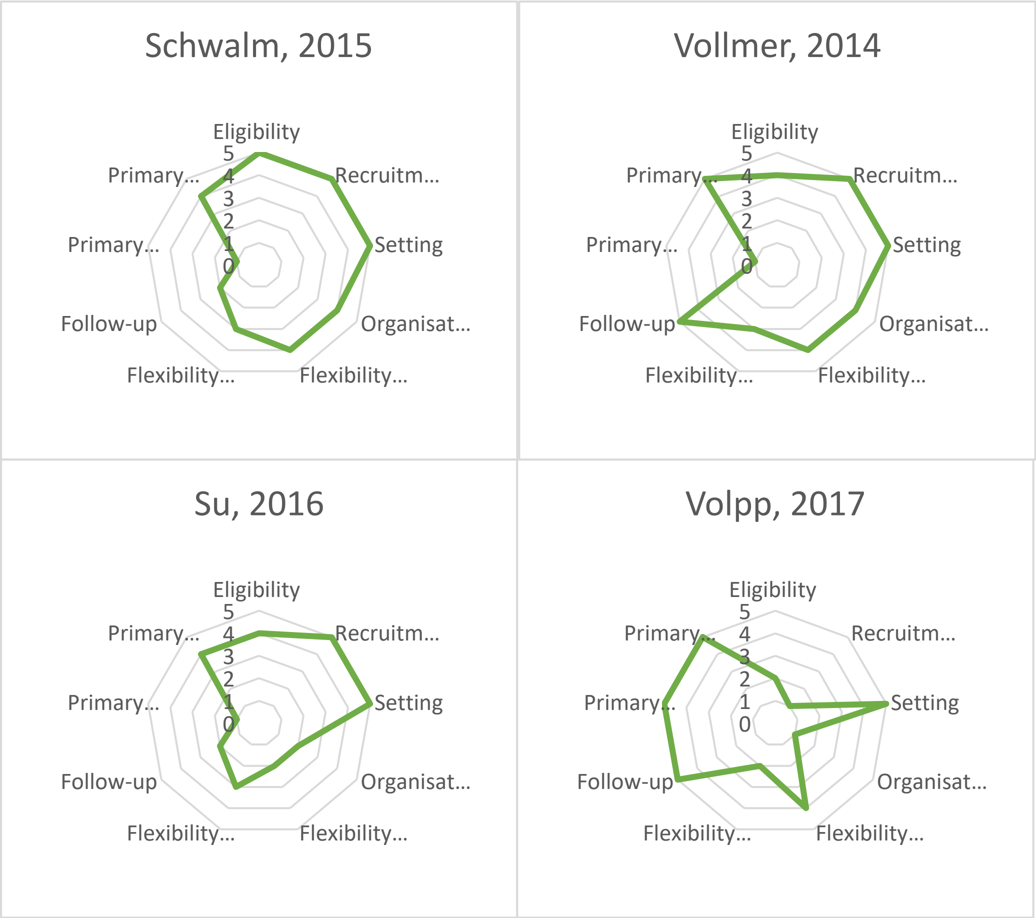

For peer review only

Supplementary eFigure 2 – PRECIS-2 graphs of trials identified as explanatory

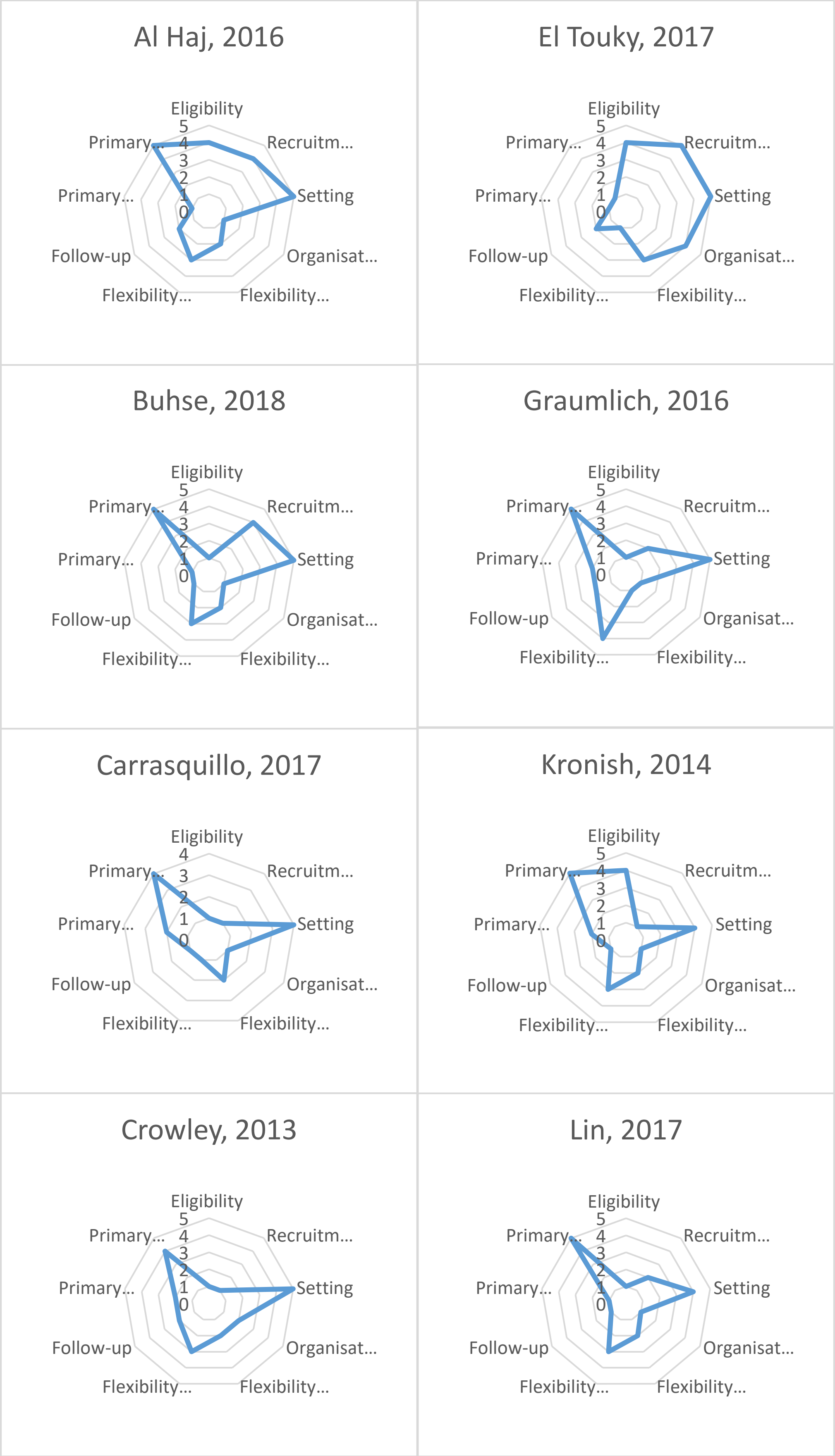

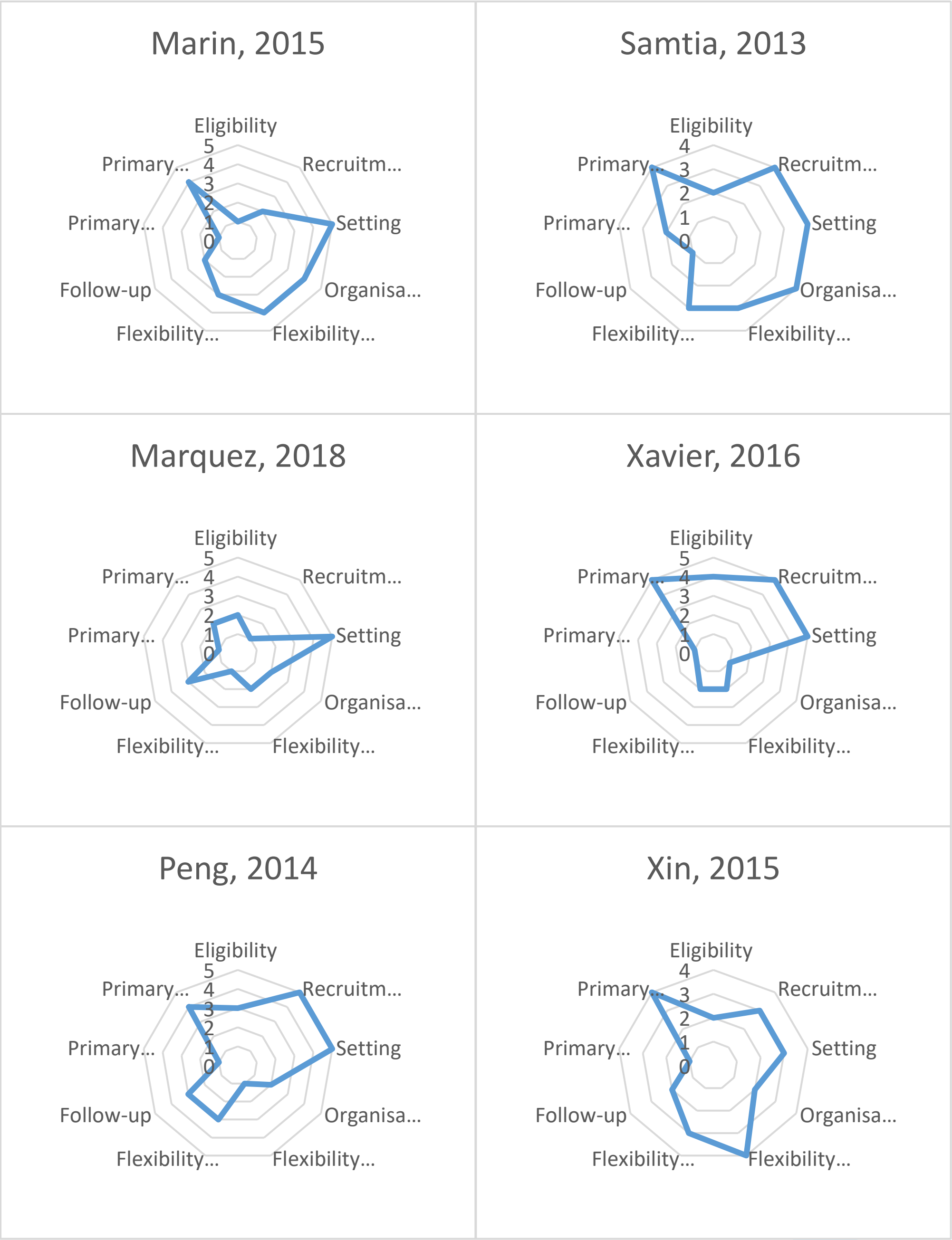
Marin, 2015
Eligibility
Recruitm...
Setting
Organisa...
Flexibility...
Flexibility...
Follow-up
Primary...
Primary...
0
1
2
3
4
5
Samtia, 2013
Eligibility
Recruitm...
Setting
Organisa...
Flexibility...
Flexibility...
Follow-up
Primary...
Primary...
0
1
2
3
4
Marquez, 2018
Eligibility
Recruitm...
Setting
Organisa...
Flexibility...
Flexibility...
Follow-up
Primary...
Primary...
0
1
2
3
4
5
Xavier, 2016
Eligibility
Recruitm...
Setting
Organisa...
Flexibility...
Flexibility...
Follow-up
Primary...
Primary...
0
1
2
3
4
5
Peng, 2014
Eligibility
Recruitm...
Setting
Organisa...
Flexibility...
Flexibility...
Follow-up
Primary...
Primary...
0
1
2
3
4
5
Xin, 2015
Eligibility
Recruitm...
Setting
Organisa...
Flexibility...
Flexibility...
Follow-up
Primary...
Primary...
0
1
2
3
4

Supplementary eFigure 3. Average PRECIS-2 wheel domain scores for studies identified as pragmatic or explanatory

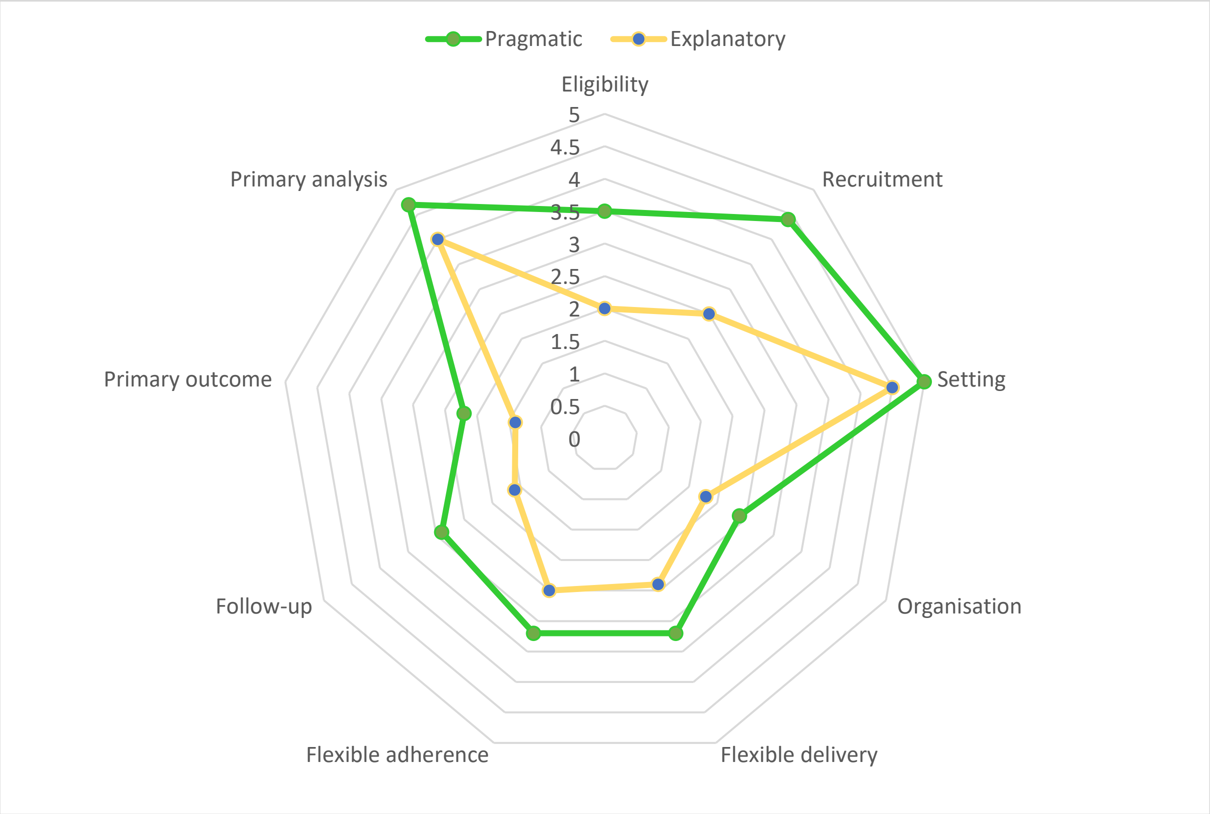

For peer review only - http://bmjopen.bmj.com/site/about/guidelines.xhtml

Supplementary eFigure 4 - Forest plot of pooled standardised mean differences for medication adherence, stratified by PRECIS score

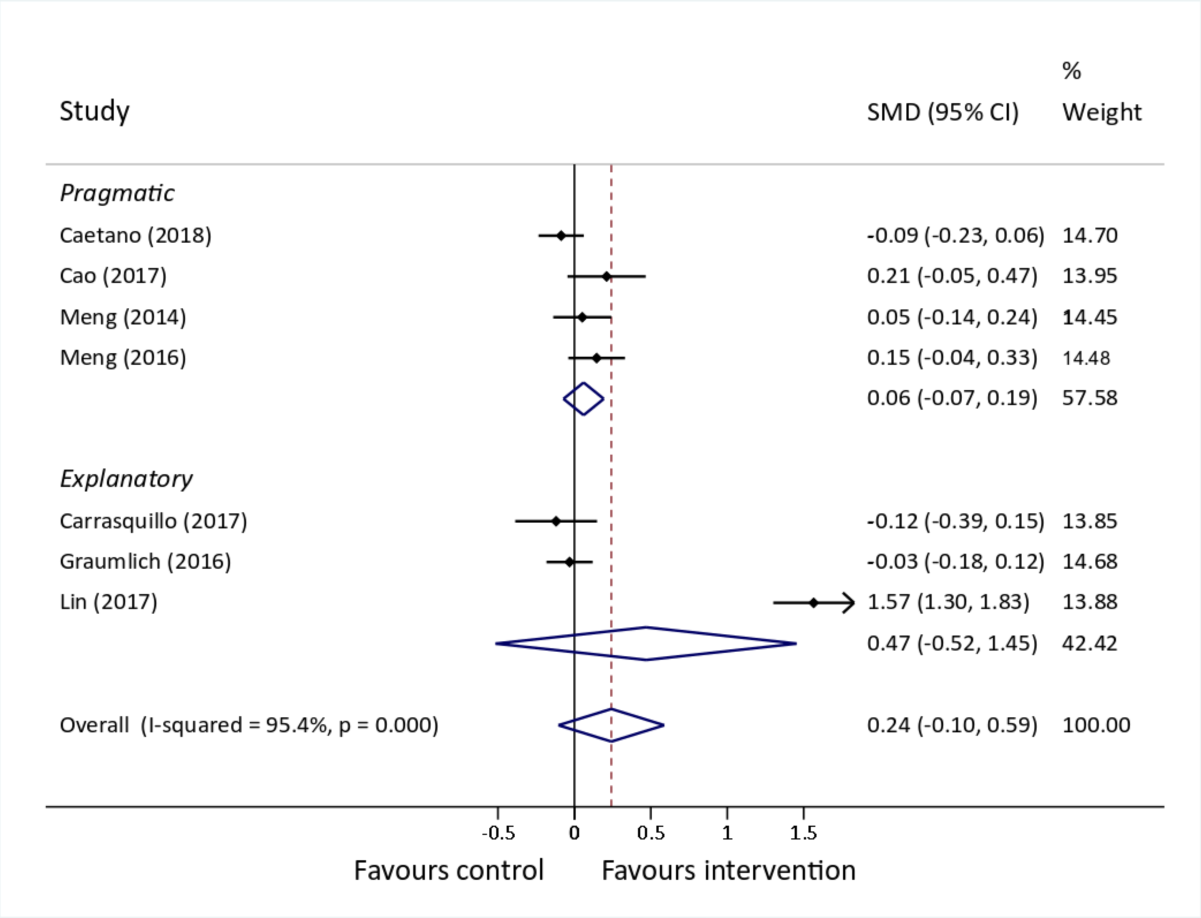

Supplementary eTable 4 - Meta-regression results showing the effects of study level covariates on medication adherence

	Coefficient (95% CI)	p-value
Odds Ratios		
Study length	0.01 (-0.01, 0.03)	0.408
Outcome (1^0 or 2^0)	0.01 (-0.39, 0.41)	0.964
Mean age	-0.02 (-0.06, 0.02)	0.317
Percent male	0.01 (-0.01, 0.02)	0.469
Disease	-0.09 (-0.42, 0.24)	0.581
Precis score	-0.02 (-0.07, 0.03)	0.367
Standardised mean differences		
Study length	0.07 (-0.04, 0.18)	0.162
Outcome (1^0 or 2^0)	-0.67 (-1.82, 0.48)	0.195
Mean age	0.06 (-0.01, 0.12)	0.071
Percent male	0.004 (-0.03, 0.04)	0.760
Disease	0.57 (-0.52, 1.65)	0.237
Precis score	-0.03 (-0.12, 0.06)	0.429

For peer review only

Supplementary eTable 5 - Results of sub-group analyses

	N studies	Effect size (95% CI)	p-value*
Odds ratios of adherence			
**Explanatory**	9	1.69 (1.24, 2.31)	0.598
**Pragmatic**	13	1.53 (1.23, 1.89)	
**Self-reported adherence measure**	15	1.63 (1.33, 1.98)	0.568
**Objective adherence measure**	7	1.47 (1.03, 2.08)	
**Multi-faceted intervention**	8	2.05 (1.62, 2.60)	0.010
**Single-faceted intervention**	14	1.28 (1.12, 1.46)	
SMD of adherence			
**Explanatory**	3	0.47 (-0.52, 1.45)	0.441
**Pragmatic**	4	0.06 (-0.07, 0.19)	
**Self-reported adherence measure**	7	*Not calculated*†	-
**Objective adherence measure**	0	*Not calculated*†	
**Multi-faceted intervention**	1	*Not calculated*†	-
**Single-faceted intervention**	6	*Not calculated*†	

*Comparing the effect size between the two sub-groups. †Pooled estimates by sub-group were not calculated where there was less than 2 studies to combine for one of the groupings

For peer review only

Supplementary eFigure 5 - Funnel plot for the meta-analysis of standardised mean differences

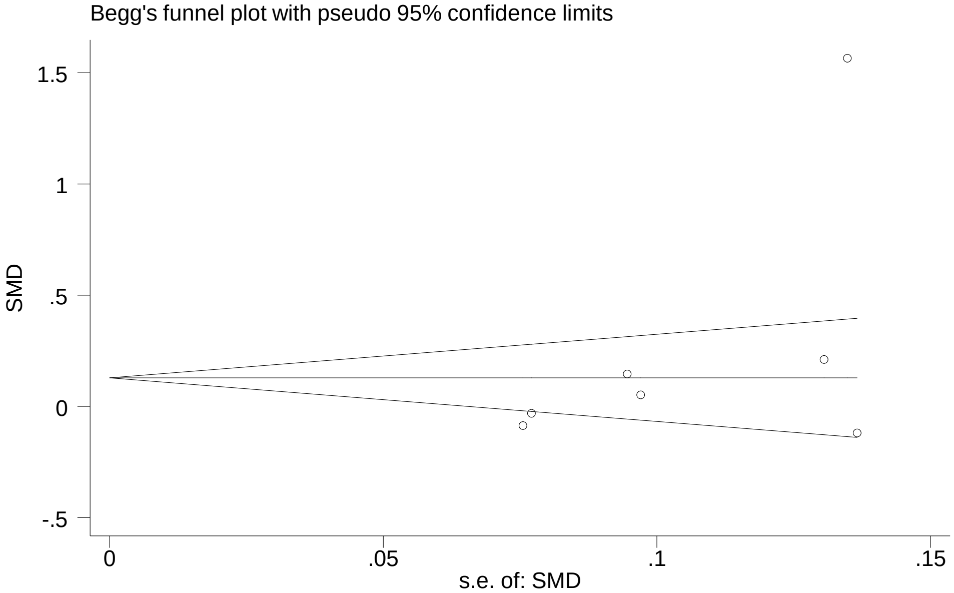

standardised mean difference of adherence

For peer review only

Supplementary eFigure 6 - Funnel plot for the meta-analyses of odds ratios

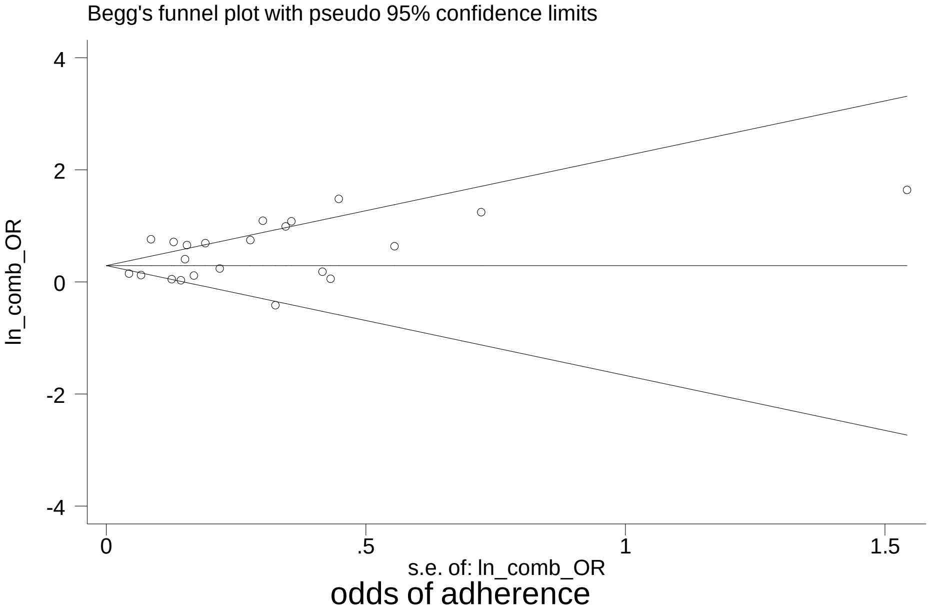

odds of adherence

For peer review only

Supplementary eTable 6 - Number and proportion of texts which report insufficient data for PRECIS-2 domain scoring

Domains (n=9)	n	%
Eligibility	0	0
Recruitment	1	2.9
Setting	1	2.9
Organisation	0	0
Flexible delivery	2	5.9
Flexible Adherence	23	67.6
Follow-up	2	5.9
Primary outcome	0	0
Primary analysis	0	0

For peer review only

PRISMA 2009 Checklist based on draft 9

Section/topic	#	Checklist item	Reported on page #
**TITLE**			
Title	1	Identify the report as a systematic review, meta-analysis, or both.	1
**ABSTRACT**			
Structured summary	2	Provide a structured summary including, as applicable: background; objectives; data sources; study eligibility criteria, participants, and interventions; study appraisal and synthesis methods; results; limitations; conclusions and implications of key findings; systematic review registration number.	3+4
**INTRODUCTION**			
Rationale	3	Describe the rationale for the review in the context of what is already known.	5
Objectives	4	Provide an explicit statement of questions being addressed with reference to participants, interventions, comparisons, outcomes, and study design (PICOS).	5
**METHODS**			
Protocol and registration	5	Indicate if a review protocol exists, if and where it can be accessed (e.g., Web address), and, if available, provide registration information including registration number.	6
Eligibility criteria	6	Specify study characteristics (e.g., PICOS, length of follow-up) and report characteristics (e.g., years considered, language, publication status) used as criteria for eligibility, giving rationale.	6+7
Information sources	7	Describe all information sources (e.g., databases with dates of coverage, contact with study authors to identify additional studies) in the search and date last searched.	6
Search	8	Present full electronic search strategy for at least one database, including any limits used, such that it could be repeated.	26+27
Study selection	9	State the process for selecting studies (i.e., screening, eligibility, included in systematic review, and, if applicable, included in the meta-analysis).	6+7
Data collection process	10	Describe method of data extraction from reports (e.g., piloted forms, independently, in duplicate) and any processes for obtaining and confirming data from investigators.	7
Data items	11	List and define all variables for which data were sought (e.g., PICOS, funding sources) and any assumptions and simplifications made.	7
Risk of bias in individual studies	12	Describe methods used for assessing risk of bias of individual studies (including specification of whether this was done at the study or outcome level), and how this information is to be used in any data synthesis.	7

For peer review only

PRISMA 2009 Checklist based on draft 9

Summary measures	13	State the principal summary measures (e.g., risk ratio, difference in means).	8
Synthesis of results	14	Describe the methods of handling data and combining results of studies, if done, including measures of consistency (e.g., I^2) for each meta-analysis.	8+9

Section/topic	#	Checklist item	Reported on page #
Risk of bias across studies	15	Specify any assessment of risk of bias that may affect the cumulative evidence (e.g., publication bias, selective reporting within studies).	8+9
Additional analyses	16	Describe methods of additional analyses (e.g., sensitivity or subgroup analyses, meta-regression), if done, indicating which were pre-specified.	8+9
**RESULTS**			
Study selection	17	Give numbers of studies screened, assessed for eligibility, and included in the review, with reasons for exclusions at each stage, ideally with a flow diagram.	21
Study characteristics	18	For each study, present characteristics for which data were extracted (e.g., study size, PICOS, follow-up period) and provide the citations.	29-35
Risk of bias within studies	19	Present data on risk of bias of each study and, if available, any outcome level assessment (see item 12).	25
Results of individual studies	20	For all outcomes considered (benefits or harms), present, for each study: (a) simple summary data for each intervention group (b) effect estimates and confidence intervals, ideally with a forest plot.	10-13, 22, 23 + 42
Synthesis of results	21	Present results of each meta-analysis done, including confidence intervals and measures of consistency.	12, 22, 23 + 42
Risk of bias across studies	22	Present results of any assessment of risk of bias across studies (see Item 15).	13
Additional analysis	23	Give results of additional analyses, if done (e.g., sensitivity or subgroup analyses, meta-regression [see Item 16]).	12, 43 + 44
**DISCUSSION**			
Summary of evidence	24	Summarize the main findings including the strength of evidence for each main outcome; consider their relevance to key groups (e.g., healthcare providers,	13-15

For peer review only

PRISMA 2009 Checklist based on draft 9

		users, and policy makers).	
Limitations	25	Discuss limitations at study and outcome level (e.g., risk of bias), and at review-level (e.g., incomplete retrieval of identified research, reporting bias).	15
Conclusions	26	Provide a general interpretation of the results in the context of other evidence, and implications for future research.	16
**FUNDING**			
Funding	27	Describe sources of funding for the systematic review and other support (e.g., supply of data); role of funders for the systematic review.	16

From: Moher D, Liberati A, Tetzlaff J, Altman DG, The PRISMA Group (2009). Preferred Reporting Items for Systematic Reviews and Meta-Analyses: The PRISMA Statement. PLoS Med 6(7): e1000097. doi:10.1371/journal.pmed1000097

For more information, visit: **www.prisma-statement.org**.

For peer review only

For peer review only - http://bmjopen.bmj.com/site/about/guidelines.xhtml
